# Enantioselective Synthesis of Oxazocines via MQ‐Phos Enabled Palladium‐Catalyzed Asymmetric Formal [4+4]‐Cycloadditions

**DOI:** 10.1002/advs.202402170

**Published:** 2024-06-17

**Authors:** Qiaojing Meng, Yinggao Meng, Qinglin Liu, Bing Yu, Zhong‐Jun Li, Er‐Qing Li, Junliang Zhang

**Affiliations:** ^1^ College of Chemistry Green Catalysis Center Zhengzhou University Zhengzhou 450001 P. R. China; ^2^ College of Chemical and Environmental Engineering Hanjiang Normal University Shiyan 442000 P. R. China; ^3^ Department of Chemistry Fudan University Shanghai 200438 P. R. China

**Keywords:** [4+4]‐cycloaddition, enantioselectivity, medium‐sized ring, MQ‐Phos ligand, palladium catalysis

## Abstract

Oxazocines are key structural intermediates in the synthesis of natural products and pharmaceutical molecules. However, the synthesis of oxazocines especially in a highly enantioselective manner, is a long‐standing formidable challenge due to unfavorable energetics involved in cyclization. Herein, a series of new PNP‐Ligand *P*‐chiral stereocenter is first designed and synthesized, called **MQ‐Phos**, and successfully applied it in the Pd‐catalyzed enantioselective higher‐order formal [4+4]‐cycloaddition of *α*, *β*‐unsaturated imines with 2‐(hydroxymethyl)‐1‐arylallyl carbonates. The reaction features mild conditions, excellent regio‐ and enantiocontrol and a broad substrate scope (54 examples). Various medium‐sized rings can be afforded in moderate to excellent yields (up to 92%) and excellent enantioselectivity (up to 99% ee). The newly developed **MQ‐Phos** is critical for synthesis of the medium‐sized ring in excellent catalytic reactivity and enantioselectivity.

## Graphical abstract

A series of new PNP‐Ligands *P*‐chiral stereocenter, called MQ‐Phos, are designed and successfully applied it in the Pd‐catalyzed enantioselective higher‐order formal [4+4]‐cycloaddition of *α*, *β*‐unsaturated imines with 2‐(hydroxymethyl)‐1‐arylallyl carbonates.

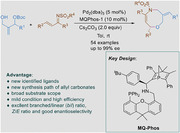

## 1 Introduction

Medium‐sized rings are common core structures of natural products and bioactive molecules.^[^ ^1^ ^]^ Particularly, oxazocine skeletons are commonly present in bioactive molecules such as Nefopam (nonopioid analgesic drug), Otonecine (hepatotoxic activity), ML341 (trypanocidal activity), and bremazocine (ĸ‐opioid receptor (KOR) agonists).^[^ ^2^ ^]^ At present, the available strategies for the synthesis of medium‐sized rings mainly include intramolecular cyclization, intermolecular higher‐order cycloaddition, multicomponent cyclization, and ring expansion. However, due to the presence of competitive reaction pathways and low levels of site‐ and stereoselectivity, the development of a general, efficient access to medium‐sized rings by chemical synthesis, particularly in an asymmetric catalytic manner is quite challenging.

**Scheme 1:**
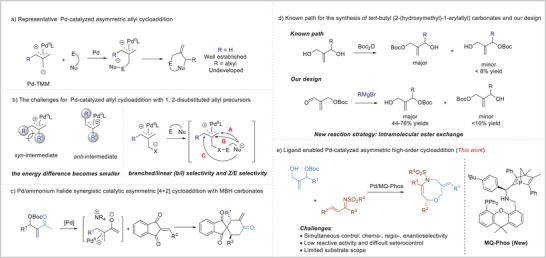
Background and Strategy.

Pd‐trimethylenemethane (TMM) cycloaddition reactions have evolved into a versatile tool for the construction of carbo‐ and heterocycles with rich stereochemistry.^[^ ^3^ ^]^ In the field, various zwitterion precursors that contain different nucleophilic moieties have been developed by Trost,^[^ ^4^ ^]^ Chen,^[^ ^5^ ^]^ Zi,^[^ ^6^ ^]^ Guo,^[^ ^7^ ^]^ Deng,^[^ ^8^ ^]^ Li,^[^ ^9^ ^]^ and other groups in the past decades. However, almost all these reactions depend on the non‐substituted alkyl carbonates as zwitterion precursors, and the substitution patterns of the TMM moiety are relative underuse (**Scheme** **1**a). As early as 1971, Faller explained the reason: when an extra 2‐alkyl group was introduced to allylic substrates, both the *syn*‐ and the *anti*‐isomer existed in solutions of certain 1, 2‐disubstituted‐π‐allylmetal complexes.^[^ ^10^ ^]^ Due to steric repulsion between the R^1^ and R' groups of 1, 2‐disubstituted‐*π*‐allylmetal complexes, the energy difference between its *syn* and *anti* isomer became smaller. Moreover, a challenging dynamic kinetic asymmetric transformation process was presented after the introduction of the 2‐R' group. Besides, the regioselectiviy of the three different reactive sites on the Pd‐π‐allyl intermediate and resultant the problem of *Z*/*E* control also made it more difficult to get high efficiency and stereoselectivity (Scheme 1b).^[^ ^11^ ^]^ To the best of our knowledge, the only successful example was realized by Chen and coworkers through a new double activation mode combining an achiral palladium complex and a chiral ammonium halide as an ion‐pair catalyst, enabling the asymmetric [4+2] annulations of MBH adducts with diverse activated alkenes (Scheme 1c).^[^ ^12^ ^]^ Recently, Lu,^[^ ^13^ ^]^ Shibata^[^ ^14^ ^]^ and Huang^[^ ^15^ ^]^ respectively reported Pd‐catalyzed enantio‐, diastereo‐, and regioselective [4+2] cycloadditions by using substituted‐2‐alkylidenetrimethylene carbonates. However, due to the unfavorable transannular interactions and entropic factors, these achievements are largely limited to the construction of five‐ or six‐membered rings, the synthesis of enantioenriched medium‐sized rings through asymmetric higher‐order cycloaddition remains rare in the literature. Thus the development of new efficient catalyst systems and allylic precursors for the preparation of medium‐sized rings is highly desired.

Li group has been devoted to the development of new *P*‐chiral ligands derived from 1‐phosphanorbornenes and apply them to metal‐catalyzed asymmetric cycloadditions.^[^ ^16^ ^]^ Their excellent regio‐, chemo‐ and stereo‐control ability prompt us to investigate the more challenging asymmetric high‐order cycloaddition of 1, 2‐disubstituted‐*π*‐allyl precursors with *α*, *β*‐unsaturated imines. To achieve this goal, we selected *tert*‐butyl (2‐(hydroxymethyl)−1‐arylallyl) carbonates as 1, 2‐disubstituted‐*π*‐allyl precursors to produce the optically active substituted oxazocines. However, several challenges would be encountered in this scenario: 1) *tert*‐butyl (2‐(hydroxymethyl)−1‐arylallyl) carbonates were obtained in <8% yields by using the known methods.^[^ ^17^ ^]^ Thus a new synthesis path should be explored to give higher yield. 2) The second challenging part was finding ideal ligands for the construction of oxazocines with complete *Z/E* selectivity, *b*/*l* selectivity, and high enantioselectivity. As far as we know, the synthesis of target chiral oxazocines could not be achieved by the asymmetric high‐order cycloaddition reactions using the corresponding *tert*‐butyl (2‐(hydroxymethyl)−1‐arylallyl) or the chiral catalysts based on known chiral ligands, which demonstrated the uniqueness of this strategy. 3) In most cases, the substrate scope was quite limited for the synthesis of medium‐sized rings. To circumvent these issues, we first designed an intramolecular ester exchange strategy to form the *tert*‐butyl (2‐(hydroxymethyl)−1‐arylallyl) carbonates (*see* Supporting Information *in detail*), which were obtained in 44–76% yields (Scheme 1d). Second, we designed a kind of new chiral PNP‐ligands based on 1‐phosphanorbornenes and (9, 9‐dimethyl‐9*H*‐xanthen‐4‐yl)diphenylphosphane (**MQ‐Phos**), which could be used in highly regio‐ and enantioselective high‐order cycloaddition of 1, 2‐disubstituted allylic carbonates. Chiral oxazocines could be obtained in up to 92% yield with >20:1 branched/linear (*b*/*l*) ratio, >20:1 *Z*/*E* ratio and 99% ee (Scheme 1e).

## 2 Results and Discussion

**Scheme 2:**
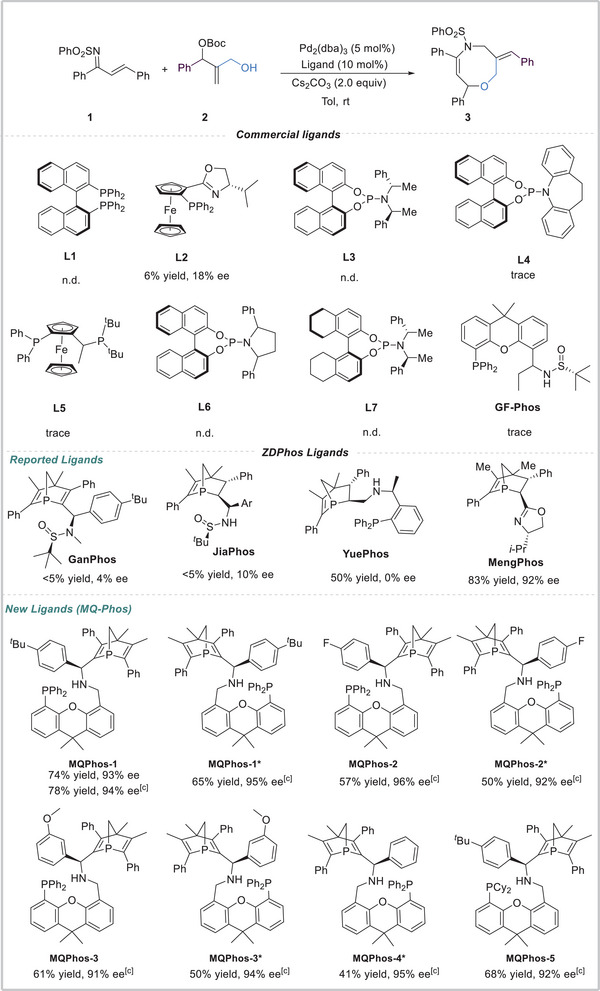
Screening of commercial ligands and optimization of the **ZD‐Phos** ligands(a),(b). a) Reaction conditions: **1** (0.1 mmol), **2** (0.2 mmol), Pd_2_(dba)_3_ (5 mol %), L* (10 mol %), Cs_2_CO_3_ (2.0 equiv), toluene (1 mL), N_2_, rt, 48 h. b) Isolated yield. c) Reaction temperature: 40 °C.

Initially, we employed N‐((1*E*, 2*E*)−1, 3‐diphenylallylidene)benzenesulfonamide **1** and *tert*‐butyl (2‐(hydroxymethyl)−1‐phenylallyl) carbonate **2** as model substrates to investigate various chiral ligands (**Scheme** **2**), bases, palladium sources, solvents, and run the reactions at different temperatures. First, different representative ligands (**L1**‐**L7**) were tested, most of them could not produce the desired product **3**, and only ligand **L2** was able to give the product in 6% yield with 18% ee, which confirmed the less reactivity of racemic 1, 2‐disubstituted allylic products. Next, **GF‐Phos**, which performed well in palladium‐catalyzed allylation, was used as a chiral ligand, unfortunately, only trace product **3** was obtained.^[^ ^18^ ^]^ Thus we checked the efficiency of a series of **ZD‐Phos** ligands that we developed. Using **Gan‐Phos**, **Jia‐Phos**, and **Yue‐Phos** as the chiral ligand did not furnish satisfactory results. When **Meng‐Phos** was tested in toluene at rt, to our delight, the desired product **3** was obtained in 83% yield with 92% ee, while Pd/**Meng‐Phos** complex showed poor substrate tolerance, only a handful of products **3** were obtained with high enantioselectivity. It was possible that extremely twisted conformations of eight‐member cycle resulted in the difficulty in stereocontrol. Thus we tested our newly designed chiral ligands **MQ‐Phos** and discovered **MQ‐Phos**, which was able to achieve a higher yield and excellent ee (74% yield, 93% ee), slightly higher yield was produced by appropriately raising the temperature (78% yield, 94% ee). The structure and configuration of **MQ‐Phos** were unambiguously determined via X‐ray diffraction analysis.^[^ ^19^ ^]^ Interestingly, all **MQ‐Phos** performed well in our palladium‐catalyzed [4+4] cycloaddition, the desired product **3** was obtained in 41–78% yields with 91–96% ee. Overall consideration, we selected **MQPhos‐1** as the ligand to screen other conditions (*See the Supporting Information* (*SI*) *in detail*). Eventually, the optimal conditions for the reaction were identified as *N*‐((1*E*, 2*E*)−1, 3‐diphenylallylidene)benzenesulfonamide (1.0 equiv), *tert*‐butyl (2‐(hydroxymethyl)−1‐phenylallyl) carbonate (2.0 equiv), Cs_2_CO_3_ (2.0 equiv), Pd_2_(dba)_3_ (5 mol%), **MQPhos‐1** (10 mol%), and toluene as the solvent at 40 °C (Scheme 2).

**Scheme 3:**
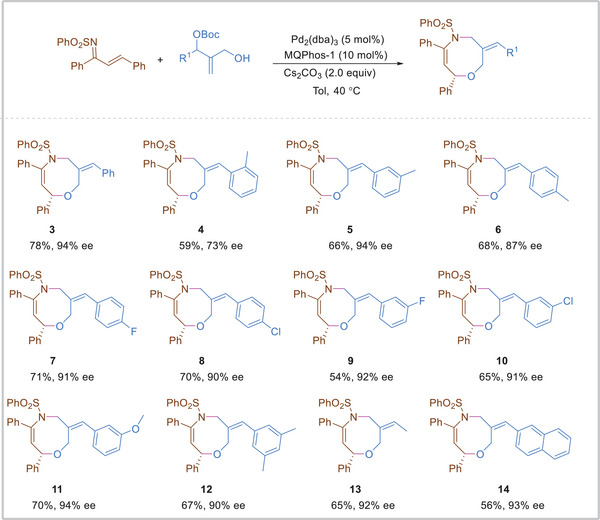
Substrate scope of different 2‐(hydroxymethyl)−1‐arylallyl carbonates (a),(b). a) Reaction conditions: **1a** (0.1 mmol), **2** (0.2 mmol), Pd_2_(dba)_3_ (5 mol %), **MQPhos‐1** (10 mol %), Cs_2_CO_3_ (2.0 equiv), toluene (1 mL), N_2_, 40 °C, 48 h. b) Isolated yield.

Under the optimal conditions in hand, the substituent effect on the aryl groups of different 1,2‐disubstituted allyl carbonates was first investigated, which was often relatively less reactive than those unsubstituted 2‐(hydroxymethyl)allyl) carbonate for the asymmetric cycloaddition. As outlined in **Scheme** **3**, electron‐donating, Electron‐neutral, and electron‐withdrawing groups could be tolerated at the *ortho*‐, *meta*‐, *para*‐position of the allyl carbonates, the desired products **3**–**12** were obtained in 54–78% yields with 73–94% ee. Obviously, the substituent position of 1,2‐ disubstituted allyl carbonates played the key role in the control of enantioselectivity, a high enantioselectivity was obtained when *meta*‐position of the allyl carbonates was used in the reaction. The 1,2‐disubstituted allyl carbonates were further examined, interestingly, the 1,2‐disubstituted allyl carbonates with a small hindered methyl group reacted smoothly to give the corresponding oxazocine in 65% yield with 92% *ee* (**13**). Besides 2‐naphthyl‐substituted allylic carbonates was also a suitable substrate in this reaction to produce the product **14** in 56% yield with 93% *ee* (**14**).

**Scheme 4:**
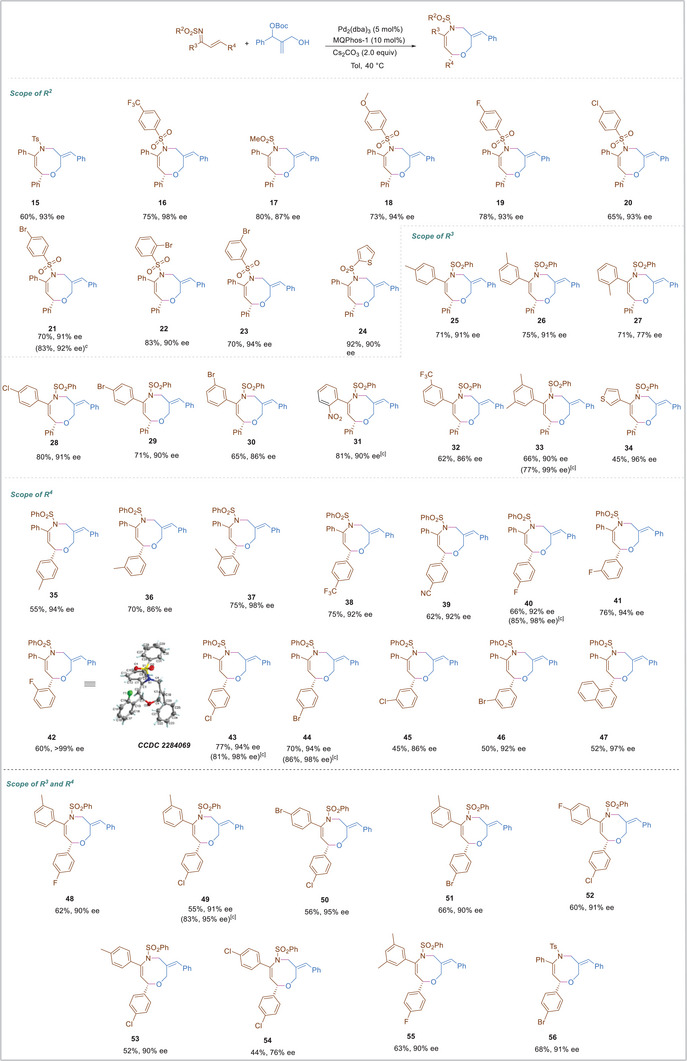
Substrate scope of *α*, *β*‐unsaturated imines(a),(b). a) Reaction conditions: **1** (0.1 mmol), **2** (0.2 mmol), Pd_2_(dba)_3_ (5 mol %), MQPhos‐1 (10 mol %), Cs_2_CO_3_ (2.0 equiv) in toluene (1 mL), N_2_, 40 °C, 48 h. b) Isolated yield. c) **Meng‐Phos** (10 mol%) instead of **MQPhos‐1**.

We next turned to investigate the catalytic Pd‐oxyallyl species with *α*, *β*‐unsaturated imines under standard reaction conditions (**Scheme** **4**). Usually, R^2^ groups of *α*, *β*‐unsaturated imines preformed predominantly in enantiomer control. And only specific R^2^ groups of *α*, *β*‐unsaturated imines could show well stereocontrol in asymmetric cycloaddition. Notably, a wide range of *α*, *β*‐unsaturated imines with electronically varied R^2^ substituents, such as 4‐MeC_6_H_4_, 4‐CF_3_C_6_H_4_, 4‐MeOC_6_H_4_, 4‐FC_6_H_4_, 4‐ClC_6_H_4_, 4‐BrC_6_H_4_, 2‐BrC_6_H_4_ and 3‐BrC_6_H_4_, could readily participate in the [4+4]‐cycloaddition, affording the corresponding oxazocines in 60–83% yields and with 90–98% ee (**15**, **16**, **18**–**23**). Even R^2^ substituents of *α*, *β*‐unsaturated imines were methyl and 2‐thiethyl, The desired oxazocines were prepared with 80% and 92% yields, 87% and 90% ee respectively (**17**, **24**). We next turned to investigate the R^3^ substituents of *α*, *β*‐unsaturated imines under standard reaction conditions. Notably, *α*, *β*‐unsaturated imines with different R^3^ substituents were applicable to the reaction, furnishing the corresponding products **25**–**33** with satisfactory outcomes. It was worth noting that *ortho*‐position of R^3^ of *α*, *β*‐unsaturated imine was applied, only 77% ee was obtained, which might be due to steric hindrance. The *α*, *β*‐unsaturated imine with 3‐thienyl could participate in the present cycloaddition to give enantioenriched oxazocine in 45% yield with 96% *ee* (**34**). Next, we turned to investigate the R^4^ substituents of *α*, *β*‐unsaturated imines. The cycloaddition was also readily scalable, The *α*, *β*‐unsaturated imines with different R^4^ aromatic motifs could all participate in the present cycloaddition to afford the desired oxazocines in 45–75% yields with 86→99% ee (**35**‐**47**). In addition, the *α*, *β*‐unsaturated imines with different R^3^ and R^4^ groups proceeded smoothly in the cycloaddition to give 52−63% yields of **48**–**55** with 90−95% ee. Unfortunately, when R^3^ and R^4^ were 4‐Cl group, slightly lower yield and enantioselectivity were obtained (**54**). The *α*, *β*‐unsaturated imines with different R^2^ and R^4^ groups proceeded smoothly in the cycloaddition to give 68% yields of **56** with 91% ee. The structure and configuration of **3** and **42** were unambiguously determined via X‐ray diffraction analysis (*See the Supporting Information* (*SI*) *in details*).^[^ ^20^ ^]^

**Scheme 5:**
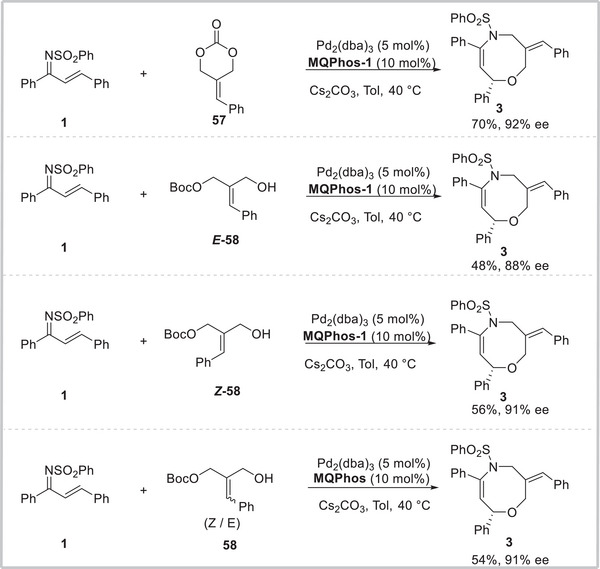
The effect of *E*/*Z* allyl carbonates geometric isomers.

To gain insights into the effect of *E*/*Z* allyl carbonates geometric isomers on the steric control of the reaction, other types of allyl carbonates were then carried out (**Scheme** **5**).^[^ ^21^ ^]^ When 2‐benzylidenetrimethylene carbonate **57**, prepared by Hayashi,^[^ ^22^ ^]^ was used as an allyl precursor, the desired **3** was obtained in 70% yield with 92% ee. Next, we chose allyl carbonates ***E*‐58** and ***Z*‐58** as allyl precursors, the [4+4]‐cycloaddition proceeded smoothly to lead to **3** as the sole product in 48% and 56% yields with 88% and 91% ee, respectively. Even the mixed *Z*/*E* isomers **58** were used under the standard conditions, the desired oxazocine **3** as the sole product was still produced in 54% yield with 91% ee.^[^ ^23^ ^]^ These results suggested that Pd/MQ‐Phos complex preformed excellent abilities in *Z*/*E*, *b*/*l*, and enantio‐control.

**Scheme 6:**
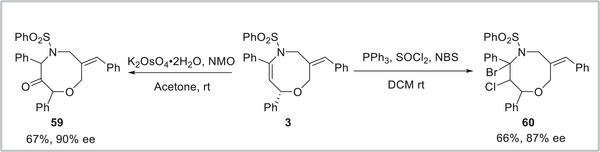
Synthetic transformations.

To demonstrate the practicality of our approach, some further synthetic transformations of **3** were then carried out (**Scheme** **6**). First, oxidation of **3** using K_2_OsO_4_•2H_2_O and NMO was studied, to our delight, the desired adduct **59** was obtained as a single diastereomer in 67% yield with 90% ee. Then, the halogenation of **3** led to the desired compound **60** in 66% yield with 87% ee.

## 3 Conclusion

In conclusion, we have developed a highly regio‐, chemo‐, and enantioselective palladium‐catalyzed asymmetric high‐order [4+4]‐cycloaddition reaction of 1,2‐disubstituted allylic carbonates with a variety of *α*, *β*‐unsaturated imines, which provided an efficient method for the synthesis of oxazocine compounds. The salient features of the method include high efficiency, mild reaction conditions, simple operation, and excellent *Z*/*E*‐, *b*/*l*, and enantioselectivity. A rationally designed **MQ‐Phos** ligand played a critical role in the reaction efficiency and selectivity. Further studies including the allocation of **MQ‐Phos** in asymmetric palladium catalysis, especially the tandem cycloaddition reactions are underway and will be reported in due course.

## Conflict of Interest

The authors declare no conflict of interest.

## Supporting information



Supporting Information

## Data Availability

The data that support the findings of this study are available in the supplementary material of this article.
