# Supplementary material for: Enantioselective Synthesis of Oxazocines via MQ‐Phos Enabled Palladium‐Catalyzed Asymmetric Formal [4+4]‐Cycloadditions
Source: Adv Sci (Weinh). 2024 Jun 17;11(31):2402170. doi: 10.1002/advs.202402170 (PMC11336936; doi:10.1002/advs.202402170)
Supplement: Supplementary file 1 — Supporting Information [file ADVS-11-2402170-s001.docx]

***Supporting Information***

**Enantioselective Synthesis of Oxazocines *via* MQ-Phos Enabled Palladium-Catalyzed Asymmetric Formal [4+4]-Cycloadditions**

Qiaojing Meng,^†,[a]^ Yinggao Meng,^†,[a],[c]^ Qinglin Liu,^[a]^ Bing Yu,^[a]^ Zhong-Jun Li^[a]^ Er-Qing Li*^,[a]^ and Junliang Zhang*^,[b]^

[a] College of Chemistry, Green Catalysis Center, Zhengzhou University, Zhengzhou 450001, P. R. China.

[b] Department of Chemistry, Fudan University, Shanghai 200438, P. R. China

[c] College of Chemical and Environmental Engineering, Hanjiang Normal University, Shiyan, 442000, China.

**目录**

**1. General information** S1

**2. Experimental procedures** S2

**3. Reaction Optimizations** S17

**4. General Procedure for Reactions** S20

**5. Copies of ^31^P NMR, ^1^H NMR, ^13^C NMR spectra** S65

**6. Copies of HPLC Chromatograms** S134

**7. X-ray crystal structures** S134

**1. General information**

All commercially available reagents were used without further purification. Solvents were treated prior to use according to the standard methods. All reactions were performed under nitrogen using solvents dried by standard methods. NMR spectra were obtained using Bruker spectrometer. Chemical shifts are expressed in parts per million (ppm) downfield from internal TMS. ^1^H and ^13^C chemical shifts are reported in ppm relative to either the residual solvent peak (^13^C) or tetramethylsilane (*δ* = 0 ppm) as an internal standard. HRMS spectra were obtained on an Agilent 1290-6540 UHPLC Q-Tof HR-MS spectrometer. X-ray crystallographic analyses were performed on an Oxford diffraction Gemini E diffractometer. Melting Point: heating rate: 4°C/min, the thermometer was not corrected. Enantiomer excesses were determined by chiral HPLC analysis on Chiralcel IA/IG in comparison with the authentic racemates. Chiral HPLC analysis recorded on Shanghaiyice instruments and Equipment Co. Ltd. and Shimadzu LC-20A. Silica gel (200-300 mesh) was used for the chromatographic separations.

**2. Experimental procedures**

**General procedure for the preparation of chiral ligands**

Phenylacetylene (11 mL, 100 mmol, 1.0 equiv) was dissolved in THF (160 mL) and placed at - 40 ºC in a nitrogen atmosphere. *n*-BuLi (62.5 mL, 1.6 mol/L, 100 mmol, 1.0 equiv) was slowly added dropwise. After reaction for 2 hours, DMF (15.4 mL, 200 mmol, 2.0 equiv) was added for reaction for 2~3 hours, and the reaction was quenched with a saturated KH_2_PO_4_ solution. The organic layer was removed, and the aqueous layer was extracted twice with AcOEt. The combined organic layers were dried over MgSO_4_, filtered, concentrated to obtain crude product **S1**, which can be directly used for the next reaction without purification.

Compound **S1** (6.5 g, 50 mmol, 1.0 equiv) was dissolved in CH_3_OH (30 mL), followed by the addition of trimethyl orthoformate (10.6 g, 100 mmol, 2.0 equiv) and TsOH·H_2_O (190 mg, 1 mmol, 0.02 equiv). The product was refluxed at 60 ºC for 4 hours, and then was purified by flash column chromatrography directly (petroleum ether : AcOEt = 80 : 1) to afford 6.2 g the product **S2** (70% yield).

A mixture of compound **S2** (4.6 g, 46 mmol, 1.3 equiv) and 3, 4-dimethyl-1-phenyl-1H-phosphole (3.8 g, 20 mmol, 1.0 equiv) was heated at 150 °C for 4 h. The progress of the reaction was followed by ^31^P NMR. After concentration, the crude product was obtained. Subsequently, the crude product was dissolved in DCM (50 mL) at 0 ºC, and CF_3_COOH (3.0 mL, 2.0 equiv) was added dropwise for overnight reaction. The product was purified by column chromatography (petroleum ether : AcOEt = 80 : 1) to afford 5.7 g the product **S3** (90% yield).

A mixture of compound **S3** (1.6 g, 5 mmol, 1.0 equiv) and (*R*)-*tert*-butyl sulfinyl amide (726 mg, 6 mmol, 1.2 equiv) and Ti(O*^i^*Pr)_4_ (3.6 g, 12.5 mmol, 2.5 equiv) was refluxed in THF at 70 °C for 3 h, and the reaction was quenched with H_2_O. The organic layer was removed, and the aqueous layer was extracted twice with AcOEt. The combined organic layers were dried over MgSO_4_, filtered, concentrated, and purified by flash chromatography (petroleum ether : AcOEt = 5 : 1) afforded the product **S4** (82% yield).

To a solution of **S4** (1.4 g, 3 mmol, 1.0 equiv) in THF at 0 °C was added a solution of 4-*tert*-butyl phenyl magnesium bromide (1.4 g, 6 mmol, 2.0 equiv) in THF. Stirring was continued at 0 °C for 0.5 h. When completed, the reaction mixture was quenched by the addition of H_2_O and diluted with AcOEt. The organic layer was removed, and the aqueous layer was extracted twice with AcOEt. The combined organic layers were dried over MgSO_4_, filtered, concentrated, and purified by flash chromatography (petroleum ether : AcOEt = 4 : 1) afforded the product **S5a** (36% yield) and **S5a*** (49% yield).^[1]^

Compound **S5a** (400 mg) was dissolved in CH_3_OH (30 mL), and HCl (1.0 mL) was added dropwise. After continuous stirring for 2 hours, a saturated NaHCO_3_ aqueous solution was added to quench the reaction. The organic layer was removed, and the aqueous layer was extracted twice with AcOEt. The combined organic layers were dried over MgSO_4_, filtered, concentrated to obtain crude product **S6a**, which can be directly used for the next reaction without purification.

To a solution of 9, 9' - dimethyloxanthracene (2.1 g, 10 mmol, 1.0 equiv) dissolved in Et_2_O was added TMEDA (2.3 g, 20 mmol, 2.0 equiv) at 0 ºC, and slowly added *n*-BuLi (19 mL, 1.6 M, 30 mmol, 3.0 equiv) dropwise. Subsequently, the reaction was placed at 40 ºC for 4 hours, and after completion, it was cooled to 0 ºC. Then, a THF solution of I_2_ substance (6.4 g, 25 mmol, 2.5 equiv) was slowly added, and the reaction lasted overnight. After the reaction was completed, quenched the reaction with saturated Na_2_S_2_O_3_ aqueous solution. The organic layer was removed, and the aqueous layer was extracted twice with AcOEt. The combined organic layers were dried over MgSO_4_, filtered, concentrated, and purified by flash chromatography (petroleum ether : AcOEt = 10 : 1) afforded the product **S7** (61% yield).

Compound **S7** (4.6 g, 10 mmol, 1.0 equiv) was dissolved in THF and placed at - 78 ºC. *n*-BuLi (4 mL, 2.5 M, 10 mmol, 1.0 equiv) was added dropwise, and PPh_2_Cl (1.8 mL, 10 mmol, 1.0 equiv) was slowly added after continuous stirring for 1.5 hours. *n*-BuLi (8 mL, 2.5 M, 20 mmol, 2.0 equiv) was added after continuous reaction for 1 hour. After reaction for 1.5 hours, DMF (2.5 mL, 20 mmol, 2.0 equiv) was added, and after stirring for 2 hours, the reaction was quenched with H_2_O. The organic layer was removed, and the aqueous layer was extracted twice with AcOEt. The combined organic layers were dried over MgSO_4_, filtered, concentrated, and purified by flash chromatography (petroleum ether : AcOEt = 15 : 1) afforded the product **S8** (60% yield).^[2]^

A mixture of **S6a** and compound **S8** (400 mg) and anhydrous MgSO_4_ refluxed in methanol at 70 ℃ for 6 hours. After the reaction, DCM (10 mL) and NaBH_4_ (114 mg, 3.0 equiv) were added for 2 hours. Then was purified by flash column chromatrography directly to afford the corresponding product **MQ Phos-1** (51% yield).

**MQ Phos-1**

**(*S*)-1-(4-(*tert*-butyl)phenyl)-1-((1*R*, 4*S*)-4, 5-dimethyl-3, 6-diphenyl-1-phosphabicyclo[2.2.1]hepta-2, 5-dien-2-yl)-*N*-((5-(diphenylphosphaneyl)-9, 9-dimethyl-9*H*-xanthen-4-yl)methyl)methanamine**

White solid. **Yield:** 51% yield. **MP:** 89.7 - 90.7 ºC. [α]_D_ = + 18 (*c* = 0.1, CH_2_Cl_2_, 20.2 ℃). **^31^P NMR (243 MHz, CDCl_3_)** *δ* - 17.74, - 20.70 ppm. **^1^H NMR (400 MHz, CDCl_3_)** *δ* 7.63 (dd, *J* = 21.2, 8.0 Hz, 4H), 7.50 - 7.37 (m, 6H), 7.35 - 7.26 (m, 10H), 7.24 - 7.18 (m, 4H), 7.13 (t, *J* = 7.3 Hz, 2H), 7.04 (t, *J* = 7.7 Hz, 1H), 6.76 (t, *J* = 7.6 Hz, 1H), 6.62 (dd, *J* = 6.3, 4.0 Hz, 1H), 6.38 (d, *J* = 7.0 Hz, 1H), 4.62 (d, *J* = 10.4 Hz, 1H), 3.33 (d, *J* = 13.6 Hz, 1H), 3.19 (d, *J* = 13.6 Hz, 1H), 2.33 (s, 3H), 2.06 (dd, *J* = 9.7, 6.0 Hz, 2H), 1.66 (d, *J* = 12.9 Hz, 6H), 1.47 (s, 9H), 1.43 (s, 3H) ppm. **^13^C NMR** **(101 MHz, CDCl_3_)** *δ* 161.4, 157.9, 157.6, 157.3, 151.7, 151.6, 149.5, 149.3, 149.2, 148.0, 140.9, 139.6, 139.3, 138.4, 136.6, 136.5, 136.4, 136.3, 134.3, 134.1, 133.9, 131.2, 129.8, 129.1, 128.7, 128.6, 128.5, 128.4, 128.37, 128.24, 128.19, 127.98, 127.93, 127.72, 127.00, 126.69, 126.06, 125.60, 125.5, 125.2, 123.9, 123.2, 122.6, 70.6, 65.2, 61.1, 60.9, 46.5, 34.6, 34.2, 32.9, 31.7, 21.1, 16.0 ppm. **HRMS** (ESI) (m/z) [M+H]^+^ Calcd for C_59_H_58_NOP_2_^+^ 858.3988; found 858.3992.

**MQ Phos-1^*^**

**(1*R*)-1-(4-(*tert*-butyl)phenyl)-1-((1*S*)-4, 5-dimethyl-3, 6-diphenyl-1-phosphabicyclo[2.2.1]hepta-2, 5-dien-2-yl)-*N*-((5-(diphenylphosphaneyl)-9, 9-dimethyl-9*H*-xanthen-4-yl)methyl)methanamine**

White solid. **Yield:** 67% yield. **MP:** 93.3 - 94.3 ºC. [α]_D_ = - 80 (*c* = 0.1, CH_2_Cl_2_, 18.5 ℃). **^31^P NMR (243 MHz, CDCl_3_)** *δ* - 17.76, - 20.72. **^1^H NMR (400 MHz, CDCl_3_)** *δ* 7.58 (dd, *J* = 23.6, 8.0 Hz, 4H), 7.45 - 7.33 (m, 7H), 7.31 - 7.27 (m, 5H), 7.25 - 7.15 (m, 8H), 7.08 (t, *J* = 7.3 Hz, 2H), 7.01 (t, *J* = 7.6 Hz, 1H), 6.71 (t, *J* = 7.6 Hz, 1H), 6.60 - 6.55 (m, 1H), 6.33 (d, *J* = 7.0 Hz, 1H), 4.57 (d, *J* = 10.4 Hz, 1H), 3.28 (d, *J* = 13.7 Hz, 1H), 3.14 (d, *J* = 13.6 Hz, 1H), 2.28 (s, 3H), 2.02 (dd, *J* = 9.7, 6.0 Hz, 2H), 1.62 (d, *J* = 13.3 Hz, 6H), 1.43 (s, 9H), 1.39 (s, 3H). **^13^C NMR (101 MHz, CDCl_3_)** *δ* 161.3, 157.8, 157.5, 157.3, 151.7, 151.5, 149.4, 149.3, 149.2, 148.0, 140.8, 139.5, 139.3, 138.4, 136.5, 136.4, 136.4, 136.2, 134.2, 134.0, 133.8, 131.1, 129.8, 129.0, 128.6, 128.5, 128.4, 128.3, 128.2, 128.1, 127.9, 127.7, 126.9, 126.6, 126.0, 125.6, 125.4, 125.2, 123.9, 123.1, 122.5, 70.6, 70.6, 65.2, 61.0, 60.9, 46.4, 34.5, 34.1, 32.8, 31.7, 31.6, 21.0, 15.9. **HRMS** (ESI) (m/z) [M+H]^+^ Calcd for C_59_H_58_NOP_2_^+^ 858.3988; found 858.3989.

**MQ Phos-2**

**(*S*)-1-((1*R*, 4*S*)-4,5-dimethyl-3, 6-diphenyl-1-phosphabicyclo[2.2.1]hepta-2, 5-dien-2-yl)-*N*-((5-(diphenylphosphaneyl)-9, 9-dimethyl-9*H*-xanthen-4-yl)methyl)-1-(4-fluorophenyl)methanamine**

White solid. **Yield:** 50% yield. **MP:** 88.6 - 89.6 ºC. [α]_D_ = + 85 (*c* = 0.1, CH_2_Cl_2_, 20.6 ℃). **^31^P NMR (243 MHz, CDCl_3_)** *δ* - 17.53, - 21.10 ppm. **^1^H NMR (400 MHz, CDCl_3_)** *δ* 7.61 - 7.51 (m, 4H), 7.45 - 7.25 (m, 10H), 7.24 - 7.07 (m, 8H), 7.08 - 6.96 (m, 5H), 6.71 (t, *J* = 7.6 Hz, 1H), 6.55 (ddd, *J* = 7.5, 4.0, 1.4 Hz, 1H), 6.29 (dd, *J* = 7.4, 1.1 Hz, 1H), 4.51 (d, *J* = 10.2 Hz, 1H), 3.21 (d, *J* = 13.6 Hz, 1H), 3.15 (d, *J* = 13.6 Hz, 1H), 2.22 (s, 3H), 2.00 (t, *J* = 9.7 Hz, 1H), 1.89 (t, *J* = 9.7 Hz, 1H), 1.59 (d, *J* = 17.7 Hz, 6H), 1.34 (s, 3H) ppm. **^13^C NMR (101 MHz, CDCl_3_)** *δ* 163.0, 161.7, 160.6, 157.6, 157.5, 157.3, 151.6, 151.4, 149.2, 148.9, 148.0, 139.5, 139.4, 139.2, 138.3, 136.4, 136.3, 136.2, 136.1, 134.1, 133.9, 133.7, 131.1, 129.7, 129.6, 129.5, 129.1, 128.6, 128.5, 128.4, 128.3, 128.2, 127.9, 127.6, 127.0, 126.7, 126.1, 125.3, 125.2, 124.1, 123.2, 122.5, 115.0, 114.8, 70.7, 70.6, 65.4, 60.8, 60.7, 46.4, 34.1, 32.8, 31.6, 20.8, 15.9 ppm. **HRMS** (ESI) (m/z) [M+H]^+^ Calcd for C_55_H_49_FNOP_2_^+^ 820.3268; found 820.3271.

**MQ Phos-2^*^**

**(1*R*)-1-((1*S*)-4, 5-dimethyl-3, 6-diphenyl-1-phosphabicyclo[2.2.1]hepta-2, 5-dien-2-yl)-*N*-((5-(diphenylphosphaneyl)-9, 9-dimethyl-9*H*-xanthen-4-yl)methyl)-1-(4-fluorophenyl)methanamine**

White solid. **Yield:** 61% yield. **MP:** 89.3 - 90.3 ºC. [α]_D_ = - 42 (*c* = 0.1, CH_2_Cl_2_, 20.8 ℃). **^31^P NMR (243 MHz, CDCl_3_)** δ - 17.53, - 21.10 ppm. **^1^H NMR (400 MHz, CDCl_3_)** *δ* 7.61 - 7.53 (m, 4H), 7.45 - 7.25 (m, 10H), 7.23 - 7.09 (m, 8H), 7.09 - 6.97 (m, 5H), 6.72 (t, *J* = 7.6 Hz, 1H), 6.56 (ddd, *J* = 7.4, 4.0, 1.4 Hz, 1H), 6.31 (dd, *J* = 7.4, 1.0 Hz, 1H), 4.52 (d, *J* = 10.2 Hz, 1H), 3.23 (d, *J* = 13.6 Hz, 1H), 3.16 (d, *J* = 13.6 Hz, 1H), 2.23 (s, 3H), 2.01 (t, *J* = 9.7 Hz, 1H), 1.90 (t, *J* = 9.7 Hz, 1H), 1.60 (d, *J* = 17.5 Hz, 6H), 1.35 (s, 3H) ppm. **^13^C NMR (101 MHz, CDCl_3_)** *δ* 163.0, 161.7, 160.6, 157.6, 157.5, 157.3, 151.6, 151.5, 149.2, 149.0, 148.0, 139.5, 139.2, 138.3, 136.3, 134.1, 133.9, 133.7, 131.1, 129.5, 129.1, 128.6, 128.5, 128.4, 128.3, 128.2, 127.9, 127.6, 127.0, 126.7, 126.1, 125.2, 124.1, 123.2, 122.5, 115.1, 114.8, 77.4, 77.1, 76.8, 70.7, 70.6, 65.4, 60.9, 46.4, 34.1, 32.9, 31.6, 20.9, 15.9 ppm. **HRMS** (ESI) (m/z) [M+H]^+^ Calcd for C_55_H_49_FNOP_2_^+^ 820.3268; found 820.3272.

**MQ Phos-3**

**(*S*)-1-((1*R*, 4*S*)-4,5-dimethyl-3, 6-diphenyl-1-phosphabicyclo[2.2.1]hepta-2, 5-dien-2-yl)-*N*-((5-(diphenylphosphaneyl)-9, 9-dimethyl-9*H*-xanthen-4-yl)methyl)-1-(3-methoxyphenyl)methanamine**

White solid. **Yield:** 57% yield. **MP:** 90.5 - 91.5 ºC. [α]_D_ = + 38 (*c* = 0.1, CH_2_Cl_2_, 18.6 ℃). **^31^P NMR (243 MHz, CDCl_3_)** *δ* - 17.50, - 20.46 ppm. **^1^H NMR (400 MHz, CDCl_3_)** *δ* 7.60 (d, *J* = 7.9 Hz, 2H), 7.44 (d, *J* = 7.7 Hz, 1H), 7.42 - 7.28 (m, 8H), 7.26 - 7.22 (m, 4H), 7.22 - 7.15 (m, 8H), 7.10 - 7.05 (m, 2H), 7.01 (t, *J* = 7.6 Hz, 1H), 6.88 (dd, *J* = 8.0, 2.0 Hz, 1H), 6.75 (t, *J* = 7.6 Hz, 1H), 6.58 (ddd, *J* = 7.5, 4.0, 1.4 Hz, 1H), 6.41 (dd, *J* = 7.4, 1.0 Hz, 1H), 4.54 (d, *J* = 10.1 Hz, 1H), 3.85 (s, 3H), 3.27 (d, *J* = 13.7 Hz, 1H), 3.17 (d, *J* = 13.7 Hz, 1H), 2.25 (s, 3H), 2.03 (t, *J* = 9.7 Hz, 1H), 1.97 (t, *J* = 9.7 Hz, 1H), 1.62 (d, *J* = 11.5 Hz, 6H), 1.37 (s, 3H) ppm. **^13^C NMR (101 MHz, CDCl_3_)** *δ* 161.6, 159.6, 157.6, 157.4, 151.6, 151.5, 149.4, 149.2, 147.9, 145.6, 139.5, 139.3, 138.3, 136.5, 136.4, 136.3, 136.1, 134.2, 134.0, 133.8, 131.1, 129.7, 129.2, 129.1, 128.6, 128.5, 128.4, 128.3, 128.2, 128.1, 127.9, 127.0, 126.7, 126.0, 125.5, 125.3, 124.0, 123.1, 122.5, 120.5, 113.6, 113.5, 112.4, 70.6, 65.3, 61.5, 61.4, 55.2, 46.4, 34.1, 32.8, 31.8, 20.9, 16.0 ppm. **HRMS** (ESI) (m/z) [M+Na]^+^ Calcd for C_56_H_52_NO_2_P_2_^+^ 854.3287; found 854.3284.

**MQ Phos-3^*^**

**(1*R*)-1-((1*S*)-4, 5-dimethyl-3, 6-diphenyl-1-phosphabicyclo[2.2.1]hepta-2, 5-dien-2-yl)-*N*-((5-(diphenylphosphaneyl)-9, 9-dimethyl-9*H*-xanthen-4-yl)methyl)-1-(3-methoxyphenyl)methanamine**

White solid. **Yield:** 64% yield. **MP:** 88.9 - 89.9 ºC. [α]_D_ = - 57 (*c* = 0.1, CH_2_Cl_2_, 19.7 ℃). **^31^P NMR (243 MHz, CDCl_3_)** *δ* - 17.47, - 20.43 ppm. **^1^H NMR (400 MHz, CDCl_3_)** *δ* 7.64 (d, *J* = 7.9 Hz, 2H), 7.47 (d, *J* = 7.7 Hz, 1H), 7.44 - 7.27 (m, 13H), 7.26 – 7.18 (m, 7H), 7.15 - 7.09 (m, 2H), 7.04 (t, *J* = 7.6 Hz, 1H), 6.91 (dd, *J* = 8.0, 2.0 Hz, 1H), 6.79 (t, *J* = 7.6 Hz, 1H), 6.63 (ddd, *J* = 7.4, 4.0, 1.3 Hz, 1H), 6.46 (d, 1H), 4.59 (d, *J* = 10.1 Hz, 1H), 3.88 (s, 3H), 3.32 (d, *J* = 13.7 Hz, 1H), 3.22 (d, *J* = 13.7 Hz, 1H), 2.29 (s, 3H), 2.06 (t, *J* = 9.7 Hz, 1H), 2.01 (t, *J* = 9.7 Hz, 1H), 1.65 (d, *J* = 10.9 Hz, 6H), 1.41 (s, 3H) ppm. **^13^C NMR (101 MHz, CDCl_3_)** *δ* 161.6, 159.7, 157.7, 157.4, 151.7, 151.5, 149.5, 149.2, 148.0 145.6, 139.6, 139.4, 138.4, 136.6, 136.4, 136.3, 136.2, 134.2, 134.1, 134.0, 133.9, 131.2, 129.7, 129.3, 129.1, 128.7, 128.6, 128.5, 128.4, 128.2, 127.9, 127.0, 126.7, 126.1, 125.5, 125.4, 124.0, 123.2, 122.6, 120.5, 113.6, 112.5, 70.7, 70.6, 65.4, 61.6, 61.4, 55.3, 46.4, 34.2, 32.9, 31.9, 21.0, 16.0 ppm. **HRMS** (ESI) (m/z) [M+Na]^+^ Calcd for C_56_H_51_NO_2_P_2_Na^+^ 854.3287; found 854.3283.

**MQ Phos-4^*^**

**(1*R*)-1-((1*S*)-4, 5-dimethyl-3, 6-diphenyl-1-phosphabicyclo[2.2.1]hepta-2, 5-dien-2-yl)-*N*-((5-(diphenylphosphaneyl)-9, 9-dimethyl-9*H*-xanthen-4-yl)methyl)-1-phenylmethanamine**

White solid. **Yield:** 68% yield. **MP:** 87.4 - 88.4 ºC. [α]_D_ = - 33 (*c* = 0.1, CH_2_Cl_2_, 21.3 ℃). **^31^P NMR (243 MHz, CDCl_3_)** *δ* - 17.51, - 20.69 ppm. **^1^H NMR (400 MHz, CDCl_3_)** *δ* 7.58 (t, *J* = 8.1 Hz, 4H), 7.44 - 7.25 (m, 12H), 7.23 - 7.09 (m, 9H), 7.04 (td, *J* = 8.0, 1.2 Hz, 2H), 6.99 (t, *J* = 7.6 Hz, 1H), 6.71 (t, *J* = 7.6 Hz, 1H), 6.54 (ddd, *J* = 7.5, 4.0, 1.4 Hz, 1H), 6.33 (d, *J* = 7.4 Hz, 1H), 4.54 (d, *J* = 10.3 Hz, 1H), 3.23 (d, *J* = 13.7 Hz, 1H), 3.14 (d, *J* = 13.7 Hz, 1H), 2.23 (s, 3H), 1.99 (t, *J* = 9.7 Hz, 1H), 1.92 (t, *J* = 9.7 Hz, 1H), 1.59 (d, *J* = 13.6 Hz, 6H), 1.34 (s, 3H) ppm. **^13^C NMR (101 MHz, CDCl_3_)** *δ* 161.5, 157.7, 157.4, 151.6, 149.3, 149.1, 147.9, 143.8, 139.5, 139.2, 138.3, 136.5, 136.4, 136.2, 136.1, 134.2, 134.0, 133.8, 131.1, 129.7, 129.0, 128.6, 128.5, 128.4, 128.3, 128.2, 128.1, 127.8, 127.0, 126.7, 126.6, 126.0, 125.4, 125.3, 124.0, 123.5, 123.1, 122.5, 70.6, 70.5, 65.3, 61.5, 61.3, 46.3, 34.1, 32.8, 31.7, 20.9, 15.9 ppm. **HRMS** (ESI) (m/z) [M+H]^+^ Calcd for C_55_H_50_NOP_2_^+^ 802.3362; found 802.3366.

**MQ Phos-5**

**(*S*)-1-(4-(*tert*-butyl)phenyl)-*N*-((5-(dicyclohexylphosphaneyl)-9,9-dimethyl-9*H*-xanthen-4-yl)methyl)-1-((1*R*, 4*S*)-4, 5-dimethyl-3, 6-diphenyl-1-phosphabicyclo[2.2.1]hepta-2, 5-dien-2-yl)methanamine**

White solid. **Yield:** 52% yield. **MP:** 91.2 - 92.2 ºC. [α]_D_ = + 127 (*c* = 0.1, CH_2_Cl_2_, 21.0 ℃). **^31^P NMR (162 MHz, CDCl_3_)** *δ* - 19.61, - 21.28 ppm. **^1^H NMR (400 MHz, CDCl_3_)** *δ* 7.72 (d, *J* = 7.9 Hz, 2H), 7.56 (d, *J* = 8.3 Hz, 2H), 7.43 - 7.34 (m, 8H), 7.33 - 7.26 (m, 2H), 7.23 - 7.11 (m, 4H), 7.06 (t, *J* = 7.5 Hz, 1H), 6.69 (t, *J* = 7.6 Hz, 1H), 6.21 (s, 1H), 4.72 (d, *J* = 11.1 Hz, 1H), 3.53 (s, 2H), 2.31 (s, 3H), 2.06 - 1.94 (m, 2H), 1.88 - 1.61 (m, 10H), 1.58 (d, *J* = 4.3 Hz, 6H), 1.55 - 1.47 (m, 2H), 1.39 (s, 3H), 1.36 (s, 9H), 1.21 - 0.81 (m, 10H) ppm. **^13^C NMR (101 MHz, CDCl_3_)** *δ* 161.5, 157.8, 157.5, 157.3, 149.3, 149.1, 149.0, 148.8, 140.9, 139.5, 139.3, 138.4, 130.2, 129.5, 128.7, 128.6, 128.2, 128.1, 127.6, 127.5, 126.9, 126.1, 125.0, 124.0, 123.0, 122.8, 122.2, 70.6, 65.4, 60.8, 47.5, 34.4, 34.3, 32.8, 32.4, 32.3, 32.1, 32.0, 31.5, 30.4, 30.3, 30.2, 30.1, 30.0, 29.9, 29.8, 29.7, 29.0, 29.0, 28.9, 28.8, 28.7, 28.7, 28.4, 27.8, 27.7, 27.6, 27.5, 27.4, 27.3, 27.2, 27.1, 27.0, 26.5, 26.4, 21.0, 16.1 ppm. **HRMS** (ESI) (m/z) [M+H]^+^ Calcd for C_59_H_70_NOP_2_^+^ 870.4927; found 870.4931.

**General procedure for the preparation of substrates**

To a solution of 2-methylene-1,3-propanediol (4.4 g, 50 mmol, 1.0 equiv) in DCM at 0 °C was added DMAP (305 mg, 2.5 mmol, 0.05 equiv), and added (Boc)_2_O (12.0 g, 55 mmol, 1.1 equiv) solution dissolved in DCM dropwise. After half an hour of reaction, the solvent was evaporated under reduced pressure and purified by column chromatography (petroleum ether : AcOEt= 10 : 1) to obtain product **S9** (84% yield).^[3]^

To a solution of compound **S9** (9.5 g, 50 mmol, 1.0 equiv) dissolved in DCM at 0 ℃ was slowly added Dess-Martin periodinane (25.5g, 60 mmol, 1.2 equiv), after half an hour of reaction, the solvent was evaporated under reduced pressure and purified by column chromatography (petroleum ether : AcOEt= 10 : 1) to obtain product **S10** (91% yield).

Compound **S10** (0.9 g, 5 mmol, 1.0 equiv) was dissolved in THF, and grignard reagent (10 mmol, 2.0 equiv) was added dropwise at 0 ℃. After 10 minutes of reaction, H_2_O was added to quench the reaction. The organic layer was removed, and the aqueous layer was extracted twice with AcOEt. concentrated, and purified by flash chromatography (petroleum ether : AcOEt = 10 : 1) afforded the product **2**.

Ethanone (10 mmol, 1.0 equiv) was dissolved in EtOH (20 mL), and a 10% NaOH solution (12mL) was added under intense stirring. After continuous stirring at room temperature for 30 minutes, corresponding aldehydes (10 mmol, 1.0 equiv) was added. After stirring for 2 hours, a large amount of solid precipitates were formed, cooled to 0 ℃, and filtered to remove the solvent to obtain the corresponding chalcone product **S11**. It can be used without further purification.

Chalcone **S11** (5 mmol, 1.0 equiv), sulfonamide (5 mmol, 1.0 equiv), Et_3_N (1.0 g, 10 mmol, 2.0 equiv) were dissolved in DCM (20 mL), cooled to 0 ℃, and used a syringe to measureTiCl_4_ (0.6 mL, 985 mg, 5 mmol, 1.0 equiv), placed the needle below the liquid level and slowly added. After addition, the reaction was placed at 40 ℃ for 4 hours. After the reaction is completed, H_2_O was added to quench the reaction. The organic layer was removed, and the aqueous layer was extracted twice with AcOEt. concentrated, and purified by flash chromatography (petroleum ether : AcOEt = 8 : 1) afforded the product **1** (The product nuclear magnetic resonance data is consistent with literature reports).^[4]^

***tert-*Butyl (2-formylallyl) carbonate**

**S10.** Colorless oil. Yield: 98%. **^1^H NMR (300 MHz, CDCl_3_)** *δ* 9.53 (s, 1H), 6.44 (s, 1H), 6.16 (s, 1H), 4.73 (s, 2H), 1.43 (s, 9H) ppm. **^13^C NMR** **(75 MHz, CDCl_3_)** *δ* 129.4, 153.0, 144.4, 134.7, 82.6, 62.3, 27.7 ppm. **HRMS** (ESI) (m/z) [M+Na]^+^ Calcd for C_9_H_14_NaO_4_^+^ 209.0784; found 209.0792.

***tert*-Butyl (2-(hydroxy(phenyl)methyl)allyl) carbonate**

**2a**, Colorless oil. Yield: 569 mg, 43%. **^1^H NMR (300 MHz, CDCl_3_)** *δ* 7.40 - 7.31 (m, 5H), 6.13 (s, 1H), 5.29 (t, *J* = 11.2 Hz, 2H), 4.06 (dd, *J* = 30.6, 14.1 Hz, 2H), 2.05 (bs, 1H), 1.47 (s, 9H) ppm. **^13^C NMR (75 MHz, CDCl_3_)** *δ* 152.8, 146.9, 137.7, 128.5, 128.3, 127.1, 112.7, 82.6, 78.5, 63.2, 27.8 ppm. **HRMS** (ESI) (m/z) [M+Na]^+^ Calcd for C_15_H_20_NaO_4_^+^ 287.1254; found 287.1258.

***tert*-Butyl (2-(hydroxy(o-tolyl)methyl)allyl) carbonate**

**2b**, Colorless oil. Yield: 640 mg, 46%. **^1^H NMR (300 MHz, CDCl_3_)** *δ* 7.41 - 7.36 (m, 1H), 7.24 - 7.14 (m, 3H), 6.36 (s, 1H), 5.32 (s, 1H), 5.11 (s, 1H), 4. 08 (q, *J* = 14.1 Hz, 2H), 2.52 (bs, 1H), 2.38 (s, 3H), 1.47 (s, 9H) ppm. **^13^C NMR** **(75 MHz, CDCl_3_)** *δ* 153.1, 146.2, 136.1, 135.8, 130.5, 128.2, 126.8, 126.1, 113.6, 82.5, 75.3, 63.4, 27.8, 19.8 ppm. **HRMS** (m/z) [M+Na]^+^ Calcd for C_16_H_22_NaO_4_^+^ 301.1410, found 301.1432.

***tert*-Butyl (2-(hydroxy(m-tolyl)methyl)allyl) carbonate**

**2c**, Colorless oil. Yield: 666 mg, 48%. **^1^H NMR** (300 MHz, CDCl_3_) *δ* 7.26 - 7.16 (m, 3H), 7.11 (d, *J* = 7.2 Hz, 1H), 6.09 (s, 1H), 5.29 (s, 1H), 5.16 (s, 1H), 4.02 (q, *J* = 14.1 Hz, 2H), 2.84 (bs, 1H), 2.34 (s, 3H), 1.47 (s, 9H) ppm. **^13^C NMR** (75 MHz, CDCl_3_) δ 152.9, 147.0, 138.1, 137.6, 129.1, 128.4, 127.8, 112.0, 82.5, 78.6, 62.9, 27.8, 21.4 ppm. **HRMS** (ESI) (m/z) [M+Na]^+^ Calcd for C_16_H_22_NaO_4_^+^ 301.1410, found 301.1432.

***tert*-Butyl (2-(hydroxy(p-tolyl)methyl)allyl) carbonate**

**2d**, Colorless oil. Yield: 739 mg, 53%. **^1^H NMR (300 MHz, CDCl_3_)** *δ* 7.23 - 7.30 (m, 2H, overlapped with the peak of chloroform), 7.17 (d, *J* = 7.8 Hz, 2H), 6.10 (s, 1H), 5.28 (d, *J* = 8.3 Hz, 2H), 4.03 (q, *J* = 13.9 Hz, 2H), 2.35 (s, 3H) 2.10 (bs, 1H), 1.48 (s, 9H) ppm. **^13^C NMR (75 MHz, CDCl_3_)** *δ* 152.9, 147.1, 138.1, 134.7, 129.2, 127.1, 112.4, 82.5, 78.4, 63.3, 27.8, 21.2 ppm. **HRMS** (ESI) (m/z) [M+Na]^+^ Calcd for C_16_H_22_NaO_4_^+^ 301.1410; found 301.1419.

***tert*-Butyl (2-((4-fluorophenyl)(hydroxy)methyl)allyl) carbonate**

**2e**, Colorless oil. Yield: 857 mg, 61%. **^1^H NMR (300 MHz, CDCl_3_)** *δ* 7.35 - 7.30 (m, 2H), 7.05 - 6.98 (m, 2H), 5.34 (s, 1H), 5.27 (d, *J* = 8.7 Hz, 2H), 4.45 (dd, *J* = 52.2, 13.2 Hz, 2H), 2.8 (bs, 1H), 1.45 (s, 9H) ppm. **^13^C NMR (75 MHz, CDCl_3_)** *δ* 162.3 (d, *J* = 245.8 Hz, F-C), 160.7, 153.3, 145.3, 136.9 (d, *J* = 3.1 Hz, F-CH), 128.3, 128.2, 115.3 (d, *J* = 21.4 Hz, F-CH), 114.6, 82.5, 74.3, 66.3, 27.7 ppm. **HRMS** (ESI) (m/z) [M+Na]^+^ Calcd for C_15_H_19_NaO_4_^+^ 305.1160; found 305.1189.

***tert*-Butyl (2-((4-chlorophenyl)(hydroxy)methyl)allyl) carbonate**

**2f**, Colorless oil. Yield: 867 mg, 58%. **^1^H NMR (300 MHz, CDCl_3_)** *δ* 7.31 - 7.25 (m, 4H, overlapped with the peak of chloroform), 5.31 (s, 1H), 5.27 (s, 2H), 5.22 (s, 2H), 4.44 (dd, *J* = 52.8, 13.5 Hz, 2H), 3.16 (bs, 1H), 1.43 (s, 9H) ppm. **^13^C NMR (75 MHz, CDCl_3_)** *δ* 153.3, 145.1, 139.8, 133.5, 128.6, 127.9, 114.9, 82.5, 74.3, 66.2, 27.7 ppm. **HRMS** (ESI) (m/z) [M+Na]^+^ Calcd for C_15_H_19_ClNaO_4_^+^ 321.0864; found 321.0894.

***tert*-Butyl (2-((3-fluorophenyl)(hydroxy)methyl)allyl) carbonate**

**2g**, Colorless oil. **Yield**: 744 mg, 66%. **^1^H NMR (300 MHz, CDCl_3_)** *δ* 7.35 – 7.25 (m, 1H), 7.17 – 7.06 (m, 2H), 6.99 (td, *J* = 8.3, 1.8 Hz, 1H), 6.10 (s, 1H), 5.27 (d, *J* = 17.0 Hz, 2H), 4.09 (d, *J* = 14.0 Hz, 1H), 3.97 (d, *J* = 14.0 Hz, 1H), 2.62 (s, 1H), 1.46 (s, 9H) ppm. **^13^C NMR (151 MHz, CDCl_3_)** *δ* 162.8 (d, *J* = 246.5 Hz, F-C), 152.7, 146.5, 140.4 (d, *J* = 7.1 Hz, F-C), 130.1 (d, *J* = 8.1 Hz, F-C), 122.8, 115.2 (d, *J* = 21.1 Hz, F-C), 114.0 (d, *J* = 22.3 Hz, F-C), 113.2, 82.9, 77.8, 63.0, 27.7 ppm. **HRMS** (ESI) (m/z) [M+H]^+^ Calcd for C_15_H_20_FO_4_^+^ 283.1340; found 283.1322.

***tert*-Butyl (2-((3-chlorophenyl)(hydroxy)methyl)allyl) carbonate**

**2h**, Colorless oil. **Yield**: 763 mg, 64%. **^1^H NMR (600 MHz, CDCl_3_)** *δ* 7.37 (s, 1H), 7.29 – 7.19 (m, 3H), 6.08 (s, 1H), 5.27 (d, *J* = 32.6 Hz, 2H), 4.07 (d, *J* = 14.1 Hz, 1H), 3.95 (d, *J* = 14.1 Hz, 1H), 2.75 (s, 1H), 1.46 (s, 9H) ppm. **^13^C NMR (151 MHz, CDCl_3_)** *δ* 152.7, 146.4, 139.9, 134.5, 129.8, 128.5, 127.2, 125.4, 113.0, 82.9, 77.7, 62.9, 27.7 ppm. **HRMS** (ESI) (m/z) [M+Na]^+^ Calcd for C_15_H_19_ClNaO_4_^+^ 321.0864; found 321.0867.

***tert*-Butyl (2-(hydroxymethyl)-1-(3-methoxyphenyl)allyl) carbonate**

**2i**, Colorless oil. **Yield**: 694 mg, 59%. **^1^H NMR (600 MHz, CDCl_3_)** *δ* 7.23 (t, *J* = 7.9 Hz, 1H), 6.94 (d, *J* = 7.6 Hz, 1H), 6.90 (s, 1H), 6.82 (dd, *J* = 8.1, 2.2 Hz, 1H), 6.08 (s, 1H), 5.26 (d, *J* = 21.7 Hz, 2H), 4.06 (d, *J* = 14.1 Hz, 1H), 3.97 (d, *J* = 14.1 Hz, 1H), 3.76 (s, 3H), 2.68 (s, 1H), 1.45 (s, 8H) ppm. **^13^C NMR (151 MHz, CDCl_3_)** *δ* 159.7, 152.8, 146.9, 139.3, 129.6, 119.5, 113.9, 112.6, 112.4, 82.6, 78.4, 62.9, 55.2, 27.8 ppm. **HRMS** (ESI) (m/z) [M+Na]^+^ Calcd for C_16_H_22_NaO_5_^+^ 317.1359; found 317.1349.

***tert*-Butyl (2-((3, 5-dimethylphenyl)(hydroxy)methyl)allyl) carbonate**

**2j**, Colorless oil. **Yield**: 685 mg, 47%. **^1^H NMR (300 MHz, CDCl_3_)** *δ* 7.00 (s, 2H), 6.95 (s, 1H), 6.06 (s, 1H), 5.28 (d, *J* = 3.8 Hz, 1H), 4.04 (q, *J* = 14.1 Hz, 2H), 2.62 (bs, 1H), 2.31 (s, 6H), 1.49 (s, 9H) ppm. **^13^C NMR** **(75 MHz, CDCl_3_)** *δ* 152.9, 147.1, 138.0, 137.5, 130.0, 124.9, 111.9, 82.5, 78.7, 63.1, 27.8, 21.3 ppm. **HRMS** (m/z) [M+Na]^+^ Calcd for C_17_H_24_NaO_4_^+^ 315.1567, found 315.1587.

***tert*-Butyl (3-hydroxy-2-methylenebutyl) carbonate**

**2k**, Colorless oil. Yield: 766 mg, 76%. **^1^H NMR (300 MHz, CDCl_3_)** *δ* 5.18-5.09 (m, 3H), 4.10 (s, 2H), 2.76 (s, 1H), 1.42 (s, 9H), 1.35 (d, *J* = 6.6 Hz, 3H) ppm. **^13^C NMR (75 MHz, CDCl_3_)** *δ* 153.0, 148.4, 111.4, 82.1, 73.4, 63.1, 27.7, 19.7 ppm. **HRMS** (ESI) (m/z) [M+H]^+^ Calcd for C_10_H_19_O_4_^+^ 203.1278; found 203.1296.

***tert*-Butyl (2-(hydroxymethyl)-1-(naphthalen-2-yl)allyl) carbonate**

**2l**, Colorless oil. Yield: 716 mg, 57%. **^1^H NMR (600 MHz, CDCl_3_)** *δ* 7.83 (s, 1H), 7.79 – 7.70 (m, 3H), 7.49 – 7.34 (m, 3H), 6.29 (s, 1H), 6.29 (s, 1H), 5.29 (s, 2H), 4.05 (d, *J* = 14.2 Hz, 1H), 3.96 (d, *J* = 14.2 Hz, 1H), 3.06 (s, 1H), 1.41 (s, 9H) ppm. **^13^C NMR (151 MHz, CDCl_3_)** *δ* 153.0, 147.0, 135.3, 133.3, 133.2, 128.5, 128.2, 127.8, 126.6, 126.4, 126.4, 124.9, 112.5, 82.7, 78.8, 63.0, 27.9 ppm. **HRMS** (ESI) (m/z) [M+Na]^+^ Calcd for C_19_H_22_NaO_4_^+^ 337.1410; found 337.1399.

[1] S. Jia, M. Ma, E.-Q. Li, Z. Duan, F. Mathey, *Org. Lett.* **2021**, *23*, 3337-3342.

[2] G. Zhao, Y. Wu, H.-H. Wu, J. Yang, J.-L. Zhang, *J. Am. Chem. Soc.* **2021**, *143*, 17983–17988.

[3] Y. Meng, Q. Wang, X. Yao, D. Wei, Y.-G. Liu, E.-Q. Li, Z. Duan, *Org. Lett.* **2022**, *24*, 9205–9209.

[4] E. Li, P. Jia, L. Liang, Y. Huang, *ACS Catal.* **2014**, *4*, 600-603.

**3. Reaction Optimizations**

**Table S1.** Screening of metal*^a^*

| Entry | Metal | Yield(%) | ee (%)*^b^* |
| --- | --- | --- | --- |
| 1 | Pd_2_(dba)_3_ | 78 | 94 |
| 2 | Pd(dba)_2_ | 53 | 93 |
| 3 | [Rh(COD)Cl]_2_ | NR |  |
| 4 | Pd(OAc)_2_ | 49 | 64 |
| 5 | [Ir(COD)Cl]_2_ | NR |  |
| 6 | [RhCl_2_Cp^*^]_2_ | NR |  |
| 7 | Pd(COD)Cl_2_ | 53 | 93 |

*^a^* Reactions were performed with **1** (0.05 mmol), **2** (0.075 mmol) in toluene (1.0 mL), N_2_.

*^b^* Determined by chiral HPLC analysis.

**Table S2.** Screening of solvents*^a^*

| Entry | Solvent | Yield(%) | ee (%)*^b^* |
| --- | --- | --- | --- |
| 1 | toluene | 78 | 94 |
| 2 | CH_2_Cl_2_ | 45 | 61 |
| 3 | CHCl_3_ | 53 | 83 |
| 4 | THF | 49 | 61 |
| 5 | Et_2_O | 41 | 95 |
| 6 | dioxane | Trace |  |
| 7 | EA | 37 | 88 |
| 8 | MeCN | 29 | 50 |
| 9 | 1,2-dimethoxyethane | 37 | 85 |
| 10 | DMSO | NR |  |
| 11 | DMF | NR |  |

*^a^* Reactions were performed with **1** (0.05 mmol), **2** (0.075 mmol) in toluene (1.0 mL), N_2_.

*^b^* Determined by chiral HPLC analysis.

**Table S3.** Screening of bases*^a^*

| Entry | Base | Yield (%) | ee (%)*^b^* |
| --- | --- | --- | --- |
| 1 | Cs_2_CO_3_ | 78 | 94 |
| 2 | LiOH | 33 | 95 |
| 3 | Na_2_CO_3_ | 41 | 95 |
| 4 | DBU | 65 | 24 |
| 5 | DABCO | 49 | 90 |
| 6 | NaHCO_3_ | Trace |  |
| 7 | KH_2_PO_4_ | Trace |  |
| 8*^c^* | Cs_2_CO_3_ | 33 | 95 |
| 9*^d^* | Cs_2_CO_3_ | 45 | 96 |
| 10*^e^* | Cs_2_CO_3_ | 49 | 95 |
| 11*^f^* | Cs_2_CO_3_ | 61 | 90 |

*^a^* Reactions were performed with **1** (0.05 mmol), **2** (0.075 mmol) in toluene (1.0 mL), N_2_.

*^b^* Determined by chiral HPLC analysis.

*^c^* Base (1.0 equiv). *^d^* Base (20 mol%). *^e^* Base (60 mol%). *^f^* Base (1.5 equiv)

**Table S4.** Screening of catalyst ratios*^a^*

| Entry | x | y | Yield(%) | ee (%)*^b^* |
| --- | --- | --- | --- | --- |
| 1 | 5 | 10 | 78 | 94 |
| 2 | 5 | 5 | 41 | 95 |
| 3 | 5 | 7.5 | 33 | 90 |
| 4 | 5 | 8 | 57 | 95 |
| 5 | 5 | 12 | 49 | 95 |
| 6 | 3 | 5 | 41 | 95 |
| 7 | 1 | 2 | 37 | 84 |

*^a^* Reactions were performed with **1** (0.05 mmol), **2** (0.075 mmol) in toluene (1.0 mL), N_2_.

*^b^* Determined by chiral HPLC analysis.

**4. General Procedure for Reactions**

**Condition A**

Ligand **MQ Phos-1** (4.3 mg, 0.005 mmol, 10 mol %) and Pd_2_(dba)_3_ (2.3 mg, 0.0025 mmol, 5 mol %) were dissolved in toluene (1.0 mL) in a 10 mL Schlenk tube under N_2_. After stirring at room temperature for 40 minutes, *α, β*-unsaturated imines (0.05 mmol)‚ 1, 2-disubstituted-π-allyl precursors (0.075 mmol), Cs_2_CO_3_ (32.5 mg, 0.1 mmol, 2.0 equiv) were added. The reaction mixture was stirred at 40 °C until the substrate was consumed (monitored by TLC), and then was purified by flash column chromatrography (petroleum ether : AcOEt = 20 : 1), afford the corresponding product **3-56**.

**Condition B**

Ligand **Meng Phos-1** (2.2 mg, 0.005 mmol, 10 mol %) and Pd_2_(dba)_3_ (2.3 mg, 0.0025 mmol, 5 mol %) were dissolved in toluene (1.0 mL) in a 10 mL Schlenk tube under N_2_. After stirring at room temperature for 40 minutes, *α, β*-unsaturated imines (0.05 mmol)‚ 1, 2-disubstituted-π-allyl precursors (0.075 mmol), Cs_2_CO_3_ (32.5 mg, 0.1 mmol, 2.0 equiv) were added. The reaction mixture was stirred at room temperature until the substrate was consumed (monitored by TLC), and then was purified by flash column chromatrography (petroleum ether : AcOEt = 20 : 1), afford the corresponding product.

**3**

**(*S, Z*)-3-((*Z*)-benzylidene)-6, 8-diphenyl-5-(phenylsulfonyl)-3, 4, 5, 8-tetrahydro-2*H*-1, 5-oxazocine**

The reaction of **1a** (17.4 mg, 0.05 mmol) and **2a** (19.8 mg, 0.075 mmol), Cs_2_CO_3_ (32.5 mg, 0.1 mmol), after a flash column chromatography (petroleum ether : AcOEt = 20 : 1), afforded the product **3** (18.2 mg, 74% yield). (**Condition B** : 83% yield, 92% ee)

**3**. White solid. **MP:** 127 - 128 ºC. [α]_D_ = - 23 (*c* = 0.1, CH_2_Cl_2_, 26.8 °C). **^1^H NMR (300 MHz, CDCl_3_)** *δ* 7.70 (d, *J* = 7.7 Hz, 2H), 7.62 (d, *J* = 7.3 Hz, 2H), 7.53 - 7.31 (m, 11H), 7.26 - 7.14 (m, 5H), 6.90 (s, 1H), 6.38 (d, *J* = 8.9 Hz, 1H), 5.38 (d, *J* = 8.9 Hz, 1H), 5.07 (d, *J* = 13.4 Hz, 1H), 4.43 (q, *J* = 12.2 Hz, 2H), 4.13 (d, *J* = 13.1 Hz, 1H) ppm. **^13^C NMR (75 MHz, CDCl_3_)** *δ* 141.3, 140.6, 138.8, 136.3, 135.6, 133.3, 133.1, 132.5, 129.6, 128.9, 128.7, 128.5, 128.4, 128.4, 128.1, 127.7, 127.3, 126.5, 126.4, 75.7, 68.7, 61.4 ppm. **HRMS** (ESI) (m/z) [M+H]^+^ Calcd for C_31_H_28_NO_3_S^+^ 494.1784; found 494.1791. **HPLC:** The product was analyzed by HPLC to determine the enantiomeric excess: 94% ee (Chiralpak IA-H, *n*-hexane/*i*-propanol = 95/5, 1 mL/min, 254 nm) t_R_ = 16.694 min, 19.330 min.

**4**

**(*S, Z*)-3-((*Z*)-2-methylbenzylidene)-6, 8-diphenyl-5-(phenylsulfonyl)-3, 4, 5, 8-tetrahydro-2*H*-1, 5-oxazocine**

The reaction of **1a** (17.4 mg, 0.05 mmol) and **2b** (20.9 mg, 0.075 mmol), Cs_2_CO_3_ (32.5 mg, 0.1 mmol), after a flash column chromatography (petroleum ether : AcOEt = 20 : 1), afforded the product **4** (15.0 mg, 59% yield).

**4**. White solid. **MP:** 137 - 138 ºC. [α]_D_ = - 23 (*c* = 0.1, CH_2_Cl_2_, 30.1 °C). **^1^H NMR (300 MHz, CDCl_3_)** *δ* 7.71 - 7.62 (m, 3H), 7.52 - 7.32 (m, 8H), 7.25 - 7.13 (m, 8H), 6.93 (s, 1H), 6.37 (d, *J* = 8.6 Hz, 1H), 5.33 (d, *J* = 8.2 Hz, 1H), 5.12 - 4.96 (m, 1H), 4.32 (dd, *J* = 28.3, 12.1 Hz, 2H), 4.25 - 4.14 (m, 1H), 2.27 (s, 3H) ppm. **^13^C NMR (75 MHz, CDCl_3_)** *δ* 141.3, 140.6, 137.3, 136.4, 136.2, 134.6, 133.5, 133.5, 132.5, 130.2, 129.7, 128.8, 128.6, 128.5, 128.3, 128.2, 127.6, 127.3, 126.5, 126.4, 125.9, 75.9, 68.8, 61.0, 20.1 ppm. **HRMS** (ESI) (m/z) [M+H]^+^ Calcd for C_32_H_30_NO_3_S^+^ 508.1941; found 508.1949. **HPLC**: The product was analyzed by HPLC to determine the enantiomeric excess: 73% ee (Chiralpak IA-H, *n*-hexane/*i*-propanol = 95/5, 1 mL/min, 254 nm) t_R_ = 11.891 min, 13.801 min.

**5**

**(*S, Z*)-3-((*Z*)-3-methylbenzylidene)-6, 8-diphenyl-5-(phenylsulfonyl)-3, 4, 5, 8-tetrahydro-2*H*-1, 5-oxazocine**

The reaction of **1a** (17.4 mg, 0.05 mmol) and **2c** (20.9 mg, 0.075 mmol), Cs_2_CO_3_ (32.5 mg, 0.1 mmol) at room temperature, after a flash column chromatography (petroleum ether : AcOEt = 20 : 1), afforded the product **5** (16.7 mg, 66% yield).

**5**. White solid. **MP:** 107 - 108 ºC. [α]_D_ = - 10 (*c* = 0.1, CH_2_Cl_2_, 30.7 °C). **^1^H NMR (300 MHz, CDCl_3_)** *δ* 7.69 (d, *J* = 7.6 Hz, 2H), 7.52 - 7.29 (m, 11H), 7.25 - 7.11 (m, 6H), 6.85 (s, 1H), 6.36 (d, *J* = 8.9 Hz, 1H), 5.34 (d, *J* = 8.9 Hz, 1H), 5.04 (d, *J* = 13.1 Hz, 1H), 4.41 (q, *J* = 12.1 Hz, 2H), 4.11 (d, *J* = 13.1 Hz, 1H), 2.37 (s, 3H) ppm. **^13^C NMR (75 MHz, CDCl_3_)** *δ* 141.4, 140.6, 138.9, 137.9, 136.3, 135.6, 133.3, 132.9, 132.5, 130.3, 128.9, 128.8, 128.6, 128.5, 128.3, 127.6, 127.3, 126.6, 126.5, 126.3, 75.6, 68.7, 61.4, 21.5 ppm. **HRMS** (ESI) (m/z) [M+H]^+^ Calcd for C_32_H_30_NO_3_S^+^ 508.1941; found 508.1946. **HPLC**: The product was analyzed by HPLC to determine the enantiomeric excess: 94% ee (Chiralpak IA-H, *n*-hexane/*i*-propanol = 95/5, 1 mL/min, 254 nm) t_R_ = 13.869 min, 19.473 min.

**6**

**(*S, Z*)-3-((*Z*)-4-methylbenzylidene)-6, 8-diphenyl-5-(phenylsulfonyl)-3, 4, 5, 8-tetrahydro-2*H*-1, 5-oxazocine**

The reaction of **1a** (17.4 mg, 0.05 mmol) and **2d** (20.9 mg, 0.075 mmol), Cs_2_CO_3_ (32.5 mg, 0.1 mmol) at room temperature, after a flash column chromatography (petroleum ether : AcOEt = 20 : 1), afforded the product **6** (17.2 mg, 68% yield).

**6**. White solid. **MP:** 112 - 113 ºC. [α]_D_ = - 4 (*c* = 0.1, CH_2_Cl_2_, 24.8 °C). **^1^H NMR (300 MHz, CDCl_3_)** *δ* 7.70 (d, *J* = 7.6 Hz, 2H), 7.55 - 7.41 (m, 7H), 7.39 - 7.31 (m, 3H), 7.26 - 7.15 (m, 7H), 6.87 (s, 1H), 6.37 (d, *J* = 9.0 Hz, 1H), 5.34 (d, *J* = 9.0 Hz, 1H), 5.06 (d, *J* = 13.4 Hz, 1H), 4.49 - 4.34 (m, 2H), 4.12 (d, *J* = 13.3 Hz, 1H), 2.38 (s, 3H) ppm. **^13^C NMR (75 MHz, CDCl_3_)** *δ* 141.3, 140.6, 138.8, 138.1, 136.4, 133.2, 132.9, 132.5, 132.1, 129.6, 129.2, 128.8, 128.6, 128.5, 128.4, 127.6, 127.3, 126.5, 126.4, 75.6, 68.7, 61.4, 21.3 ppm. **HRMS** (ESI) (m/z) [M+H]^+^ Calcd for C_32_H_30_NO_3_S^+^ 508.1941; found 508.1951. **HPLC**: The product was analyzed by HPLC to determine the enantiomeric excess: 87% ee (Chiralpak IA-H, *n*-hexane/*i*-propanol = 95/5, 1 mL/min, 254 nm) t_R_ = 15.875 min, 17.795 min.

**7**

**(*S, Z*)-3-((*Z*)-4-fluorobenzylidene)-6, 8-diphenyl-5-(phenylsulfonyl)-3, 4, 5, 8-tetrahydro-2*H*-1, 5-oxazocine**

The reaction of **1a** (17.4 mg, 0.05 mmol) and **2e** (21.2 mg, 0.075 mmol), Cs_2_CO_3_ (32.5 mg, 0.1 mmol), after a flash column chromatography (petroleum ether : AcOEt = 20 : 1), afforded the product **7** (18.1 mg, 71% yield).

**7**. Yellow oil. [α]_D_ = - 22 (*c* = 0.1, CH_2_Cl_2_, 30.0 °C). **^19^F NMR (282 MHz, CDCl_3_)** *δ* - 113.70 ppm. **^1^H NMR (300 MHz, CDCl_3_)** *δ* 7.68 (d, *J* = 7.5 Hz, 2H), 7.59 (dd, *J* = 8.3, 5.6 Hz, 2H), 7.51 - 7.40 (m, 5H), 7.37 - 7.32 (m, 3H), 7.25 - 7.13 (m, 5H), 7.06 (t, *J* = 8.7 Hz, 2H), 6.85 (s, 1H), 6.36 (d, *J* = 9.0 Hz, 1H), 5.37 (d, *J* = 9.0 Hz, 1H), 5.04 (d, *J* = 13.5 Hz, 1H), 4.46 - 4.31 (m, 2H), 4.08 (d, *J* = 13.5 Hz, 1H) ppm. **^13^C NMR (75 MHz, CDCl_3_)** *δ* 162.6 (d, *J*_C-F_ = 248.5 Hz), 144.9, 141.2, 140.6, 137.7, 136.1, 133.2, 132.9, 132.9, 132.8, 132.7, 132.6, 131.8 (d, *J*_C-F_ = 3.3 Hz), 131.5, 131.4, 130.6, 129.1, 129.0, 128.9, 128.8, 128.7, 128.6, 128.5, 128.5, 128.5, 128.4, 127.7, 127.3, 126.5, 126.4, 122.1, 115.4 (d, *J*_C-F_ = 21.4 Hz), 75.7, 68.4, 61.2 ppm. **HRMS** (ESI) (m/z) [M+Na]^+^ Calcd for C_31_H_26_FNO_3_SNa^+^ 534.1510; found 534.1519. **HPLC**: The product was analyzed by HPLC to determine the enantiomeric excess: 91% ee (Chiralpak IA-H, *n*-hexane/*i*-propanol = 95/5, 1 mL/min, 254 nm) t_R_ = 18.145 min, 24.580 min.

**8**

**(*S, Z*)-3-((*Z*)-4-chlorobenzylidene)-6, 8-diphenyl-5-(phenylsulfonyl)-3, 4, 5, 8-tetrahydro-2*H*-1, 5-oxazocine**

The reaction of **1a** (17.4 mg, 0.05 mmol) and **2f** (22.4 mg, 0.075 mmol), Cs_2_CO_3_ (32.5 mg, 0.1 mmol), after a flash column chromatography (petroleum ether : AcOEt = 20 : 1), afforded the product **8** (18.4 mg, 70% yield).

**8**. Yellow oil. [α]_D_ = - 11 (*c* = 0.1, CH_2_Cl_2_, 24.5 °C). **^1^H NMR (300 MHz, CDCl_3_)** *δ* 7.68 (d, *J* = 7.6 Hz, 2H), 7.58 - 7.42 (m, 7H), 7.35 (t, *J* = 7.0 Hz, 5H), 7.25 - 7.13 (m, 5H), 6.84 (s, 1H), 6.36 (d, *J* = 9.0 Hz, 1H), 5.37 (d, *J* = 8.9 Hz, 1H), 5.04 (d, *J* = 13.4 Hz, 1H), 4.47 - 4.31 (m, 2H), 4.08 (d, *J* = 13.4 Hz, 1H) ppm. **^13^C NMR (75 MHz, CDCl_3_)** *δ* 141.1, 140.6, 137.5, 136.1, 134.1, 133.8, 133.2, 132.6, 130.9, 128.9, 128.7, 128.6, 128.6, 128.4, 127.7, 127.3, 126.5, 126.4, 75.7, 68.3, 61.1 ppm. **HRMS** (ESI) (m/z) [M+H]^+^ Calcd for C_31_H_27_ClNO_3_S^+^ 528.1395; found 528.1389. **HPLC**: The product was analyzed by HPLC to determine the enantiomeric excess: 90% ee (Chiralpak IA-H, *n*-hexane/*i*-propanol = 95/5, 1 mL/min, 254 nm) t_R_ = 19.657 min, 29.480 min.

**9**

**(*S, Z*)-3-((*Z*)-3-fluorobenzylidene)-6, 8-diphenyl-5-(phenylsulfonyl)-3, 4, 5, 8-tetrahydro-2*H*-1, 5-oxazocine**

The reaction of **1a** (17.4 mg, 0.05 mmol) and **2g** (21.2 mg, 0.075 mmol), Cs_2_CO_3_ (32.5 mg, 0.1 mmol) at room temperature, after a flash column chromatography (petroleum ether : AcOEt = 20 : 1), afforded the product **9** (13.8 mg, 54% yield).

**9**. Yellow oil. [α]_D_ = - 20 (*c* = 0.1, CH_2_Cl_2_, 25.0 °C). **^19^F NMR (282 MHz, CDCl_3_)** *δ* - 113.44 ppm. **^1^H NMR (600 MHz, CDCl_3_)** δ 7.65 (d, *J* = 7.5 Hz, 2H), 7.49 - 7.45 (m, 3H), 7.41 (t, *J* = 7.6 Hz, 2H), 7.38 - 7.29 (m, 6H), 7.22 (t, *J* = 7.1 Hz, 1H), 7.19 - 7.12 (m, 4H), 7.01 - 6.97 (m, 1H), 6.81 (s, 1H), 6.33 (d, *J* = 8.9 Hz, 1H), 5.36 (d, *J* = 8.8 Hz, 1H), 5.02 (d, *J* = 12.9 Hz, 1H), 4.38 (dd, *J* = 38.6, 12.2 Hz, 2H), 4.07 (d, *J* = 13.4 Hz, 1H) ppm. **^13^C NMR (151 MHz, CDCl_3_)** δ 162.7 (d, *J*_C-F_ = 245.6 Hz), 141.3, 140.6, 137.8 (d, *J*_C-F_ = 7.9 Hz), 137.4, 136.1, 134.6, 133.5, 132.6, 129.9 (d, *J*_C-F_ = 8.3 Hz), 128.9, 128.7, 128.6, 128.4, 127.7, 127.3, 126.6, 126.3, 125.4 (d, *J*_C-F_ = 2.7 Hz), 116.3 (d, *J*_C-F_ = 22.3 Hz), 115.0 (d, *J*_C-F_ = 21.2 Hz), 75.8, 68.4, 61.0 ppm. **HRMS** (ESI) (m/z) [M+H]^+^ Calcd for C_31_H_27_FNO_3_S^+^ 512.1690; found 512.1691. **HPLC**: The product was analyzed by HPLC to determine the enantiomeric excess: 92% ee (Chiralpak IA-H, *n*-hexane/*i*-propanol = 95/5, 1 mL/min, 254 nm) t_R_ = 17.621 min, 22.744 min.

**10**

**(*S, Z*)-3-((*Z*)-3-chlorobenzylidene)-6, 8-diphenyl-5-(phenylsulfonyl)-3, 4, 5, 8-tetrahydro-2*H*-1, 5-oxazocine**

The reaction of **1a** (17.4 mg, 0.05 mmol) and **2h** (22.4 mg, 0.075 mmol), Cs_2_CO_3_ (32.5 mg, 0.1 mmol) at room temperature, after a flash column chromatography (petroleum ether : AcOEt = 20 : 1), afforded the product **10** (17.1 mg, 65% yield).

**10**. Yellow oil. [α]_D_ = - 34 (*c* = 0.1, CH_2_Cl_2_, 29.6 °C). **^1^H NMR (600 MHz, CDCl_3_)** *δ* 7.65 (d, *J* = 7.4 Hz, 2H), 7.59 (s, 1H), 7.49 - 7.44 (m, 4H), 7.41 (t, *J* = 7.6 Hz, 2H), 7.32 (t, *J* = 7.7 Hz, 3H), 7.29 - 7.25 (m, 2H), 7.23 - 7.19 (m, 1H), 7.18 - 7.16 (m, 2H), 7.15 - 7.12 (m, 2H), 6.77 (s, 1H), 6.33 (d, *J* = 8.9 Hz, 1H), 5.36 (d, *J* = 8.7 Hz, 1H), 5.01 (d, *J* = 12.6 Hz, 1H), 4.36 (dd, *J* = 35.5, 12.2 Hz, 2H), 4.06 (d, *J* = 12.2 Hz, 1H) ppm. **^13^C NMR (151 MHz, CDCl_3_)** *δ* 141.3, 140.6, 137.4, 137.2, 136.1, 134.8, 134.9, 134.3, 133.5, 132.6, 129.7, 129.6, 128.9, 128.7, 128.5, 128.4, 128.1, 127.7, 127.3, 126.6, 126.2, 75.7, 68.4, 61.0 ppm. **HRMS** (ESI) (m/z) [M+Na]^+^ Calcd for C_31_H_26_ClNO_3_SNa^+^ 550.1214; found 550.1201. **HPLC**: The product was analyzed by HPLC to determine the enantiomeric excess: 91% ee (Chiralpak IA-H, *n*-hexane/*i*-propanol = 95/5, 1 mL/min, 254 nm) t_R_ = 17.294 min, 20.455 min.

**11**

**(*S, Z*)-3-((*Z*)-3-methoxybenzylidene)-6, 8-diphenyl-5-(phenylsulfonyl)-3, 4, 5, 8-tetrahydro-2*H*-1, 5-oxazocine**

The reaction of **1a** (17.4 mg, 0.05 mmol) and **2i** (22.1 mg, 0.075 mmol), Cs_2_CO_3_ (32.5 mg, 0.1 mmol) at room temperature, after a flash column chromatography (petroleum ether : AcOEt = 20 : 1), afforded the product **11** (18.3 mg, 70% yield).

**11**. Yellow oil. [α]_D_ = - 6 (*c* = 0.1, CH_2_Cl_2_, 30.5 °C). **^1^H NMR (600 MHz, CDCl_3_)** *δ* 7.66 (d, *J* = 7.3 Hz, 2H), 7.48 - 7.44 (m, 3H), 7.41 - 7.37 (m, 2H), 7.34 - 7.31 (m, 3H), 7.27 - 7.25 (m, 1H), 7.24 - 7.17 (m, 4H), 7.16 - 7.09 (m, 3H), 6.87 - 6.83 (m, 2H), 6.32 (d, *J* = 9.0 Hz, 1H), 5.36 (d, *J* = 9.0 Hz, 1H), 5.03 (d, *J* = 12.4 Hz, 1H), 4.40 (dd, *J* = 31.7, 12.1 Hz, 2H), 4.07 (d, *J* = 12.5 Hz, 1H), 3.77 (s, 3H) ppm. **^13^C NMR (151 MHz, CDCl_3_)** *δ* 159.6, 141.4, 140.7, 137.0, 133.4, 132.5, 129.3, 128.8, 128.7, 128.5, 128.4, 127.6, 127.3, 126.6, 126.3, 122.1, 114.6, 114.4, 75.6, 68.7, 55.2 ppm. **HRMS** (ESI) (m/z) [M+H]^+^ Calcd for C_32_H_30_NO_4_S^+^ 524.1890; found 524.1889. **HPLC**: The product was analyzed by HPLC to determine the enantiomeric excess: 94% ee (Chiralpak IA-H, *n*-hexane/*i*-propanol = 95/5, 1 mL/min, 254 nm) t_R_ = 19.761 min, 30.527 min.

**12**

**(*S, Z*)-3-((*Z*)-3, 5-dimethylbenzylidene)-6, 8-diphenyl-5-(phenylsulfonyl)-3, 4, 5, 8-tetrahydro-2*H*-1, 5-oxazocine**

The reaction of **1a** (17.4 mg, 0.05 mmol) and **2j** (21.9 mg, 0.075 mmol), Cs_2_CO_3_ (32.5 mg, 0.1 mmol) at room temperature, after a flash column chromatography (petroleum ether : AcOEt = 20 : 1), afforded the product **12** (17.5 mg, 67% yield).

**12**. White solid. **MP:** 129 - 130 ºC. [α]_D_ = - 25 (*c* = 0.1, CH_2_Cl_2_, 30.4 °C). **^1^H NMR (300 MHz, CDCl_3_)** *δ* 7.70 (d, *J* = 7.5 Hz, 2H), 7.53 - 7.40 (m, 5H), 7.35 (t, *J* = 7.7 Hz, 3H), 7.27 - 7.15 (m, 7H), 6.98 (s, 1H), 6.83 (s, 1H), 6.36 (d, *J* = 8.9 Hz, 1H), 5.34 (d, *J* = 8.9 Hz, 1H), 5.05 (d, *J* = 13.2 Hz, 1H), 4.43 (q, *J* = 12.1 Hz, 2H), 4.12 (d, *J* = 13.5 Hz, 1H), 2.35 (s, 6H) ppm. **^13^C NMR (75 MHz, CDCl_3_)** *δ* 141.5, 140.6, 139.0, 137.8, 136.4, 135.6, 133.4, 132.7, 132.5, 129.8, 128.8, 128.6, 128.5, 128.3, 127.5, 127.4, 127.3, 126.5, 126.2, 75.4, 68.8, 61.4, 21.4 ppm. **HRMS** (ESI) (m/z) [M+Na]^+^ Calcd for C_33_H_31_NO_3_SNa^+^ 544.1917; found 544.1924. **HPLC**: The product was analyzed by HPLC to determine the enantiomeric excess: 90% ee (Chiralpak IA-H, *n*-hexane/*i*-propanol = 95/5, 1 mL/min, 254 nm) t_R_ = 11.865 min, 15.599 min.

**13**

**(*S,* 3*Z*, 6*Z*)-3-ethylidene-6, 8-diphenyl-5-(phenylsulfonyl)-3, 4, 5, 8-tetrahydro-2*H*-1, 5-oxazocine**

The reaction of **1a** (17.4 mg, 0.05 mmol) and **2k** (15.2 mg, 0.075 mmol), Cs_2_CO_3_ (32.5 mg, 0.1 mmol) at room temperature, after a flash column chromatography (petroleum ether : AcOEt = 20 : 1), afforded the product **13** (14.0 mg, 65% yield).

**13**. White solid. **MP:** 115 - 116 ºC. [α]_D_ = - 15 (*c* = 0.1, CH_2_Cl_2_, 27.3 °C). **^1^H NMR** **(600 MHz, CDCl_3_)** *δ* 7.62 (d, *J* = 7.6 Hz, 2H), 7.45 (t, *J* = 7.4 Hz, 1H), 7.41 - 7.35 (m, 4H), 7.33 - 7.28 (m, 3H), 7.23 - 7-19 (m, 1H), 7.19 - 7.16 (m, 2H), 7.16 - 7.13 (m, 2H), 6.23 (d, *J* = 8.9 Hz, 1H), 5.96 - 5.91 (m, 1H), 5.13 (d, *J* = 8.8 Hz, 1H), 4.86 – 4.74 (m, 1H), 4.32 (dd, *J* = 42.4, 12.3 Hz, 2H), 3.94 – 3.80 (m, 1H), 1.76 (d, *J* = 6.9 Hz, 3H) ppm. **^13^C NMR (151 MHz, CDCl_3_)** *δ* 141.4, 140.9, 136.5, 135.0, 132.9, 132.5, 132.4, 129.6, 128.8, 128.5, 128.5, 128.3, 127.6, 127.3, 126.5, 126.4, 75.2, 67.0, 59.7, 13.8 ppm. **HRMS** (ESI) (m/z) [M+H]^+^ Calcd for C_26_H_26_NO_3_S^+^ 432.1628; found 432.1621. **HPLC**: The product was analyzed by HPLC to determine the enantiomeric excess: 92% ee (Chiralpak IA-H, *n*-hexane/*i*-propanol = 95/5, 1 mL/min, 254 nm) t_R_ = 17.350 min, 20.451 min.

**14**

**(*S,* 3*Z*, 6*Z*)-3-(naphthalen-2-ylmethylene)-6, 8-diphenyl-5-(phenylsulfonyl)-3, 4, 5, 8-tetrahydro-2*H*-1, 5-oxazocine**

The reaction of **1a** (17.4 mg, 0.05 mmol) and **2l** (23.6 mg, 0.075 mmol), Cs_2_CO_3_ (32.5 mg, 0.1 mmol) at room temperature, after a flash column chromatography (petroleum ether : AcOEt = 20 : 1), afforded the product **14** (15.2 mg, 56% yield).

**14**. White solid. **MP:** 137 - 138 ºC. [α]_D_ = - 13 (*c* = 0.1, CH_2_Cl_2_, 20.8 °C). **^1^H NMR (600 MHz, CDCl_3_)** *δ* 8.14 (s, 1H), 7.84 - 7.81 (m, 1H), 7.80 - 7.77 (m, 2H), 7.68 (d, *J* = 7.4 Hz, 2H), 7.63 (d, *J* = 8.2 Hz, 1H), 7.51 (d, *J* = 7.4 Hz, 2H), 7.48 - 7.40 (m, 5H), 7.36 - 7.29 (m, 3H), 7.24 - 7.20 (m, 3H), 7.17 - 7.13 (m, 2H), 7.00 (s, 1H), 6.37 (d, *J* = 9.0 Hz, 1H), 5.38 (d, *J* = 8.6 Hz, 1H), 5.08 (d, *J* = 12.0 Hz, 1H), 4.45 (dd, *J* = 26.5, 12.2 Hz, 2H), 4.12 (d, *J* = 11.4 Hz, 1H) ppm. **^13^C NMR (151 MHz, CDCl_3_)** *δ* 141.5, 140.7, 138.9, 136.3, 133.6, 133.3, 133.2, 132.9, 132.6, 129.2, 128.9, 128.7, 128.6, 128.6, 128.4, 127.9, 127.7, 127.6, 127.4, 127.3, 126.6, 126.4, 126.4, 126.2, 75.6, 68.8, 61.3. **HRMS** (ESI) (m/z) [M+Na]^+^ Calcd for C_35_H_29_NO_3_SNa^+^ 566.1760; found 566.1768. **HPLC**: The product was analyzed by HPLC to determine the enantiomeric excess: 93% ee (Chiralpak IA-H, *n*-hexane/*i*-propanol = 95/5, 1 mL/min, 254 nm) t_R_ = 22.137 min, 30.650 min.

**15**

**(*S, Z*)-3-((*Z*)-benzylidene)-6, 8-diphenyl-5-tosyl-3, 4, 5, 8-tetrahydro-2*H*-1, 5-oxazocine**

The reaction of **1b** (18.1 mg, 0.05 mmol) and **2a** (19.8 mg, 0.075 mmol), Cs_2_CO_3_ (32.5 mg, 0.1 mmol), after a flash column chromatography (petroleum ether : AcOEt = 20 : 1), afforded the product **15** (15.2 mg, 60% yield). (**Condition B** : 79% yield, 77% ee)

**15**. White solid. **MP:** 117 - 118 ºC. [α]_D_ = - 9 (*c* = 0.1, CH_2_Cl_2_, 27.0 °C). **^1^H NMR (300 MHz, CDCl_3_)** *δ* 7.61 - 7.56 (m, 4H), 7.48 - 7.30 (m, 9H), 7.26 - 7.18 (m, 4H), 7.15 - 7.12 (m, 2H), 6.87 (s, 1H), 6.36 (d, *J* = 9.0 Hz, 1H), 5.30 (d, *J* = 9.0 Hz, 1H), 5.02 (d, *J* = 13.4 Hz, 1H), 4.40 (q, *J* = 12.1 Hz, 2H), 4.10 (d, *J* = 13.6 Hz, 1H), 2.37 (s, 3H) ppm. **^13^C NMR (75 MHz, CDCl_3_)** *δ* 143.4, 141.3, 138.7, 137.6, 136.5, 135.6, 133.2, 133.0, 129.6, 129.4, 128.6, 128.5, 128.4, 128.3, 128.1, 127.6, 127.4, 126.5, 126.4, 75.6, 68.7, 61.2, 42.0, 21.5 ppm. **HRMS** (ESI) (m/z) [M+H]^+^ Calcd for C_32_H_30_NO_3_S^+^ 508.1941; found 508.1935. **HPLC:** The product was analyzed by HPLC to determine the enantiomeric excess: 93% ee (Chiralpak IA-H, *n*-hexane/*i*-propanol = 95/5, 1 mL/min, 254 nm) t_R_ = 18.922 min, 25.505 min.

**16**

**(*S, Z*)-3-((*Z*)-benzylidene)-6, 8-diphenyl-5-((4-(trifluoromethyl)phenyl)sulfonyl)-3, 4, 5, 8-tetrahydro-2*H*-1, 5-oxazocine**

The reaction of **1c** (20.8 mg, 0.05 mmol) and **2a** (19.8 mg, 0.075 mmol), Cs_2_CO_3_ (32.5 mg, 0.1 mmol), after a flash column chromatography (petroleum ether : AcOEt = 20 : 1), afforded the product **16** (21.0 mg, 75% yield).

**16**. White solid. **MP:** 126 - 127 ºC. [α]_D_ = - 19 (*c* = 0.1, CH_2_Cl_2_, 23.3 °C). **^19^F NMR (282 MHz, CDCl_3_)** *δ* - 63.13 ppm. **^1^H NMR (300 MHz, CDCl_3_)** *δ* 7.80 - 7.70 (m, 2H), 7.67 - 7.53 (m, 5H), 7.51 - 7.32 (m, 9H), 7.18 - 7.12 (m, 3H), 6.96 (s, 1H), 6.37 (d, *J* = 8.8 Hz, 1H), 5.39 (d, *J* = 7.9 Hz, 1H), 5.12 (d, *J* = 12.3 Hz, 1H), 4.46 (s, 2H), 4.13 (d, *J* = 11.7 Hz, 1H) ppm. **^13^C NMR (75 MHz, CDCl_3_)** *δ* 143.9, 143.3, 141.0, 139.4, 135.9, 135.5, 134.8, 134.1 (d, *J*_C-F_ = 33.4 Hz), 133.7, 132.7, 130.5, 129.6, 129.0, 128.9, 128.6, 128.5, 128.4, 128.3, 127.8, 127.7, 126.6, 126.4, 125.9 (d, *J*_C-F_ = 3.7 Hz), 125.4, 123.2 (d, *J*_C-F_ = 271.2 Hz), 75.6, 68.6, 61.7 ppm. **HRMS** (ESI) (m/z) [M+H]^+^ Calcd for C_32_H_27_F_3_NO_3_S^+^ 562.1658; found 562.1650. **HPLC:** The product was analyzed by HPLC to determine the enantiomeric excess: 98% ee (Chiralpak IA-H, *n*-hexane/*i*-propanol = 95/5, 1 mL/min, 254 nm) t_R_ = 13.025 min, 16.928 min.

**17**

**(*S, Z*)-3-((*Z*)-benzylidene)-5-(methylsulfonyl)-6, 8-diphenyl-3, 4, 5, 8-tetrahydro-2*H*-1, 5-oxazocine**

The reaction of **1d** (14.3 mg, 0.05 mmol) and **2a** (19.8 mg, 0.075 mmol), Cs_2_CO_3_ (32.5 mg, 0.1 mmol), after a flash column chromatography (petroleum ether : AcOEt = 20 : 1), afforded the product **17** (17.2 mg, 80% yield).

**17**. Yellow oil. [α]_D_ = - 21 (*c* = 0.1, CH_2_Cl_2_, 23.5 °C). **^1^H NMR (300 MHz, CDCl_3_)** *δ* 7.65 - 7.50 (m, 5H), 7.47 - 7.31 (m, 10H), 6.96 (s, 1H), 6.36 (d, *J* = 8.6 Hz, 1H), 5.60 - 5.45 (m, 1H), 5.01 - 4.86 (m, 1H), 4.58 - 4.42 (m, 2H), 4.26 - 4.08 (m, 1H), 2.92 (s, 3H) ppm. **^13^C NMR (75 MHz, CDCl_3_)** *δ* 141.1, 139.5, 136.8, 135.6, 133.1, 129.7, 129.1, 128.8, 128.6, 128.5, 128.4, 128.2, 127.8, 126.5, 126.4, 75.6, 68.4, 60.9, 41.4 ppm. **HRMS** (ESI) (m/z) [M+H]^+^ Calcd for C_26_H_26_NO_3_S^+^ 432.1628; found 432.1621. **HPLC:** The product was analyzed by HPLC to determine the enantiomeric excess: 87% ee (Chiralpak IG-H, *n*-hexane/*i*-propanol = 95/5, 1 mL/min, 254 nm) t_R_ = 53.425 min, 59.764 min.

**18**

**(*S, Z*)-3-((*Z*)-benzylidene)-5-((4-methoxyphenyl)sulfonyl)-6, 8-diphenyl-3, 4, 5, 8-tetrahydro-2*H*-1, 5-oxazocine**

The reaction of **1e** (18.9 mg, 0.05 mmol) and **2a** (19.8 mg, 0.075 mmol), Cs_2_CO_3_ (32.5 mg, 0.1 mmol), after a flash column chromatography (petroleum ether : AcOEt = 20 : 1), afforded the product **18** (19.1 mg, 73% yield).

**18**. Yellow oil. [α]_D_ = - 20 (*c* = 0.1, CH_2_Cl_2_, 24.3 °C). **^1^H NMR (300 MHz, CDCl_3_)** *δ* 7.65 - 7.56 (m, 4H), 7.50 - 7.43 (m, 3H), 7.43 - 7.30 (m, 6H), 7.26 - 7.20 (m, 4H), 6.87 (s, 1H), 6.84 - 6.76 (m, 2H), 6.36 (d, *J* = 9.0 Hz, 1H), 5.32 (d, *J* = 8.9 Hz, 1H), 5.01 (d, *J* = 13.5 Hz, 1H), 4.40 (q, *J* = 12.1 Hz, 2H), 4.10 (d, *J* = 13.5 Hz, 1H), 3.80 (s, 3H) ppm. **^13^C NMR (75 MHz, CDCl_3_)** *δ* 162.8, 141.3, 140.7, 138.7, 136.5, 135.7, 133.2, 133.0, 132.3, 129.6, 129.4, 128.6, 128.5, 128.4, 128.3, 128.1, 127.6, 126.6, 126.4, 113.9, 75.6, 68.7, 61.2, 55.6 ppm. **HRMS** (ESI) (m/z) [M+H]^+^ Calcd for C_32_H_30_NO_4_S^+^ 524.1890; found 524.1884. **HPLC:** The product was analyzed by HPLC to determine the enantiomeric excess: 94% ee (Chiralpak IA-H, *n*-hexane/*i*-propanol = 95/5, 1 mL/min, 254 nm) t_R_ = 30.077 min, 37.870 min.

**19**

**(*S, Z*)-3-((*Z*)-benzylidene)-5-((4-fluorophenyl)sulfonyl)-6, 8-diphenyl-3, 4, 5, 8-tetrahydro-2*H*-1, 5-oxazocine**

The reaction of **1f** (18.3 mg, 0.05 mmol) and **2a** (19.8 mg, 0.075 mmol), Cs_2_CO_3_ (32.5 mg, 0.1 mmol), after a flash column chromatography (petroleum ether : AcOEt = 20 : 1), afforded the product **19** (19.9 mg, 78% yield).

**19**. White solid. **MP:** 138 - 139 ºC. [α]_D_ = - 6 (*c* = 0.1, CH_2_Cl_2_, 28.3 °C). **^19^F NMR (282 MHz, CDCl_3_)** *δ* – 105.40 ppm. **^1^H NMR (300 MHz, CDCl_3_)** *δ* 7.70 - 7.58 (m, 4H), 7.54 - 7.32 (m, 8H), 7.32 - 7.24 (m, 1H, overlapped with the peak of chloroform), 7.23 - 7.16 (m, 4H), 7.00 (t, *J* = 8.5 Hz, 2H), 6.92 (s, 1H), 6.37 (d, *J* = 8.9 Hz, 1H), 5.40 (d, *J* = 8.8 Hz, 1H), 5.05 (d, *J* = 13.2 Hz, 1H), 4.55 - 4.37 (m, 2H), 4.12 (d, *J* = 14.1 Hz, 1H) ppm. **^13^C NMR (75 MHz, CDCl_3_)** *δ* 164.9 (d, *J*_C-F_ = 254.8 Hz), 163.2, 141.2, 140.3, 139.0, 136.7 (d, *J*_C-F_ = 3.2 Hz), 136.2, 135.6, 133.5, 133.0, 130.1, 129.9, 129.6, 128.8, 128.6, 128.5, 128.4, 128.2, 127.7, 126.6, 126.4, 115.9 (d, *J*_C-F_ = 22.5 Hz), 75.7, 68.7, 61.4 ppm. **HRMS** (ESI) (m/z) [M+H]^+^ Calcd for C_31_H_27_FNO_3_S^+^ 512.1690; found 512.1682. **HPLC:** The product was analyzed by HPLC to determine the enantiomeric excess: 93% ee (Chiralpak IA-H, *n*-hexane/*i*-propanol = 98/2, 1 mL/min, 254 nm) t_R_ = 30.921 min, 36.721 min.

**20**

**(*S, Z*)-3-((*Z*)-benzylidene)-5-((4-cholophenyl)sulfonyl)-6, 8-diphenyl-3, 4, 5, 8-tetrahydro-2*H*-1, 5-oxazocine**

The reaction of **1g** (19.1 mg, 0.05 mmol) and **2a** (19.8 mg, 0.075 mmol), Cs_2_CO_3_ (32.5 mg, 0.1 mmol), after a flash column chromatography (petroleum ether : AcOEt = 20 : 1), afforded the product **20** (17.1 mg, 65% yield). (**Condition B** : 87% yield, 82% ee)

**20**. White solid. **MP:** 134 - 135 ºC. [α]_D_ = + 15 (*c* = 0.1, CH_2_Cl_2_, 28.5 °C). **^1^H NMR (300 MHz, CDCl_3_)** *δ* 7.63 (d, *J* = 6.9 Hz, 2H), 7.57 (d, *J* = 8.5 Hz, 2H), 7.52 - 7.33 (m, 9H), 7.32 - 7.26 (m, 2H, overlapped with the peak of chloroform), 7.23 - 7.16 (m, 4H), 6.94 (s, 1H), 6.38 (d, *J* = 8.9 Hz, 1H), 5.38 (d, *J* = 8.9 Hz, 1H), 5.06 (d, *J* = 13.0 Hz, 1H), 4.45 (s, 2H), 4.12 (d, *J* = 13.1 Hz, 1H) ppm. **^13^C NMR (75 MHz, CDCl_3_)** *δ* 141.1, 140.3, 139.1, 139.0, 136.1, 135.5, 133.5, 132.9, 129.6, 129.0, 128.8, 128.7, 128.6, 128.5, 128.4, 128.2, 127.8, 126.6, 126.4, 75.7, 68.7, 61.5 ppm. **HRMS** (ESI) (m/z) [M+H]^+^ Calcd for C_31_H_27_ClNO_3_S^+^ 528.1395; found 528.1387. **HPLC:** The product was analyzed by HPLC to determine the enantiomeric excess: 93% ee (Chiralpak IA-H, *n*-hexane/*i*-propanol = 98/2, 1 mL/min, 254 nm) t_R_ = 30.578 min, 36.608 min.

**21**

**(*S, Z*)-3-((*Z*)-benzylidene)-5-((4-bromophenyl)sulfonyl)-6, 8-diphenyl-3, 4, 5, 8-tetrahydro-2*H*-1, 5-oxazocine**

The reaction of **1h** (21.3 mg, 0.05 mmol) and **2a** (19.8 mg, 0.075 mmol), Cs_2_CO_3_ (32.5 mg, 0.1 mmol), after a flash column chromatography (petroleum ether : AcOEt = 20 : 1), afforded the product **21** (20.0 mg, 70% yield). (**Condition B** : 83% yield, 92% ee)

**21**. White solid. **MP:** 148 - 149 ºC. [α]_D_ = - 11 (*c* = 0.1, CH_2_Cl_2_, 28.7 °C). **^1^H NMR (300 MHz, CDCl_3_)** *δ* 7.63 (d, *J* = 6.9 Hz, 2H), 7.54 - 7.41 (m, 9H), 7.41 - 7.33 (m, 3H), 7.32 - 7.25 (m, 1H, overlapped with the peak of chloroform), 7.20 (d, *J* = 4.2 Hz, 4H), 6.94 (s, 1H), 6.38 (d, *J* = 8.9 Hz, 1H), 5.37 (d, *J* = 8.8 Hz, 1H), 5.06 (d, *J* = 12.9 Hz, 1H), 4.51 - 4.39 (m, 2H), 4.12 (d, *J* = 12.6 Hz, 1H) ppm. **^13^C NMR (75 MHz, CDCl_3_)** *δ* 141.1, 140.2, 139.5, 139.1, 136.1, 135.5, 133.5, 132.9, 132.0, 129.6, 128.8, 128.6, 128.5, 128.4, 128.2, 127.8, 127.5, 126.6, 126.4, 75.6, 68.7, 61.5 ppm. **HRMS** (ESI) (m/z) [M+H]^+^ Calcd for C_31_H_27_BrNO_3_S^+^ 572.0890; found 572.0885. **HPLC:** The product was analyzed by HPLC to determine the enantiomeric excess: 91% ee (Chiralpak IG-H, *n*-hexane/*i*-propanol = 95/5, 1 mL/min, 254 nm) t_R_ = 29.889 min, 42.928 min.

**22**

**(*S, Z*)-3-((*Z*)-benzylidene)-5-((2-bromophenyl)sulfonyl)-6, 8-diphenyl-3, 4, 5, 8-tetrahydro-2*H*-1, 5-oxazocine**

The reaction of **1i** (21.3 mg, 0.05 mmol) and **2a** (19.8 mg, 0.075 mmol), Cs_2_CO_3_ (32.5 mg, 0.1 mmol), after a flash column chromatography (petroleum ether : AcOEt = 20 : 1), afforded the product **22** (23.7 mg, 83% yield).

**22**. White solid. **MP:** 141 - 142 ºC. [α]_D_ = - 7 (*c* = 0.1, CH_2_Cl_2_, 28.9 °C). **^1^H NMR (300 MHz, CDCl_3_)** *δ* 7.75 - 7.71 (m, 1H), 7.70 - 7.57 (m, 5H), 7.48 - 7.30 (m, 7H), 7.24 - 7.20 (m, 2H), 7.17 - 7.12 (m, 1H), 7.10 -7.02 (m, 4H), 6.33 (d, *J* = 9.2 Hz, 1H), 5.63 (t, *J* = 10.8 Hz, 2H), 4.50 (dd, *J* = 37.9, 12.0 Hz, 3H), 4.36 (d, *J* = 13.7 Hz, 1H) ppm. **^13^C NMR (75 MHz, CDCl_3_)** *δ* 141.5, 140.3, 139.8, 139.7, 135.8, 135.8, 135.1, 134.2, 133.4, 133.2, 132.7, 129.8, 128.5, 128.5, 128.4, 128.1, 127.6, 127.3, 126.5, 126.2, 119.8, 75.1, 68.2, 63.8 ppm. **HRMS** (ESI) (m/z) [M+H]^+^ Calcd for C_31_H_27_BrNO_3_S^+^ 572.0890; found 572.0880. **HPLC:** The product was analyzed by HPLC to determine the enantiomeric excess: 90% ee (Chiralpak IG-H, *n*-hexane/*i*-propanol = 95/5, 1 mL/min, 254 nm) t_R_ = 23.787 min, 30.737 min.

**23**

**(*S, Z*)-3-((*Z*)-benzylidene)-5-((3-bromophenyl)sulfonyl)-6, 8-diphenyl-3, 4, 5, 8-tetrahydro-2*H*-1,5-oxazocine**

The reaction of **1j** (21.3 mg, 0.05 mmol) and **2a** (19.8 mg, 0.075 mmol), Cs_2_CO_3_ (32.5 mg, 0.1 mmol), after a flash column chromatography (petroleum ether : AcOEt = 20 : 1), afforded the product **23** (20.0 mg, 70% yield).

**23**. White solid. **MP:** 134 - 135 ºC. [α]_D_ = - 10 (*c* = 0.1, CH_2_Cl_2_, 28.9 °C). **^1^H NMR (600 MHz, CDCl_3_)** *δ* 7.70 (s, 1H), 7.62 – 7.54 (m, 4H), 7.48 (d, *J* = 7.7 Hz, 2H), 7.41 (t, *J* = 7.5 Hz, 2H), 7.38 – 7.29 (m, 4H), 7.27 - 7.22 (m, 1H), 7.19 - 7.16 (m, 5H), 6.90 (s, 1H), 6.32 (d, *J* = 8.9 Hz, 1H), 5.34 (d, *J* = 7.5 Hz, 1H), 5.02 (s, 1H), 4.42 (q, *J* = 12.2 Hz, 2H), 4.14 - 4.04 (m, 1H) ppm. **^13^C NMR (151 MHz, CDCl_3_)** *δ* 142.3, 141.2, 140.3, 139.2, 135.5, 135.5, 133.6, 132.8, 130.4, 130.2, 129.6, 129.0, 128.6, 128.5, 128.4, 128.2, 127.7, 126.6, 126.4, 125.7, 122.9, 75.6, 68.7, 61.6 ppm. **HRMS** (ESI) (m/z) [M+H]^+^ Calcd for C_31_H_27_BrNO_3_S^+^ 572.0890; found 572.0880. **HPLC:** The product was analyzed by HPLC to determine the enantiomeric excess: 94% ee (Chiralpak IG-H, *n*-hexane/*i*-propanol = 95/5, 1 mL/min, 254 nm) t_R_ = 24.405 min, 31.001 min.

**24**

**(*S, Z*)-3-((*Z*)-benzylidene)-6, 8-diphenyl-5-(thiophen-2-ylsulfonyl)-3, 4, 5, 8-tetrahydro-2*H*-1, 5-oxazocine**

The reaction of **1k** (17.7 mg, 0.05 mmol) and **2a** (19.8 mg, 0.075 mmol), Cs_2_CO_3_ (32.5 mg, 0.1 mmol), after a flash column chromatography (petroleum ether : AcOEt = 20 : 1), afforded the product **24** (23.0 mg, 92% yield).

**24**. White solid. **MP:** 127 - 128 ºC. [α]_D_ = - 39 (*c* = 0.1, CH_2_Cl_2_, 30.3 °C). **^1^H NMR (600 MHz, CDCl_3_)** *δ* 7.59 (d, *J* = 6.3 Hz, 2H), 7.48 (d, *J* = 7.5 Hz, 2H), 7.44 - 7.42 (m, 1H), 7.39 (t, *J* = 7.6 Hz, 2H), 7.36 - 7.29 (m, 5H), 7.27 - 7.24 (m, 2H), 7.23 - 7.21 (m, 1H), 7.21 - 7.17 (m, 2H), 6.89 - 6.85 (m, 2H), 6.33 (d, *J* = 9.0 Hz, 1H), 5.35 (d, *J* = 8.2 Hz, 1H), 4.96 - 5.06 (m, 1H), 4.39 (dd, *J* = 31.8, 12.1 Hz, 2H), 4.12 (d, *J* = 11.2 Hz, 1H) ppm. **^13^C NMR (151 MHz, CDCl_3_)** *δ* 141.5, 141.3, 140.5, 139.1, 136.2, 135.7, 133.3, 132.9, 132.4, 131.8, 129.7, 128.8, 128.6, 128.5, 128.2, 127.7, 127.3, 126.5, 75.6, 68.7, 61.5 ppm. **HRMS** (ESI) (m/z) [M+Na]^+^ Calcd for C_29_H_25_NO_3_S_2_Na^+^ 522.1168; found 522.1172. **HPLC:** The product was analyzed by HPLC to determine the enantiomeric excess: 90% ee (Chiralpak IA-H, *n*-hexane/*i*-propanol = 95/5, 1 mL/min, 254 nm) t_R_ = 19.466 min, 25.384 min.

**25**

**(*S, Z*)-3-((*Z*)-benzylidene)-8-phenyl-5-(phenylsulfonyl)-6-(*p*-tolyl)-3, 4, 5, 8-tetrahydro-2*H*-1, 5-oxazocine**

The reaction of **1l** (18.1 mg, 0.05 mmol) and **2a** (19.8 mg, 0.075 mmol), Cs_2_CO_3_ (32.5 mg, 0.1 mmol), after a flash column chromatography (petroleum ether : AcOEt = 20 : 1), afforded the product **25** (18.0 mg, 71% yield).

**25**. White solid. **MP:** 161 - 162 ºC. [α]_D_ = - 18 (*c* = 0.1, CH_2_Cl_2_, 24.6 °C). **^1^H NMR (300 MHz, CDCl_3_)** *δ* 7.72 (d, *J* = 7.6 Hz, 2H), 7.61 (d, *J* = 7.2 Hz, 2H), 7.50 - 7.31 (m, 11H), 7.15 (d, *J* = 8.1 Hz, 2H), 7.00 (d, *J* = 8.0 Hz, 2H), 6.88 (s, 1H), 6.34 (d, *J* = 9.0 Hz, 1H), 5.32 (d, *J* = 9.0 Hz, 1H), 5.04 (d, *J* = 13.4 Hz, 1H), 4.40 (q, *J* = 12.1 Hz, 2H), 4.10 (d, *J* = 13.4 Hz, 1H), 2.32 (s, 3H) ppm. **^13^C NMR (75 MHz, CDCl_3_)** *δ* 141.4, 140.7, 138.7, 135.7, 135.3, 133.5, 133.2, 132.5, 132.1, 129.6, 129.1, 128.8, 128.5, 128.4, 128.1, 127.6, 127.3, 126.4, 75.6, 68.6, 61.3, 21.2 ppm. **HRMS** (ESI) (m/z) [M+H]^+^ Calcd for C_32_H_30_NO_3_S^+^ 508.1941; found 508.1939. **HPLC:** The product was analyzed by HPLC to determine the enantiomeric excess: 91% ee (Chiralpak IA-H, *n*-hexane/*i*-propanol = 95/5, 1 mL/min, 254 nm) t_R_ = 17.574 min, 24.120 min.

**26**

**(*S, Z*)-3-((*Z*)-benzylidene)-8-phenyl-5-(phenylsulfonyl)-6-(*m*-tolyl)-3, 4, 5, 8-tetrahydro-2*H*-1, 5-oxazocine**

The reaction of **1m**(18.1 mg, 0.05 mmol) and **2a** (19.8 mg, 0.075 mmol), Cs_2_CO_3_ (32.5 mg, 0.1 mmol), after a flash column chromatography (petroleum ether : AcOEt = 20 : 1), afforded the product **26** (19.0 mg, 75% yield).

**26**. White solid. **MP:** 152 - 153 ºC. [α]_D_ = - 33 (*c* = 0.1, CH_2_Cl_2_, 23.5 °C). **^1^H NMR (300 MHz, CDCl_3_)** *δ* 7.69 (d, *J* = 7.4 Hz, 2H), 7.61 (d, *J* = 7.1 Hz, 2H), 7.54 - 7.48 (m, 3H), 7.47 - 7.42 (m, 2H), 7.41 - 7.32 (m, 6H), 7.09 - 6.99 (m, 3H), 6.92 (s, 1H), 6.88 (s, 1H), 6.35 (d, *J* = 8.9 Hz, 1H), 5.41 (d, *J* = 8.9 Hz, 1H), 5.05 (d, *J* = 13.3 Hz, 1H), 4.50 - 4.35 (m, 2H), 4.09 (d, *J* = 11.4 Hz, 1H), 2.17 (s, 3H) ppm. **^13^C NMR (75 MHz, CDCl_3_)** *δ* 141.4, 140.7, 138.8, 137.9, 135.9, 135.7, 133.2, 133.1, 132.5, 129.6, 129.5, 128.8, 128.5, 128.4, 128.3, 128.1, 127.6, 127.3, 127.1, 126.4, 123.8, 75.8, 68.6, 61.4, 21.3 ppm. **HRMS** (ESI) (m/z) [M+H]^+^ Calcd for C_32_H_30_NO_3_S^+^ 508.1941; found 508.1943. **HPLC:** The product was analyzed by HPLC to determine the enantiomeric excess: 91% ee (Chiralpak IG-H, *n*-hexane/*i*-propanol = 95/5, 1 mL/min, 254 nm) t_R_ = 29.217 min, 36.565 min.

**27**

**(*S, Z*)-3-((*Z*)-benzylidene)-8-phenyl-5-(phenylsulfonyl)-6-(*o*-tolyl)-3, 4, 5, 8-tetrahydro-2*H*-1, 5-oxazocine**

The reaction of **1n** (18.1 mg, 0.05 mmol) and **2a** (19.8 mg, 0.075 mmol), Cs_2_CO_3_ (32.5 mg, 0.1 mmol), after a flash column chromatography (petroleum ether : AcOEt = 20 : 1), afforded the product **27** (18.0 mg, 71% yield).

**27**. White solid. **MP:** 169 - 170 ºC. [α]_D_ = - 31 (*c* = 0.1, CH_2_Cl_2_, 23.7 °C). **^1^H NMR (300 MHz, CDCl_3_)** *δ* 7.65 (d, *J* = 7.4 Hz, 2H), 7.57 (d, *J* = 7.5 Hz, 2H), 7.47 - 7.30 (m, 9H), 7.26 - 7.08 (m, 4H), 6.98 (s, 1H), 6.81 (d, *J* = 4.0 Hz, 2H), 5.86 (d, *J* = 8.7 Hz, 1H), 5.60 (d, *J* = 8.7 Hz, 1H), 4.99 (d, *J* = 12.9 Hz, 1H), 4.55 (dd, *J* = 37.5, 12.2 Hz, 2H), 4.20 (d, *J* = 12.9 Hz, 1H), 2.40 (s, 3H) ppm. **^13^C NMR (75 MHz, CDCl_3_)** *δ* 141.6, 139.8, 138.8, 137.7, 137.2, 136.7, 136.6, 135.7, 133.3, 132.1, 131.0, 129.6, 128.5, 128.5, 128.4, 128.2, 128.1, 128.0, 127.5, 126.9, 126.2, 125.2, 76.4, 69.4, 61.8, 20.7 ppm. **HRMS** (ESI) (m/z) [M+H]^+^ Calcd for C_32_H_30_NO_3_S^+^ 508.1941; found 508.1934. **HPLC:** The product was analyzed by HPLC to determine the enantiomeric excess: 77% ee (Chiralpak IA-H, *n*-hexane/*i*-propanol = 95/5, 1 mL/min, 254 nm) t_R_ = 12.008 min, 16.684 min.

**28**

**(*S, Z*)-3-((*Z*)-benzylidene)-6-(4-chlorophenyl)-8-phenyl-5-(phenylsulfonyl)-3, 4, 5, 8-tetrahydro-2*H*-1, 5-oxazocine**

The reaction of **1o** (19.1 mg, 0.05 mmol) and **2a** (19.8 mg, 0.075 mmol), Cs_2_CO_3_ (32.5 mg, 0.1 mmol), after a flash column chromatography (petroleum ether : AcOEt = 20 : 1), afforded the product **28** (21.1 mg, 80% yield).

**28**. White solid. **MP:** 167 - 168 ºC. [α]_D_ = - 18 (*c* = 0.1, CH_2_Cl_2_, 29.2 °C). **^1^H NMR (300 MHz, CDCl_3_)** *δ* 7.72 (d, *J* = 7.5 Hz, 2H), 7.60 (d, *J* = 7.3 Hz, 2H), 7.56 - 7.49 (m, 1H), 7.48 - 7.41 (m, 5H), 7.40 - 7.29 (m, 5H), 7.22 - 7.12 (m, 4H), 6.88 (s, 1H), 6.36 (d, *J* = 8.9 Hz, 1H), 5.30 (d, *J* = 8.9 Hz, 1H), 5.04 (d, *J* = 13.6 Hz, 1H), 4.40 (q, *J* = 12.2 Hz, 2H), 4.09 (d, *J* = 13.5 Hz, 1H) ppm. **^13^C NMR (75 MHz, CDCl_3_)** *δ* 141.0, 140.5, 139.5, 139.0, 135.5, 134.9, 134.6, 133.7, 132.9, 132.7, 129.6, 129.0, 128.6, 128.5, 128.2, 127.8, 127.3, 126.4, 75.7, 68.8, 61.3 ppm. **HRMS** (ESI) (m/z) [M+H]^+^ Calcd for C_31_H_27_ClNO_3_S^+^ 528.1395; found 528.1387. **HPLC:** The product was analyzed by HPLC to determine the enantiomeric excess: 91% ee (Chiralpak IA-H, *n*-hexane/*i*-propanol = 98/2, 1 mL/min, 254 nm) t_R_ = 35.952 min, 42.456 min.

**29**

**(*S, Z*)-3-((*Z*)-benzylidene)-6-(4-bromophenyl)-8-phenyl-5-(phenylsulfonyl)-3, 4, 5, 8-tetrahydro-2*H*-1, 5-oxazocine**

The reaction of **1p** (21.3 mg, 0.05 mmol) and **2a** (19.8 mg, 0.075 mmol), Cs_2_CO_3_ (32.5 mg, 0.1 mmol), after a flash column chromatography (petroleum ether : AcOEt = 20 : 1), afforded the product **29** (20.3 mg, 71% yield).

**29**. White solid. **MP:** 121 - 122 ºC. [α]_D_ = - 18 (*c* = 0.1, CH_2_Cl_2_, 29.2 °C). **^1^H NMR (300 MHz, CDCl_3_)** *δ* 7.72 (d, *J* = 7.7 Hz, 2H), 7.62 (d, *J* = 7.3 Hz, 2H), 7.62 - 7.56 (m, 2H), 7.54 - 7.49 (m, 1H), 7.45 - 7.28 (m, 12H), 7.13 - 7.07 (m, 2H), 6.86 (s, 1H), 6.36 (d, *J* = 8.9 Hz, 1H), 5.28 (d, *J* = 8.9 Hz, 1H), 5.03 (d, *J* = 13.7 Hz, 1H), 4.39 (dd, *J* = 25.5, 12.2 Hz, 2H), 4.13 - 4.04 (m, 1H) ppm. **^13^C NMR (75 MHz, CDCl_3_)** *δ* 141.0, 140.5, 138.9, 135.5, 135.4, 133.8, 132.8, 132.7, 131.5, 129.6, 129.0, 128.6, 128.4, 128.2, 128.0, 127.8, 127.2, 126.4, 122.8, 75.7, 68.8, 61.3 ppm. **HRMS** (ESI) (m/z) [M+H]^+^ Calcd for C_31_H_27_BrNO_3_S^+^ 572.0890; found 572.0882. **HPLC:** The product was analyzed by HPLC to determine the enantiomeric excess: 90% ee (Chiralpak IA-H, *n*-hexane/*i*-propanol = 98/2, 1 mL/min, 254 nm) t_R_ = 38.971 min, 46.646 min.

**30**

**(*S, Z*)-3-((*Z*)-benzylidene)-6-(3-bromophenyl)-8-phenyl-5-(phenylsulfonyl)-3, 4, 5, 8-tetrahydro-2*H*-1, 5-oxazocine**

The reaction of **1q** (21.3 mg, 0.05 mmol) and **2a** (19.8 mg, 0.075 mmol), Cs_2_CO_3_ (32.5 mg, 0.1 mmol), after a flash column chromatography (petroleum ether : AcOEt = 20 : 1), afforded the product **30** (18.6 mg, 65% yield).

**30**. White solid. **MP:** 134 - 135 ºC. [α]_D_ = - 4 (*c* = 0.1, CH_2_Cl_2_, 29.1 °C). **^1^H NMR (300 MHz, CDCl_3_)** *δ* 7.71 (d, *J* = 7.4 Hz, 2H), 7.61 (d, *J* = 7.3 Hz, 2H), 7.56 - 7.46 (m, 4H), 7.46 - 7.33 (m, 8H), 7.24 - 7.15 (m, 2H), 7.06 (t, *J* = 7.8 Hz, 1H), 6.90 (s, 1H), 6.39 (d, *J* = 8.9 Hz, 1H), 5.39 (d, *J* = 8.8 Hz, 1H), 5.06 (d, *J* = 13.3 Hz, 1H), 4.51 – 4.36 (m, 2H), 4.08 (d, *J* = 13.2 Hz, 1H) ppm. **^13^C NMR (75 MHz, CDCl_3_)** *δ* 141.0, 140.3, 139.1, 138.3, 135.5, 134.7, 132.9, 132.8, 131.6, 129.9, 129.6, 129.3, 129.0, 128.6, 128.5, 128.2, 127.8, 127.2, 126.4, 125.2, 122.5, 75.7, 68.8, 61.4 ppm. **HRMS** (ESI) (m/z) [M+H]^+^ Calcd for C_31_H_27_BrNO_3_S^+^ 572.0890; found 572.0881. **HPLC:** The product was analyzed by HPLC to determine the enantiomeric excess: 86% ee (Chiralpak IA-H, *n*-hexane/*i*-propanol = 95/5, 1 mL/min, 254 nm) t_R_ = 14.367 min, 16.621 min.

**31**

**(*S*, *Z*)-3-((*Z*)-benzylidene)-6-(2-nitrophenyl)-8-phenyl-5-(phenylsulfonyl)-3, 4, 5, 8-tetrahydro-2*H*-1,5-oxazocine**

The reaction of **1r** (19.6 mg, 0.05 mmol) and **2a** (19.8 mg, 0.075 mmol), Cs_2_CO_3_ (32.5 mg, 0.1 mmol), after a flash column chromatography (petroleum ether : AcOEt = 20 : 1), afforded the product **31** (21.8 mg, 81% yield).

**31.** Yellow oil. **^1^H NMR (300 MHz, CDCl_3_)** *δ* 8.41 (m, 1H), 8.21 (d, *J* = 8.2 Hz, 1H), 7.84 (d, *J* = 7.6 Hz, 1H), 7.68 (d, *J* = 7.9 Hz, 2H), 7.63 - 7.56 (m, 3H), 7.54 - 7.48 (m, 1H), 7.44 - 7.31 (m, 5H), 7.26 - 7.22 (m, 1H), 7.20 - 7.16 (m, 4H), 6.97 (s, 1H), 6.26 (d, *J* = 9.0 Hz, 1H), 5.55 (d, *J* = 9.0 Hz, 1H), 5.09 (d, *J* = 13.3 Hz, 1H), 4.47 (s, 2H), 4.10 (d, *J* = 13.3 Hz, 1H) ppm. **^13^C NMR (75 MHz, CDCl_3_)** *δ* 148.4, 143.6, 141.7, 140.5, 139.6, 135.5, 135.0, 132.8, 132.7, 132.5, 131.7, 129.6, 129.4, 129.0, 128.9, 128.5, 128.4, 128.3, 127.2, 126.6, 122.6, 121.1, 74.5, 68.6, 61.3 ppm. **HRMS** (ESI) (m/z) [M+H]^+^ Calcd for C_31_H_27_N_2_O_5_S^+^ 539.1635; found 539.1638. **HPLC**: The product was analyzed by HPLC to determine the enantiomeric excess: 90% ee (Chiralpak IA-H, *n*-hexane/*i*-propanol = 95/5, 1 mL/min, 254 nm) t_R_ = 27.826 min, 30.408 min.

**32**

**(*S, Z*)-3-((*Z*)-benzylidene)-8-phenyl-5-(phenylsulfonyl)-6-(3-(trifluoromethyl)phenyl)-3, 4, 5, 8-tetrahydro-2*H*-1, 5-oxazocine**

The reaction of **1s** (20.8 mg, 0.05 mmol) and **2a** (19.8 mg, 0.075 mmol), Cs_2_CO_3_ (32.5 mg, 0.1 mmol), after a flash column chromatography (petroleum ether : AcOEt = 20 : 1), afforded the product **32** (17.4 mg, 62% yield). (**Condition B** : 79% yield, 83% ee)

**32**. White solid. **MP:** 168 - 169 ºC. [α]_D_ = - 8 (*c* = 0.1, CH_2_Cl_2_, 29.1 °C). **^19^F NMR (282 MHz, CDCl_3_)** *δ* - 62.95 ppm. **^1^H NMR (300 MHz, CDCl_3_)** *δ* 7.79 – 7.74 (m, 1H), 7.70 (d, *J* = 7.5 Hz, 2H), 7.66 - 7.59 (m, 3H), 7.58 - 7.47 (m, 3H), 7.44 - 7.32 (m, 5H), 7.26 - 7.18 (m, 4H), 7.14 - 7.01 (m, 1H), 6.96 (s, 1H), 6.31 (d, *J* = 9.0 Hz, 1H), 5.44 (d, *J* = 8.9 Hz, 1H), 5.10 (d, *J* = 13.4 Hz, 1H), 4.45 (s, 2H), 4.12 (d, *J* = 13.4 Hz, 1H) ppm. **^13^C NMR (75 MHz, CDCl_3_)** *δ* 142.4, 141.3, 140.5, 139.3, 135.8, 135.5, 133.2, 130.8 (q, *J*_C–F_ = 32.4 Hz), 130.1, 130.0, 129.6, 129.0, 128.9, 128.5, 128.4, 128.3, 127.7 (q, *J*_C–F_ = 243.9 Hz), 127.2, 126.5, 124.5 (q, *J*_C–F_ = 3.7 Hz), 123.0 (q, *J*_C–F_ = 3.8 Hz), 122.4, 74.9, 68.7, 61.4 ppm. **HRMS** (ESI) (m/z) [M+H]^+^ Calcd for C_32_H_27_F_3_NO_3_S^+^ 562.1658; found 562.1651. **HPLC:** The product was analyzed by HPLC to determine the enantiomeric excess: 86% ee (Chiralpak IG-H, *n*-hexane/*i*-propanol = 95/5, 1 mL/min, 254 nm) t_R_ = 13.727 min, 17.716 min.

**33**

**(*S, Z*)-3-((*Z*)-benzylidene)-6-(3, 5-dimethylphenyl)-8-phenyl-5-(phenylsulfonyl)-3, 4, 5, 8-tetrahydro-2*H*-1, 5-oxazocine**

The reaction of **1t** (17.4 mg, 0.05 mmol) and **2a** (21.9 mg, 0.075 mmol), Cs_2_CO_3_ (32.5 mg, 0.1 mmol) at room temperature, after a flash column chromatography (petroleum ether : AcOEt = 20 : 1), afforded the product **33** (17.2 mg, 66% yield). (**Condition B** : 77% yield, 99% ee)

**33**. White solid. **MP:** 71 - 72 ºC. [α]_D_ = - 11 (*c* = 0.1, CH_2_Cl_2_, 29.2 °C). **^1^H NMR (300 MHz, CDCl_3_)** *δ* 7.69 (d, *J* = 7.5 Hz, 2H), 7.62 (d, *J* = 7.3 Hz, 2H), 7.56 - 7.48 (m, 3H), 7.47 - 7.31 (m, 8H), 6.89 (s, 1H), 6.86 (s, 1H), 6.73 (s, 2H), 6.35 (d, *J* = 9.0 Hz, 1H), 5.46 (d, *J* = 8.9 Hz, 1H), 5.05 (d, *J* = 13.8 Hz, 1H), 4.44 (s, 2H), 4.06 (d, *J* = 13.5 Hz, 1H), 2.13 (s, 6H) ppm. **^13^C NMR (75 MHz, CDCl_3_)** *δ* 141.5, 140.8, 138.7, 137.8, 135.7, 135.6, 133.3, 132.8, 132.4, 130.4, 129.6, 128.7, 128.5, 128.4, 128.0, 127.6, 127.3, 126.4, 124.4, 75.8, 68.5, 61.3, 21.2 ppm. **HRMS** (ESI) (m/z) [M+H]^+^ Calcd for C_33_H_32_NO_3_S^+^ 522.2097; found 522.2092. **HPLC**: The product was analyzed by HPLC to determine the enantiomeric excess: 90% ee (Chiralpak IA-H, *n*-hexane/*i*-propanol = 95/5, 1 mL/min, 254 nm) t_R_ = 9.917 min, 11.092 min.

**34**

**(*S, Z*)-3-((*Z*)-benzylidene)-8-phenyl-5-(phenylsulfonyl)-6-(thiophen-3-yl)-3, 4, 5, 8-tetrahydro-2*H*-1, 5-oxazocine**

The reaction of **1u** (17.7 mg, 0.05 mmol) and **2a** (19.8 mg, 0.075 mmol), Cs_2_CO_3_ (32.5 mg, 0.1 mmol), after a flash column chromatography (petroleum ether : AcOEt = 20 : 1), afforded the product **34** (11.2mg, 45% yield).

**34**. White solid. **MP:** 181 - 182 ºC. [α]_D_ = - 39 (*c* = 0.1, CH_2_Cl_2_, 30.3 °C). **^1^H NMR (300 MHz, CDCl_3_)** *δ* 7.80 (d, *J* = 7.5 Hz, 2H), 7.59 (t, *J* = 7.6 Hz, 2H), 7.51 - 7.31 (m, 11H), 7.15 (dd, *J* = 5.0, 3.0 Hz, 1H), 6.99 (d, *J* = 4.8 Hz, 1H), 6.92 (d, *J* = 1.7 Hz, 1H), 6.78 (s, 1H), 6.33 (d, *J* = 8.9 Hz, 1H), 5.37 (d, *J* = 8.7 Hz, 1H), 4.97 (d, *J* = 13.6 Hz, 1H), 4.38 (dd, *J* = 35.5, 12.2 Hz, 2H), 4.03 (d, *J* = 13.4 Hz, 1H) ppm. **^13^C NMR (75 MHz, CDCl_3_)** *δ* 141.3, 140.8, 138.3, 137.9, 136.0, 135.6, 133.3, 132.7, 132.7, 129.5, 129.0, 128.5, 128.4, 128.1, 127.6, 127.2, 126.4, 126.2, 125.5, 123.5, 75.7, 68.5, 60.3 ppm. **HRMS** (ESI) (m/z) [M+Na]^+^ Calcd for C_29_H_25_NO_3_S_2_Na^+^ 522.1168; found 522.1168. **HPLC:** The product was analyzed by HPLC to determine the enantiomeric excess: 96% ee (Chiralpak IA-H, *n*-hexane/*i*-propanol = 95/5, 1 mL/min, 254 nm) t_R_ = 23.135 min, 29.306 min.

**35**

**(*S, Z*)-3-((*Z*)-benzylidene)-6-phenyl-5-(phenylsulfonyl)-8-(*p*-tolyl)-3, 4, 5, 8-tetrahydro-2*H*-1, 5-oxazocine**

The reaction of **1v** (18.1 mg, 0.05 mmol) and **2a** (19.8 mg, 0.075 mmol), Cs_2_CO_3_ (32.5 mg, 0.1 mmol), after a flash column chromatography (petroleum ether : AcOEt = 20 : 1), afforded the product **35** (13.9 mg, 55% yield). (**Condition B** : 85% yield, 20% ee)

**35**. White solid. **MP:** 126 - 127 ºC. [α]_D_ = - 20 (*c* = 0.1, CH_2_Cl_2_, 28.0 °C). **^1^H NMR (300 MHz, CDCl_3_)** *δ* 7.69 (d, *J* = 7.6 Hz, 2H), 7.60 (d, *J* = 7.3 Hz, 2H), 7.49 (t, *J* = 7.4 Hz, 1H), 7.41 - 7.30 (m, 7H), 7.26 - 7.14 (m, 7H), 6.88 (s, 1H), 6.36 (d, *J* = 9.0 Hz, 1H), 5.29 (d, *J* = 8.9 Hz, 1H), 5.05 (d, *J* = 13.4 Hz, 1H), 4.39 (q, *J* = 12.2 Hz, 2H), 4.13 (t, *J* = 9.8 Hz, 1H), 2.41 (s, 3H) ppm. **^13^C NMR (75 MHz, CDCl_3_)** *δ* 140.6, 138.8, 138.3, 137.4, 136.4, 135.6, 133.4, 133.2, 132.5, 129.6, 129.2, 128.8, 128.6, 128.4, 128.3, 128.1, 127.3, 126.5, 126.4, 75.6, 68.6, 61.4, 21.2 ppm. **HRMS** (ESI) (m/z) [M+H]^+^ Calcd for C_32_H_30_NO_3_S^+^ 508.1941; found 508.1934. **HPLC:** The product was analyzed by HPLC to determine the enantiomeric excess: 94% ee (Chiralpak IA-H, *n*-hexane/*i*-propanol = 95/5, 1 mL/min, 254 nm) t_R_ = 21.892 min, 22.942 min.

**36**

**(*S, Z*)-3-((*Z*)-benzylidene)-6-phenyl-5-(phenylsulfonyl)-8-(*m*-tolyl)-3, 4, 5, 8-tetrahydro-2*H*-1, 5-oxazocine**

The reaction of **1w** (18.1 mg, 0.05 mmol) and **2a** (19.8 mg, 0.075 mmol), Cs_2_CO_3_ (32.5 mg, 0.1 mmol), after a flash column chromatography (petroleum ether : AcOEt = 20 : 1), afforded the product **36** (17.7 mg, 70% yield).

**36**. White solid. **MP:** 141 - 142 ºC. [α]_D_ = - 20 (*c* = 0.1, CH_2_Cl_2_, 28.0 °C). **^1^H NMR (300 MHz, CDCl_3_)** *δ* 7.71 (d, *J* = 7.6 Hz, 2H), 7.62 (d, *J* = 7.2 Hz, 2H), 7.51 (t, *J* = 7.4 Hz, 1H), 7.44 - 7.27 (m, 9H), 7.25 - 7.15 (m, 5H), 6.90 (s, 1H), 6.40 (d, *J* = 8.9 Hz, 1H), 5.32 (d, *J* = 8.9 Hz, 1H), 5.07 (d, *J* = 13.3 Hz, 1H), 4.43 (q, *J* = 12.1 Hz, 2H), 4.20 – 4.07 (m, 1H), 2.44 (s, 3H) ppm. **^13^C NMR (75 MHz, CDCl_3_)** *δ* 141.2, 140.6, 140.3, 138.8, 138.2, 136.4, 135.6, 133.4, 133.2, 132.5, 129.6, 128.9, 128.6, 128.5, 128.4, 128.4, 128.1, 127.3, 127.2, 126.5, 123.6, 75.9, 68.7, 61.4, 21.6 ppm. **HRMS** (ESI) (m/z) [M+H]^+^ Calcd for C_32_H_30_NO_3_S^+^ 508.1941; found 508.1948. **HPLC:** The product was analyzed by HPLC to determine the enantiomeric excess: 86% ee (Chiralpak IG-H, *n*-hexane/*i*-propanol = 95/5, 1 mL/min, 254 nm) t_R_ = 26.669 min, 34.777 min.

**37**

**(*S, Z*)-3-((*Z*)-benzylidene)-6-phenyl-5-(phenylsulfonyl)-8-(*o*-tolyl)-3, 4, 5, 8-tetrahydro-2*H*-1, 5-oxazocine**

The reaction of **1x** (18.1 mg, 0.05 mmol) and **2a** (19.8 mg, 0.075 mmol), Cs_2_CO_3_ (32.5 mg, 0.1 mmol), after a flash column chromatography (petroleum ether : AcOEt = 20 : 1), afforded the product **37** (19.0 mg, 75% yield).

**37**. White solid. **MP:** 142 - 143 ºC. [α]_D_ = - 14 (*c* = 0.1, CH_2_Cl_2_, 22.9 °C). **^1^H NMR (300 MHz, CDCl_3_)** *δ* 7.70 - 7.47 (m, 7H), 7.42 - 7.29 (m, 6H), 7.26 - 7.18 (m, 2H), 7.09 (q, *J* = 8.0 Hz, 4H), 6.80 (s, 1H), 6.31 (d, *J* = 8.4 Hz, 1H), 5.70 (d, *J* = 8.5 Hz, 1H), 5.02 (d, *J* = 12.3 Hz, 1H), 4.52 (dd, *J* = 66.8, 12.4 Hz, 2H), 4.14 (d, *J* = 12.4 Hz, 1H), 2.52 (s, 3H) ppm. **^13^C NMR (75 MHz, CDCl_3_)** *δ* 140.1, 139.7, 137.6, 136.7, 135.9, 135.7, 134.5, 133.5, 132.5, 130.7, 129.5, 128.7, 128.5, 128.4, 128.2, 128.1, 127.7, 127.5, 127.1, 127.0, 126.4, 126.2, 74.6, 69.3, 61.5, 19.8 ppm. **HRMS** (ESI) (m/z) [M+H]^+^ Calcd for C_32_H_30_NO_3_S^+^ 508.1941; found 508.1943. **HPLC:** The product was analyzed by HPLC to determine the enantiomeric excess: 98% ee (Chiralpak IA-H, *n*-hexane/*i*-propanol = 95/5, 1 mL/min, 254 nm) t_R_ = 12.788 min, 13.914 min.

**38**

**(*S, Z*)-3-((*Z*)-benzylidene)-6-phenyl-5-(phenylsulfonyl)-8-(4-(trifluoromethyl)phenyl)-3, 4, 5, 8-tetrahydro-2*H*-1, 5-oxazocine**

The reaction of **1y** (20.8 mg, 0.05 mmol) and **2a** (19.8 mg, 0.075 mmol), Cs_2_CO_3_ (32.5 mg, 0.1 mmol), after a flash column chromatography (petroleum ether : AcOEt = 20 : 1), afforded the product **38** (21.0 mg, 75% yield).

**38**. White solid. **MP:** 148 - 149 ºC. [α]_D_ = - 44 (*c* = 0.1, CH_2_Cl_2_, 22.1 °C). **^19^F NMR (282 MHz, CDCl_3_)** *δ* - 63.29 ppm. **^1^H NMR (300 MHz, CDCl_3_)** *δ* 7.71 (d, *J* = 7.5 Hz, 2H), 7.59 (d, *J* = 7.2 Hz, 2H), 7.52 (t, *J* = 7.5 Hz, 1H), 7.48 – 7.41 (m, 6H), 7.41 – 7.30 (m, 8H), 6.89 (s, 1H), 6.46 (d, *J* = 8.9 Hz, 1H), 5.30 (d, *J* = 8.8 Hz, 1H), 5.08 (d, *J* = 13.1 Hz, 1H), 4.42 (dd, *J* = 27.7, 12.2 Hz, 2H), 4.15 (d, *J* = 12.9 Hz, 1H) ppm. **^13^C NMR (75 MHz, CDCl_3_)** *δ* 140.8, 140.4, 140.1, 139.2, 135.5, 135.4, 132.8, 132.7, 130.7, 130.2, 129.6, 129.3, 129.0, 128.6, 128.5, 128.3, 127.9, 127.2, 126.8, 126.4, 125.3 (d, *J*_C–F_ = 3.7 Hz), 123.9 (q, *J*_C–F_ = 270.5 Hz), 75.7, 69.0, 61.5 ppm. **HRMS** (ESI) (m/z) [M+H]^+^ Calcd for C_32_H_27_F_3_NO_3_S^+^ 562.1658; found 562.1658. **HPLC:** The product was analyzed by HPLC to determine the enantiomeric excess: 92% ee (Chiralpak IA-H, *n*-hexane/*i*-propanol = 98/2, 1 mL/min, 254 nm) t_R_ = 28.493 min, 34.206 min.

**39**

**4-((*S, Z*)-7-((*Z*)-benzylidene)-4-phenyl-5-(phenylsulfonyl)-5, 6, 7, 8-tetrahydro-2*H*-1, 5-oxazocin-2-yl)benzonitrile**

The reaction of **1z** (18.6 mg, 0.05 mmol) and **2a** (19.8 mg, 0.075 mmol), Cs_2_CO_3_ (32.5 mg, 0.1 mmol), after a flash column chromatography (petroleum ether : AcOEt = 20 : 1), afforded the product **39** (16.1 mg, 62% yield).

**39**. White solid. **MP:** 132 - 133 ºC . [α]_D_ = - 8 (*c* = 0.1, CH_2_Cl_2_, 28.1 °C). **^1^H NMR (300 MHz, CDCl_3_)** *δ* 7.72 (d, *J* = 8.3 Hz, 2H), 7.68 - 7.61 (m, 4H), 7.61 - 7.46 (m, 4H), 7.42 - 7.31 (m, 5H), 7.26 - 7.20 (m, 1H), 7.15 - 7.10 (m, 3H), 6.93 (s, 1H), 6.20 (d, *J* = 9.0 Hz, 1H), 5.57 (d, *J* = 8.9 Hz, 1H), 5.06 (d, *J* = 14.1 Hz, 1H), 4.45 (s, 2H), 4.06 (d, *J* = 13.1 Hz, 1H) ppm. **^13^C NMR (75 MHz, CDCl_3_)** *δ* 147.9, 146.8, 142.1, 141.5, 140.5, 139.5, 135.5, 132.3, 129.5, 129.0, 128.9, 128.5, 128.4, 128.3, 127.2, 126.9, 126.6, 119.0, 111.2, 74.9, 68.6, 61.3 ppm. **HRMS** (ESI) (m/z) [M+H]^+^ Calcd for C_32_H_27_N_2_O_3_S^+^ 519.1737; found 519.1729. **HPLC:** The product was analyzed by HPLC to determine the enantiomeric excess: 92% ee (Chiralpak IA-H, *n*-hexane/*i*-propanol = 95/5, 1 mL/min, 254 nm) t_R_ = 58.433 min, 69.910 min.

**40**

**(*S, Z*)-3-((*Z*)-benzylidene)-8-(4-fluorophenyl)-6-phenyl-5-(phenylsulfonyl)-3, 4, 5, 8-tetrahydro-2*H*-1, 5-oxazocine**

The reaction of **1I** (18.3 mg, 0.05 mmol) and **2a** (19.8 mg, 0.075 mmol), Cs_2_CO_3_ (32.5 mg, 0.1 mmol), after a flash column chromatography (petroleum ether : AcOEt = 20 : 1), afforded the product **40** (16.9 mg, 66% yield). (**Condition B** : 85% yield, 98% ee)

**40**. White solid. **MP:** 120 - 121 ºC. [α]_D_ = - 7 (*c* = 0.1, CH_2_Cl_2_, 19.5 °C). **^19^F NMR (282 MHz, CDCl_3_)** *δ* - 117.05 ppm. **^1^H NMR (300 MHz, CDCl_3_)** *δ* 7.68 (d, *J* = 7.6 Hz, 2H), 7.61 (d, *J* = 7.2 Hz, 2H), 7.54 - 7.44 (m, 3H), 7.41 - 7.27 (m, 6H), 7.22 - 7.08 (m, 6H), 6.92 (s, 1H), 6.32 (d, *J* = 9.0 Hz, 1H), 5.40 (d, *J* = 8.8 Hz, 1H), 5.07 (d, *J* = 13.6 Hz, 1H), 4.47 - 4.39 (m, 2H), 4.10 (d, *J* = 13.1 Hz, 1H) ppm. **^13^C NMR (75 MHz, CDCl_3_)** *δ* 162.3 (d, *J*_C-F_ = 245.6 Hz), 140.6, 139.0, 137.2 (d, *J*_C-F_ = 3.2 Hz), 136.0, 135.6, 133.1, 133.0, 132.6, 129.6, 128.9, 128.8, 128.5, 128.4, 128.2, 128.1, 128.0, 127.3, 126.5, 115.3 (d, *J*_C-F_ = 21.4 Hz), 75.1, 68.6, 61.4 ppm. **HRMS** (ESI) (m/z) [M+H]^+^ Calcd for C_31_H_27_FNO_3_S^+^ 512.1690; found 512.1697. **HPLC:** The product was analyzed by HPLC to determine the enantiomeric excess: 92% ee (Chiralpak IA-H, *n*-hexane/*i*-propanol = 95/5, 1 mL/min, 254 nm) t_R_ = 19.077 min, 21.479 min.

**41**

**(*S, Z*)-3-((*Z*)-benzylidene)-8-(3-fluorophenyl)-6-phenyl-5-(phenylsulfonyl)-3, 4, 5, 8-tetrahydro-2*H*-1, 5-oxazocine**

The reaction of **1II** (18.3 mg, 0.05 mmol) and **2a** (19.8 mg, 0.075 mmol), Cs_2_CO_3_ (32.5 mg, 0.1 mmol), after a flash column chromatography (petroleum ether : AcOEt = 20 : 1), afforded the product **41** (19.4 mg, 76% yield).

**41**. White solid. **MP:** 110 - 111 ºC. [α]_D_ = - 41 (*c* = 0.1, CH_2_Cl_2_, 30.2 °C). **^19^F NMR (282 MHz, CDCl_3_)** *δ* - 113.37 ppm. **^1^H NMR (300 MHz, CDCl_3_)** *δ* 7.67 (d, *J* = 7.7 Hz, 2H), 7.59 (d, *J* = 7.3 Hz, 2H), 7.49 (t, *J* = 7.4 Hz, 1H), 7.43 - 7.30 (m, 6H), 7.25 - 7.12 (m, 7H), 7.09 - 6.99 (m, 1H), 6.90 (s, 1H), 6.29 (d, *J* = 9.0 Hz, 1H), 5.39 (d, *J* = 8.9 Hz, 1H), 5.06 (d, *J* = 13.6 Hz, 1H), 4.49 - 4.34 (m, 2H), 4.09 (d, *J* = 13.4 Hz, 1H) ppm. **^13^C NMR (75 MHz, CDCl_3_)** *δ* 163.0 (d, *J*_C-F_ = 245.6 Hz), 144.0 (d, *J*_C-F_ = 7.3 Hz), 140.5, 139.1, 135.9, 135.5, 132.8, 132.7, 132.6, 130.0, 129.9, 129.5, 128.9, 128.8, 128.4, 128.4, 128.2, 127.3, 126.5, 122.0 (d, *J*_C-F_ = 2.8 Hz), 114.4, 113.4, 75.0, 68.6, 61.3 ppm. **HRMS** (ESI) (m/z) [M+H]^+^ Calcd for C_31_H_27_FNO_3_S^+^ 512.1690; found 512.1696. **HPLC:** The product was analyzed by HPLC to determine the enantiomeric excess: 94% ee (Chiralpak IA-H, *n*-hexane/*i*-propanol = 95/5, 1 mL/min, 254 nm) t_R_ = 12.842 min, 15.423 min.

**42**

**(*S, Z*)-3-((*Z*)-benzylidene)-8-(2-fluorophenyl)-6-phenyl-5-(phenylsulfonyl)-3, 4, 5, 8-tetrahydro-2*H*-1, 5-oxazocine**

The reaction of **1III** (18.3 mg, 0.05 mmol) and **2a** (19.8 mg, 0.075 mmol), Cs_2_CO_3_ (32.5 mg, 0.1 mmol), after a flash column chromatography (petroleum ether : AcOEt = 20 : 1), afforded the product **42** (15.3 mg, 60% yield).

**42**. White solid. **MP:** 126 - 127 ºC. [α]_D_ = - 21 (*c* = 0.1, CH_2_Cl_2_, 30.3 °C). **^19^F NMR (282 MHz, CDCl_3_)** *δ* - 115.45 ppm. **^1^H NMR (300 MHz, CDCl_3_)** *δ* 7.74 (d, *J* = 7.6 Hz, 2H), 7.60 - 7.49 (m, 5H), 7.41 - 7.28 (m, 7H), 7.26 - 7.11 (m, 5H), 6.81 (s, 1H), 6.49 (d, *J* = 8.5 Hz, 1H), 5.53 (d, *J* = 8.6 Hz, 1H), 5.00 - 4.91 (m, 1H), 4.49 (d, *J* = 12.4 Hz, 1H), 4.34 - 4.14 (m, 2H) ppm. **^13^C NMR (75 MHz, CDCl_3_)** δ 160.3 (d, *J*_C–F_ = 248.9 Hz), 140.4, 138.2 (d, *J*_C–F_ = 6.2 Hz), 136.8, 135.6, 133.1, 132.6, 131.8, 129.7, 129.6, 129.5, 129.1, 128.9, 128.7, 128.41, 28.39, 128.1, 127.9, 127.5, 126.6, 126.4, 124.4 (d, *J*_C–F_ = 3.5 Hz), 115.9 75.1, (d, *J*_C–F_ = 21.7 Hz), 72.0, 69.2, 61.2 ppm. **HRMS** (ESI) (m/z) [M+H]^+^ Calcd for C_31_H_27_FNO_3_S^+^ 512.1690; found 512.1694. **HPLC:** The product was analyzed by HPLC to determine the enantiomeric excess: > 99% ee (Chiralpak IG-H, *n*-hexane/*i*-propanol = 90/10, 1 mL/min, 254 nm) t_R_ = 34.172 min, 58.913 min.

**43**

**(*S, Z*)-3-((*Z*)-benzylidene)-8-(4-chlorophenyl)-6-phenyl-5-(phenylsulfonyl)-3, 4, 5, 8-tetrahydro-2*H*-1, 5-oxazocine**

The reaction of **1IV** (19.1 mg, 0.05 mmol) and **2a** (19.8 mg, 0.075 mmol), Cs_2_CO_3_ (32.5 mg, 0.1 mmol), after a flash column chromatography (petroleum ether : AcOEt = 20 : 1), afforded the product **43** (20.3 mg, 77% yield). (**Condition B** : 81% yield, 98% ee)

**43**. White solid. **MP:** 137 - 138 ºC. [α]_D_ = - 9 (*c* = 0.1, CH_2_Cl_2_, 24 °C). **^1^H NMR (300 MHz, CDCl_3_)** *δ* 7.67 (d, *J* = 7.6 Hz, 2H), 7.59 (d, *J* = 7.1 Hz, 2H), 7.54 - 7.45 (m, 2H), 7.43 - 7.41 (m, 3H), 7.39 - 7.31 (m, 5H), 7.25 - 7.11 (m, 5H), 6.92 (s, 1H), 6.28 (d, *J* = 9.0 Hz, 1H), 5.40 (d, *J* = 8.9 Hz, 1H), 5.06 (d, *J* = 13.4 Hz, 1H), 4.48 - 4.36 (m, 2H), 4.09 (d, *J* = 13.4 Hz, 1H) ppm. **^13^C NMR (75 MHz, CDCl_3_)** *δ* 140.6, 139.9, 139.1, 135.9, 135.6, 133.3, 132.9, 132.8, 132.6, 129.6, 128.9, 128.8, 128.6, 128.4, 128.4, 128.2, 127.7, 127.3, 126.5, 75.0, 68.6, 61.4 ppm. **HRMS** (ESI) (m/z) [M+H]^+^ Calcd for C_31_H_27_ClNO_3_S^+^ 528.1395; found 528.1390. **HPLC:** The product was analyzed by HPLC to determine the enantiomeric excess: 94% ee (Chiralpak IG-H, *n*-hexane/*i*-propanol = 95/5, 1 mL/min, 254 nm) t_R_ = 32.726 min, 52.394 min.

**44**

**(*S, Z*)-3-((*Z*)-benzylidene)-8-(4-bromophenyl)-6-phenyl-5-(phenylsulfonyl)-3, 4, 5, 8-tetrahydro-2*H*-1, 5-oxazocine**

The reaction of **1V** (21.3 mg, 0.05 mmol) and **2a** (19.8 mg, 0.075 mmol), Cs_2_CO_3_ (32.5 mg, 0.1 mmol), after a flash column chromatography (petroleum ether : AcOEt = 20 : 1), afforded the product **44** (20.0 mg, 70% yield). (**Condition B** : 86% yield, 98% ee)

**44**. White solid. **MP:** 127 - 128 ºC. [α]_D_ = - 18 (*c* = 0.1, CH_2_Cl_2_, 29.2 °C). **^1^H NMR (300 MHz, CDCl_3_)** *δ* 7.68 (d, *J* = 7.6 Hz, 2H), 7.60 (d, *J* = 7.3 Hz, 2H), 7.53 - 7.29 (m, 10H), 7.26 - 7.12 (m, 5H), 6.92 (s, 1H), 6.28 (d, *J* = 9.0 Hz, 1H), 5.41 (d, *J* = 8.9 Hz, 1H), 5.07 (d, *J* = 13.5 Hz, 1H), 4.50 - 4.36 (m, 2H), 4.10 (d, *J* = 13.2 Hz, 1H) ppm. **^13^C NMR (75 MHz, CDCl_3_)** *δ* 140.6, 139.9, 139.1, 135.9, 135.6, 133.3, 132.9, 132.8, 132.6, 129.6, 128.9, 128.8, 128.6, 128.5, 128.4, 128.2, 127.8, 127.3, 126.5, 75.0, 68.6, 61.3 ppm. **HRMS** (ESI) (m/z) [M+Na]^+^ Calcd for C_31_H_26_BrNO_3_SNa^+^ 594.0709; found 594.0701. **HPLC:** The product was analyzed by HPLC to determine the enantiomeric excess: 94% ee (Chiralpak IG-H, *n*-hexane/*i*-propanol = 95/5, 1 mL/min, 254 nm) t_R_ = 34.267 min, 56.116 min.

**45**

**(*S, Z*)-3-((*Z*)-benzylidene)-8-(4-chlorophenyl)-6-phenyl-5-(phenylsulfonyl)-3, 4, 5, 8-tetrahydro-2*H*-1, 5-oxazocine**

The reaction of **1VI** (19.1 mg, 0.05 mmol) and **2a** (19.8 mg, 0.075 mmol), Cs_2_CO_3_ (32.5 mg, 0.1 mmol), after a flash column chromatography (petroleum ether : AcOEt = 20 : 1), afforded the product **45** (11.9 mg, 45% yield).

**45**. White solid. **MP:** 143 - 144 ºC. [α]_D_ = - 15 (*c* = 0.1, CH_2_Cl_2_, 29.4 °C). **^1^H NMR (300 MHz, CDCl_3_)** *δ* 7.67 (d, *J* = 7.6 Hz, 2H), 7.59 (d, *J* = 7.0 Hz, 2H), 7.53 - 7.46 (m, 1H), 7.43 - 7.30 (m, 7H), 7.25 - 7.12 (m, 6H), 7.06 - 7.01 (m, 1H), 6.90 (s, 1H), 6.29 (d, *J* = 8.9 Hz, 1H), 5.38 (d, *J* = 8.9 Hz, 1H), 5.06 (d, *J* = 13.3 Hz, 1H), 4.48 - 4.34 (m, 2H), 4.12 - 4.07 (m, 1H) ppm. **^13^C NMR (75 MHz, CDCl_3_)** *δ* 164.6, 161.3, 144.0, 143.9, 140.5, 139.1, 135.9, 135.5, 132.7, 132.6, 130.0, 129.5, 128.9, 128.4, 128.4, 128.2, 127.3, 126.5, 122.0, 114.5, 114.3, 113.4, 113.1, 75.0, 68.6, 61.3 ppm. **HRMS** (ESI) (m/z) [M+Na]^+^ Calcd for C_31_H_26_ClNO_3_SNa^+^ 550.1214; found 550.1220. **HPLC:** The product was analyzed by HPLC to determine the enantiomeric excess: 86% ee (Chiralpak IA-H, *n*-hexane/*i*-propanol = 95/5, 1 mL/min, 254 nm) t_R_ = 13.376 min, 16.238 min.

**46**

**(*S, Z*)-3-((*Z*)-benzylidene)-8-(4-bromophenyl)-6-phenyl-5-(phenylsulfonyl)-3, 4, 5, 8-tetrahydro-2*H*-1, 5-oxazocine**

The reaction of **1VII** (21.3 mg, 0.05 mmol) and **2a** (19.8 mg, 0.075 mmol), Cs_2_CO_3_ (32.5 mg, 0.1 mmol), after a flash column chromatography (petroleum ether : AcOEt = 20 : 1), afforded the product **46** (14.3 mg, 50% yield).

**46**. White solid. **MP:** 132 - 133 ºC. [α]_D_ = - 31 (*c* = 0.1, CH_2_Cl_2_, 18.3 °C). **^1^H NMR (300 MHz, CDCl_3_)** *δ* 7.68 (d, *J* = 7.6 Hz, 2H), 7.59 - 7.57 (m, 3H), 7.52 - 7.45 (m, 2H), 7.44 - 7.30 (m, 7H), 7.25 - 7.13 (m, 5H), 6.92 (s, 1H), 6.28 (d, *J* = 9.0 Hz, 1H), 5.32 (d, *J* = 8.9 Hz, 1H), 5.07 (d, *J* = 13.2 Hz, 1H), 4.48 - 4.34 (m, 2H), 4.10 (d, *J* = 13.4 Hz, 1H) ppm. **^13^C NMR (75 MHz, CDCl_3_)** *δ* 143.6, 140.5, 139.2, 135.9, 135.5, 132.7, 132.6, 132.5, 130.7, 130.1, 129.6, 129.3, 128.9, 128.8, 128.5, 128.4, 128.2, 127.3, 126.5, 125.1, 122.6, 74.9, 68.7, 61.4 ppm. **HRMS** (ESI) (m/z) [M+Na]^+^ Calcd for C_31_H_26_BrNO_3_SNa^+^ 594.0709; found 594.0717. **HPLC:** The product was analyzed by HPLC to determine the enantiomeric excess: 92% ee (Chiralpak IG-H, *n*-hexane/*i*-propanol = 95/5, 1 mL/min, 254 nm) t_R_ = 22.710 min, 31.540 min.

**47**

**(*S, Z*)-3-((*Z*)-benzylidene)-8-(naphthalen-1-yl)-6-phenyl-5-(phenylsulfonyl)-3, 4, 5, 8-tetrahydro-2*H*-1, 5-oxazocine**

The reaction of **1VIII** (19.9 mg, 0.05 mmol) and **2a** (19.8 mg, 0.075 mmol), Cs_2_CO_3_ (32.5 mg, 0.1 mmol), after a flash column chromatography (petroleum ether : AcOEt = 20 : 1), afforded the product **47** (14.1 mg, 52% yield).

**47**. White solid. **MP:** 122 - 123 ºC. [α]_D_ = - 21 (*c* = 0.1, CH_2_Cl_2_, 29.2 °C). **^1^H NMR (300 MHz, CDCl_3_)** *δ* 7.96 - 7.85 (m, 4H), 7.72 (d, *J* = 7.6 Hz, 2H), 7.65 - 7.60 (tm, 3H), 7.58 - 7.46 (m, 3H), 7.44 - 7.31 (m, 5H), 7.26 - 7.13 (m, 5H), 6.94 (s, 1H), 6.43 (d, *J* = 9.0 Hz, 1H), 5.52 (d, *J* = 9.0 Hz, 1H), 5.11 (d, *J* = 13.1 Hz, 1H), 4.56 - 4.41 (m, 2H), 4.15 (d, *J* = 13.2 Hz, 1H) ppm. **^13^C NMR (75 MHz, CDCl_3_)** *δ* 140.6, 139.0, 138.7, 136.2, 135.6, 133.3, 133.1, 133.0, 132.6, 129.6, 128.9, 128.7, 128.5, 128.4, 128.3, 128.2, 128.1, 127.7, 127.3, 126.5, 126.1, 125.9, 125.1, 124.6, 75.8, 68.7, 61.4 ppm. **HRMS** (ESI) (m/z) [M+H]^+^ Calcd for C_35_H_30_NO_3_S^+^ 544.1941; found 544.1942. **HPLC:** The product was analyzed by HPLC to determine the enantiomeric excess: 97% ee (Chiralpak IG-H, *n*-hexane/*i*-propanol = 95/5, 1 mL/min, 254 nm) t_R_ = 34.248 min, 53.847 min.

**48**

**(*S, Z*)-3-((*Z*)-benzylidene)-8-(4-fluorophenyl)-5-(phenylsulfonyl)-6-(*m*-tolyl)-3, 4, 5, 8-tetrahydro-2*H*-1, 5-oxazocine**

The reaction of **1IX** (19.0 mg, 0.05 mmol) and **2a** (19.8 mg, 0.075 mmol), Cs_2_CO_3_ (32.5 mg, 0.1 mmol), after a flash column chromatography (petroleum ether : AcOEt = 20 : 1), afforded the product **48** (16.3 mg, 62% yield).

**48**. White solid. **MP:** 132 - 133 ºC. [α]_D_ = - 23 (*c* = 0.1, CH_2_Cl_2_, 28.8 °C). **^19^F NMR (282 MHz, CDCl_3_)** *δ* - 115.52 ppm. **^1^H NMR (300 MHz, CDCl_3_)** *δ* 7.67 (d, *J* = 7.5 Hz, 2H), 7.60 (d, *J* = 7.3 Hz, 2H), 7.54 - 7.45 (m, 3H), 7.41 - 7.31 (m, 5H), 7.16 - 6.97 (m, 5H), 6.89 (d, *J* = 7.3 Hz, 2H), 6.30 (d, *J* = 9.0 Hz, 1H), 5.43 (d, *J* = 8.9 Hz, 1H), 5.05 (d, *J* = 13.5 Hz, 1H), 4.42 (s, 2H), 4.07 (d, *J* = 13.5 Hz, 1H), 2.16 (s, 3H) ppm. **^13^C NMR (75 MHz, CDCl_3_)** *δ* 162.3 (d, *J*_C-F_ = 245.5 Hz), 140.7, 139.0, 137.9, 137.2 (d, *J*_C-F_ = 2.9 Hz), 135.6, 133.0, 132.8, 132.5, 129.6, 128.8, 128.4, 128.3, 128.1, 128.0, 127.3, 127.1, 123.8, 115.3 (d, *J*_C-F_ = 25.4 Hz), 75.1, 68.5, 61.4, 21.3 ppm. **HRMS** (ESI) (m/z) [M+H]^+^ Calcd for C_32_H_29_FNO_3_S^+^ 526.1847; found 526.1841. **HPLC:** The product was analyzed by HPLC to determine the enantiomeric excess: 90% ee (Chiralpak IA-H, *n*-hexane/*i*-propanol = 95/5, 1 mL/min, 254 nm) t_R_ = 14.843 min, 16.650 min.

**49**

**(*S, Z*)-3-((*Z*)-benzylidene)-8-(4-chlorophenyl)-5-(phenylsulfonyl)-6-(*m*-tolyl)-3, 4, 5, 8-tetrahydro-2*H*-1, 5-oxazocine**

The reaction of **1X** (19.8 mg, 0.05 mmol) and **2a** (19.8 mg, 0.075 mmol), Cs_2_CO_3_ (32.5 mg, 0.1 mmol), after a flash column chromatography (petroleum ether : AcOEt = 20 : 1), afforded the product **49** (14.9 mg, 55% yield). (**Condition B** : 83% yield, 95% ee)

**49**. White solid. **MP:** 136 – 137 ºC. [α]_D_ = - 20 (*c* = 0.1, CH_2_Cl_2_, 20.3 °C). **^1^H NMR (300 MHz, CDCl_3_)** *δ* 7.67 (d, *J* = 7.6 Hz, 2H), 7.60 (d, *J* = 7.1 Hz, 2H), 7.53 - 7.30 (m, 10H), 7.10 - 6.96 (m, 3H), 6.89 (d, *J* = 11.7 Hz, 2H), 6.27 (d, *J* = 9.0 Hz, 1H), 5.44 (d, *J* = 8.8 Hz, 1H), 5.05 (d, *J* = 13.5 Hz, 1H), 4.43 (s, 2H), 4.06 (d, *J* = 13.3 Hz, 1H), 2.16 (s, 3H) ppm. **^13^C NMR (75 MHz, CDCl_3_)** *δ* 140.6, 140.0, 139.1, 137.9, 135.6, 133.3, 132.9, 132.5, 129.6, 128.8, 128.6, 128.4, 128.3, 128.2, 127.8, 127.3, 127.1, 123.8, 75.0, 68.5, 61.3, 21.3 ppm. **HRMS** (ESI) (m/z) [M+H]^+^ Calcd for C_32_H_29_ClNO_3_S^+^ 542.1551; found 542.1547. **HPLC:** The product was analyzed by HPLC to determine the enantiomeric excess: 91% ee (Chiralpak IA-H, *n*-hexane/*i*-propanol = 95/5, 1 mL/min, 254 nm) t_R_ = 15.150 min, 18.326 min.

**50**

**(*S, Z*)-3-((*Z*)-benzylidene)-6-(4-bromophenyl)-8-(4-chlorophenyl)-5-(phenylsulfonyl)-3, 4, 5, 8-tetrahydro-2*H*-1, 5-oxazocine**

The reaction of **1XI** (23.0 mg, 0.05 mmol) and **2a** (19.8 mg, 0.075 mmol), Cs_2_CO_3_ (32.5 mg, 0.1 mmol), after a flash column chromatography (petroleum ether : AcOEt = 20 : 1), afforded the product **50** (17.0 mg, 56% yield).

**50**. Yellow oil. [α]_D_ = - 10 (*c* = 0.1, CH_2_Cl_2_, 27.8 °C). **^1^H NMR (300 MHz, CDCl_3_)** *δ* 7.69 (d, *J* = 7.6 Hz, 2H), 7.60 - 7.50 (m, 3H), 7.43 - 7.29 (m, 11H), 7.05 (d, *J* = 8.5 Hz, 2H), 6.89 (s, 1H), 6.27 (d, *J* = 8.9 Hz, 1H), 5.32 (d, *J* = 8.9 Hz, 1H), 5.04 (d, *J* = 12.8 Hz, 1H), 4.45 - 4.32 (m, 2H), 4.05 (d, *J* = 13.0 Hz, 1H) ppm. **^13^C NMR (75 MHz, CDCl_3_)** *δ* 140.4, 139.6, 139.3, 135.4, 135.0, 133.5, 133.3, 132.8, 132.6, 131.5, 129.6, 129.0, 128.7, 128.5, 128.3, 128.0, 127.7, 127.7, 127.2, 123.0, 74.9, 68.7, 61.3 ppm. **HRMS** (ESI) (m/z) [M+H]^+^ Calcd for C_31_H_26_ClBrNO_3_S^+^ 606.0500; found 606.0494. **HPLC:** The product was analyzed by HPLC to determine the enantiomeric excess: 95% ee (Chiralpak IG-H, *n*-hexane/*i*-propanol = 90/10, 1 mL/min, 254 nm) t_R_ = 22.387 min, 40.280 min.

**51**

**(*S, Z*)-3-((*Z*)-benzylidene)-8-(4-bromophenyl)-5-(phenylsulfonyl)-6-(*m*-tolyl)-3, 4, 5, 8-tetrahydro-2*H*-1, 5-oxazocine**

The reaction of **1XII** (22.0 mg, 0.05 mmol) and **2a** (19.8 mg, 0.075 mmol), Cs_2_CO_3_ (32.5 mg, 0.1 mmol), after a flash column chromatography (petroleum ether : AcOEt = 20 : 1), afforded the product **51** (18.1 mg, 66% yield).

**51**. White solid. **MP:** 122 - 123 ºC. [α]_D_ = - 53 (*c* = 0.1, CH_2_Cl_2_, 30.2 °C). **^1^H NMR (300 MHz, CDCl_3_)** *δ* 7.67 (d, *J* = 7.6 Hz, 2H), 7.60 (d, *J* = 7.2 Hz, 2H), 7.54 - 7.44 (m, 3H), 7.43 - 7.31 (m, 7H), 7.10 - 6.96 (m, 3H), 6.91 (s, 1H), 6.87 (s, 1H), 6.27 (d, *J* = 9.0 Hz, 1H), 5.45 (d, *J* = 9.0 Hz, 1H), 5.06 (d, *J* = 13.3 Hz, 1H), 4.43 (s, 2H), 4.06 (d, *J* = 13.2 Hz, 1H), 2.16 (s, 3H) ppm. **^13^C NMR (75 MHz, CDCl_3_)** *δ* 140.6, 140.0, 139.0, 137.9, 135.6, 135.5, 133.3, 132.9, 132.5, 129.6, 128.8, 128.6, 128.4, 128.3, 128.2, 127.8, 127.3, 127.1, 123.8, 75.0, 68.5, 61.3, 21.3 ppm. **HRMS** (ESI) (m/z) [M+Na]^+^ Calcd for C_32_H_28_BrNO_3_SNa^+^ 608.0865; found 608.0898. **HPLC:** The product was analyzed by HPLC to determine the enantiomeric excess: 90% ee (Chiralpak IA-H, *n*-hexane/*i*-propanol = 95/5, 1 mL/min, 254 nm) t_R_ = 15.001 min, 18.143 min.

**52**

**(*S, Z*)-3-((*Z*)-benzylidene)-8-(4-chlorophenyl)-6-(4-fluorophenyl)-5-(phenylsulfonyl)-3, 4, 5, 8-tetrahydro-2*H*-1, 5-oxazocine**

The reaction of **1XIII** (20.8 mg, 0.05 mmol) and **2a** (19.8 mg, 0.075 mmol), Cs_2_CO_3_ (32.5 mg, 0.1 mmol), after a flash column chromatography (petroleum ether : AcOEt = 20 : 1), afforded the product **52** (16.4 mg, 60% yield). (**Condition B** : 81% yield, 79% ee)

**52**. Yellow oil. [α]_D_ = - 11 (*c* = 0.1, CH_2_Cl_2_, 28.8 °C). **^19^F NMR (282 MHz, CDCl_3_)** *δ* - 112.78 ppm. **^1^H NMR (300 MHz, CDCl_3_)** *δ* 7.69 - 7.64 (m, 2H), 7.58 (d, *J* = 7.2 Hz, 3H), 7.44 - 7.32 (m, 9H), 7.19 - 7.08 (m, 4H), 6.89 (s, 1H), 6.26 (d, *J* = 8.9 Hz, 1H), 5.33 (d, *J* = 8.9 Hz, 1H), 5.04 (d, *J* = 13.1 Hz, 1H), 4.48 - 4.33 (m, 2H), 4.05 (d, *J* = 12.9 Hz, 1H) ppm. **^13^C NMR (75 MHz, CDCl_3_)** *δ* 162.9 (d, *J*_C–F_ = 249.4 Hz), 140.5, 139.8, 139.2, 135.5, 133.4, 132.7, 132.6, 129.6, 128.9, 128.6, 128.5, 128.4, 128.29, 128.27, 127.7, 127.2, 115.4 (d, *J*_C–F_ = 21.7 Hz), 75.0, 68.6, 61.3 ppm. **HRMS** (ESI) (m/z) [M+H]^+^ Calcd for C_31_H_26_Cl_2_NO_3_S^+^ 562.1005; found 562.1006. **HPLC:** The product was analyzed by HPLC to determine the enantiomeric excess: 91% ee (Chiralpak IG-H, *n*-hexane/*i*-propanol = 95/5, 1 mL/min, 254 nm) t_R_ = 31.635 min, 47.033 min.

**53**

**(*S, Z*)-3-((*Z*)-benzylidene)-8-(4-chlorophenyl)-5-(phenylsulfonyl)-6-(*p*-tolyl)-3, 4, 5, 8-tetrahydro-2*H*-1, 5-oxazocine**

The reaction of **1XIV** (19.8 mg, 0.05 mmol) and **2a** (19.8 mg, 0.075 mmol), Cs_2_CO_3_ (32.5 mg, 0.1 mmol), after a flash column chromatography (petroleum ether : AcOEt = 20 : 1), afforded the product **53** (14.1 mg, 52% yield).

**53**. White solid. **MP:** 146 – 147 ºC. [α]_D_ = - 17 (*c* = 0.1, CH_2_Cl_2_, 30.5 °C). **^1^H NMR (300 MHz, CDCl_3_)** *δ* 7.68 (d, *J* = 7.4 Hz, 2H), 7.58 (d, *J* = 7.2 Hz, 2H), 7.50 (t, *J* = 7.4 Hz, 1H), 7.44 - 7.31 (m, 9H), 7.08 (d, *J* = 8.2 Hz, 2H), 6.97 (d, *J* = 8.1 Hz, 2H), 6.88 (s, 1H), 6.23 (d, *J* = 9.0 Hz, 1H), 5.33 (d, *J* = 9.0 Hz, 1H), 5.03 (d, *J* = 13.4 Hz, 1H), 4.39 (s, 2H), 4.06 (d, *J* = 13.6 Hz, 1H), 2.31 (s, 3H) ppm. **^13^C NMR (75 MHz, CDCl_3_)** *δ* 140.6, 140.0, 139.0, 138.9, 135.6, 133.3, 132.9, 132.5, 131.6, 129.5, 129.1, 128.8, 128.6, 128.4, 128.2, 127.7, 127.3, 126.4, 74.9, 68.5, 61.2, 21.2 ppm. **HRMS** (ESI) (m/z) [M+H]^+^ Calcd for C_32_H_29_ClNO_3_S^+^ 542.1551; found 542.1553. **HPLC:** The product was analyzed by HPLC to determine the enantiomeric excess: 90% ee (Chiralpak IA-H, *n*-hexane/*i*-propanol = 95/5, 1 mL/min, 254 nm) t_R_ = 22.589 min, 28.587 min.

**54**

**(*S, Z*)-3-((*Z*)-benzylidene)-6, 8-bis(4-chlorophenyl)-5-(phenylsulfonyl)-3, 4, 5, 8-tetrahydro-2*H*-1, 5-oxazocine**

The reaction of **1XV** (19.8 mg, 0.05 mmol) and **2a** (19.8 mg, 0.075 mmol), Cs_2_CO_3_ (32.5 mg, 0.1 mmol), after a flash column chromatography (petroleum ether : AcOEt = 20 : 1), afforded the product **54** (12.3 mg, 44% yield). (**Condition B** : 83% yield, 75% ee)

**54**. Yellow oil. [α]_D_ = + 19 (*c* = 0.1, CH_2_Cl_2_, 28.6 °C). **^1^H NMR (300 MHz, CDCl_3_)** *δ* 7.68 (d, *J* = 7.4 Hz, 2H), 7.60 - 7.50 (m, 1H), 7.44 - 7.31 (m, 9H), 7.16 - 7.09 (m, 4H), 6.89 (s, 1H), 6.23 (d, *J* = 9.0 Hz, 1H), 5.33 (d, *J* = 9.0 Hz, 1H), 5.03 (d, *J* = 13.4 Hz, 1H), 4.40 (dd, *J* = 15.0, 12.3 Hz, 2H), 4.06 (d, *J* = 13.6 Hz, 1H) ppm. **^13^C NMR (75 MHz, CDCl_3_)** *δ* 140.4, 139.6, 139.3, 135.4, 134.7, 134.5, 133.4, 133.2, 132.8, 132.6, 129.6, 129.0, 128.7, 128.6, 128.5, 128.3, 127.74, 127.72, 127.2, 75.0, 68.7, 61.3 ppm. **HRMS** (ESI) (m/z) [M+H]^+^ Calcd for C_32_H_29_ClNO_3_S^+^ 542.1551; found 542.1553. **HPLC:** The product was analyzed by HPLC to determine the enantiomeric excess: 76% ee (Chiralpak IG-H, *n*-hexane/*i*-propanol = 95/5, 1 mL/min, 254 nm) t_R_ = 34.355 min, 60.999 min.

**55**

**(*S, Z*)-3-((*Z*)-benzylidene)-6-(3, 5-dimethylphenyl)-8-(4-fluorophenyl)-5-(phenylsulfonyl)-3, 4, 5, 8-tetrahydro-2*H*-1, 5-oxazocine**

The reaction of **1XVI** (19.7 mg, 0.05 mmol) and **2a** (19.8 mg, 0.075 mmol), Cs_2_CO_3_ (32.5 mg, 0.1 mmol), after a flash column chromatography (petroleum ether : AcOEt = 20 : 1), afforded the product **55** (17.0 mg, 63% yield).

**55**. Yellow oil. [α]_D_ = + 5 (*c* = 0.1, CH_2_Cl_2_, 29.3 °C). **^19^F NMR (282 MHz, CDCl_3_)** *δ* - 115.59 ppm. **^1^H NMR (300 MHz, CDCl_3_)** *δ* 7.69 (d, *J* = 7.8 Hz, 2H), 7.62 (d, *J* = 7.7 Hz, 2H), 7.56 - 7.49 (m, 3H), 7.44 - 7.31 (m, 5H), 7.17 - 7.10 (m, 2H), 6.92 (s, 1H), 6.87 (s, 1H), 6.72 (s, 2H), 6.31 (d, *J* = 9.0 Hz, 1H), 5.50 (d, *J* = 8.8 Hz, 1H), 5.06 (d, *J* = 13.1 Hz, 1H), 4.45 (s, 2H), 4.06 (d, *J* = 13.5 Hz, 1H), 2.14 (s, 6H) ppm. **^13^C NMR (75 MHz, CDCl_3_)** *δ* 162.3 (d, *J*_C-F_ = 245.5 Hz), 143.4, 140.8, 138.9, 137.9, 137.4 (d, *J*_C-F_ = 3.0 Hz), 135.7, 135.3, 134.8, 133.1, 132.53, 132.46, 130.5, 129.6, 129.0, 128.7, 128.4, 128.2, 128.12, 128.05, 127.3, 125.4, 124.4, 115.3 (d, *J*_C-F_ = 21.4 Hz), 75.2, 68.5, 61.3, 21.2 ppm. **HRMS** (ESI) (m/z) [M+H]^+^ Calcd for C_33_H_31_FNO_3_S^+^ 540.2003; found 540.2006. **HPLC:** The product was analyzed by HPLC to determine the enantiomeric excess: 90% ee (Chiralpak IA-H, *n*-hexane/*i*-propanol = 95/5, 1 mL/min, 254 nm) t_R_ = 10.526 min, 13.476 min.

**56**

**(*S, Z*)-3-((*Z*)-benzylidene)-8-(4-bromophenyl)-6-phenyl-5-tosyl-3, 4, 5, 8-tetrahydro-2*H*-1, 5-oxazocine**

The reaction of **1XVII** (22.0 mg, 0.05 mmol) and **2a** (19.8 mg, 0.075 mmol), Cs_2_CO_3_ (32.5 mg, 0.1 mmol), after a flash column chromatography (petroleum ether : AcOEt = 20 : 1), afforded the product **56** (19.9 mg, 68% yield).

**56**. White solid. **MP:** 142 - 143 ºC. [α]_D_ = - 34 (*c* = 0.1, CH_2_Cl_2_, 17.8 °C). **^1^H NMR (300 MHz, CDCl_3_)** *δ* 7.61 (d, *J* = 8.0 Hz, 4H), 7.49 - 7.31 (m, 10H), 7.16 (t, *J* = 9.2 Hz, 4H), 6.87 (s, 1H), 6.37 (d, *J* = 8.9 Hz, 1H), 5.25 (d, *J* = 8.9 Hz, 1H), 5.02 (d, *J* = 13.1 Hz, 1H), 4.39 (q, *J* = 12.2 Hz, 2H), 4.08 (d, *J* = 13.3 Hz, 1H), 2.39 (s, 3H) ppm. **^13^C NMR (75 MHz, CDCl_3_)** *δ* 143.7, 141.0, 139.7, 138.9, 137.5, 135.6, 135.5, 133.6, 132.0, 131.5 129.6, 128.5, 128.5, 128.2, 128.1, 127.8, 127.3, 126.4, 122.8, 75.6, 68.8, 61.3, 21.5 ppm. **HRMS** (ESI) (m/z) [M+Na]^+^ Calcd for C_32_H_28_BrNO_3_SNa^+^ 608.0865; found 608.0870. **HPLC:** The product was analyzed by HPLC to determine the enantiomeric excess: 91% ee (Chiralpak IA-H, *n*-hexane/*i*-propanol = 95/5, 1 mL/min, 254 nm) t_R_ = 21.489 min, 26.833 min.

Ligand **MQ Phos-1** (4.3 mg, 0.005 mmol, 10 mol %) and Pd_2_(dba)_3_ (2.3 mg, 0.0025 mmol, 5 mol %) were dissolved in toluene (1.0 mL) in a 10 mL Schlenk tube under N_2_. After stirring at room temperature for 40 minutes, compound **1** (17.4 mg, 0.05 mmol)‚ **57** (14.3 mg, 0.075 mmol), Cs_2_CO_3_ (32.5 mg, 0.1 mmol, 2.0 equiv) were added. The reaction mixture was stirred at 40 °C until the substrate was consumed (monitored by TLC), and then was purified by flash column chromatrography (petroleum ether : AcOEt = 20 : 1), afford the corresponding product **3** (70% yield, 92% ee).

Ligand **MQ Phos-1** (4.3 mg, 0.005 mmol, 10 mol %) and Pd_2_(dba)_3_ (2.3 mg, 0.0025 mmol, 5 mol %) were dissolved in toluene (1.0 mL) in a 10 mL Schlenk tube under N_2_. After stirring at room temperature for 40 minutes, compound **1** (17.4 mg, 0.05 mmol)‚ **58** (19.8 mg, 0.075 mmol), Cs_2_CO_3_ (32.5 mg, 0.1 mmol, 2.0 equiv) were added. The reaction mixture was stirred at 40 °C until the substrate was consumed (monitored by TLC), and then was purified by flash column chromatrography (petroleum ether : AcOEt = 20 : 1), afford the corresponding product **3** (54% yield, 91% ee).

Dissolved **3** (49.3 mg, 0.1 mmol) and NMO (23.5 mg, 0.2 mmol) in acetone (1mL), add K_2_OsO_4_•2H_2_O (4 mg, 10 mol%), stired at room temperature for 12 hours, monitored the completion of the reaction by TLC, quenched the reaction with saturated Na_2_SO_3_ aqueous solution, and extracted by DCM, The combined organic layers were dried over MgSO4, filtered, concentrated, and purified by flash chromatography (petroleum ether : AcOEt = 10 : 1) afforded the product **59** (34.1 mg).

**59**

**(*Z*)-7-benzylidene-2, 4-diphenyl-5-(phenylsulfonyl)-1, 5-oxazocan-3-one**

**59.** Yellow oil. **^1^H NMR (600 MHz, CDCl_3_)** *δ* 8.28 (d, *J* = 7.6 Hz, 2H), 7.67 (d, *J* = 7.3 Hz, 2H), 7.57 (t, *J* = 7.4 Hz, 1H), 7.51 - 7.40 (m, 7H), 7.39 - 7.35 (m, 1H), 7.30 - 7.26 (m, 2H), 7.24 - 7.20 (m, 1H), 7.10 (t, *J* = 7.7 Hz, 2H), 7.06 (d, *J* = 7.6 Hz, 2H), 6.32 (s, 1H), 5.49 (d, *J* = 4.1 Hz, 1H), 5.09 (s, 1H), 4.54 - 4.38 (m, 2H), 4.30 - 4.20 (m, 1H), 4.19 - 4.09 (m, 1H) ppm. **^13^C NMR (151 MHz, CDCl_3_)** *δ* 200.9, 135.1, 134.0, 133.4, 132.8, 130.6, 128.7, 128.7, 128.6, 128.4, 128.3, 128.3, 127.9, 127.5, 126.8, 81.0, 74.7, 58.8 ppm. **HRMS** (ESI) (m/z) [M+NH_4_]^+^ Calcd for C_31_H_31_N_2_O_4_S^+^ 527.1999; found 527.2010. **HPLC:** The product was analyzed by HPLC to determine the enantiomeric excess: 90% ee (Chiralpak IG-H, *n*-hexane/*i*-propanol = 90/10, 1 mL/min, 254 nm) t_R_ = 39.038 min, 40.954 min.

Added **3** (49.3 mg, 0.1 mmol), PPh_3_ (1.5 mg, 5 mol%), NBS (18.7 mg, 0.11 mmol) to a 10 mL schlank tube, and exchanged nitrogen gas three times. Under nitrogen protection, added DCM (0.3 mL), SOCl_2_ (4 *μ*L, 0.6 mmol), and stirred at room temperature for 5 hours. The reaction was monitored by TLC, and purified by flash chromatography (petroleum ether : AcOEt = 15 : 1) afforded the product **60** (40.1 mg).

**60**

**(*Z*)-7-benzylidene-4-bromo-3-chloro-2, 4-diphenyl-5-(phenylsulfonyl)-1, 5-oxazocane**

**60.** Yellow oil. **^1^H NMR (600 MHz, CDCl_3_)** *δ* 7.67 - 7.59 (m, 4H), 7.47 - 7.37 (m, 5H), 7.36 - 7.30 (m, 4H), 7.23 - 7.17 (m, 5H), 7.17 - 7.11 (m, 2H), 7.01 (s, 1H), 6.23 (s, 1H), 4.92 (d, *J* = 12.9 Hz, 1H), 4.55 - 4.46 (m, 2H), 4.06 (d, *J* = 12.8 Hz, 1H) ppm. **^13^C NMR (151 MHz, CDCl_3_)** *δ* 140.6, 140.4, 139.5, 138.9, 135.9, 135.5, 134.6, 132.3, 132.0, 129.7, 129.4, 128.9, 128.7, 128.5, 128.4, 128.1, 128.1, 127.5, 127.0, 126.4, 78.5, 69.2, 62.0 ppm. **HRMS** (ESI) (m/z) [M+Na]^+^ Calcd for C_31_H_27_BrClNO_3_SNa^+^ 630.0476; found 630.0467. **HPLC:** The product was analyzed by HPLC to determine the enantiomeric excess: 86% ee (Chiralpak IG-H, *n*-hexane/*i*-propanol = 95/5, 1 mL/min, 254 nm) t_R_ = 12.828 min, 18.832 min.

**5. Copies of ^31^P NMR, ^19^F NMR, ^1^H NMR, ^13^C NMR spectra**


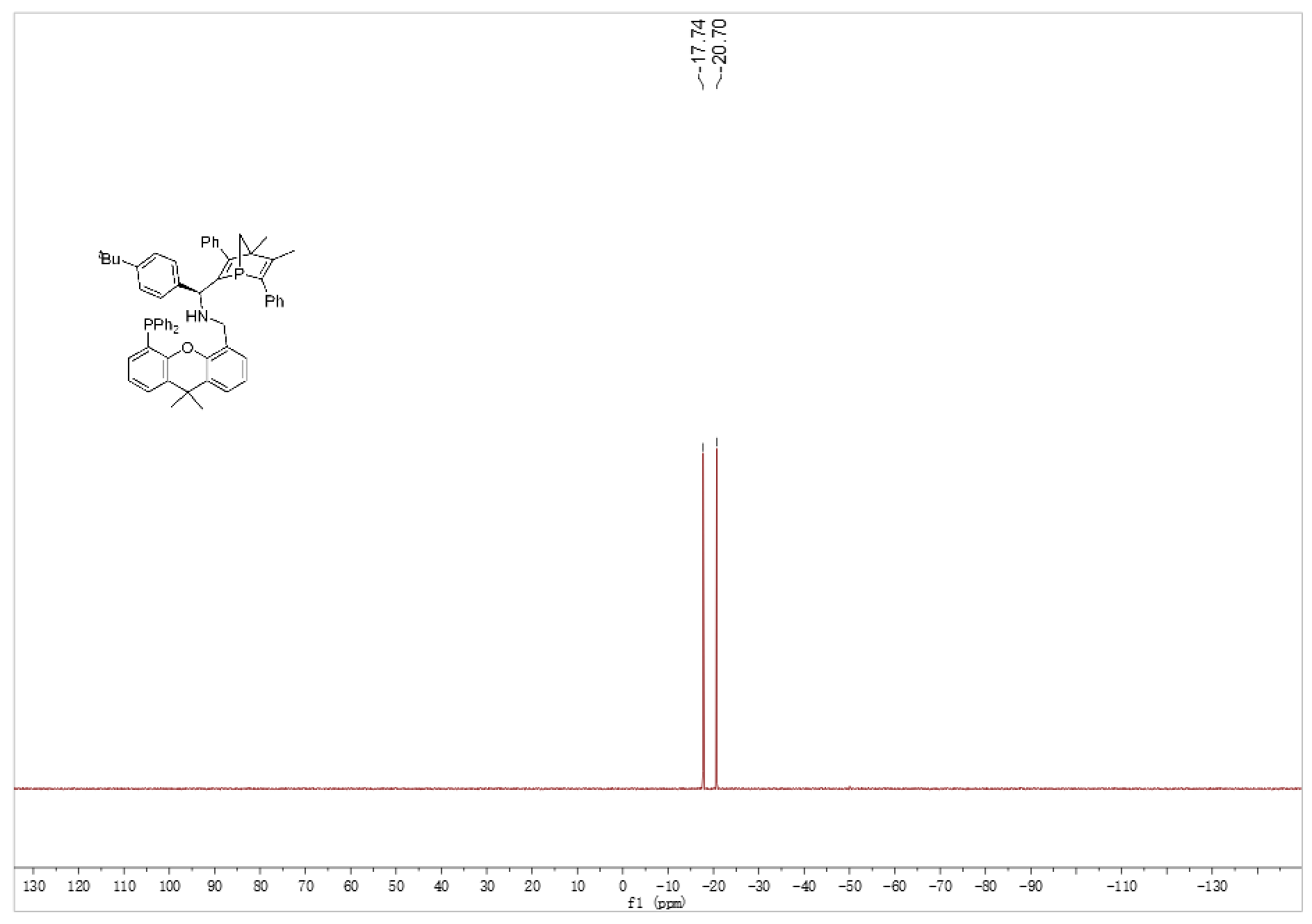


^31^P (CDCl_3_, 162 MHz) NMR of compound **MQ Phos-1**


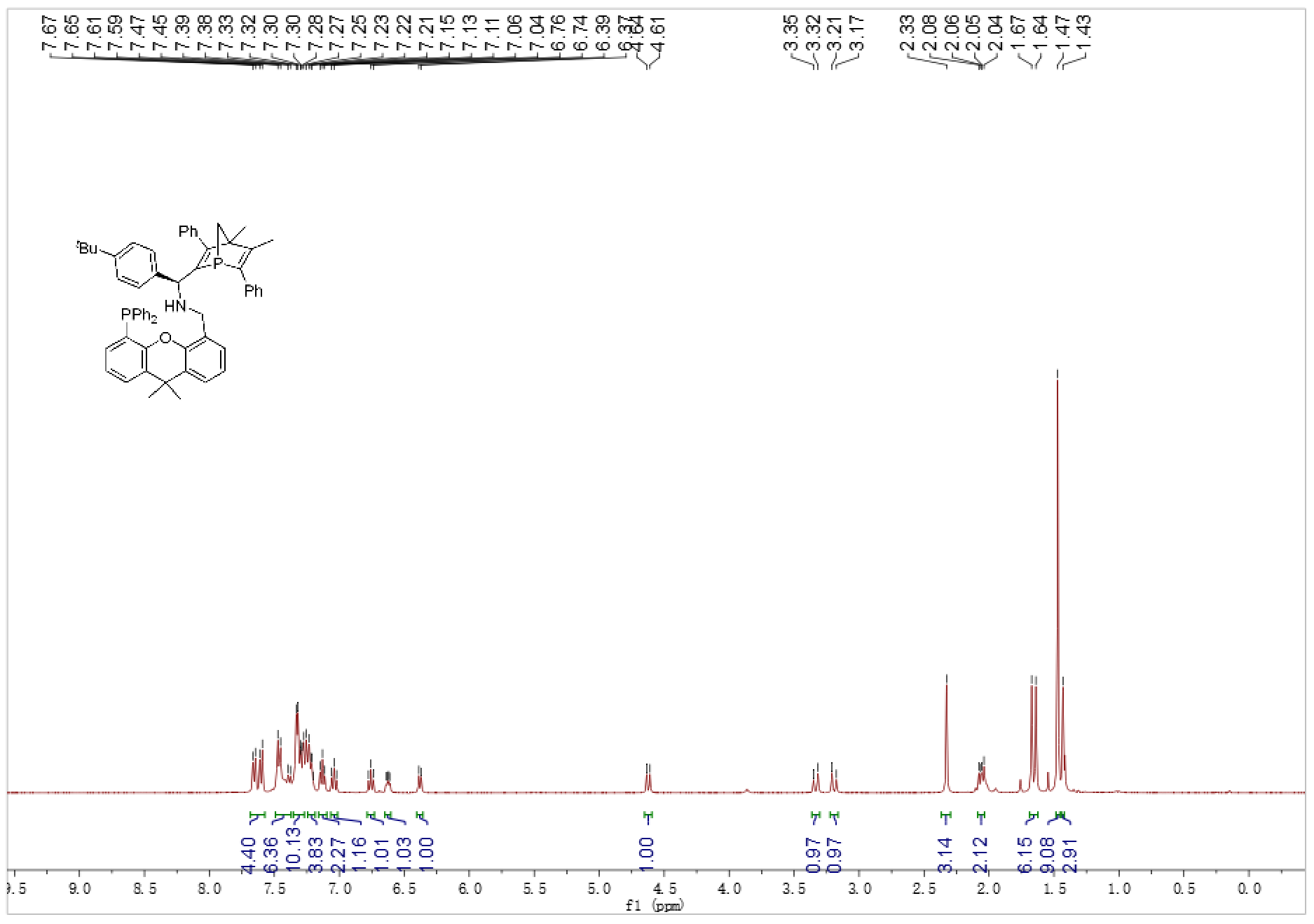


^1^H (CDCl_3_, 400 MHz) NMR of compound **MQ Phos-1**


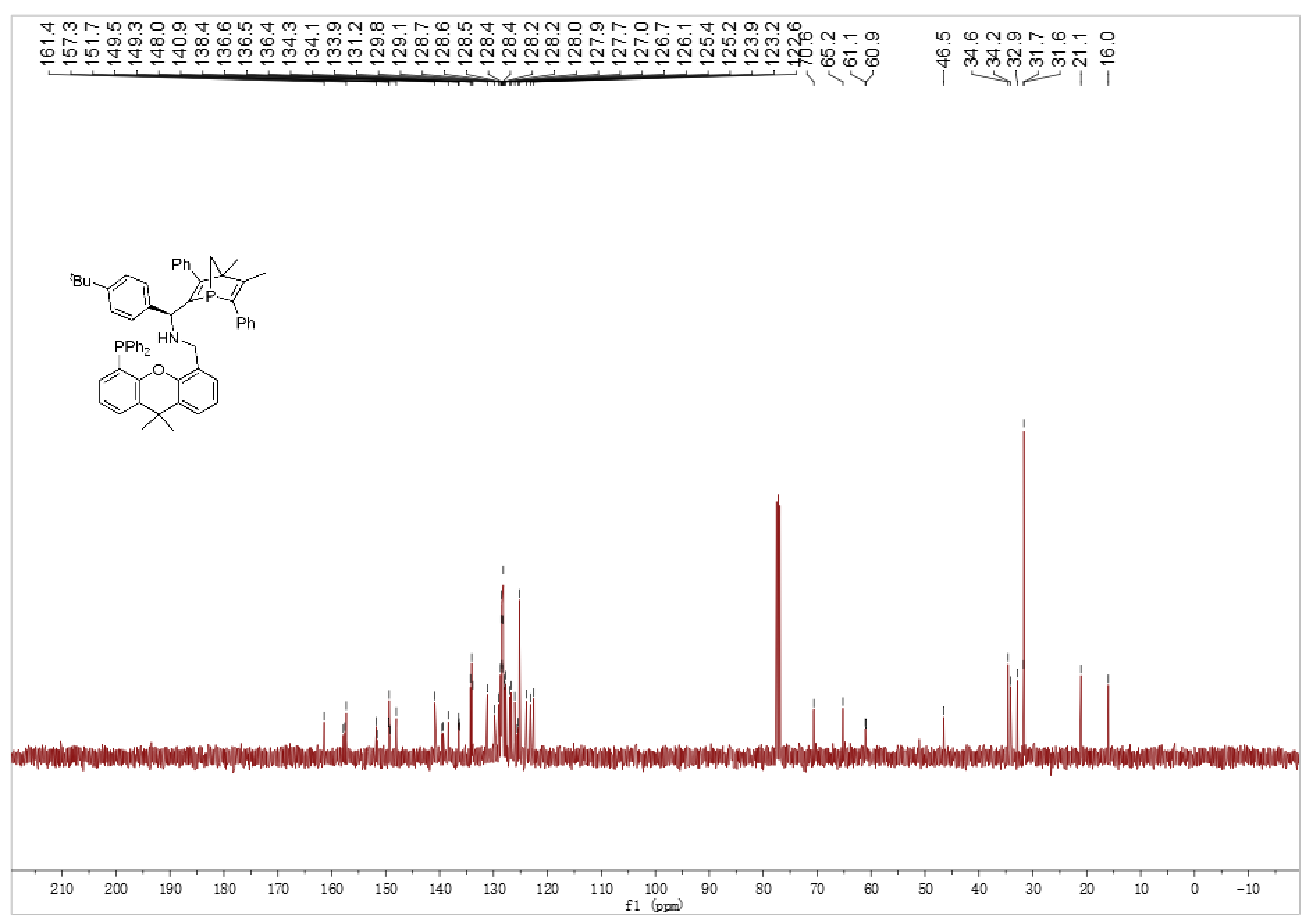


^13^C (CDCl_3_, 101 MHz) NMR of compound **MQ Phos-1**


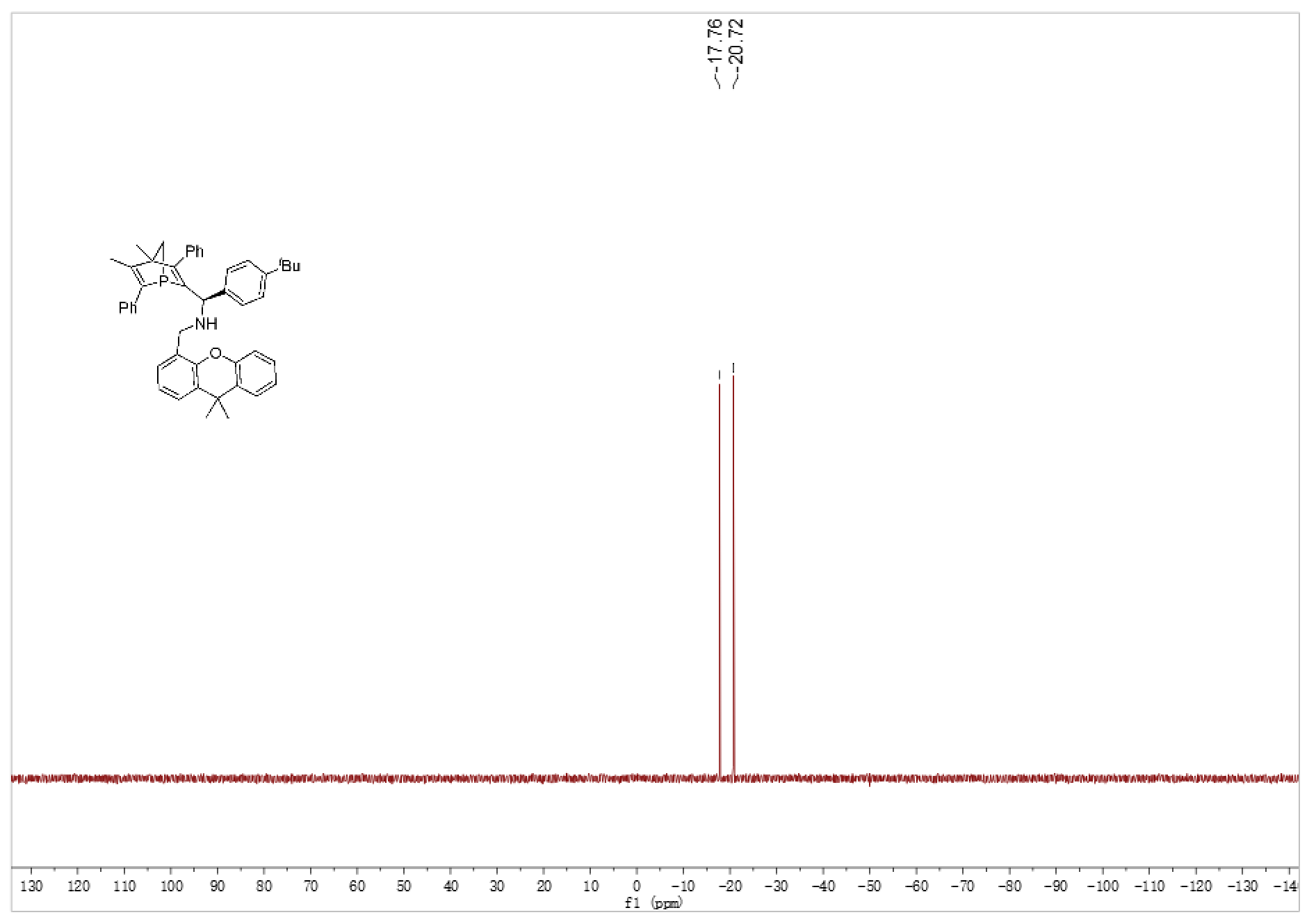


^31^P (CDCl_3_, 162 MHz) NMR of compound **MQ Phos-1^*^**

**
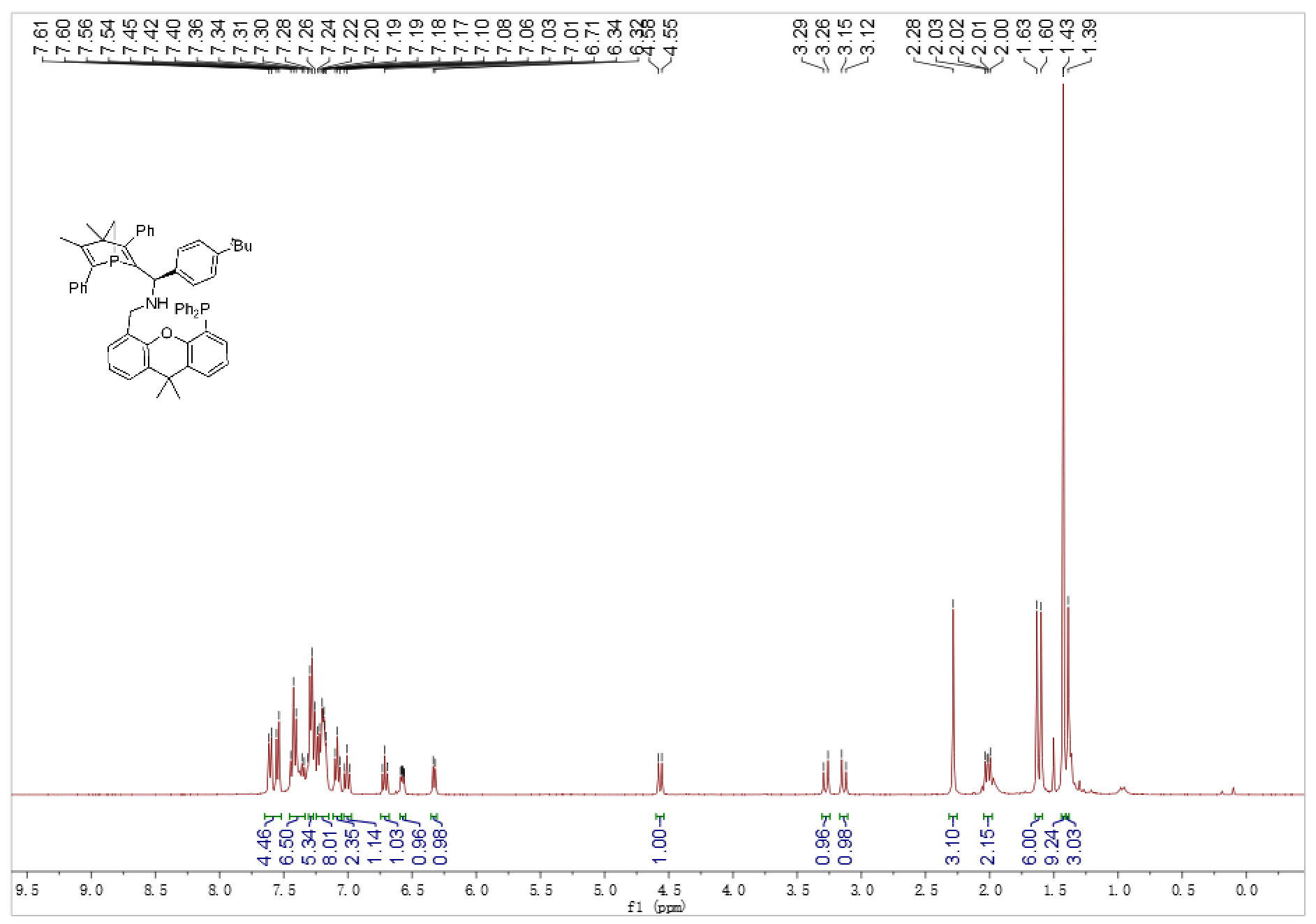
**

^1^H (CDCl_3_, 400 MHz) NMR of compound **MQ Phos-1^*^**


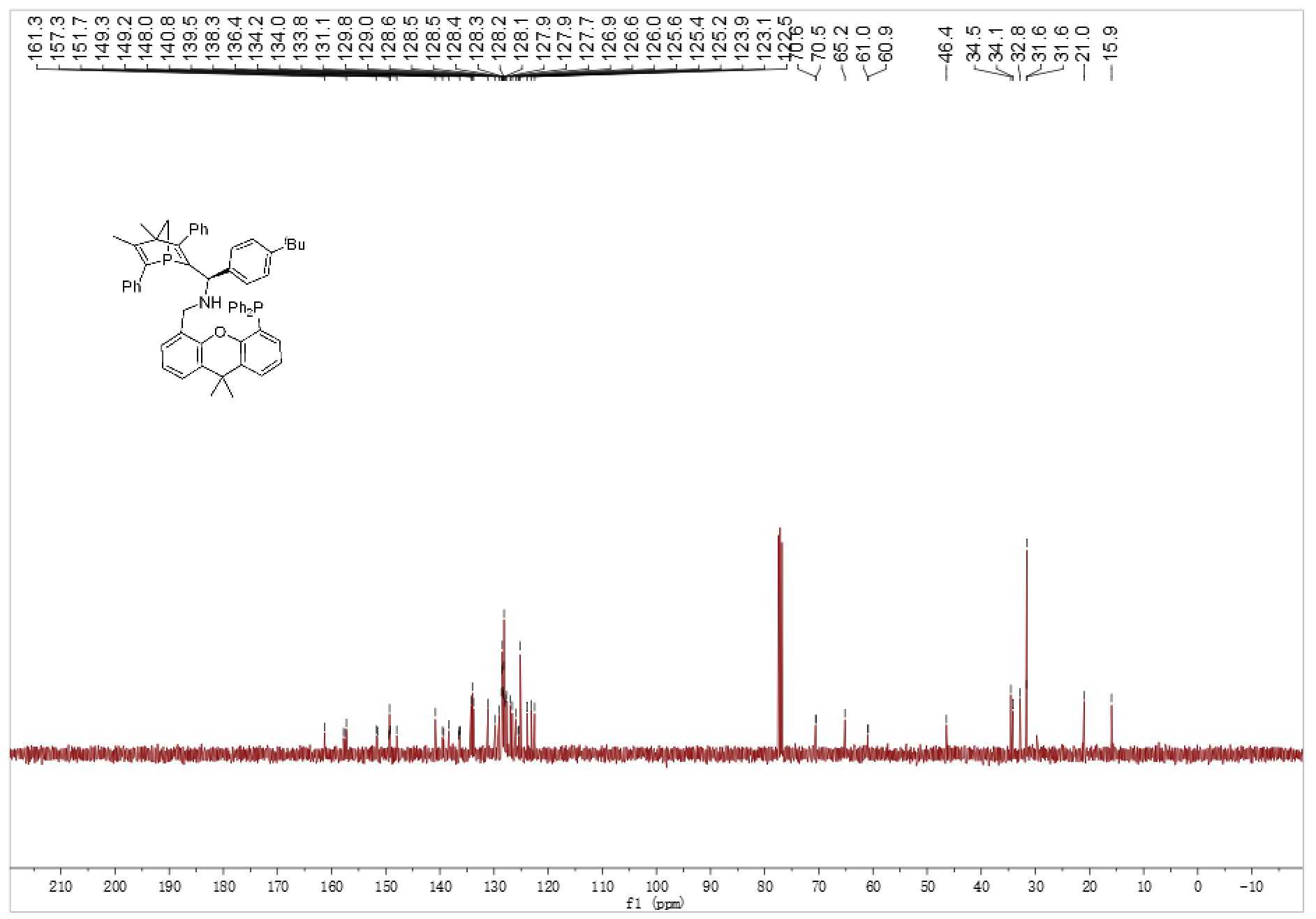


^13^C (CDCl_3_, 101 MHz) NMR of compound **MQ Phos-1^*^**

**
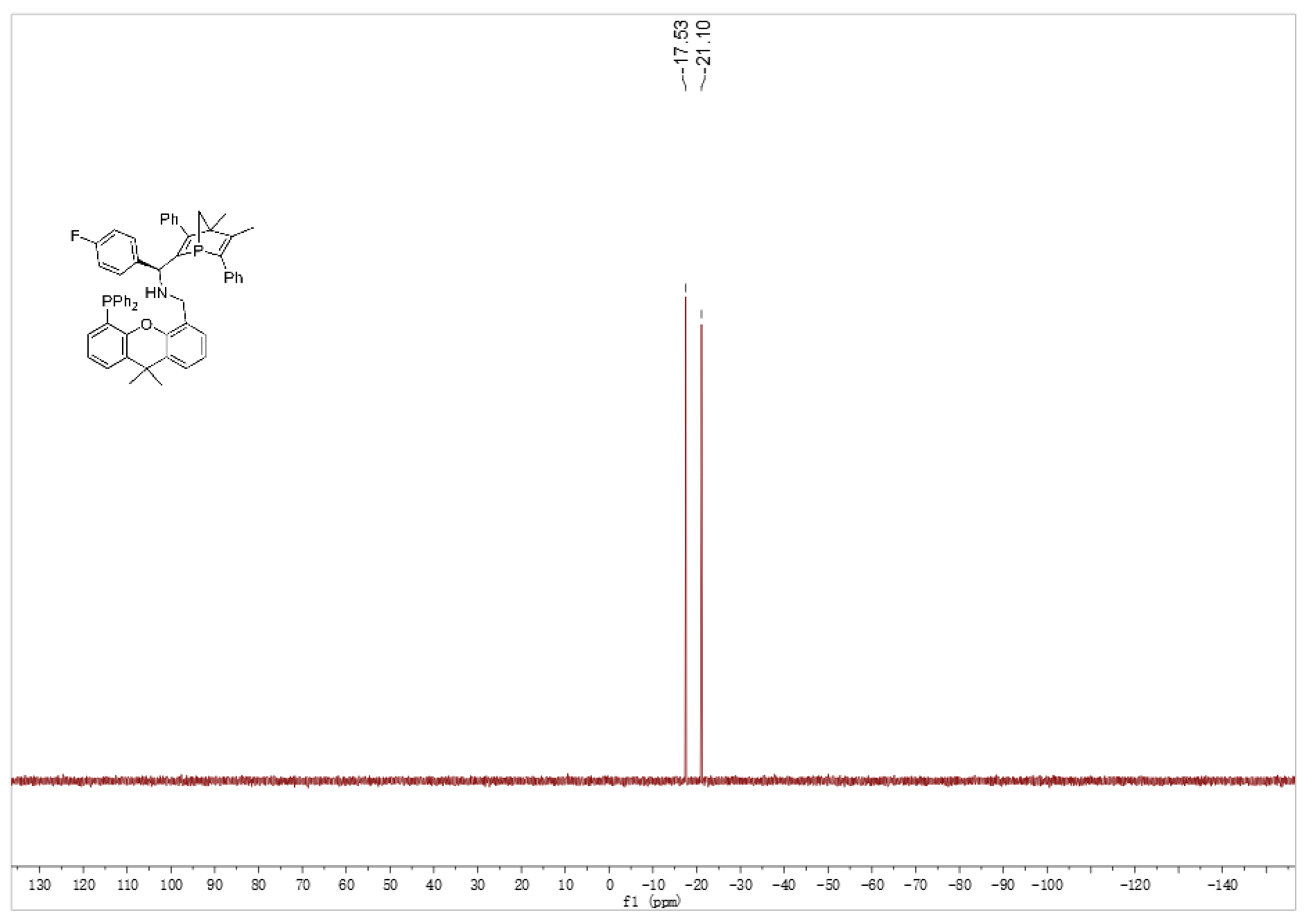
**

^31^P (CDCl_3_, 162 MHz) NMR of compound **MQ Phos-2**

**
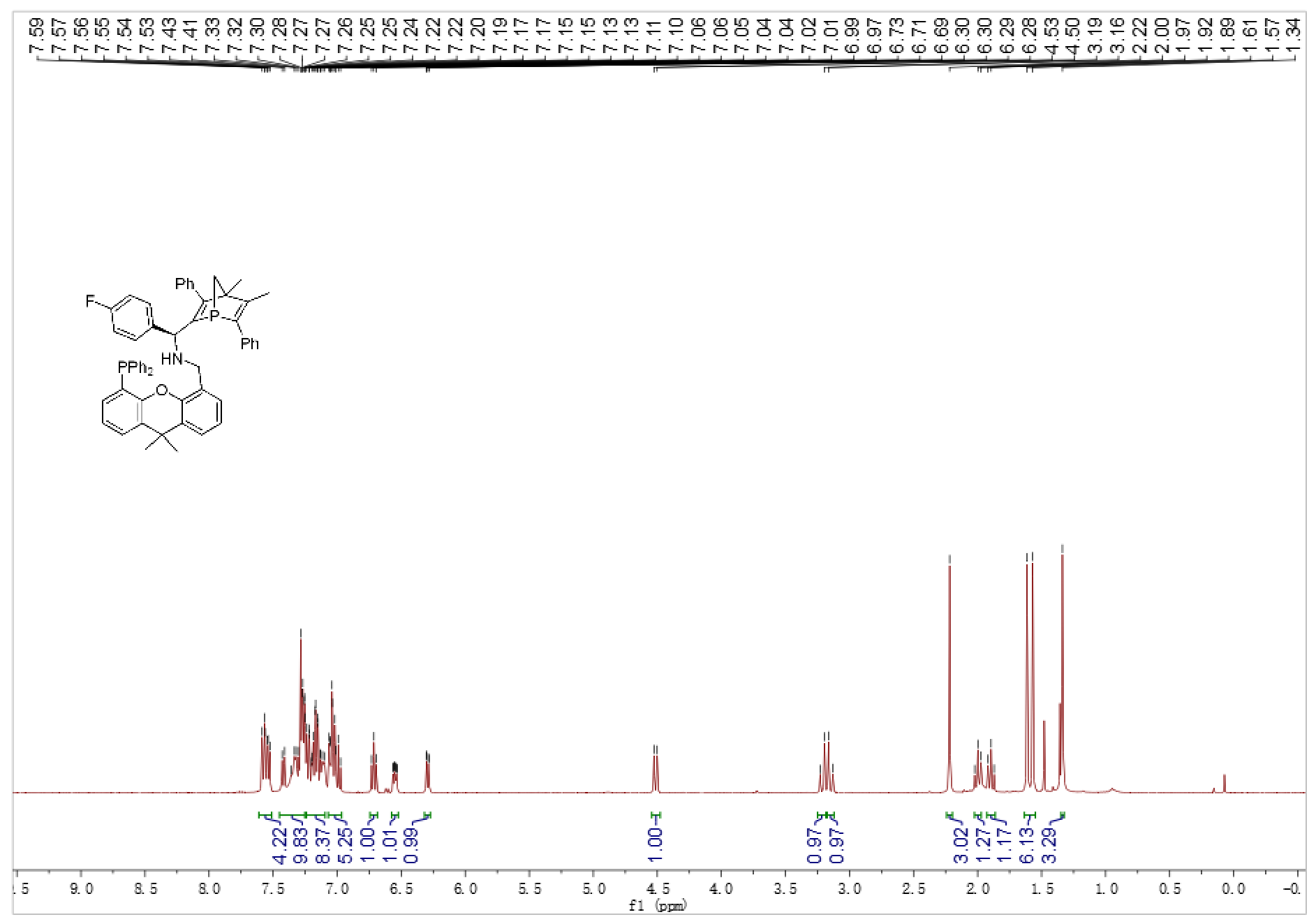
**

^1^H (CDCl_3_, 400 MHz) NMR of compound **MQ Phos-2**

**
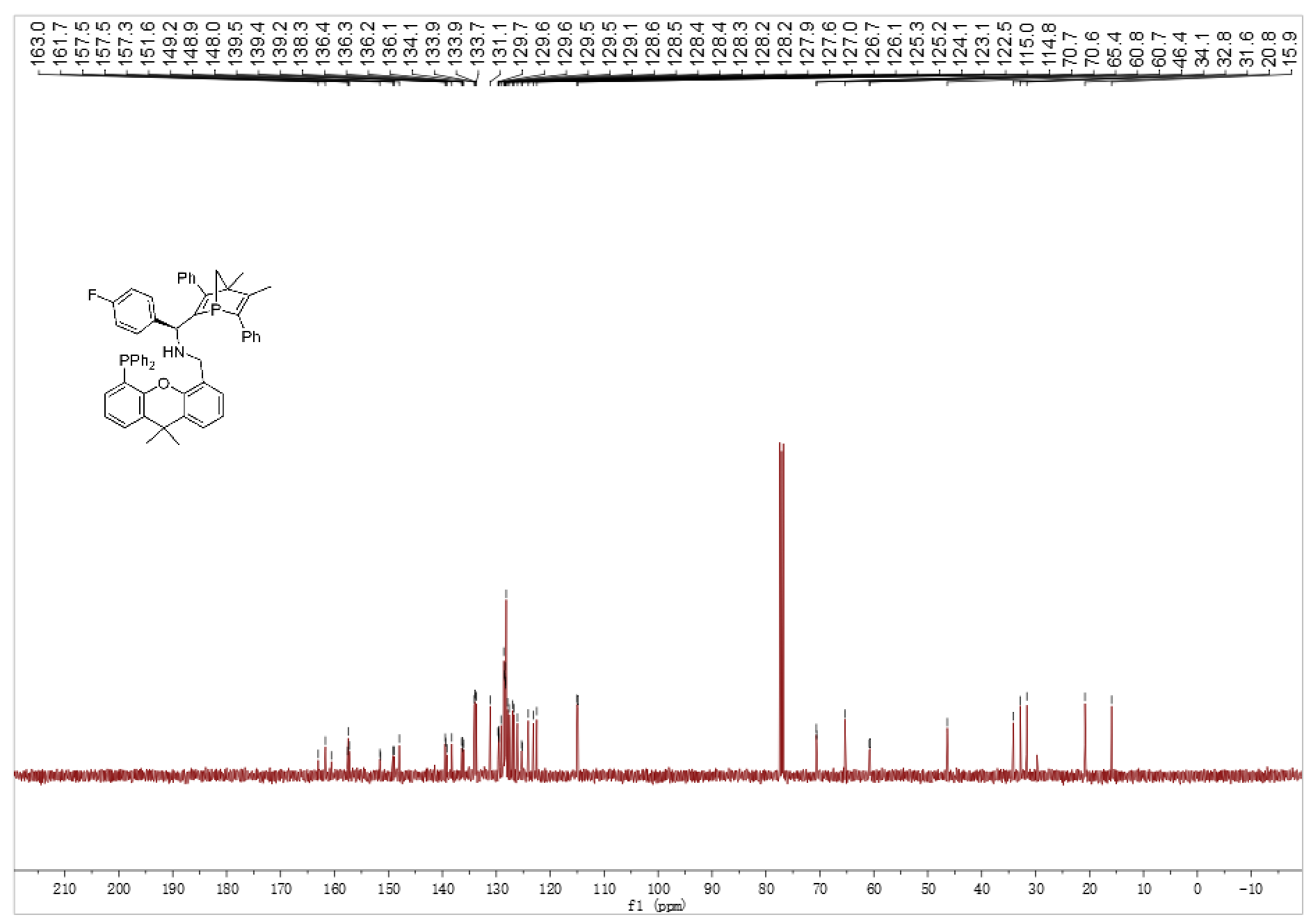
**

^13^C (CDCl_3_, 101 MHz) NMR of compound **MQ Phos-2**


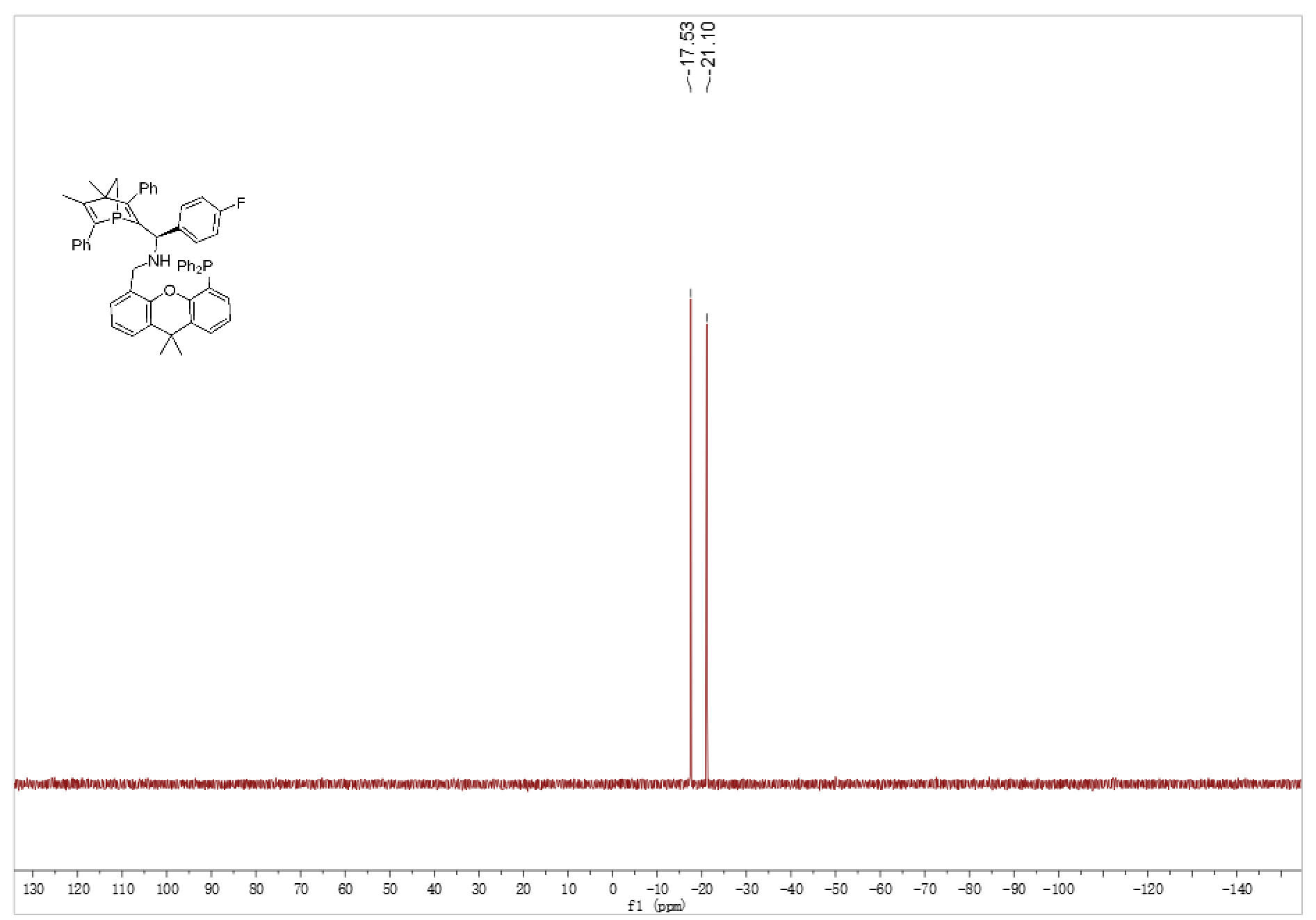


^31^P (CDCl_3_, 162 MHz) NMR of compound **MQ Phos-2^*^**


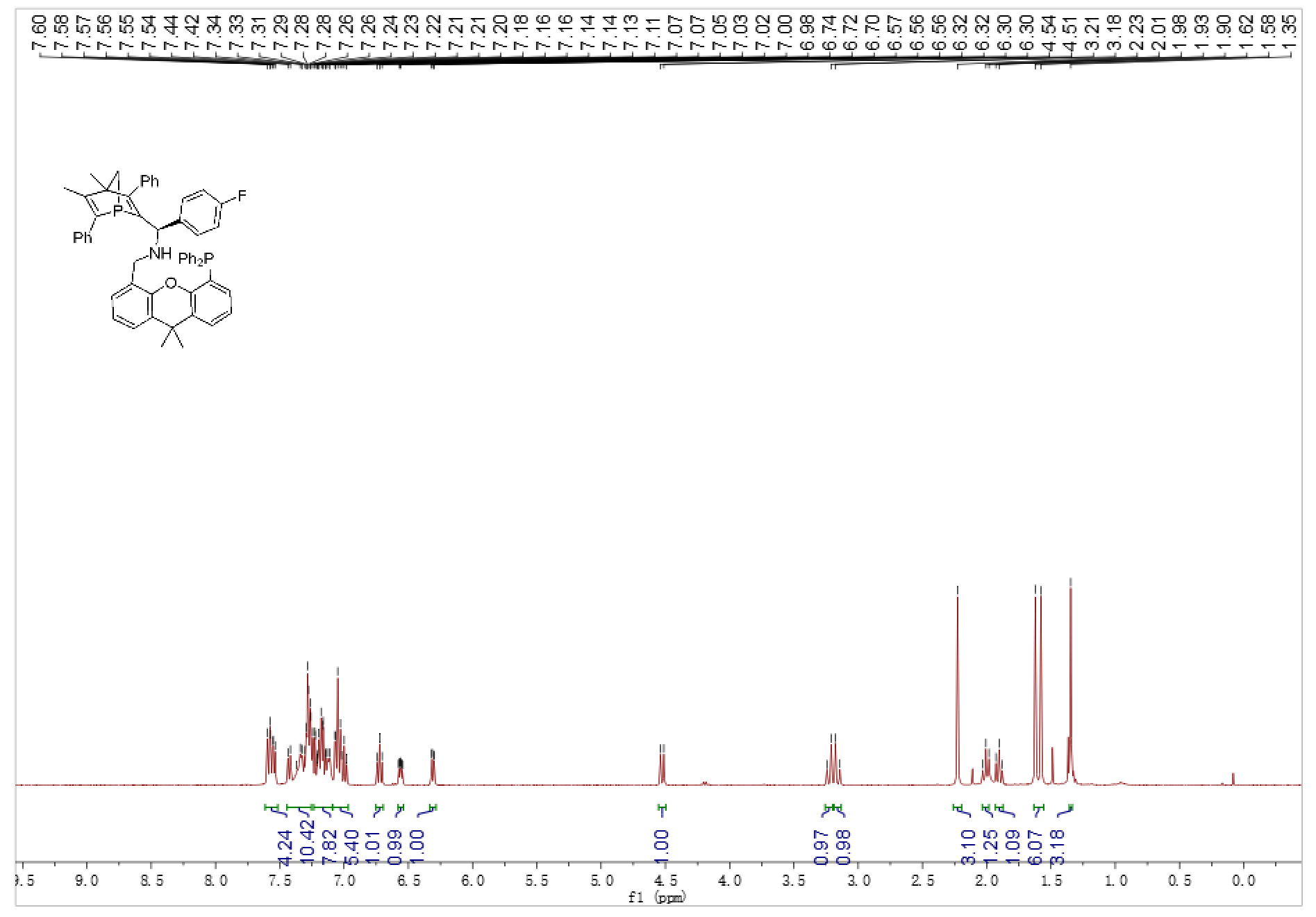


^1^H (CDCl_3_, 400 MHz) NMR of compound **MQ Phos-2^*^**


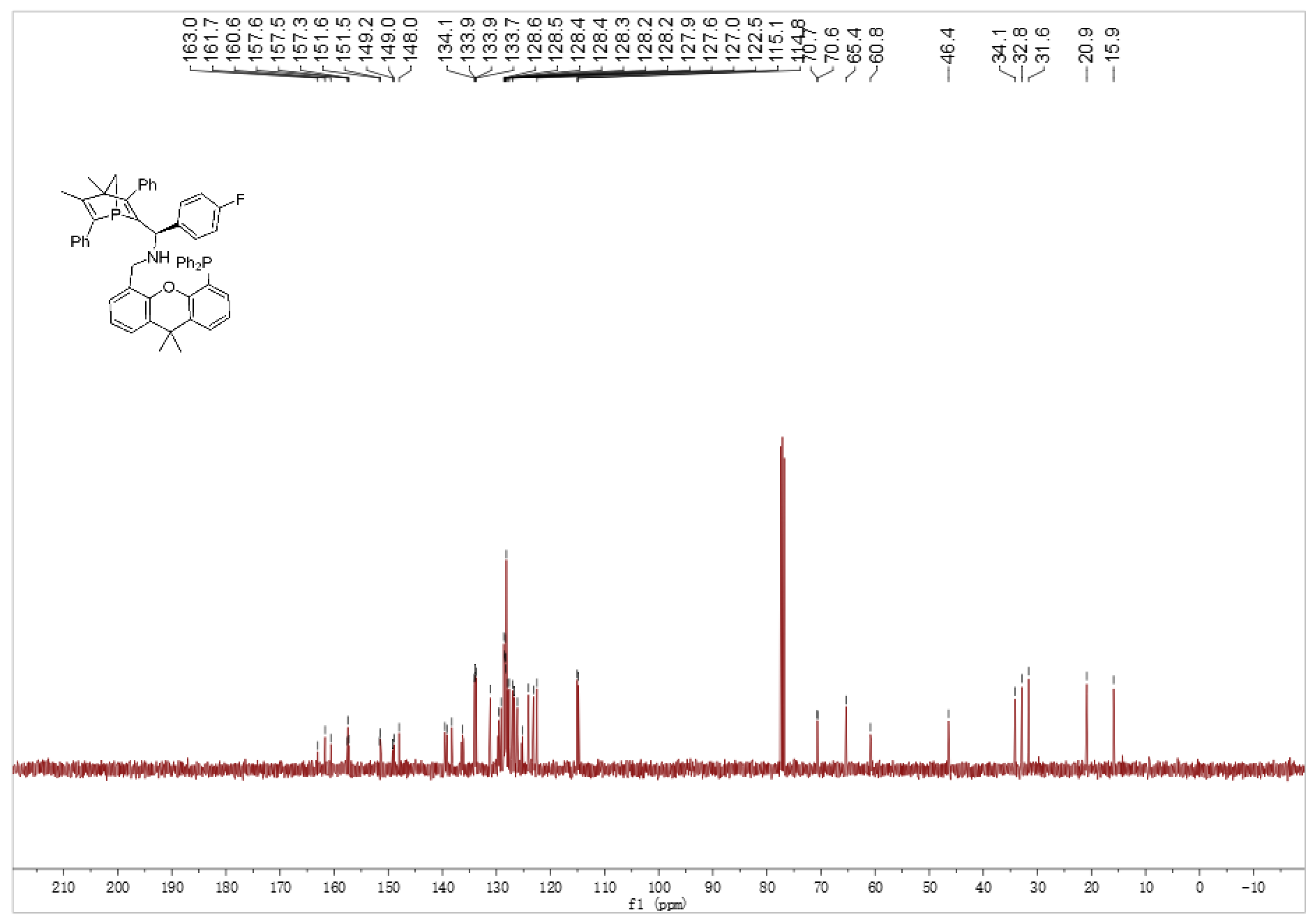


^13^C (CDCl_3_, 101 MHz) NMR of compound **MQ Phos-2^*^**

**
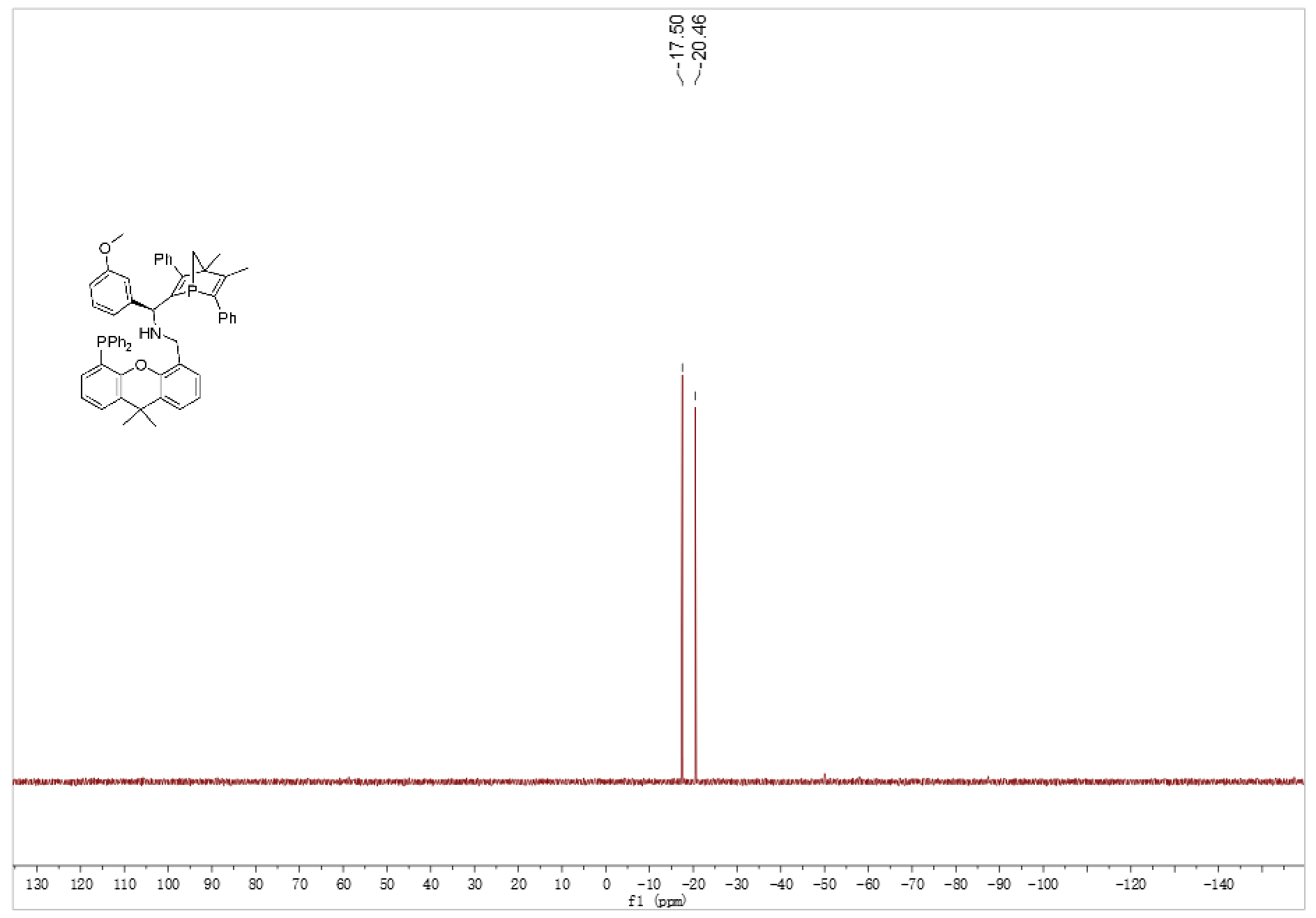
**

^31^P (CDCl_3_, 162 MHz) NMR of compound **MQ Phos-3**


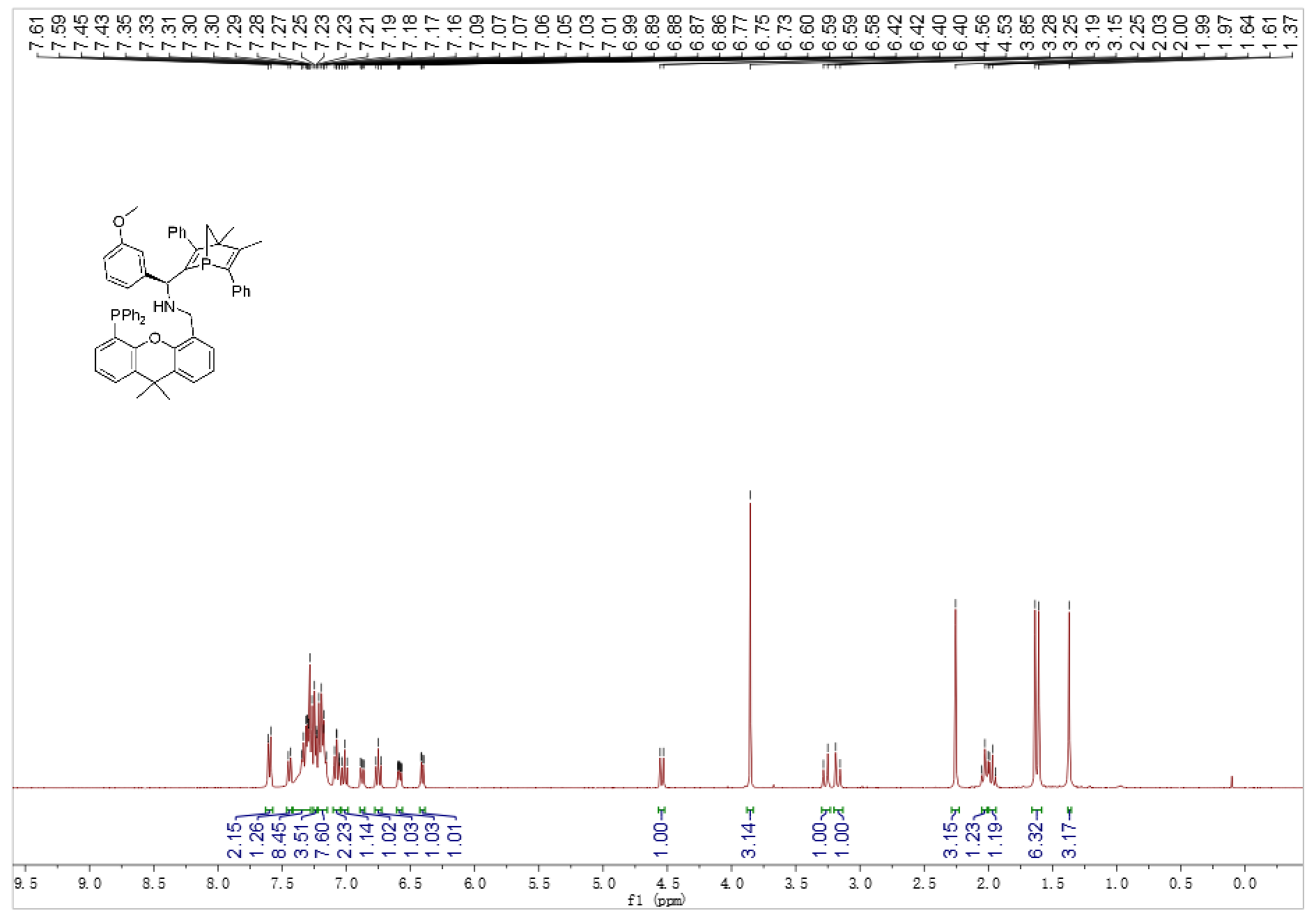


^1^H (CDCl_3_, 400 MHz) NMR of compound **MQ Phos-3**


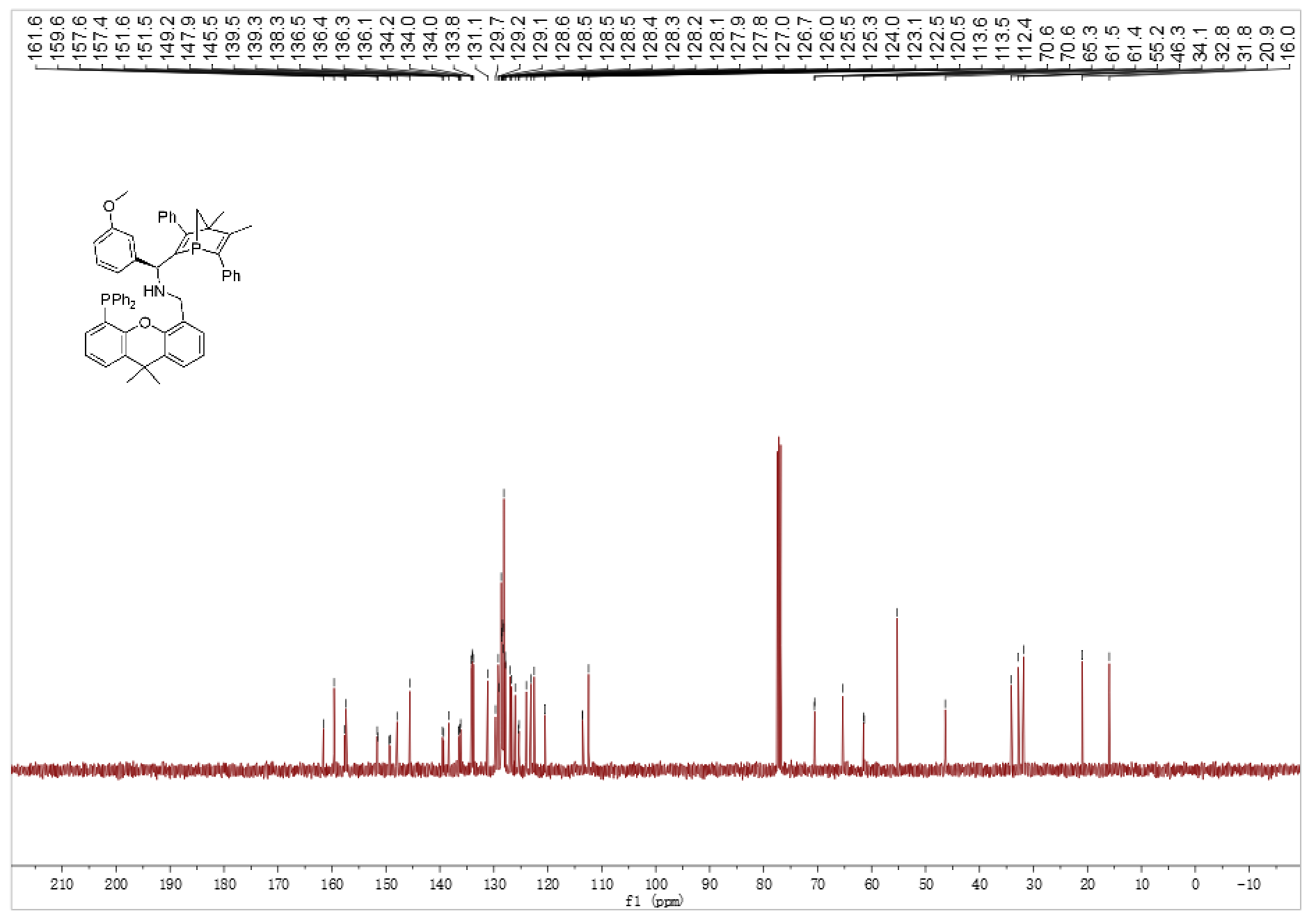


^13^C (CDCl_3_, 101 MHz) NMR of compound **MQ Phos-3**


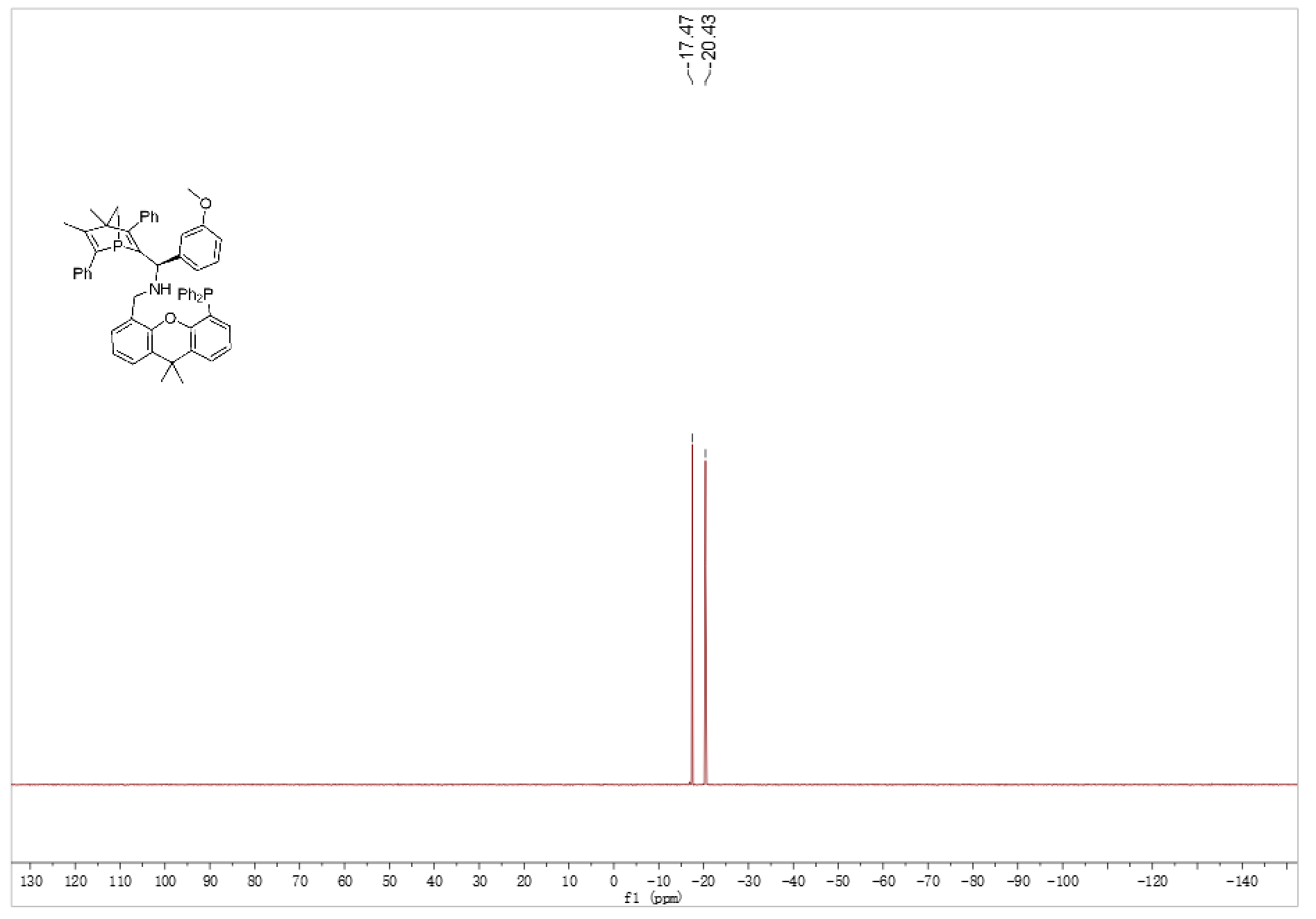


^31^P (CDCl_3_, 162 MHz) NMR of compound **MQ Phos-3^*^**


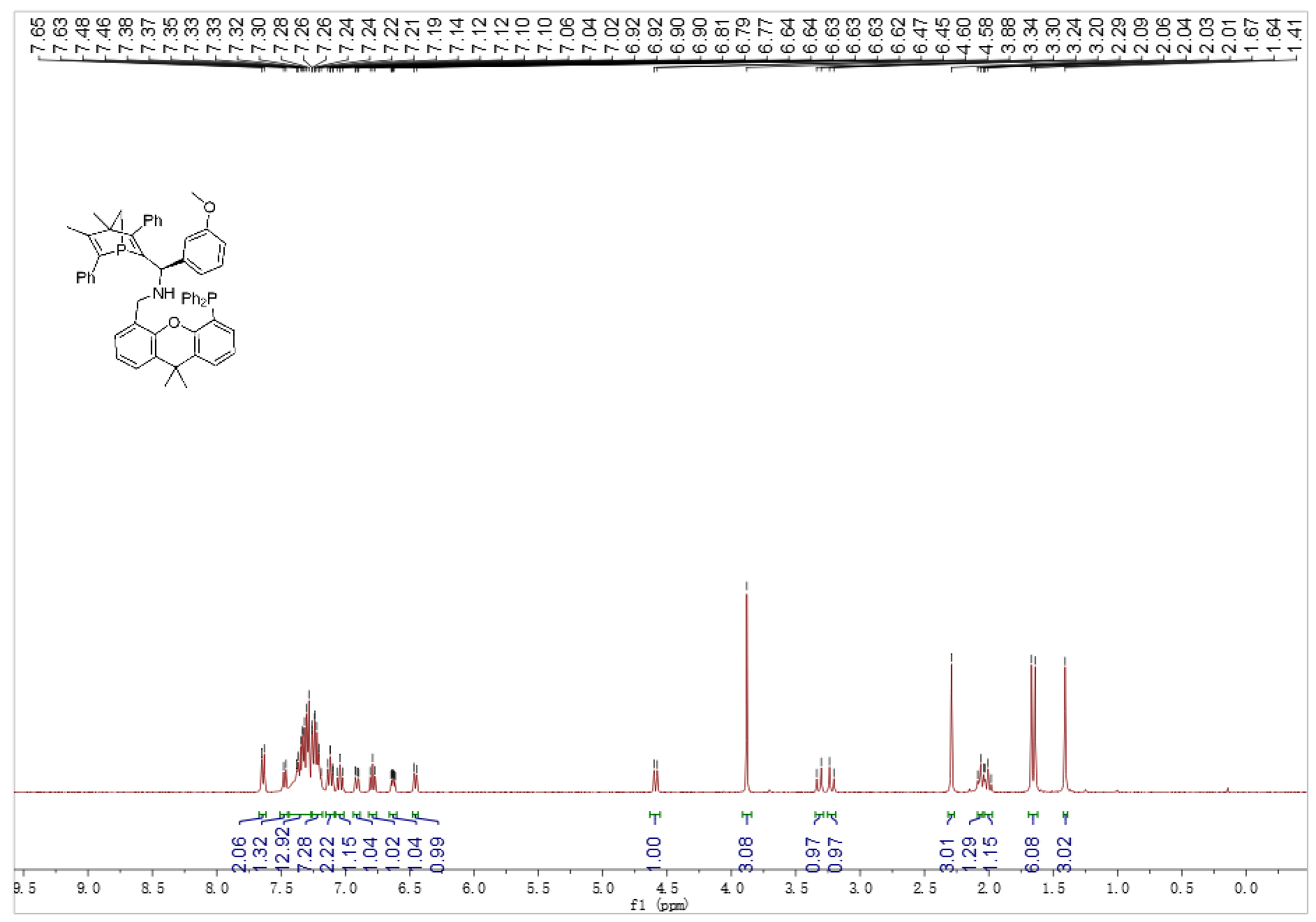


^1^H (CDCl_3_, 400 MHz) NMR of compound **MQ Phos-3^*^**


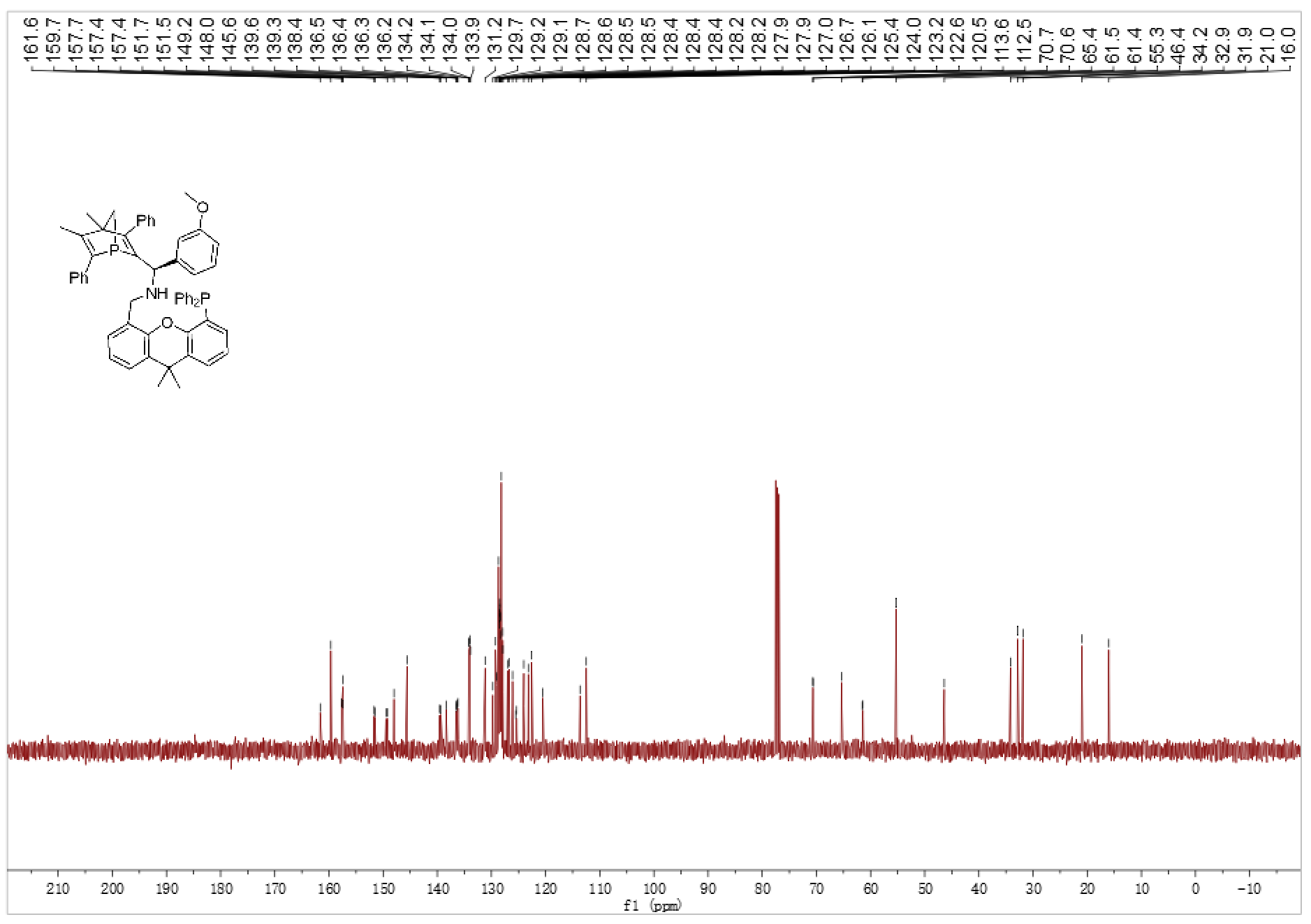


^13^C (CDCl_3_, 101 MHz) NMR of compound **MQ Phos-3^*^**


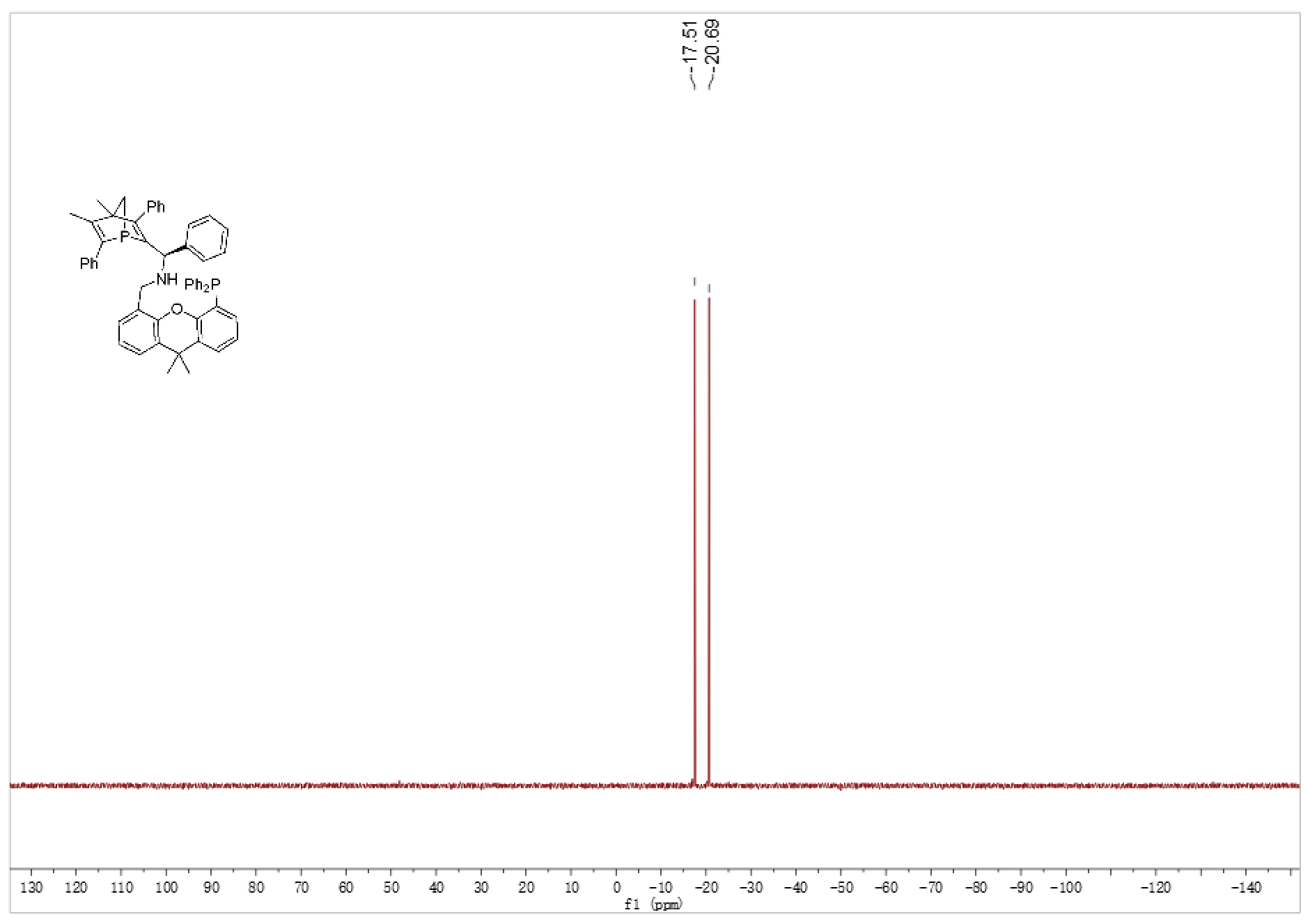


^31^P (CDCl_3_, 162 MHz) NMR of compound **MQ Phos-4^*^**


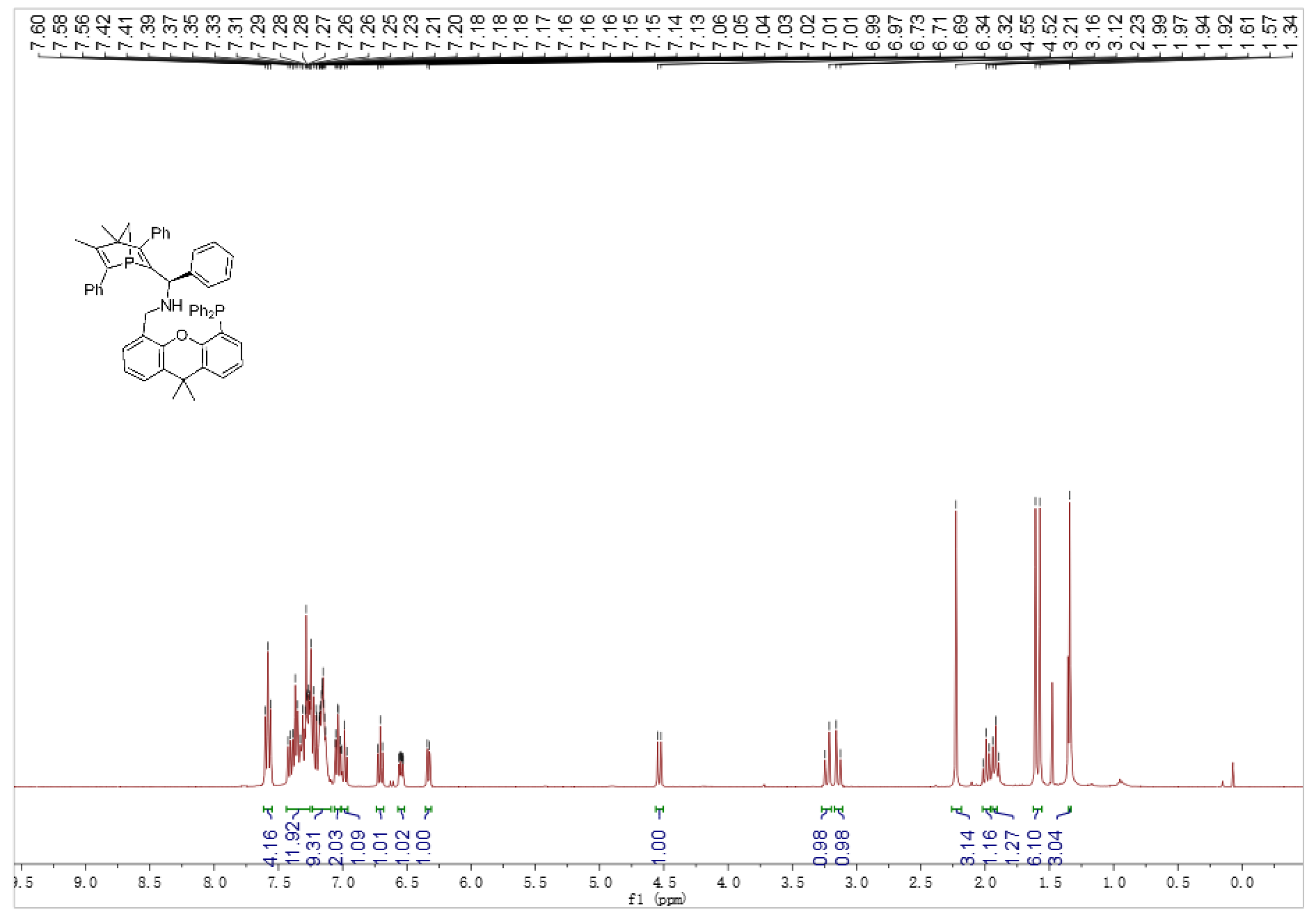


^1^H (CDCl_3_, 400 MHz) NMR of compound **MQ Phos-4^*^**


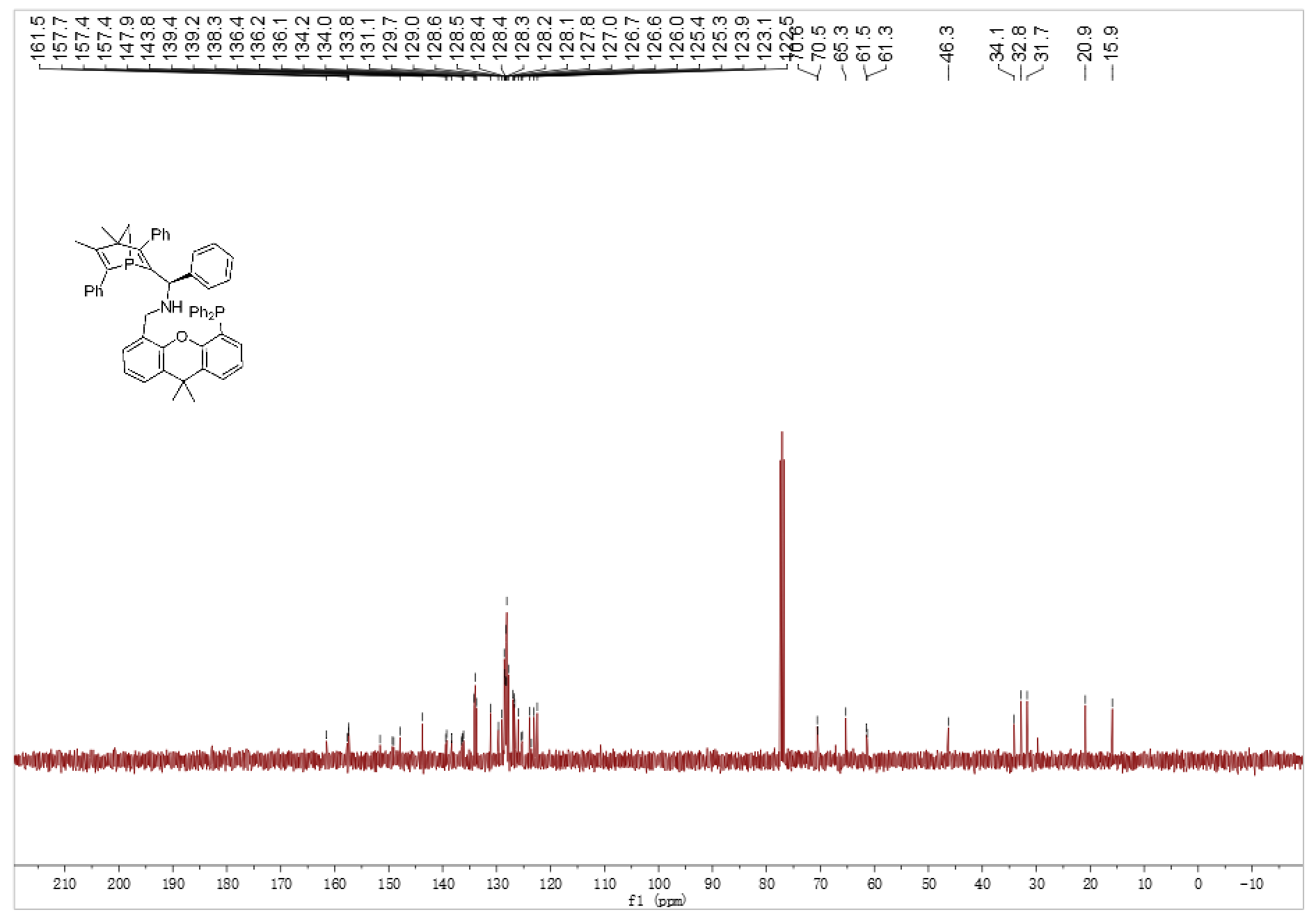


^13^C (CDCl_3_, 101 MHz) NMR of compound **MQ Phos-4^*^**

**^
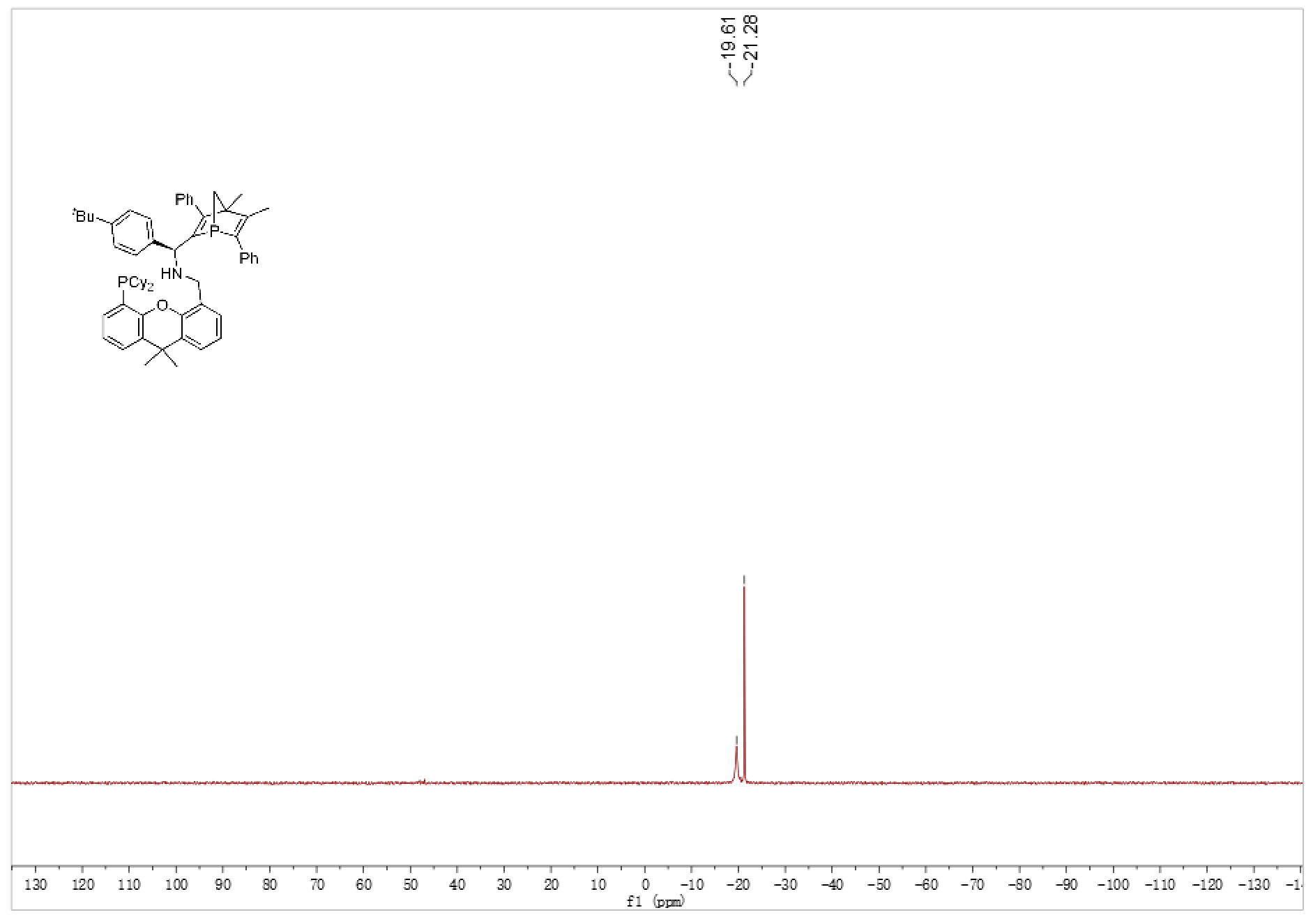
^**

^31^P (CDCl_3_, 162 MHz) NMR of compound **MQ Phos-5**


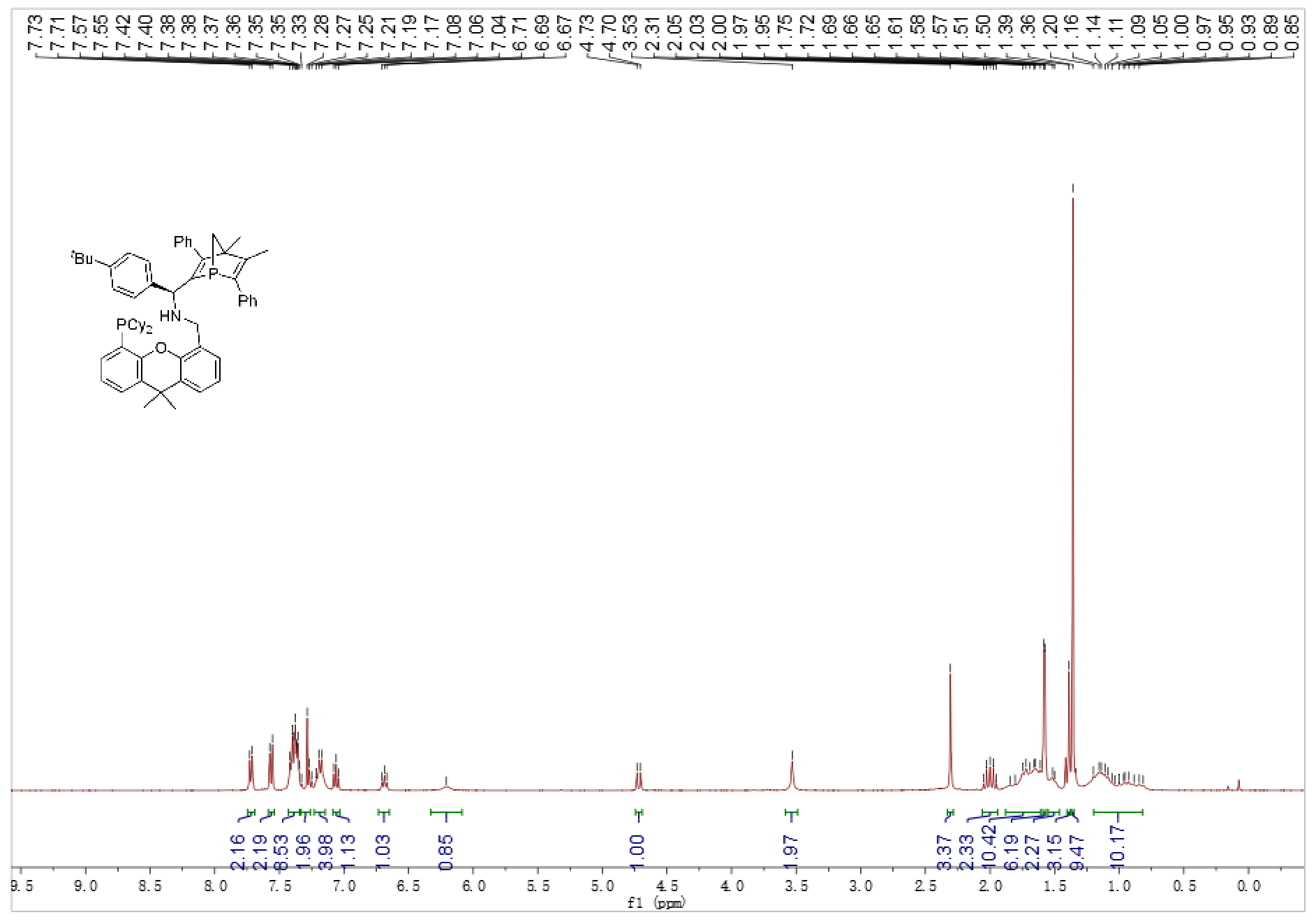


^1^H (CDCl_3_, 400 MHz) NMR of compound **MQ Phos-5**

**
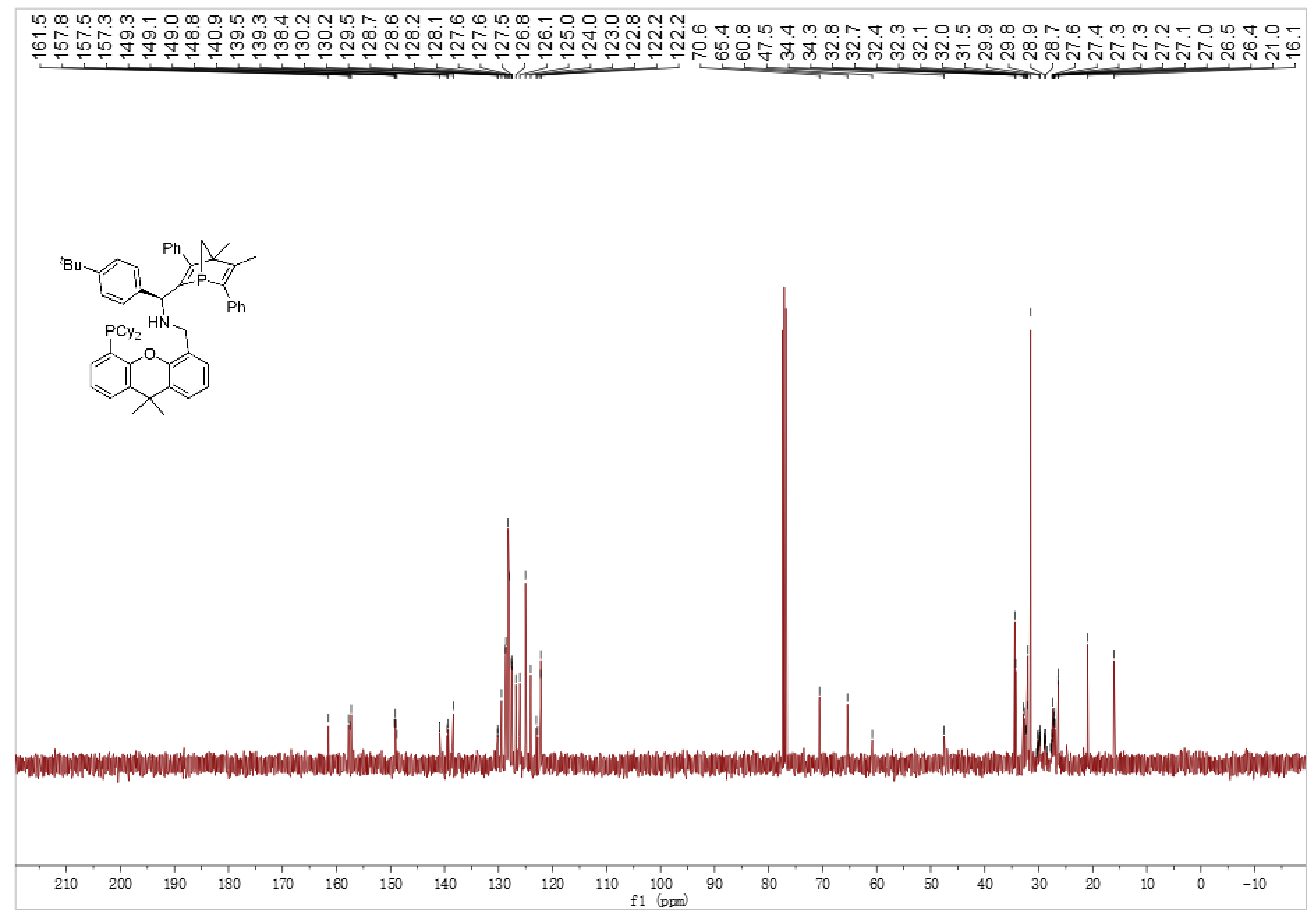
**

^13^C (CDCl_3_, 101 MHz) NMR of compound **MQ Phos-5**

**
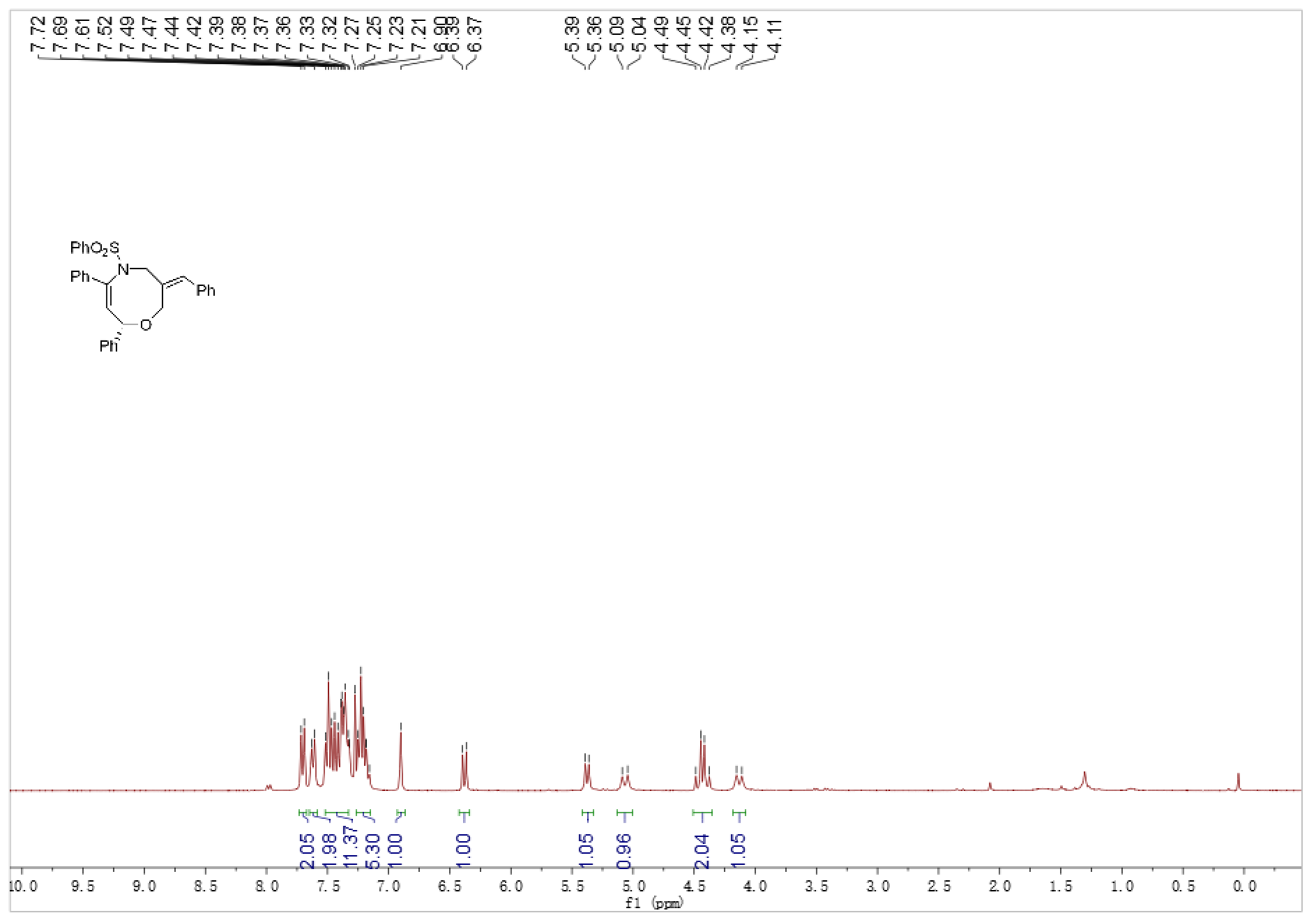
**

^1^H (CDCl_3_, 300 MHz) NMR of compound **3**


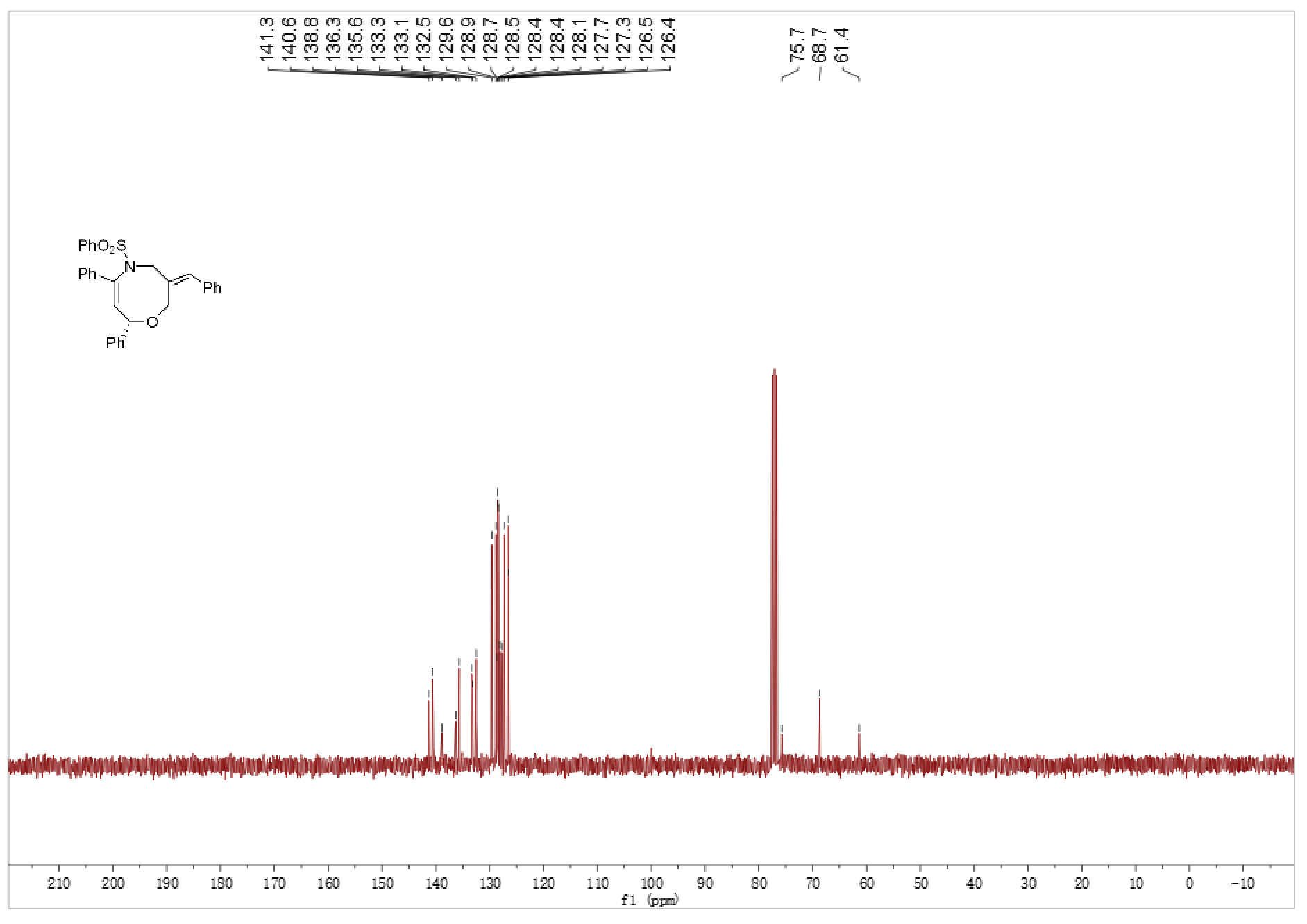


^13^C (CDCl_3_, 75 MHz) NMR of compound **3**

**
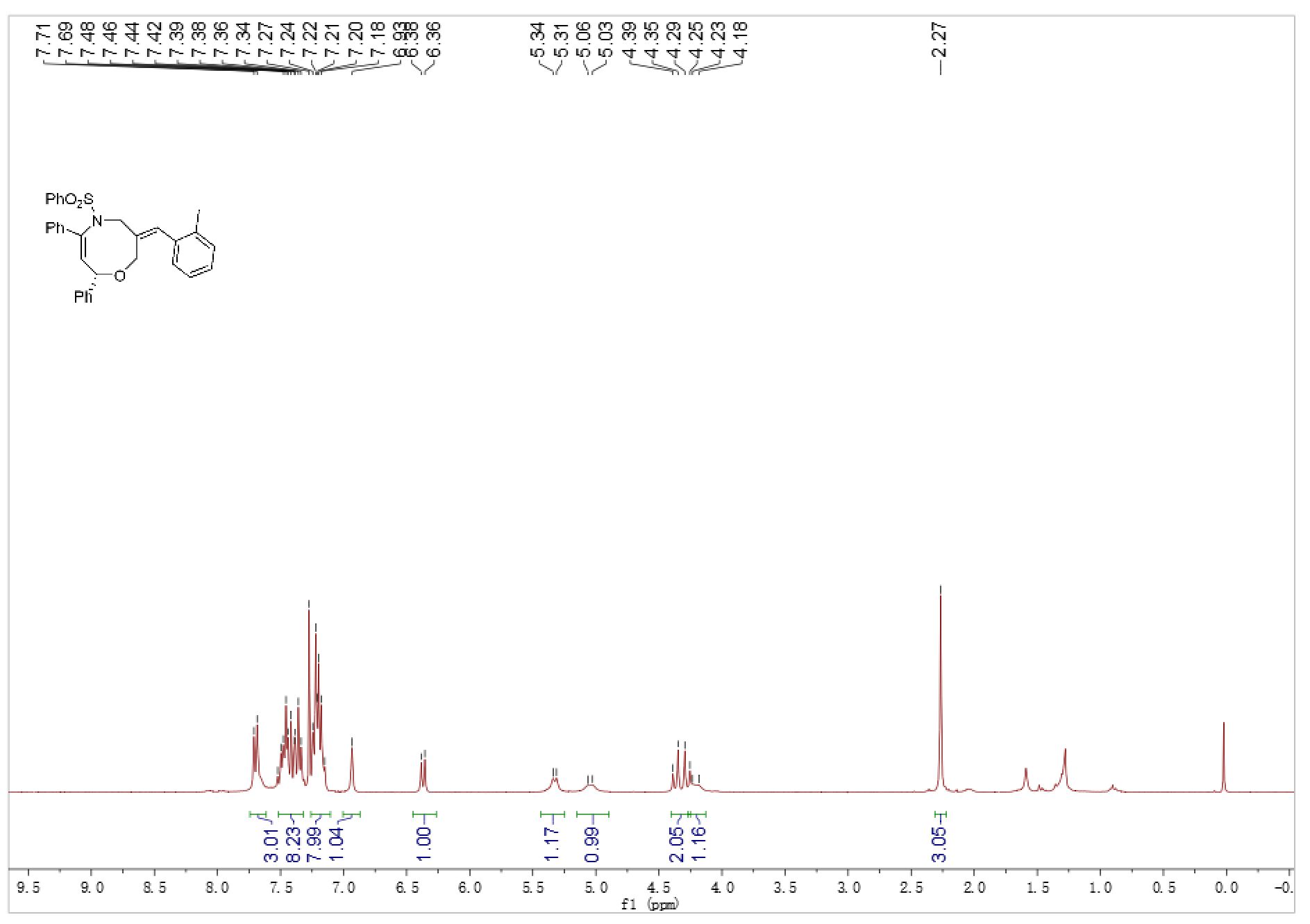
**

^1^H (CDCl_3_, 300 MHz) NMR of compound **4**

**
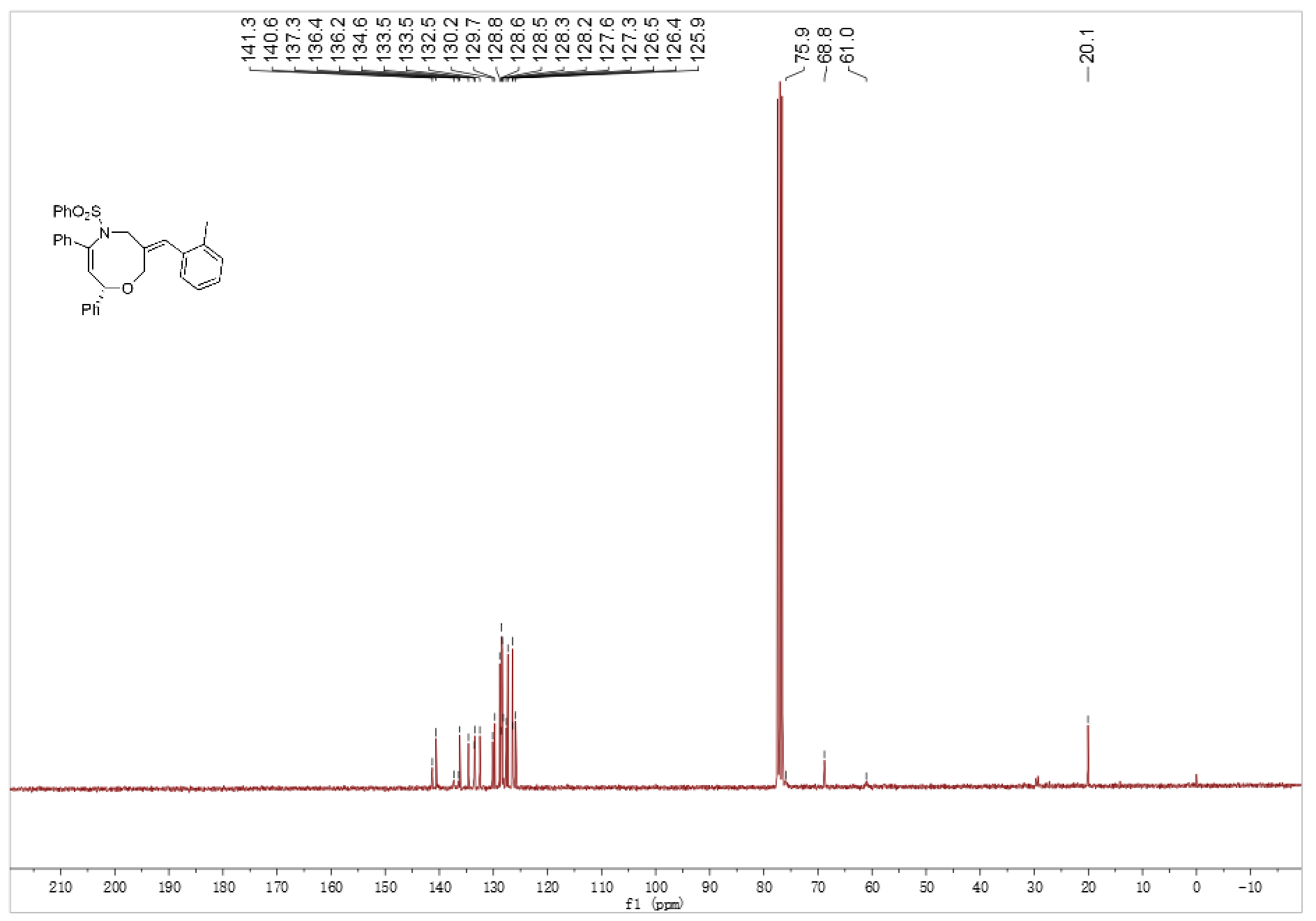
**

^13^C (CDCl_3_, 75 MHz) NMR of compound **4**

**
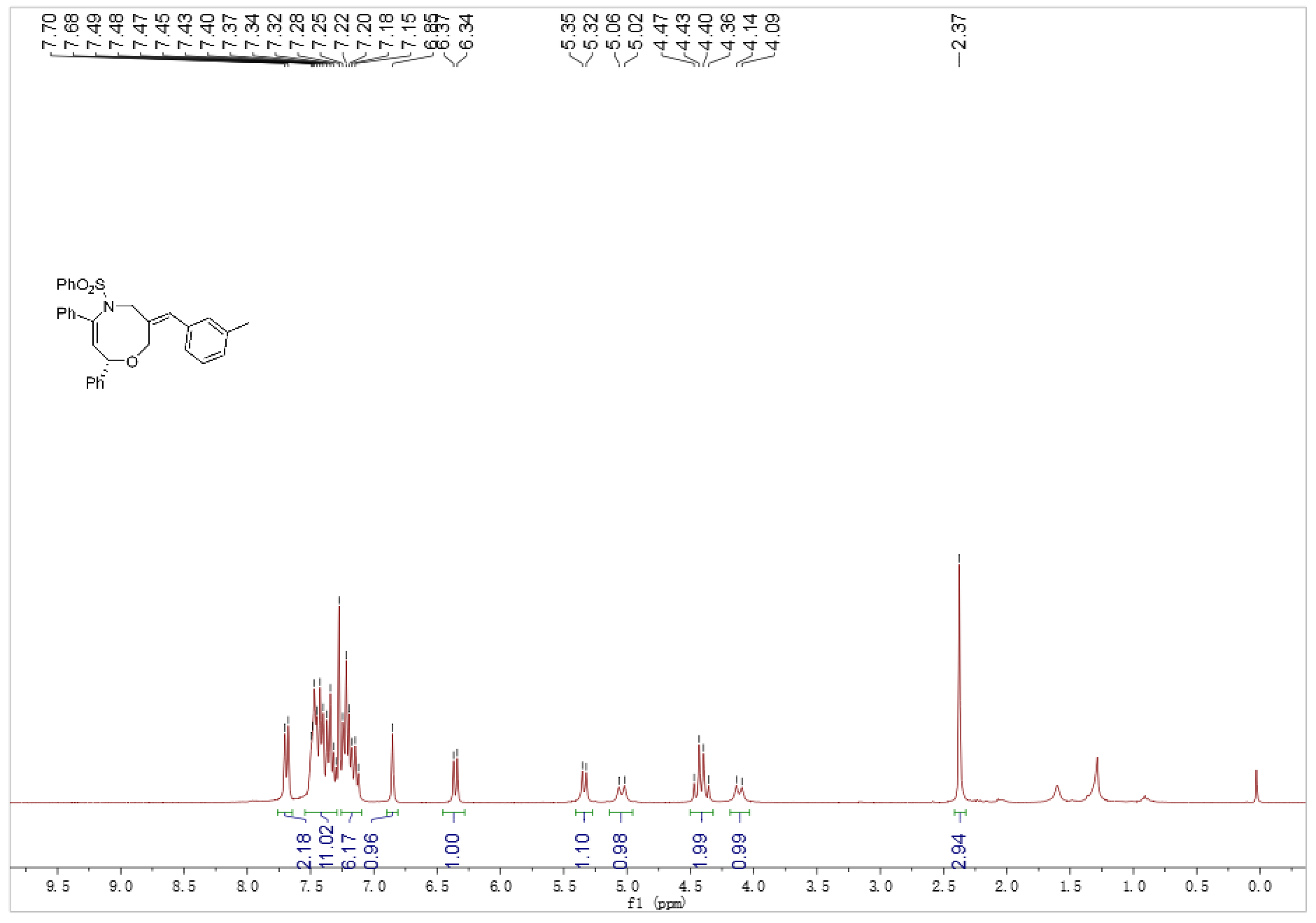
**

^1^H (CDCl_3_, 300 MHz) NMR of compound **5**

**
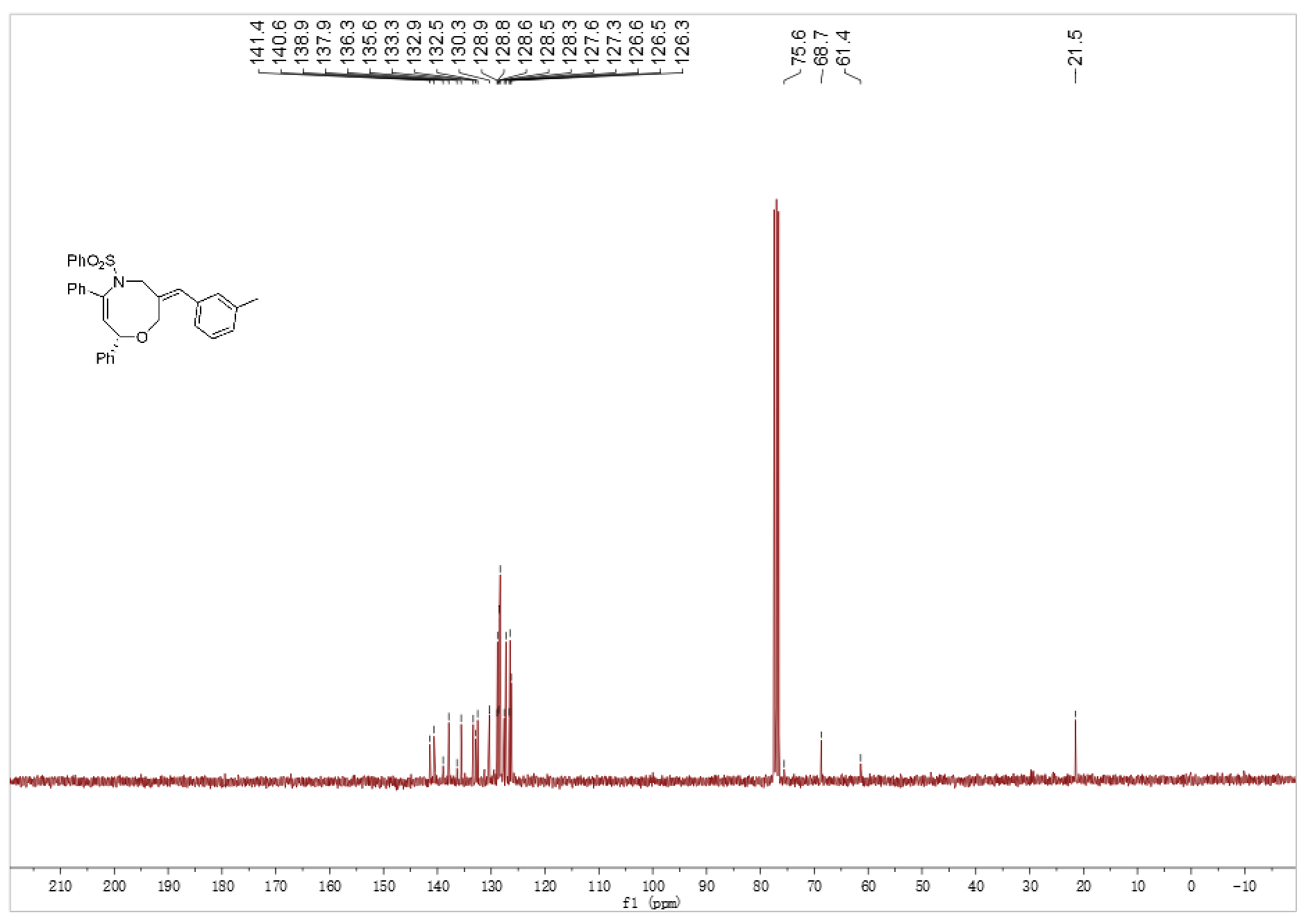
**

^13^C (CDCl_3_, 75 MHz) NMR of compound **5**

**
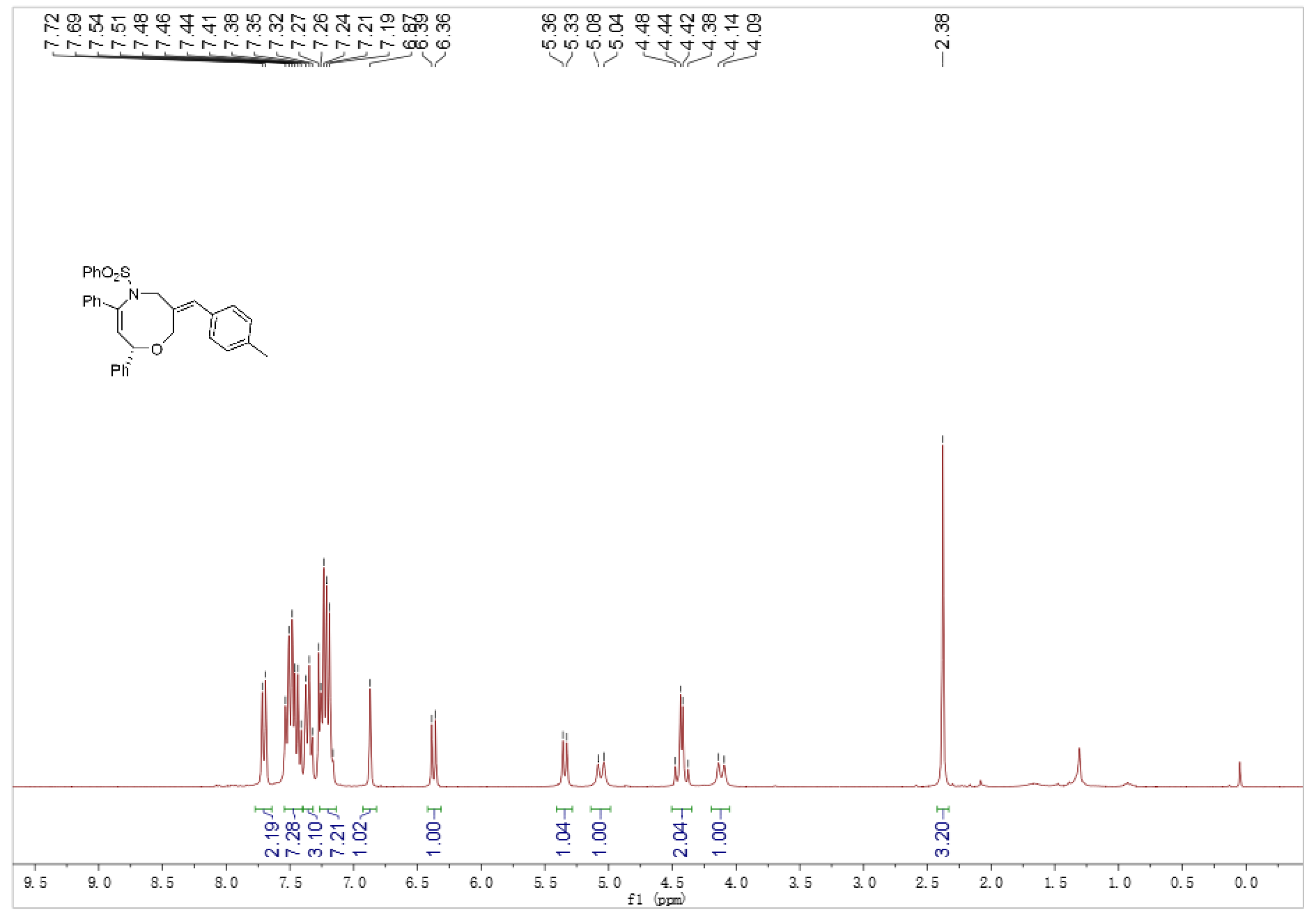
**

^1^H (CDCl_3_, 300 MHz) NMR of compound **6**

**
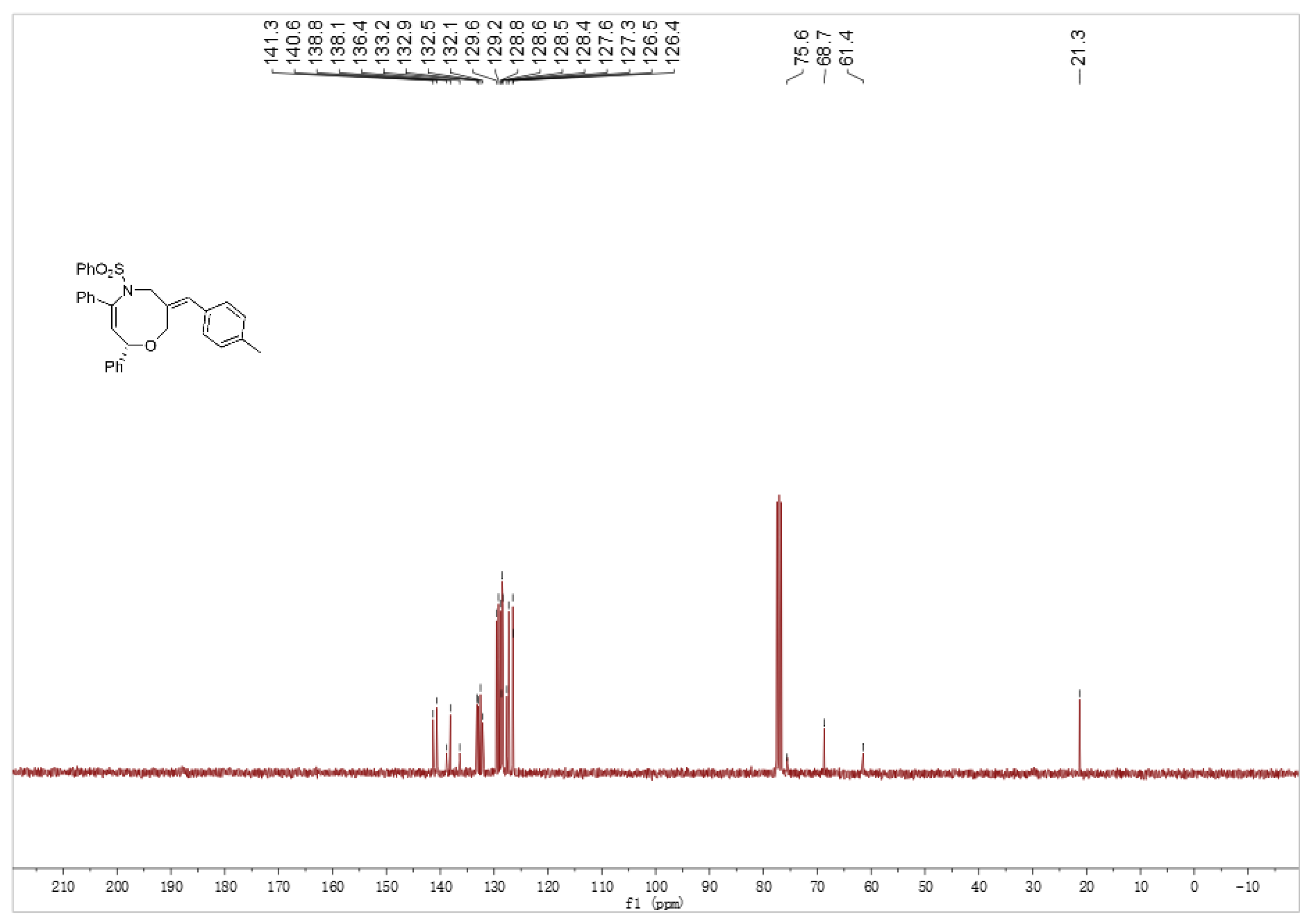
**

^13^C (CDCl_3_, 75 MHz) NMR of compound **6**

**
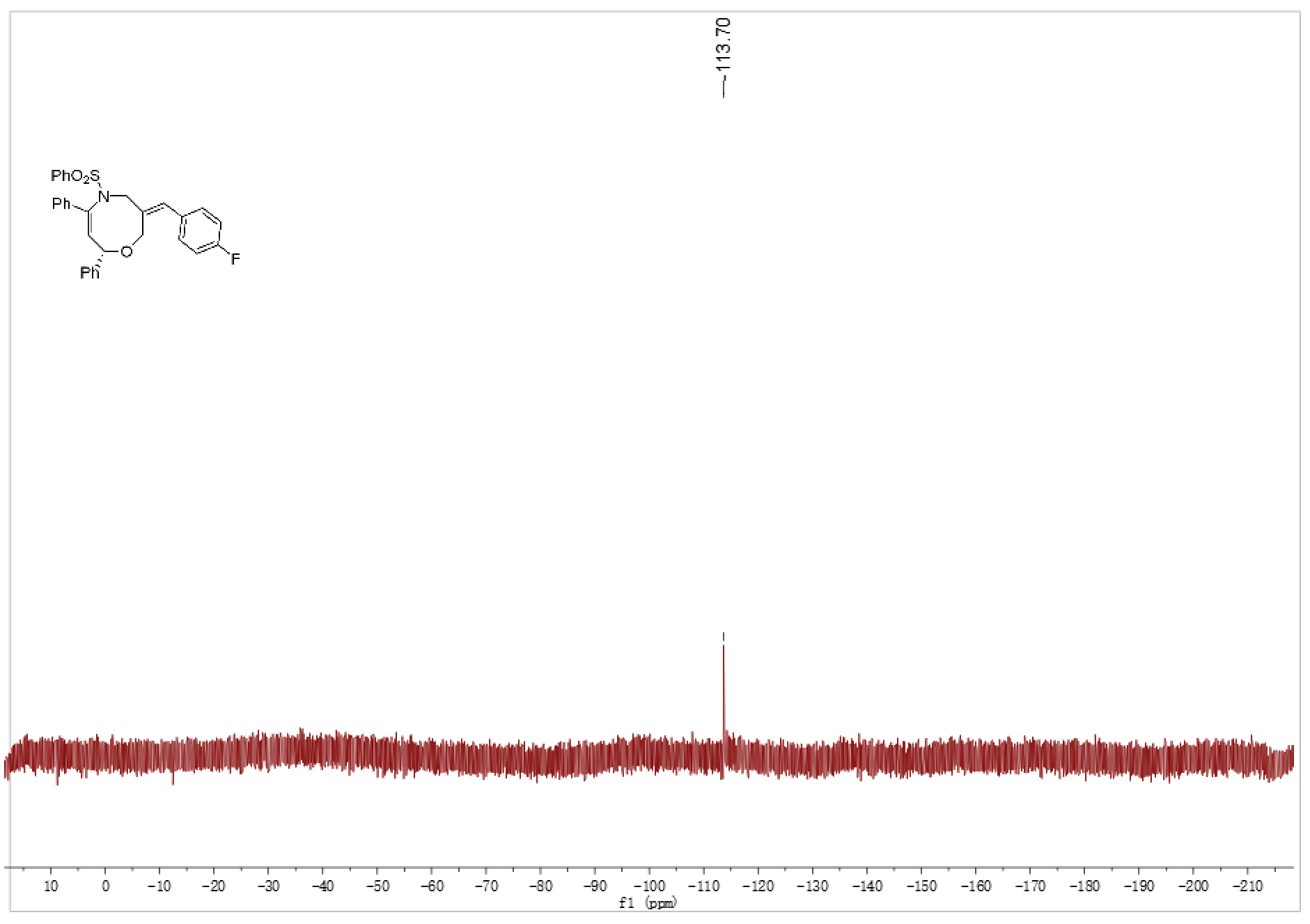
**

^19^F (CDCl_3_, 282 MHz) NMR of compound **7**

**
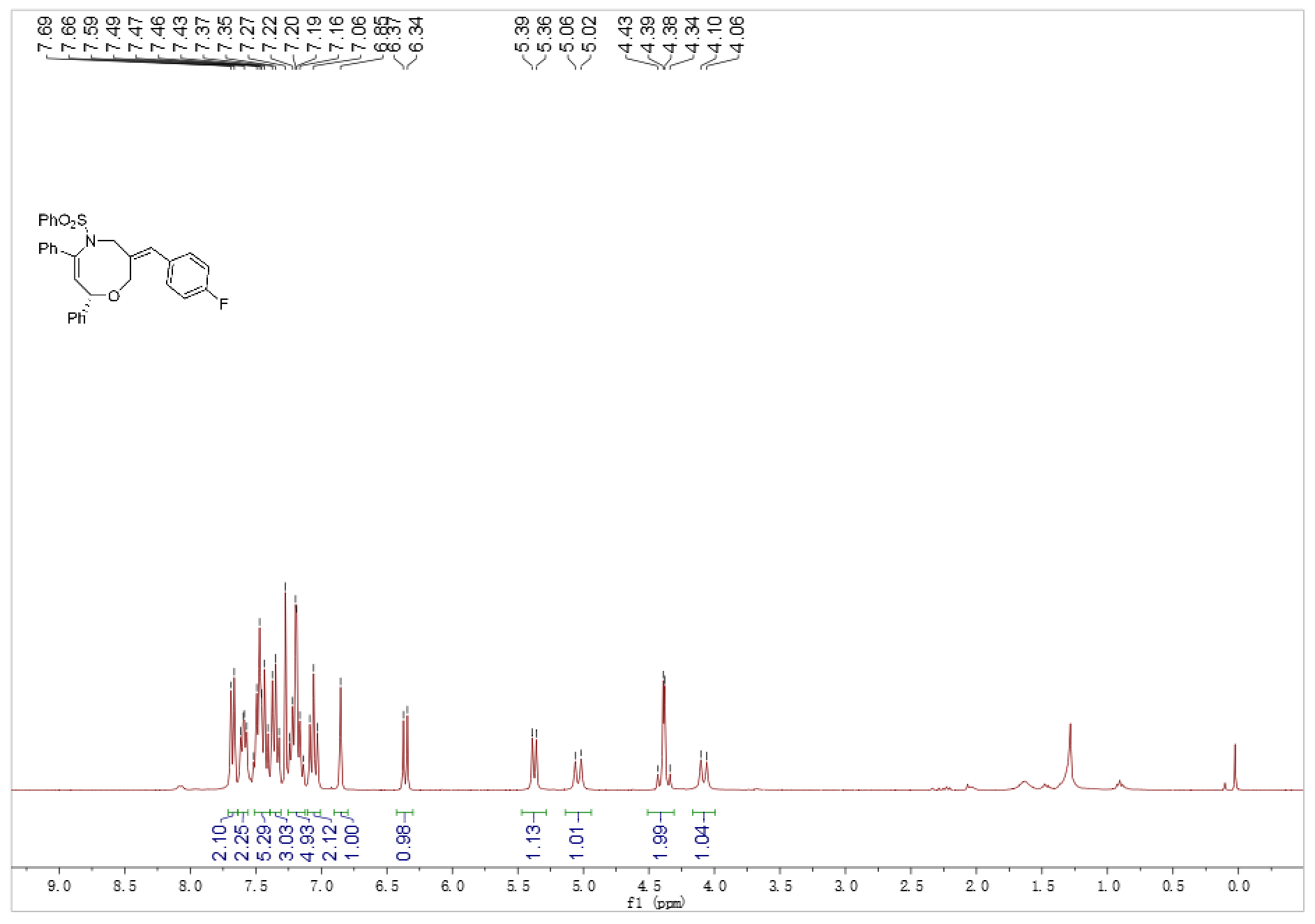
**

^1^H (CDCl_3_, 300 MHz) NMR of compound **7**

**
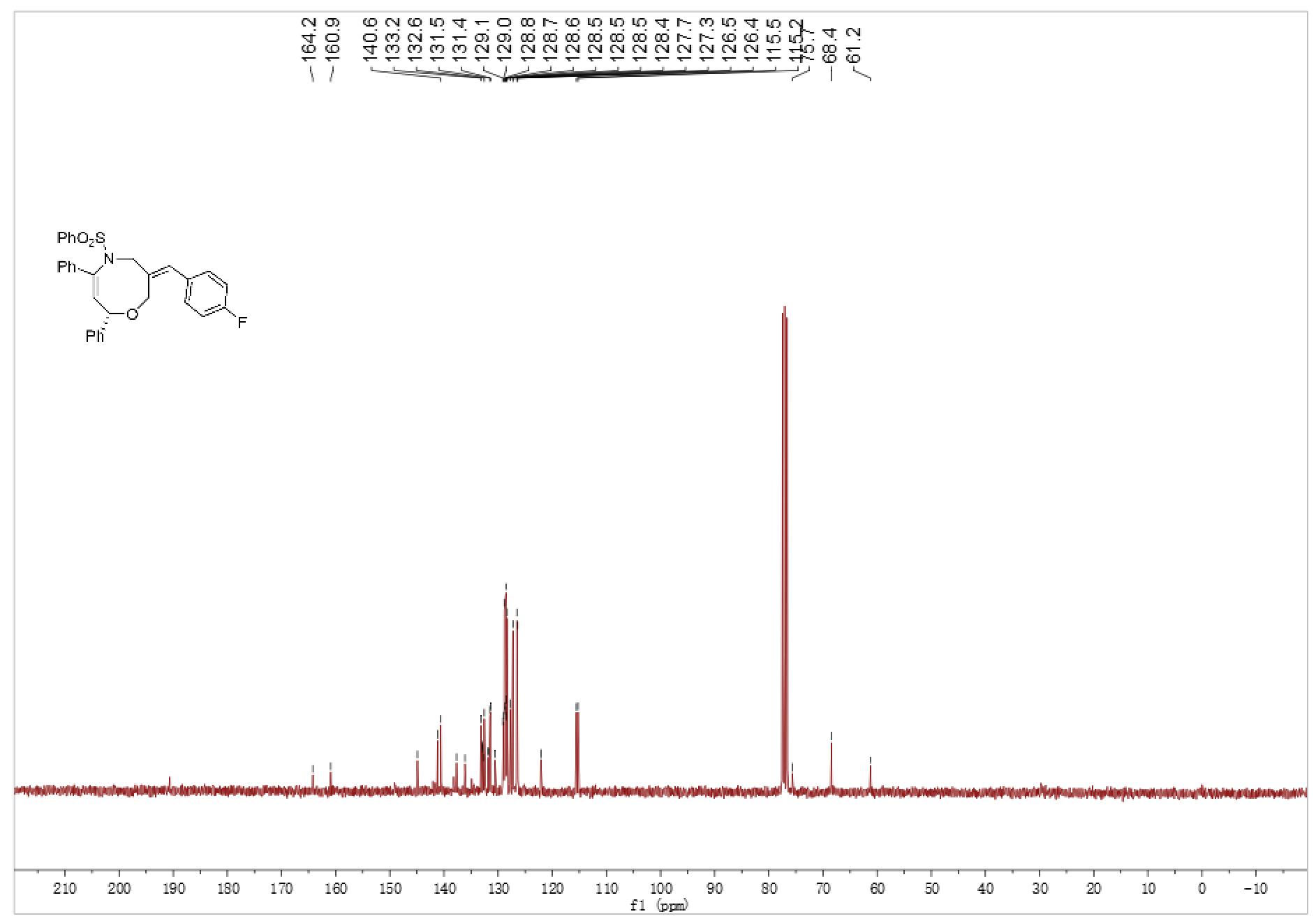
**

^13^C (CDCl_3_, 75 MHz) NMR of compound **7**

**
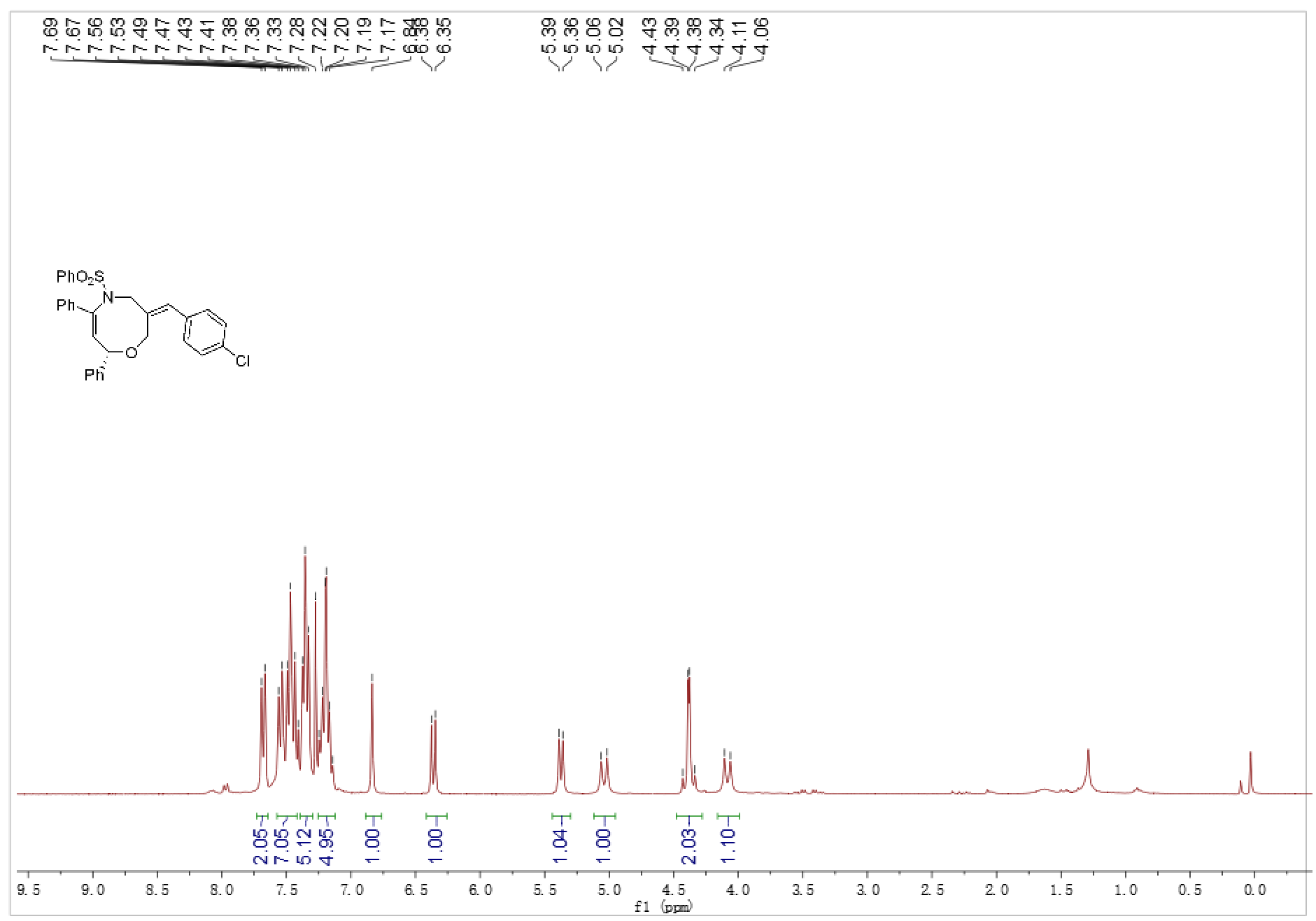
**

^1^H (CDCl_3_, 300 MHz) NMR of compound **8**

**
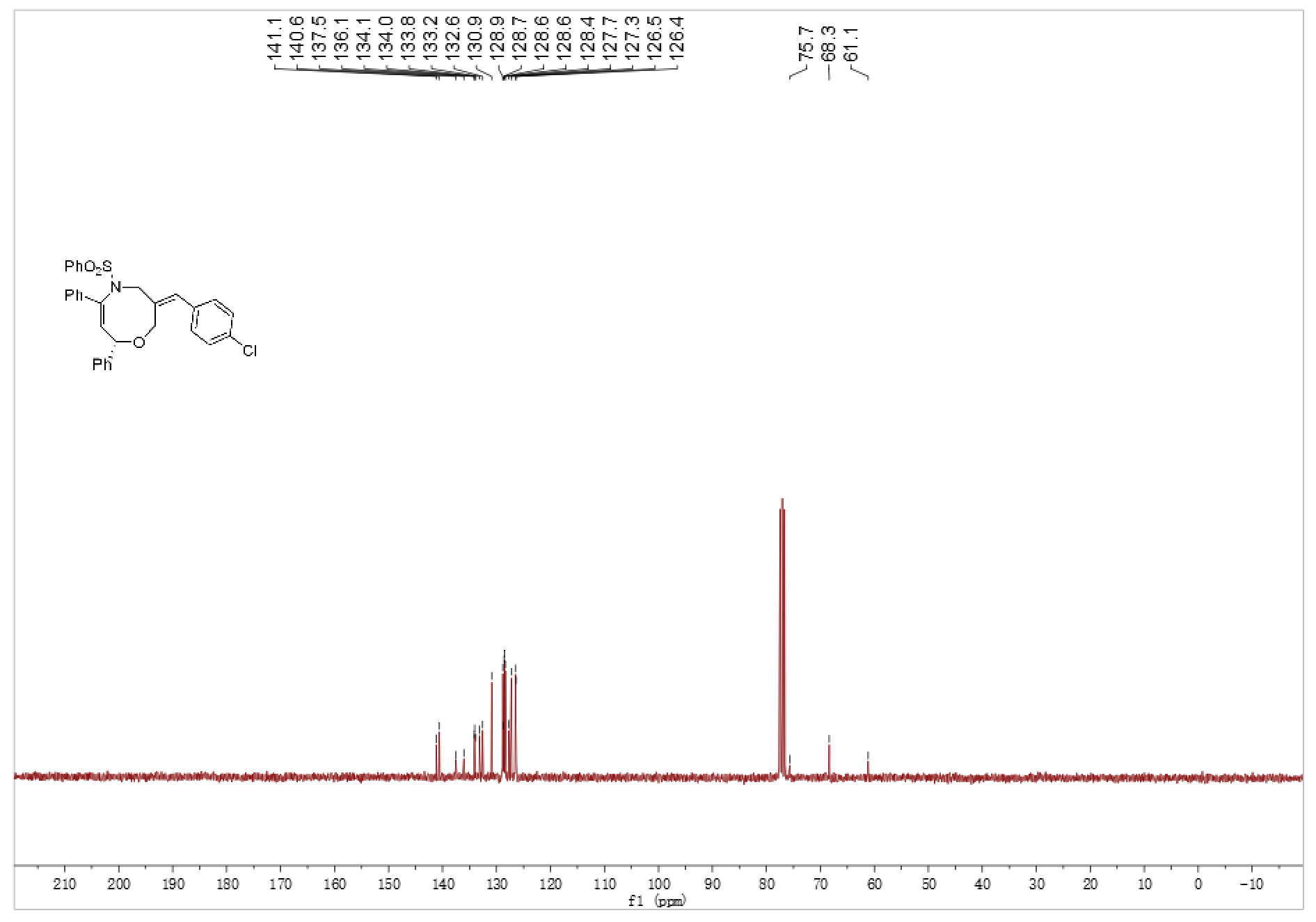
**

^13^C (CDCl_3_, 75 MHz) NMR of compound **8**

**
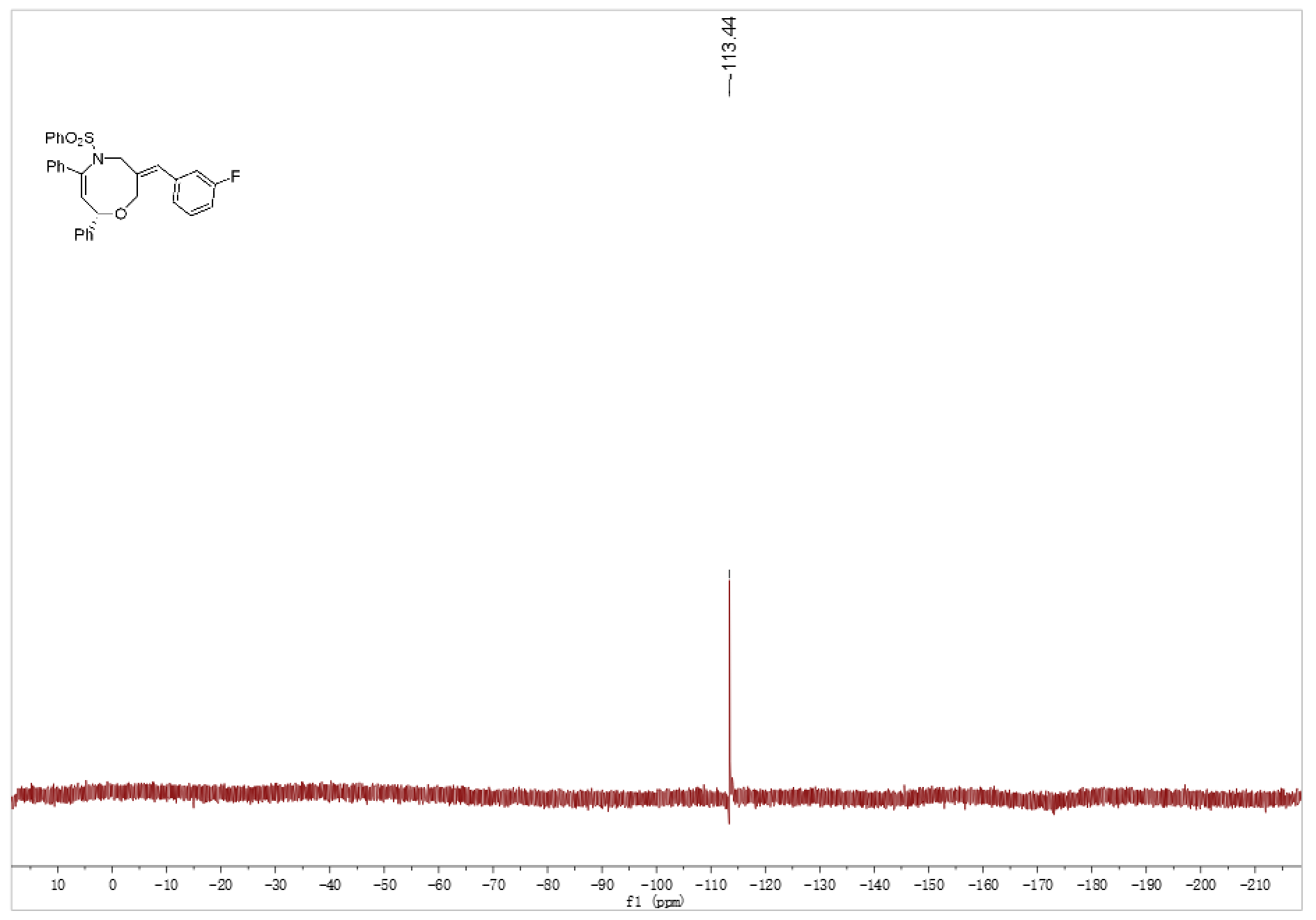
**

^19^F (CDCl_3_, 282 MHz) NMR of compound **9**

**
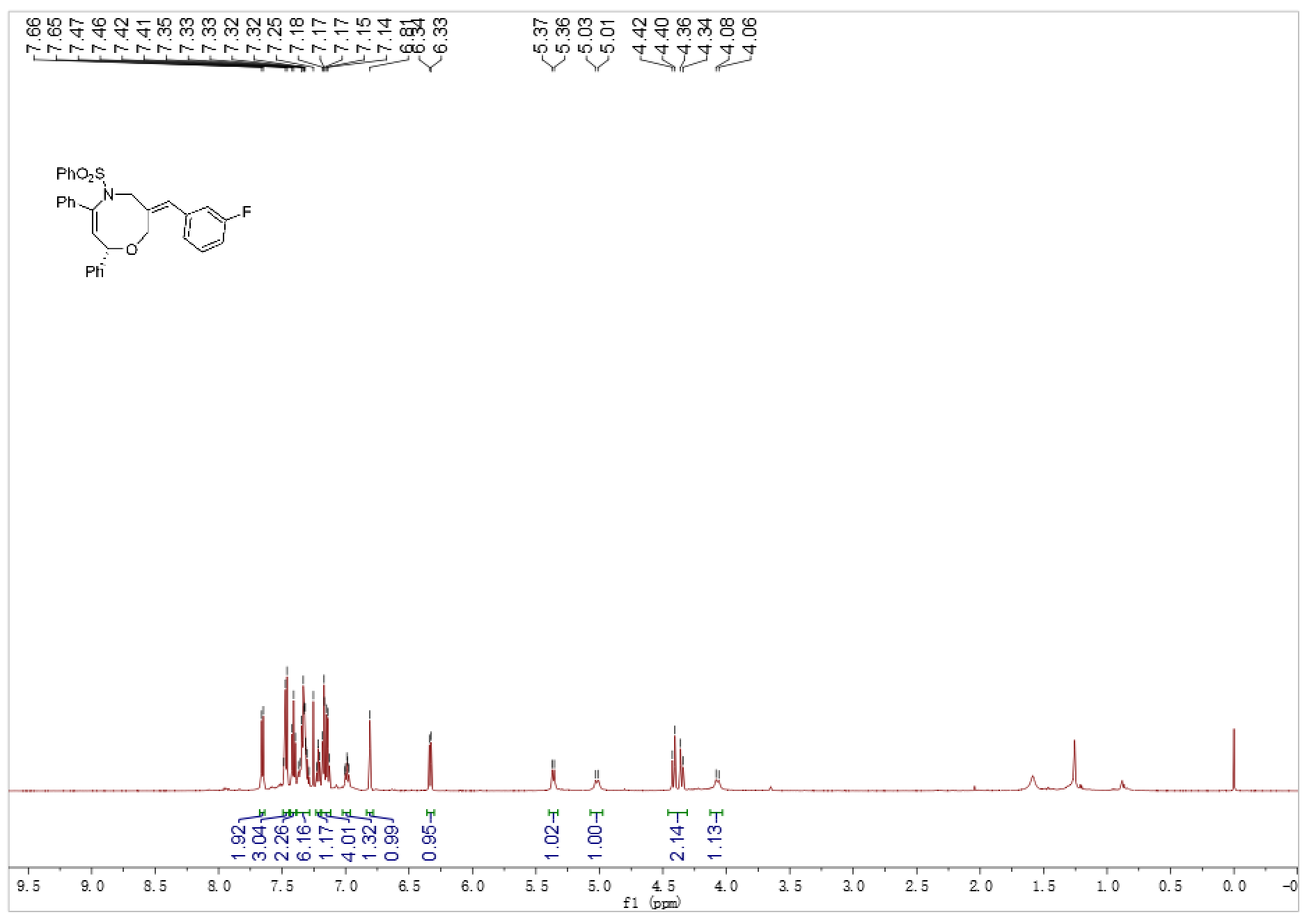
**

^1^H (CDCl_3_, 600 MHz) NMR of compound **9**

**
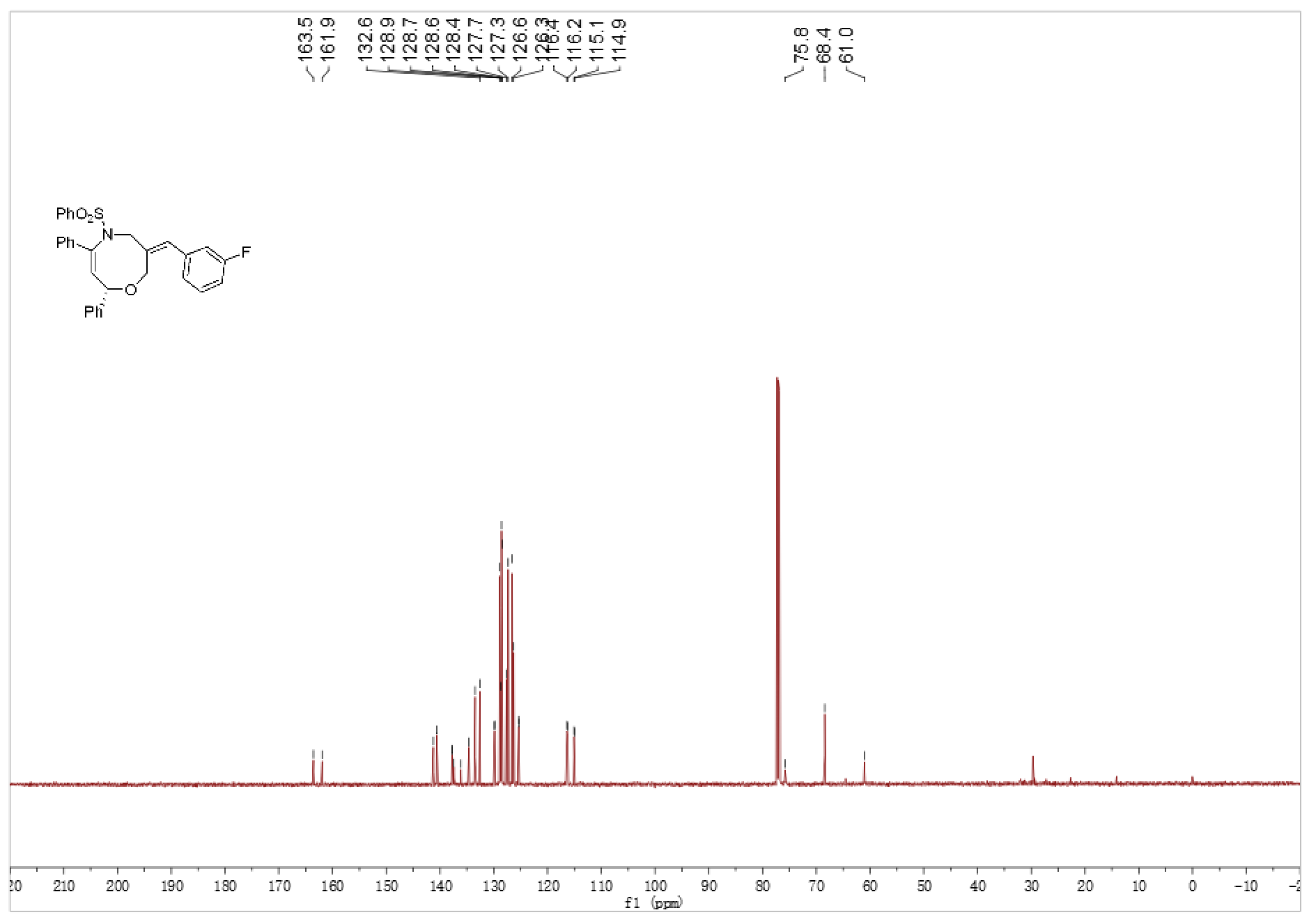
**

^13^C (CDCl_3_, 151 MHz) NMR of compound **9**

**
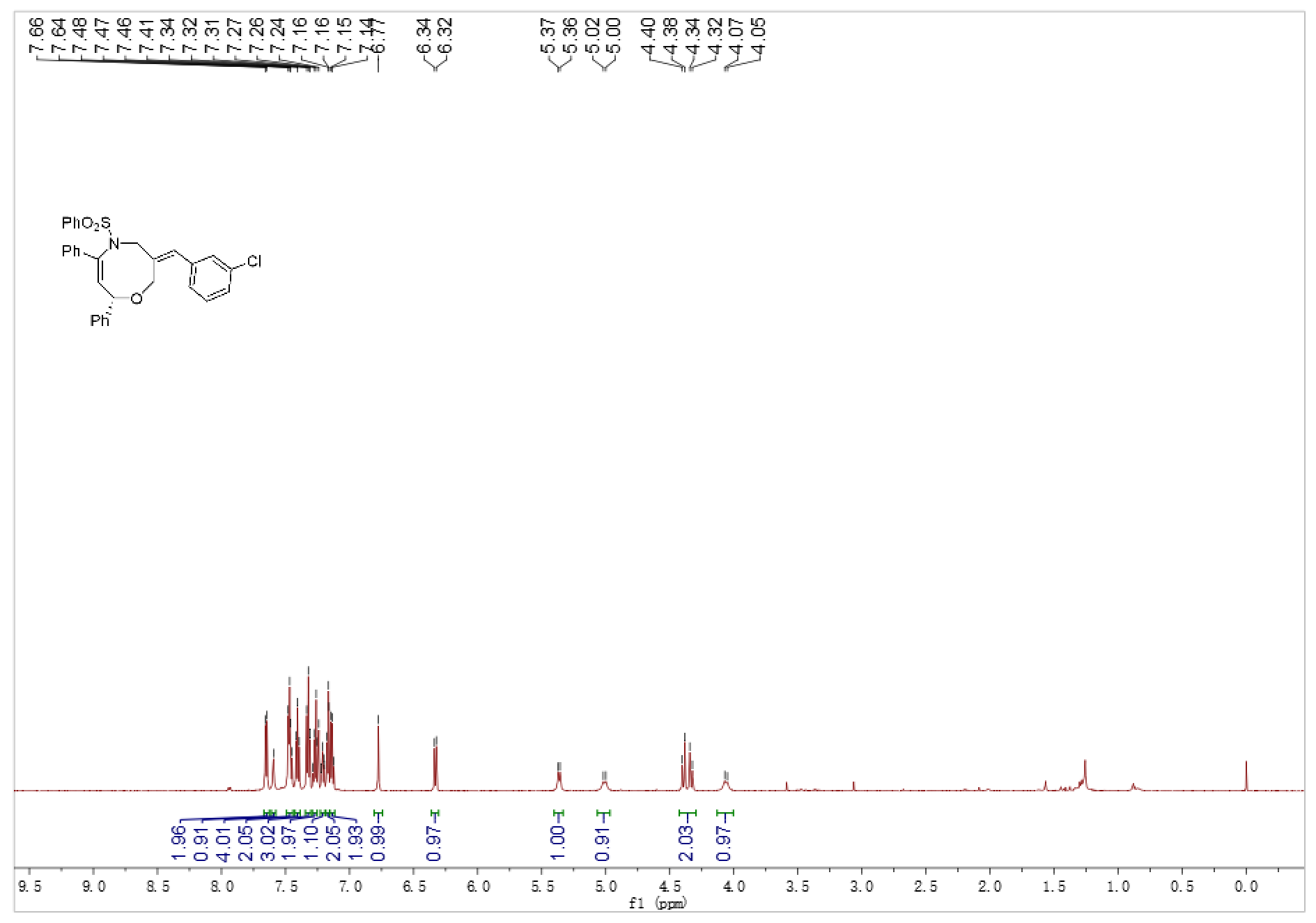
**

^1^H (CDCl_3_, 600 MHz) NMR of compound **10**

**
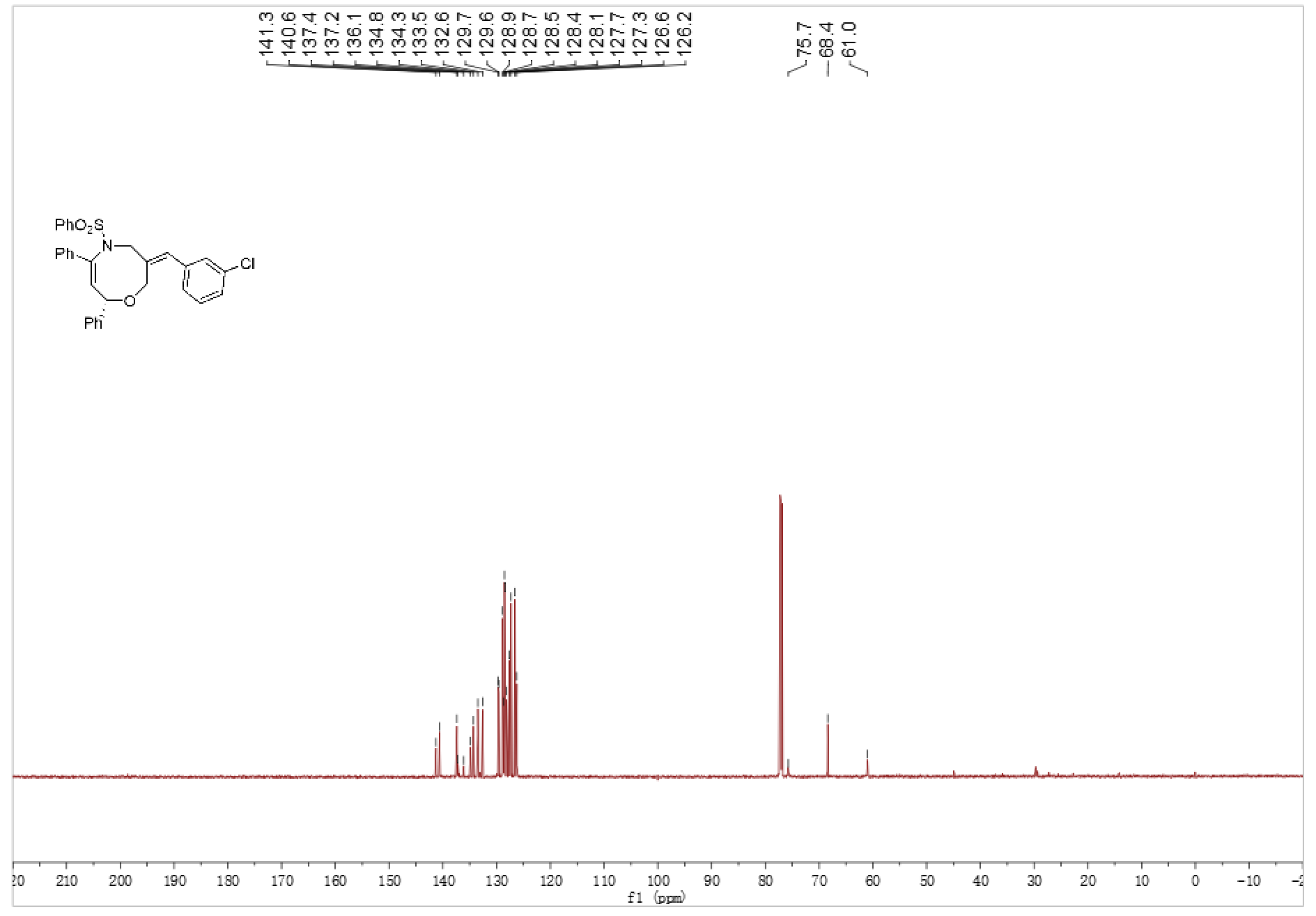
**

^13^C (CDCl_3_, 151 MHz) NMR of compound **10**

**
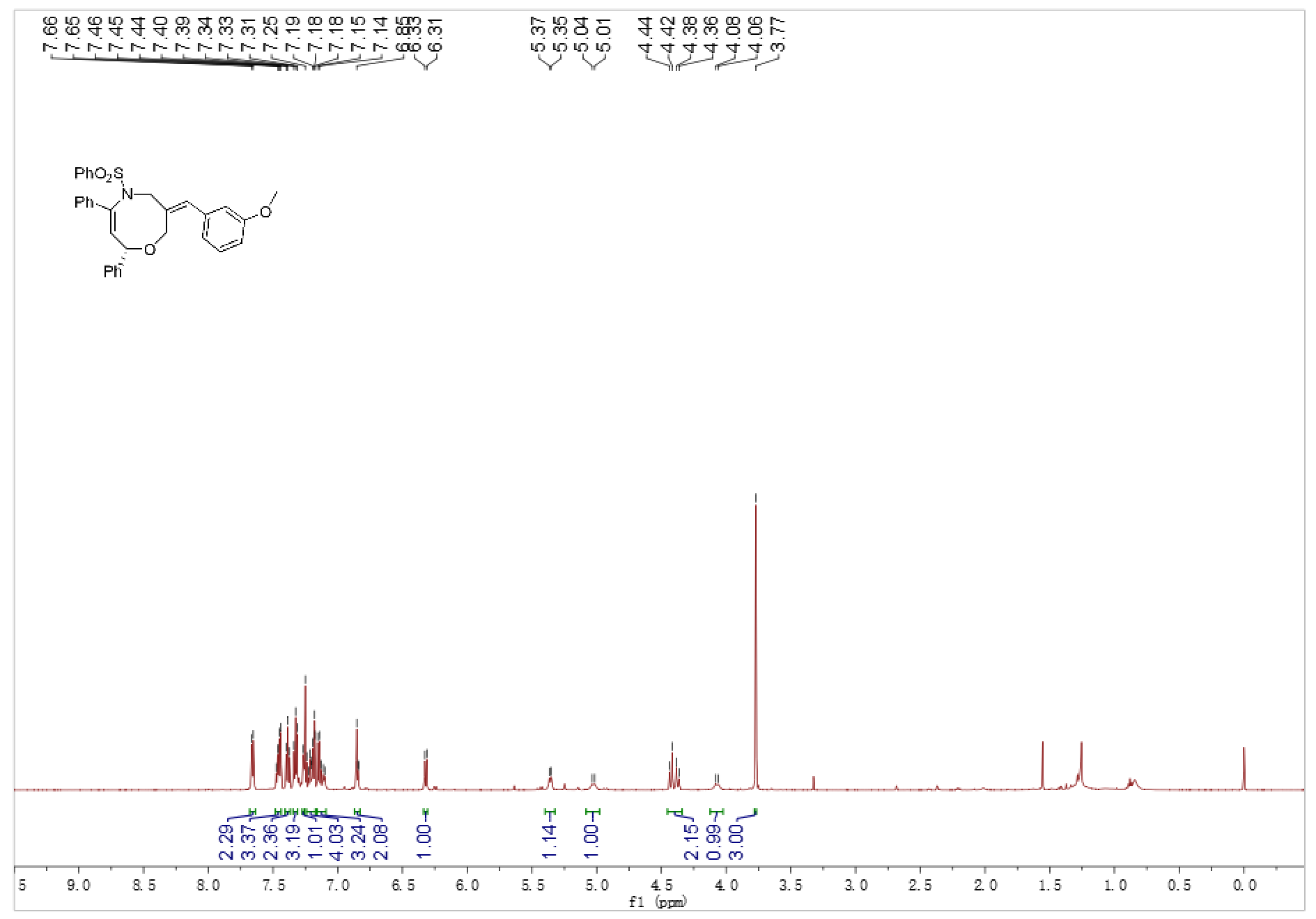
**

^1^H (CDCl_3_, 600 MHz) NMR of compound **11**

**
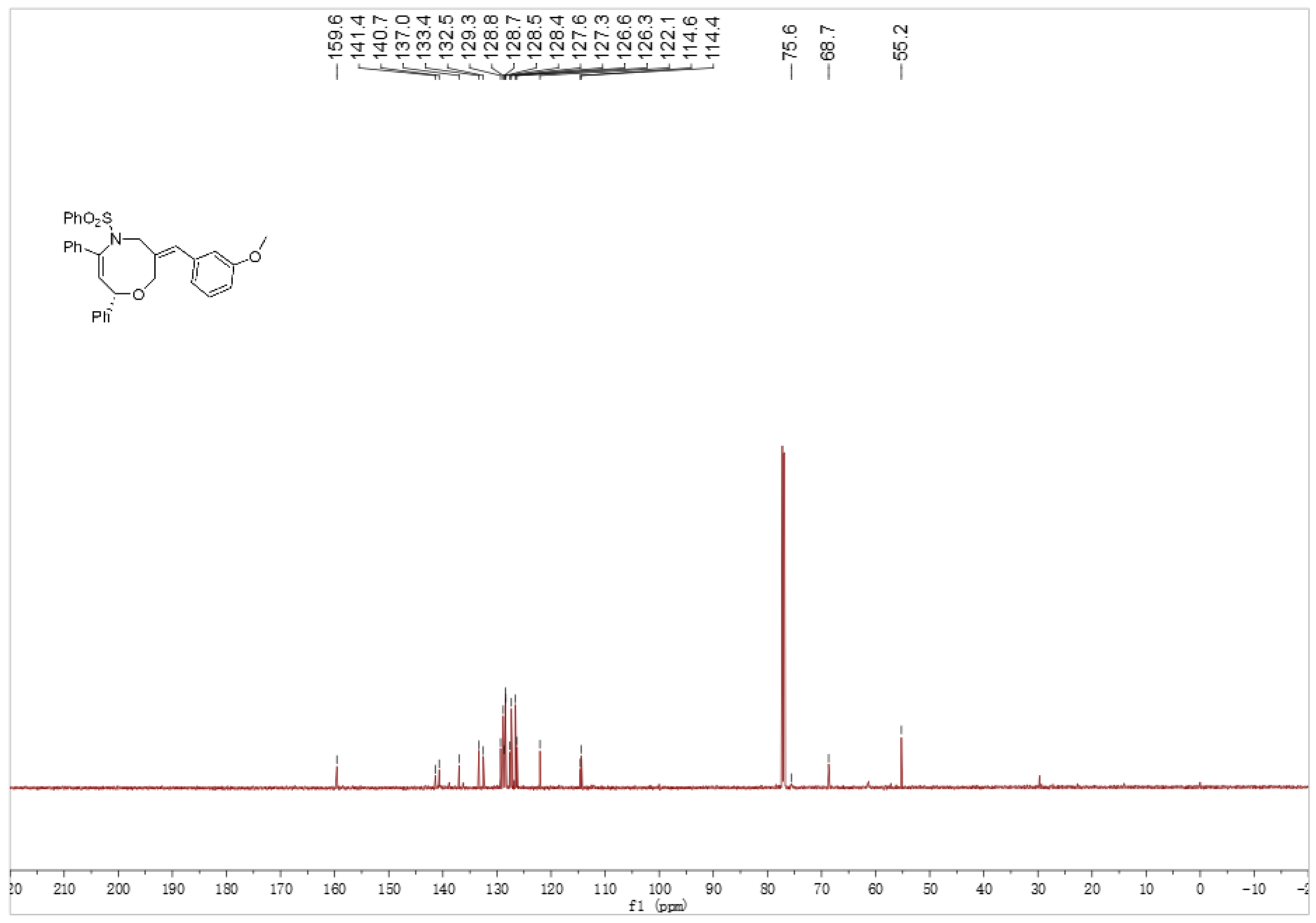
**

^13^C (CDCl_3_, 151 MHz) NMR of compound **11**

**
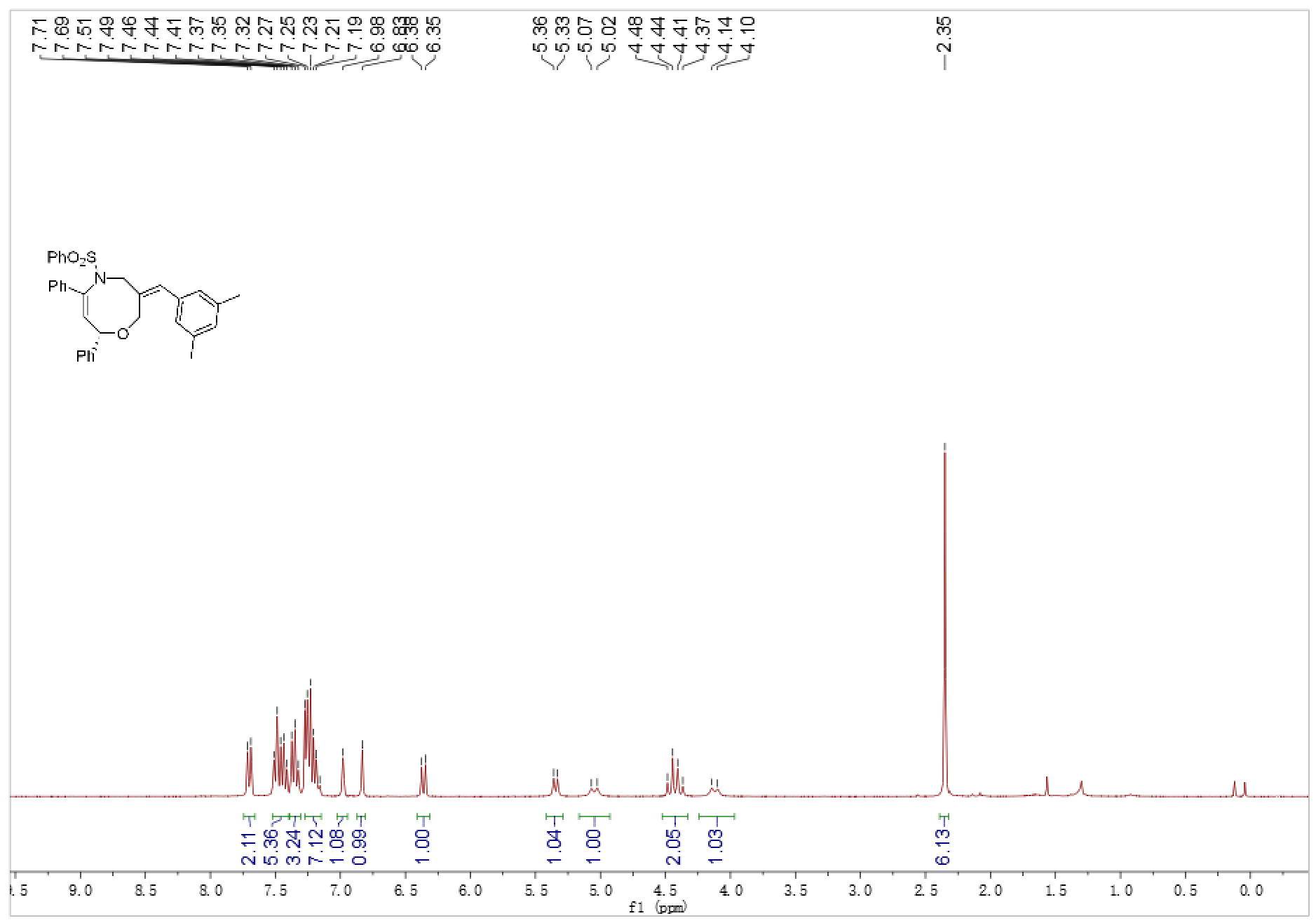
**

^1^H (CDCl_3_, 300 MHz) NMR of compound **12**

**
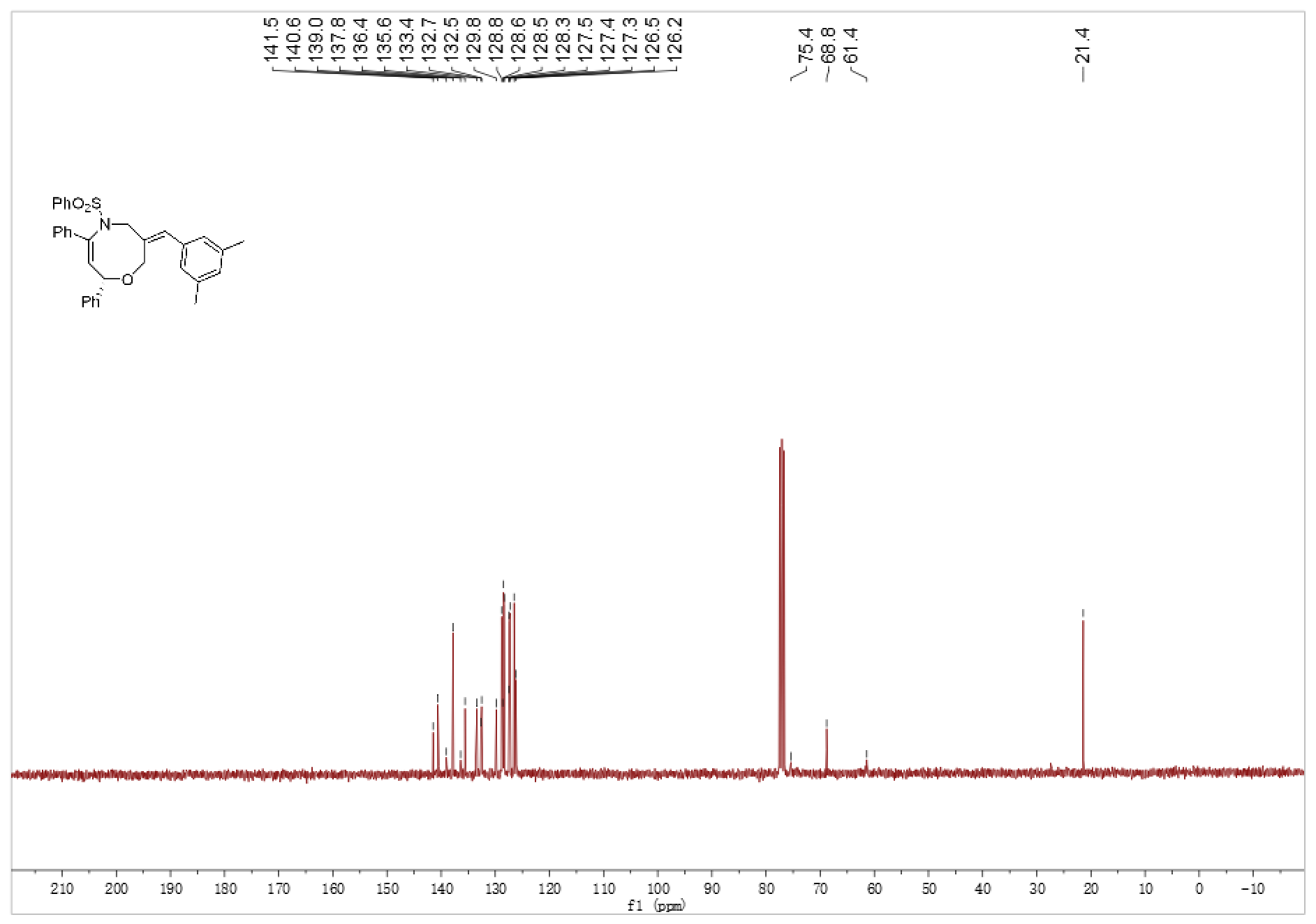
**

^13^C (CDCl_3_, 75 MHz) NMR of compound **12**

**
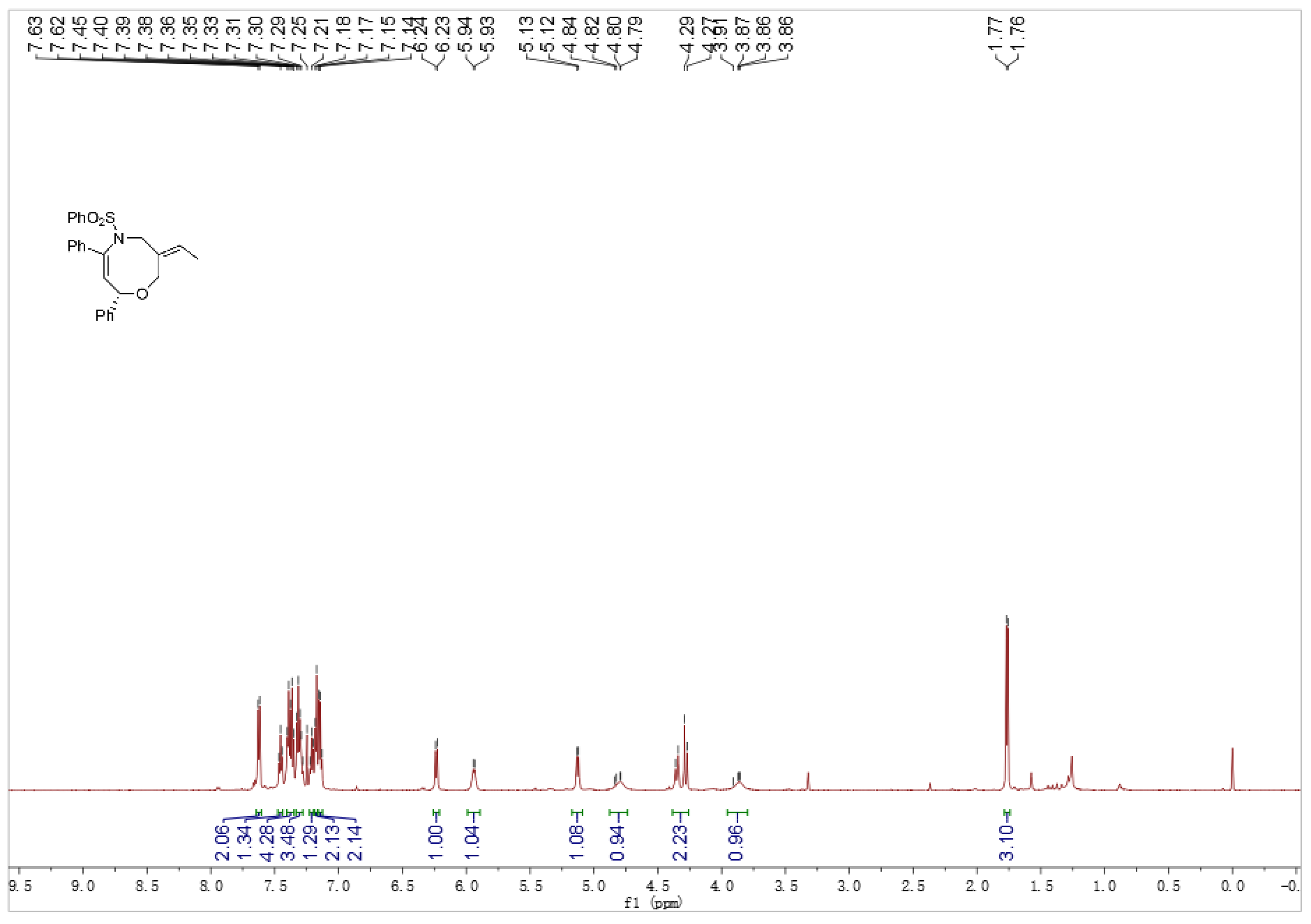
**

^1^H (CDCl_3_, 600 MHz) NMR of compound **13**

**
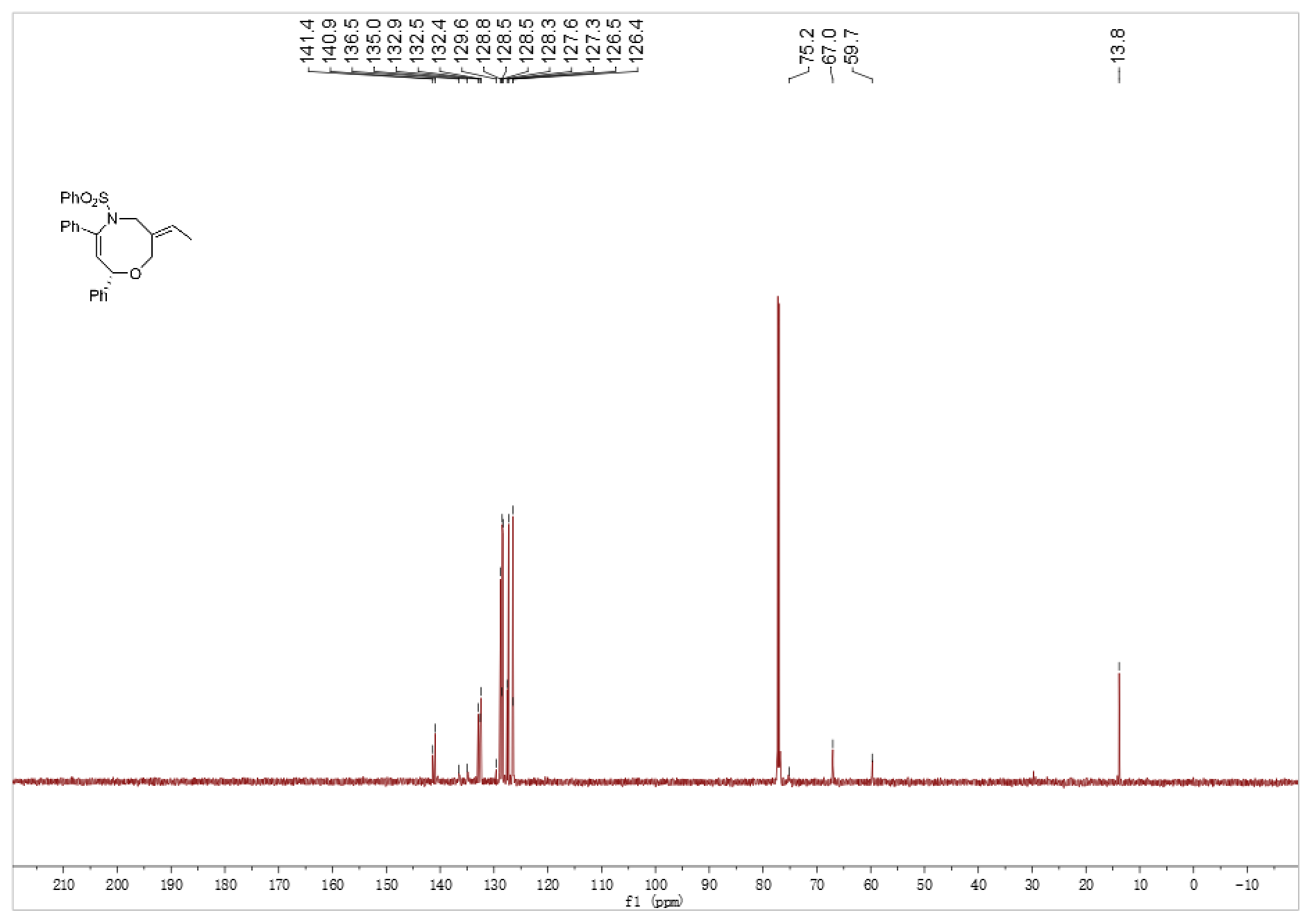
**

^13^C (CDCl_3_, 151 MHz) NMR of compound **13**

**
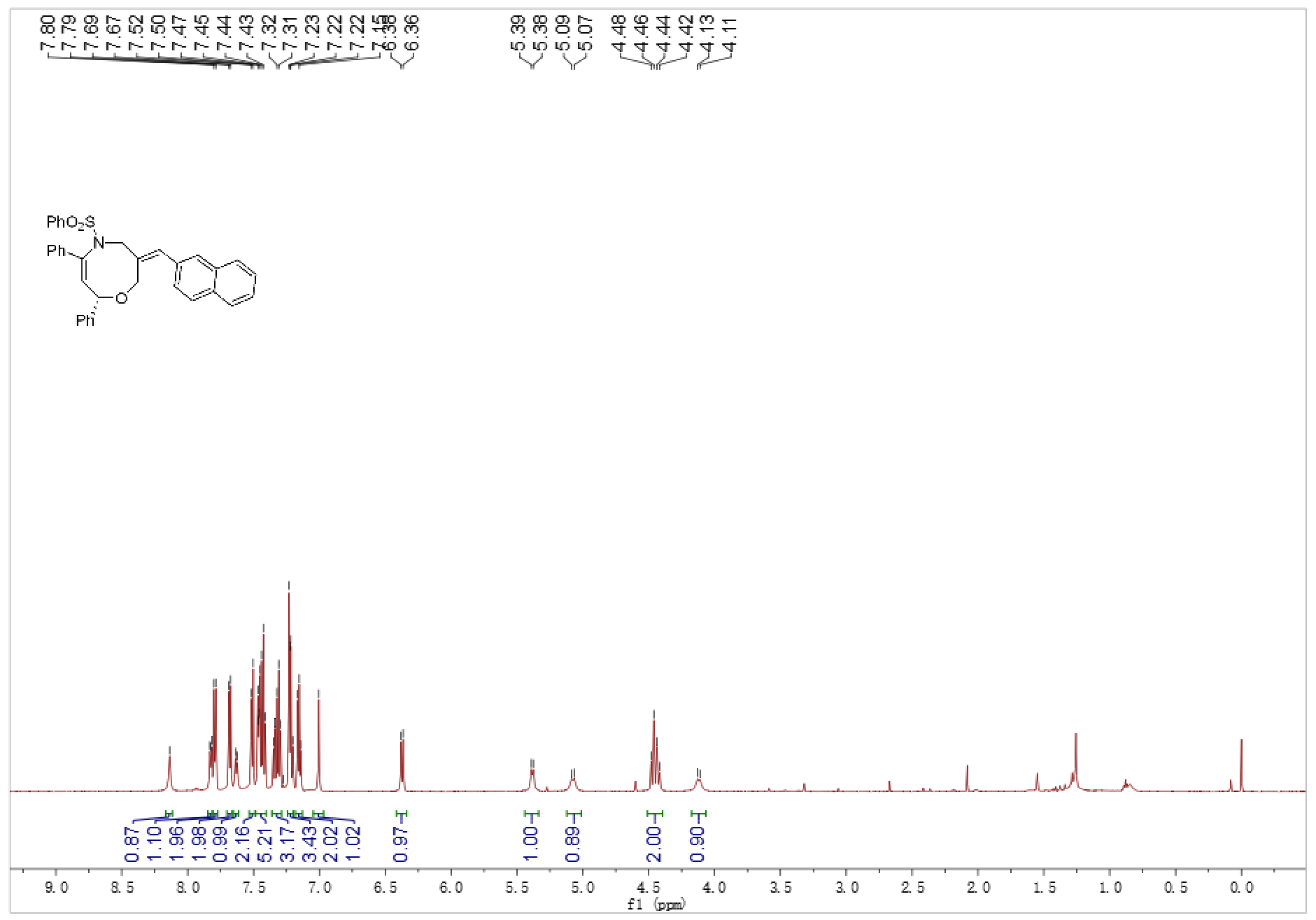
**

^1^H (CDCl_3_, 600 MHz) NMR of compound **14**

**
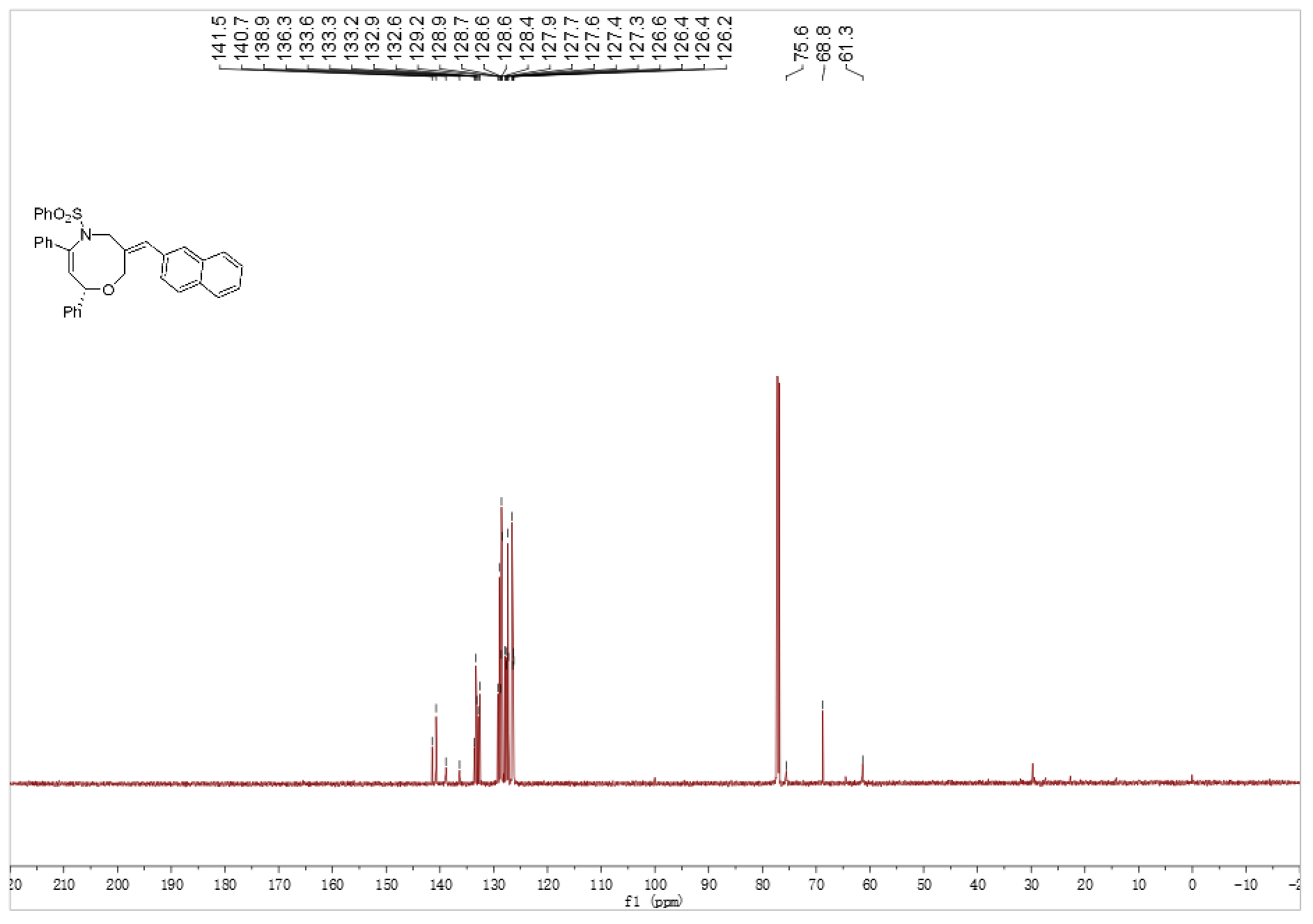
**

^13^C (CDCl_3_, 151 MHz) NMR of compound **14**

**
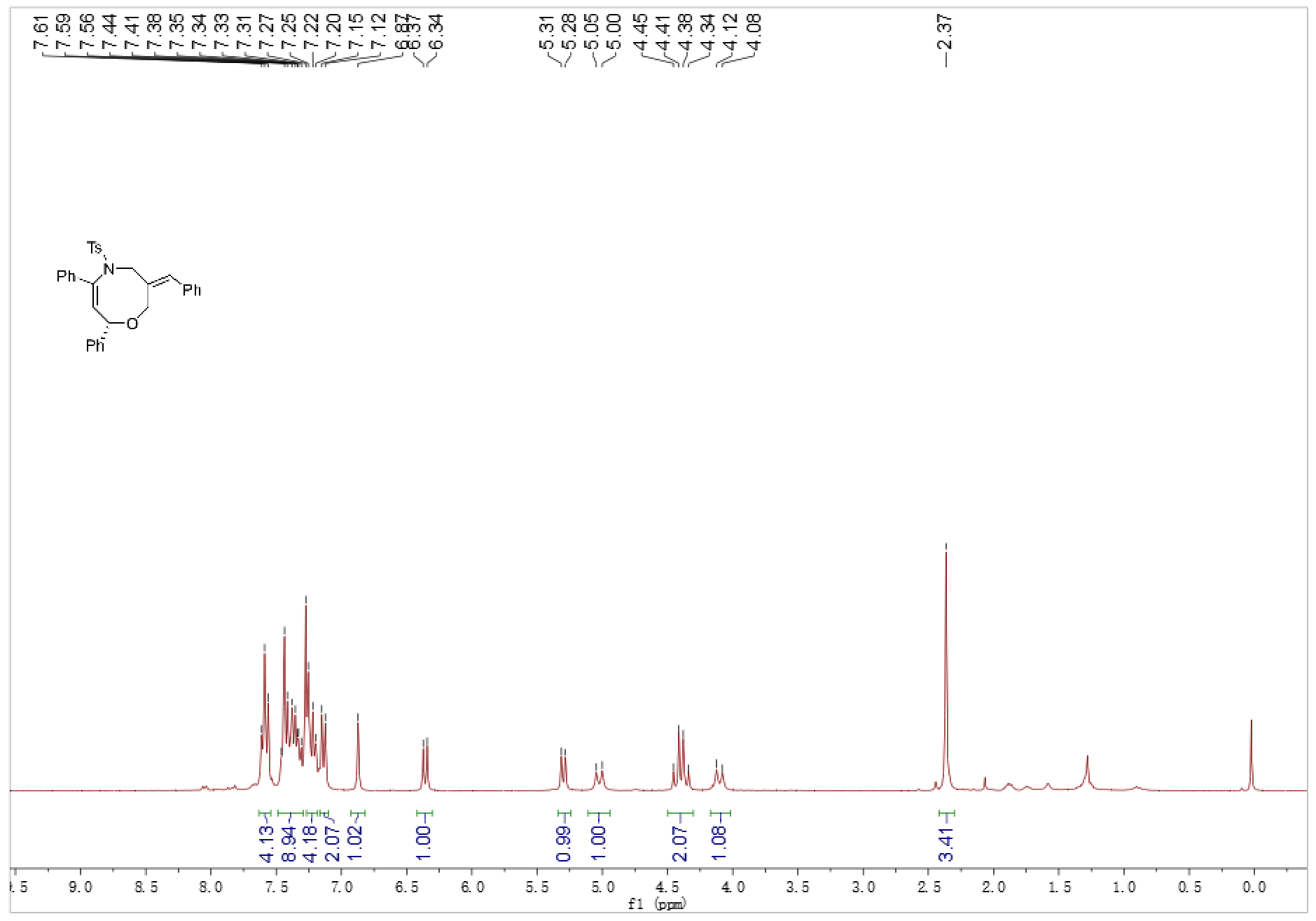
**

^1^H (CDCl_3_, 300 MHz) NMR of compound **15**

**
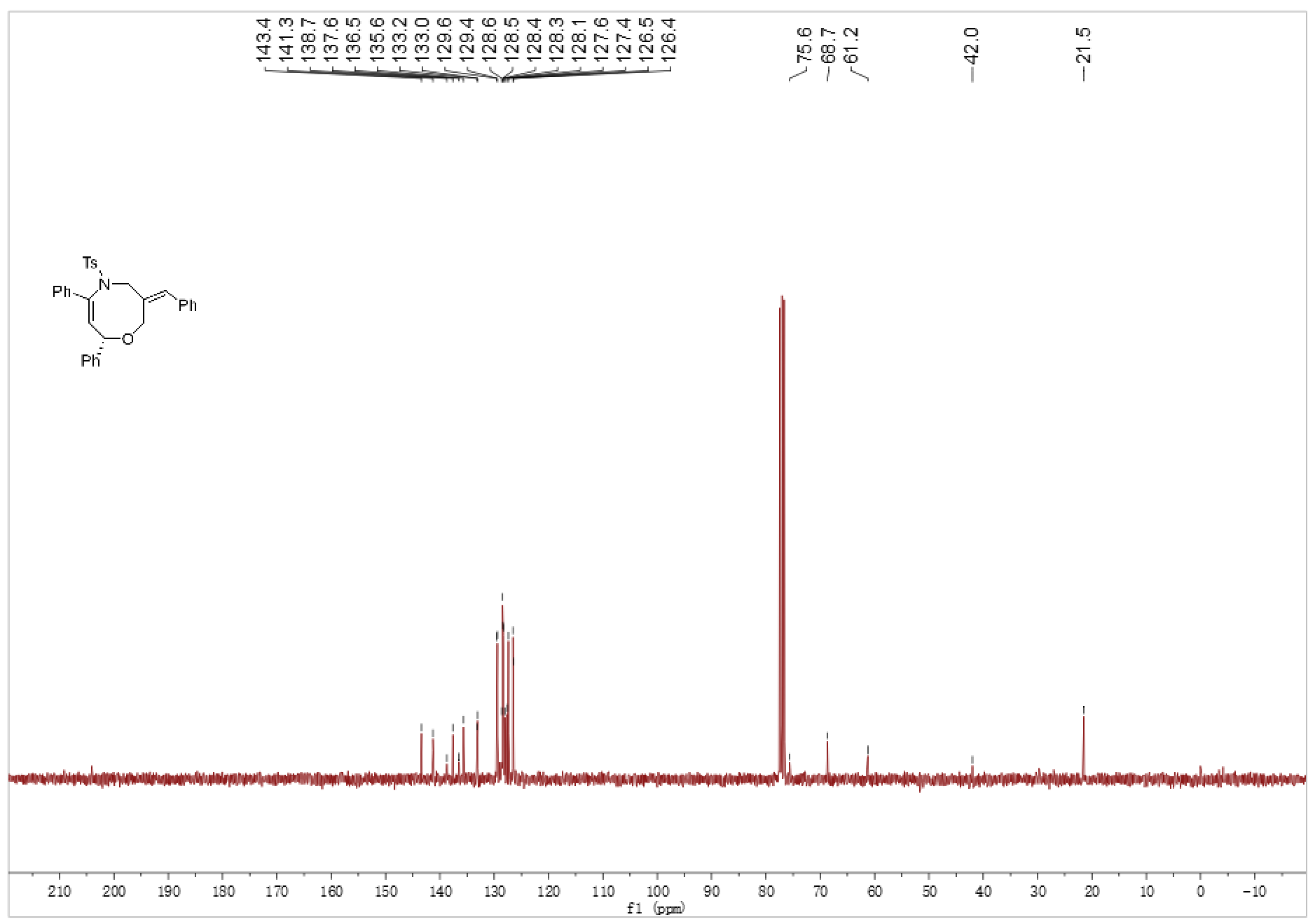
**

^13^C (CDCl_3_, 75 MHz) NMR of compound **15**

**
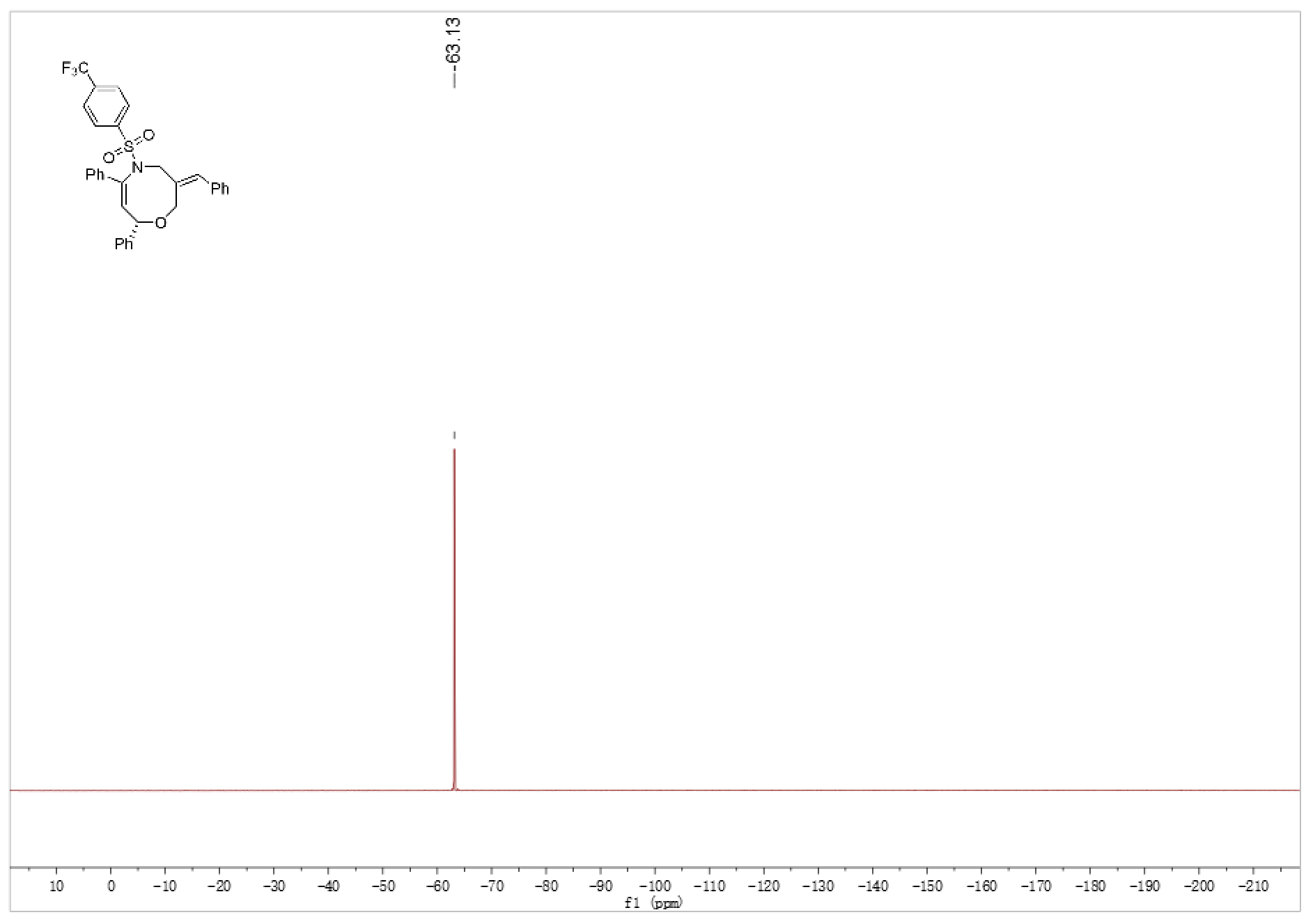
**

^19^F (CDCl_3_, 282 MHz) NMR of compound **16**

**
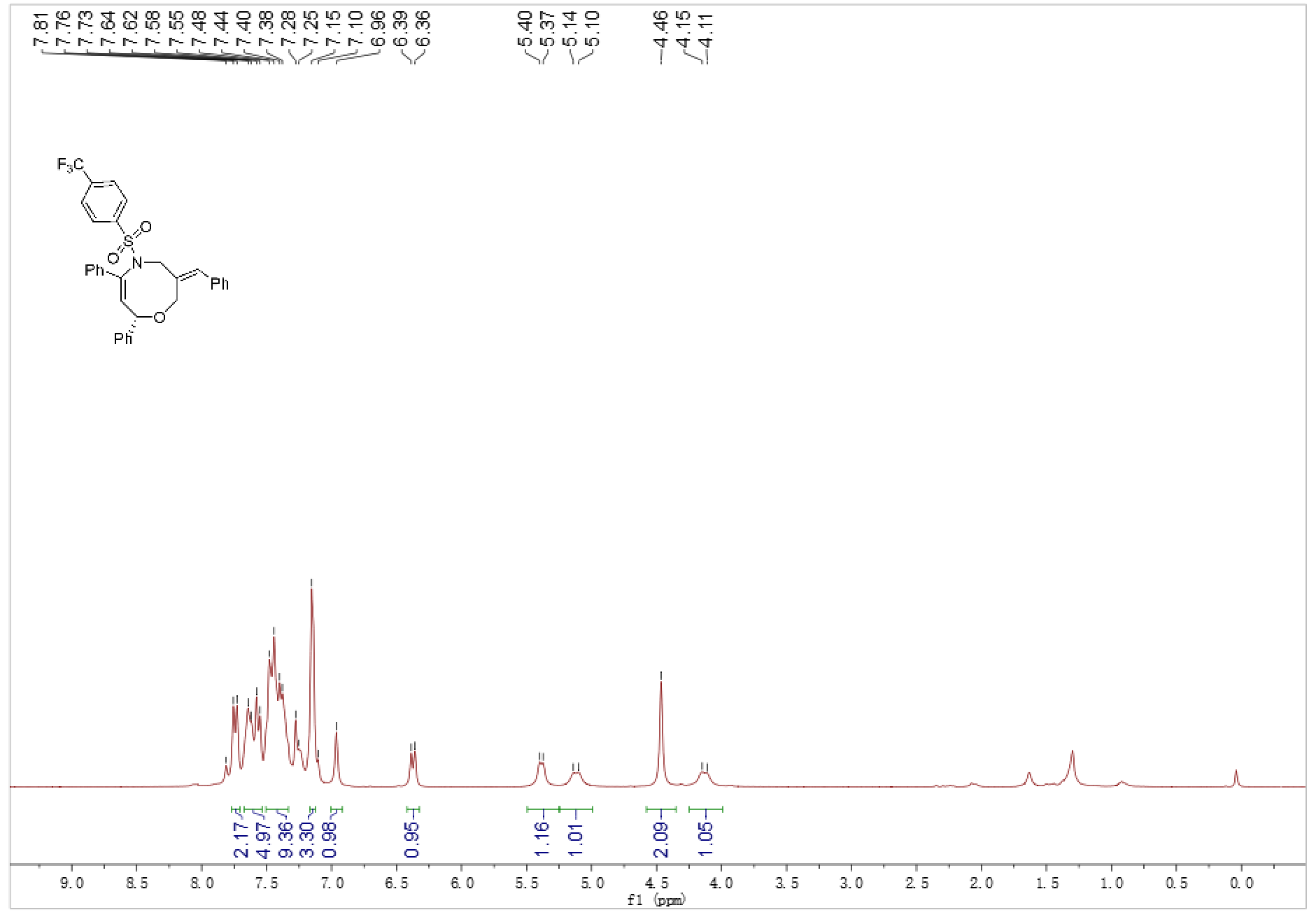
**

^1^H (CDCl_3_, 300 MHz) NMR of compound **16**

**
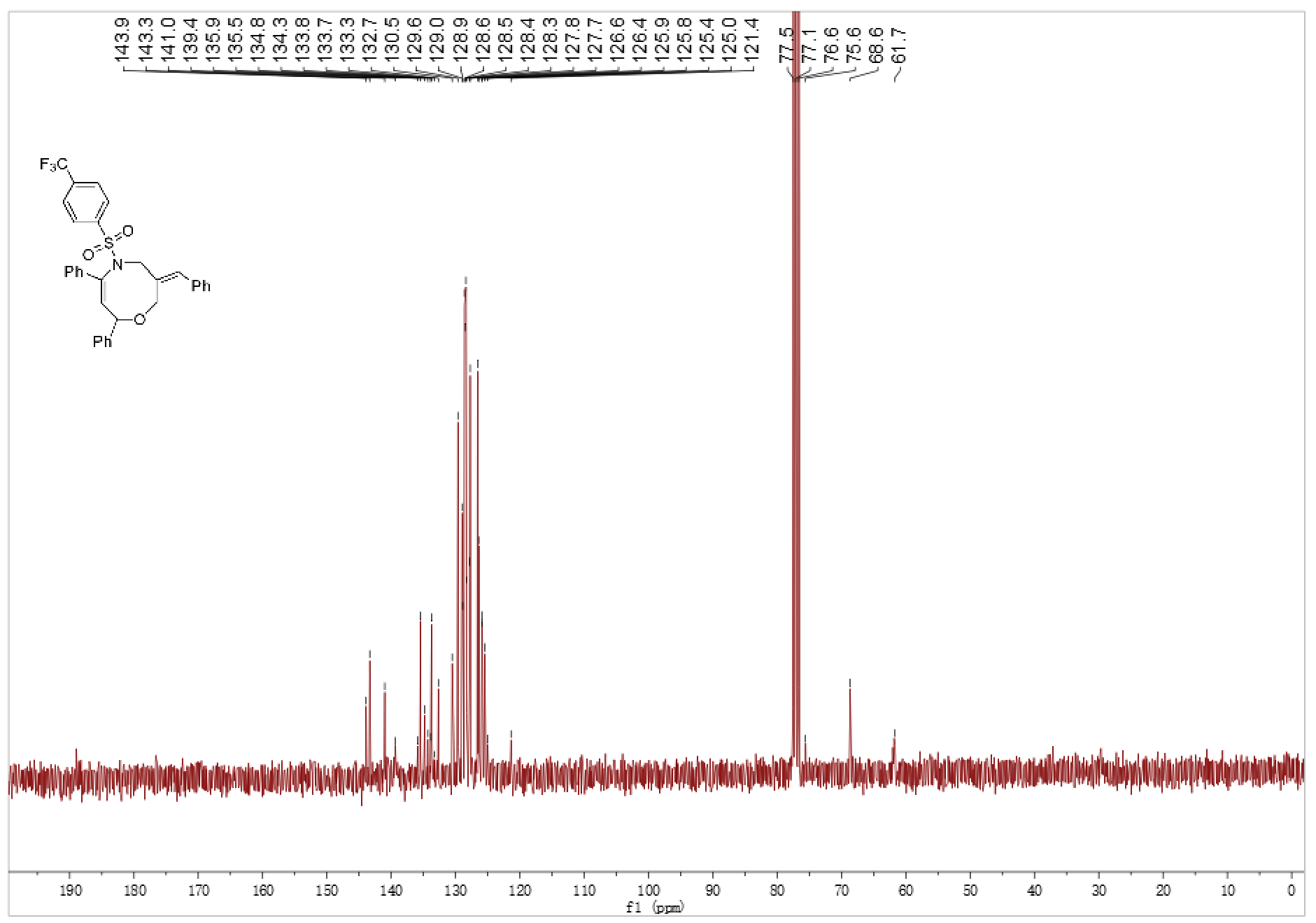
**

^13^C (CDCl_3_, 75 MHz) NMR of compound **16**

**
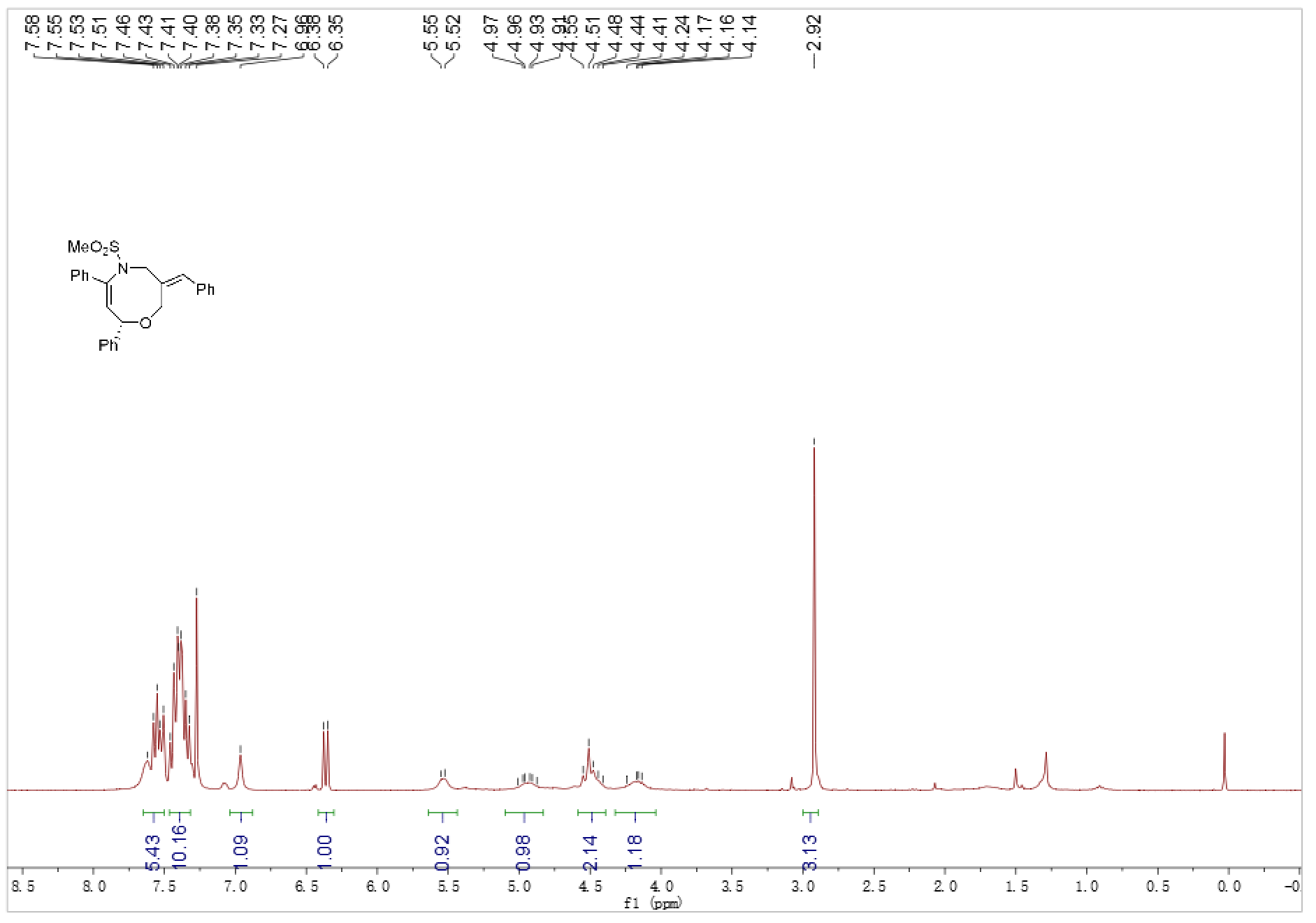
**

^1^H (CDCl_3_, 300 MHz) NMR of compound **17**


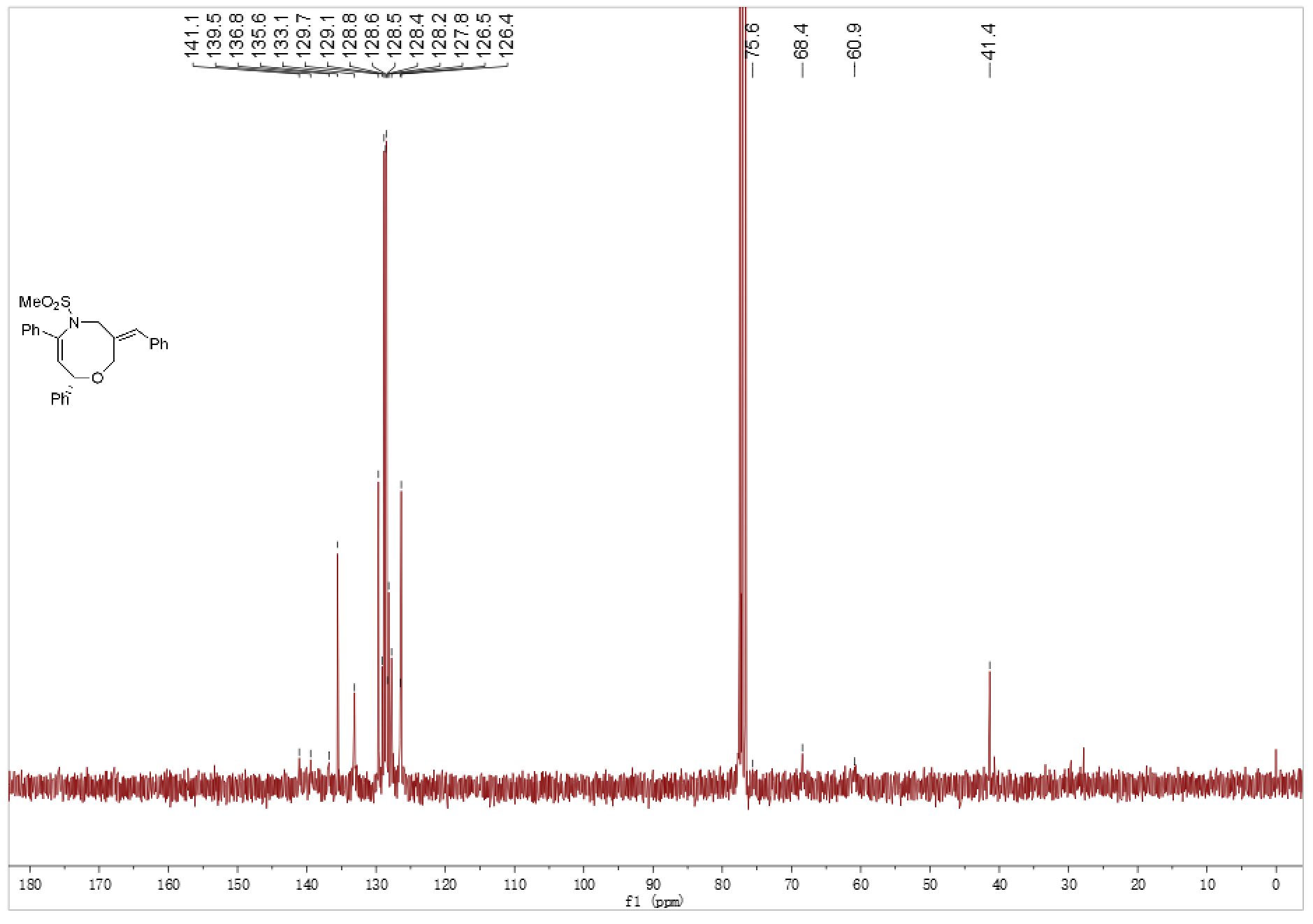


^13^C (CDCl_3_, 75 MHz) NMR of compound **17**

**
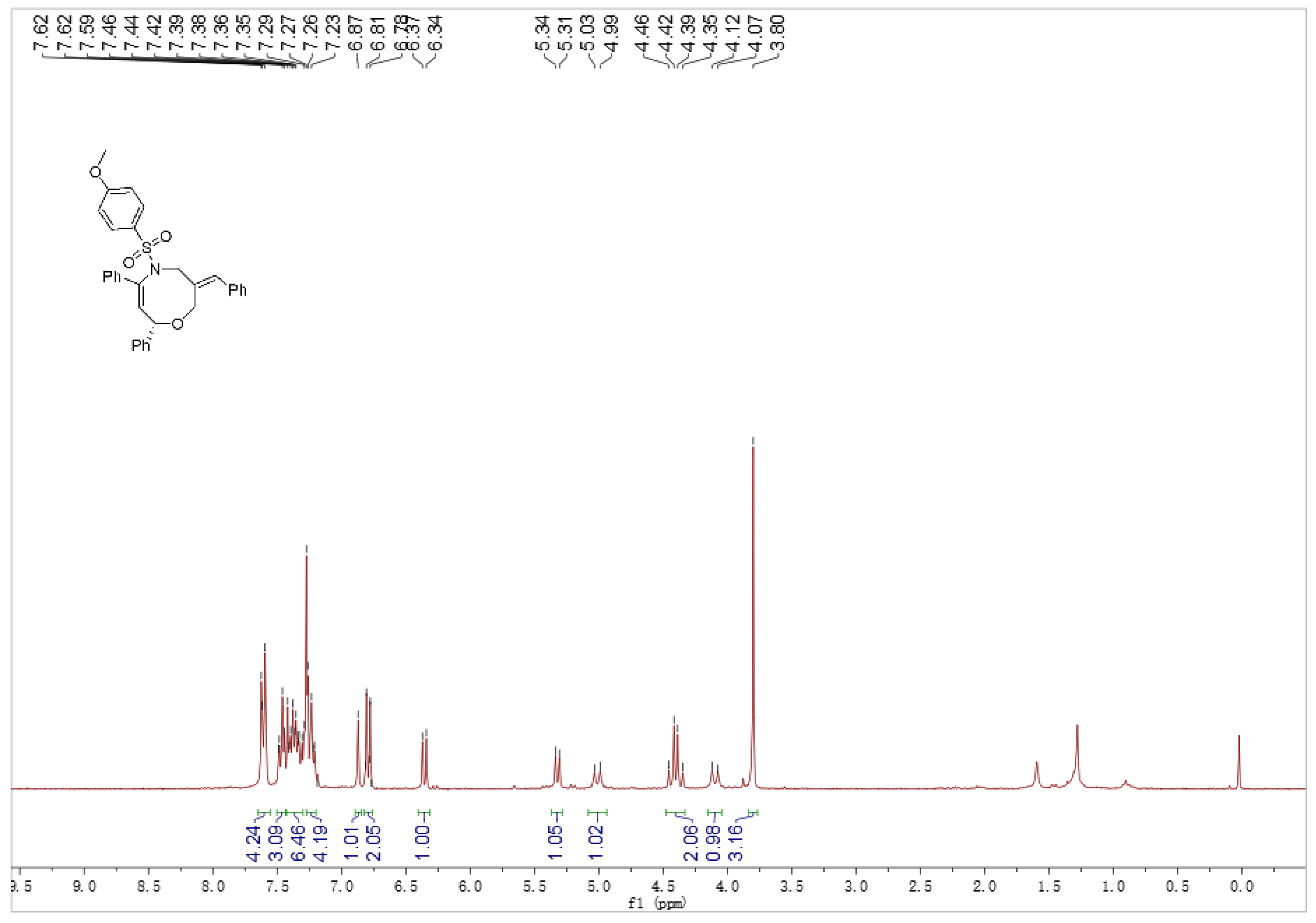
**

^1^H (CDCl_3_, 300 MHz) NMR of compound **18**

**
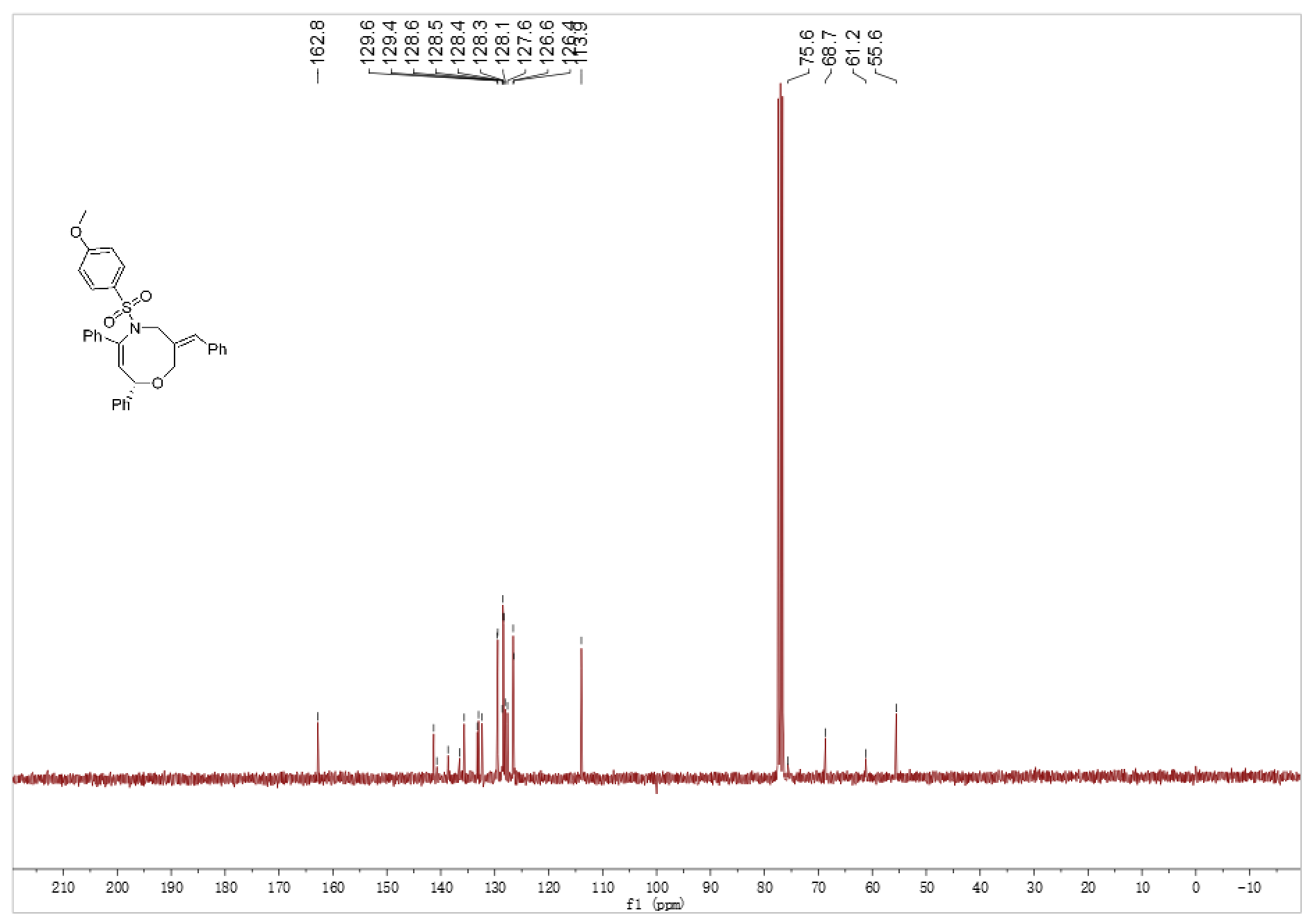
**

^13^C (CDCl_3_, 75 MHz) NMR of compound **18**

**
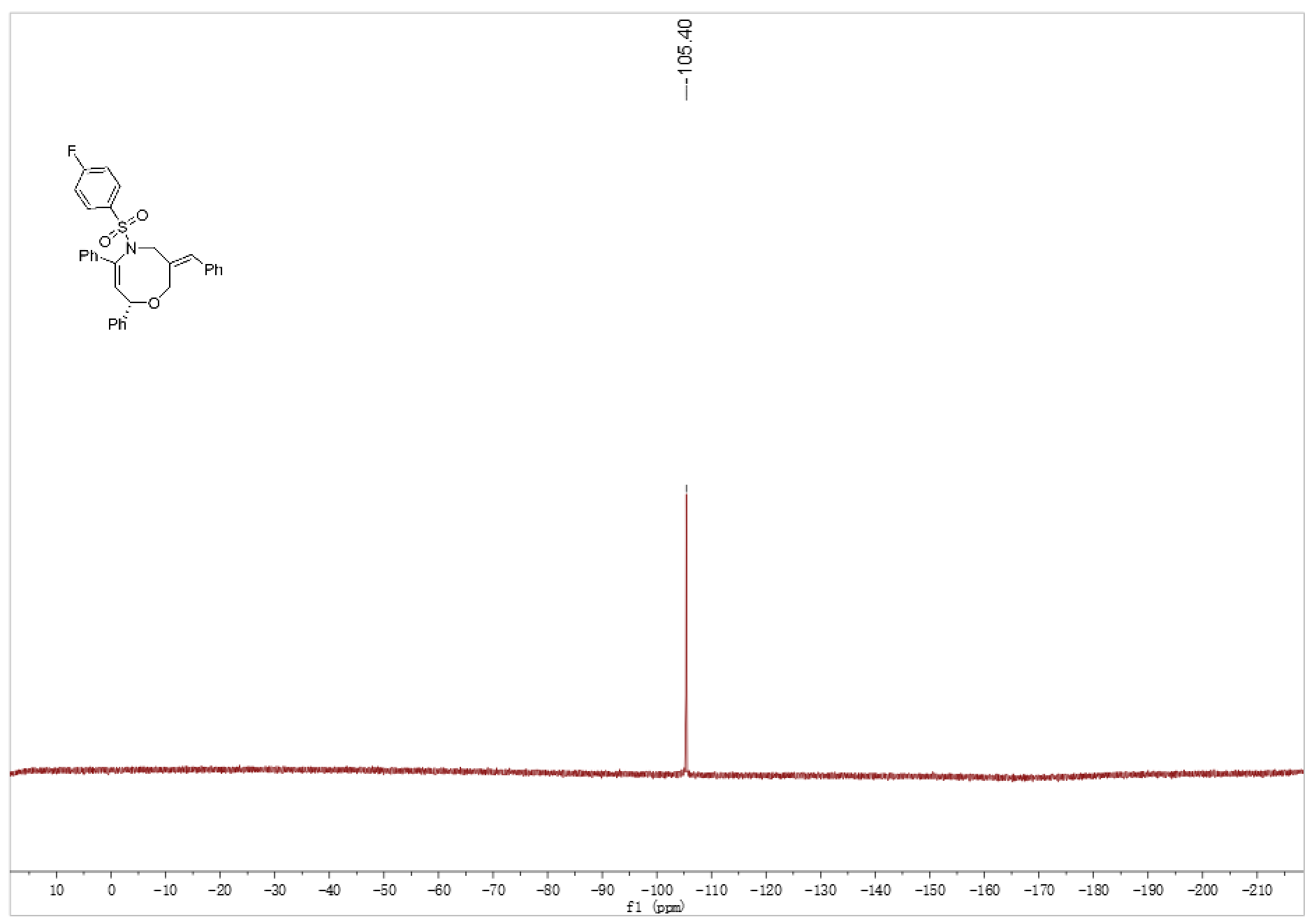
**

^19^F (CDCl_3_, 282 MHz) NMR of compound **19**

**
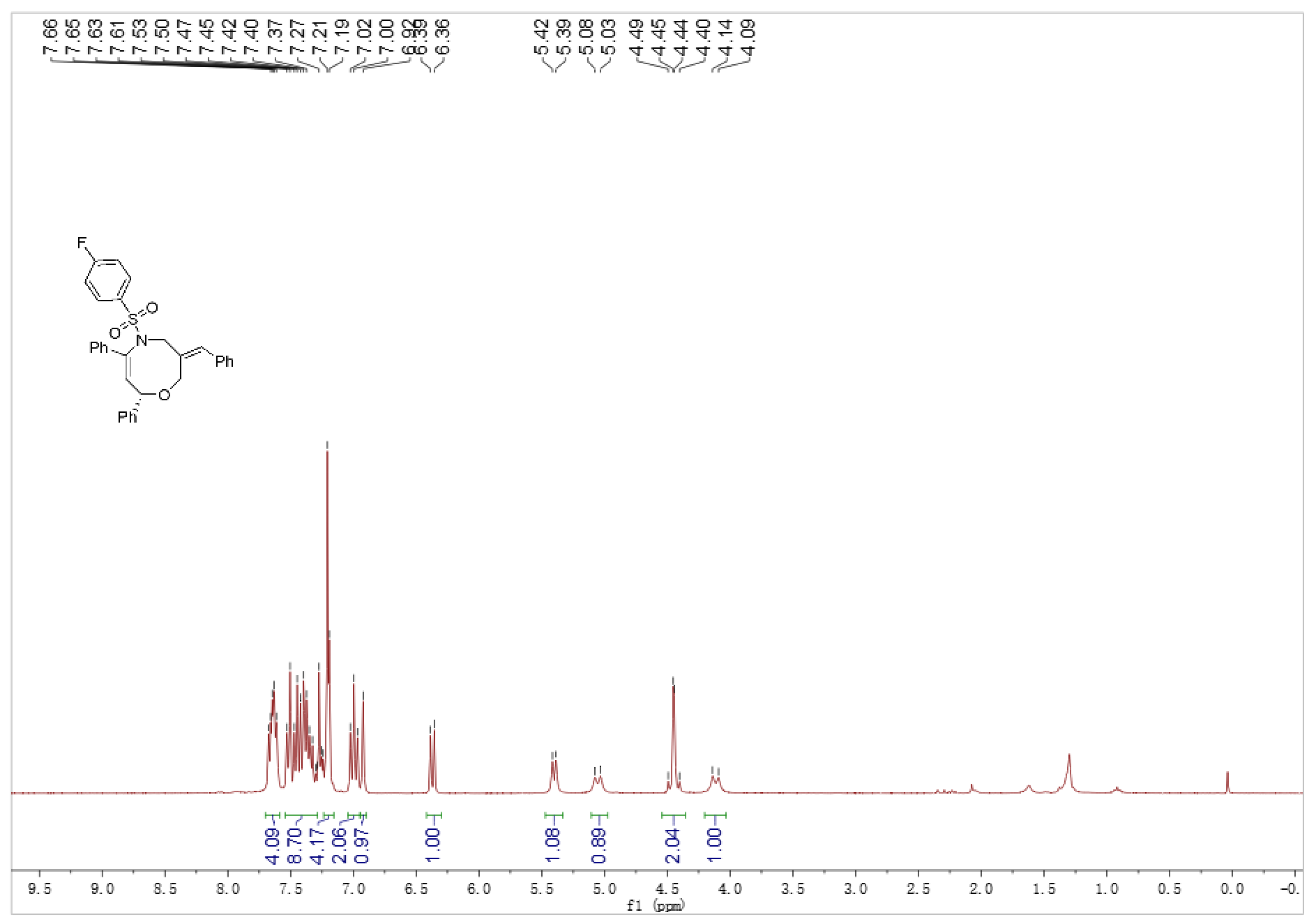
**

^1^H (CDCl_3_, 300 MHz) NMR of compound **19**

**
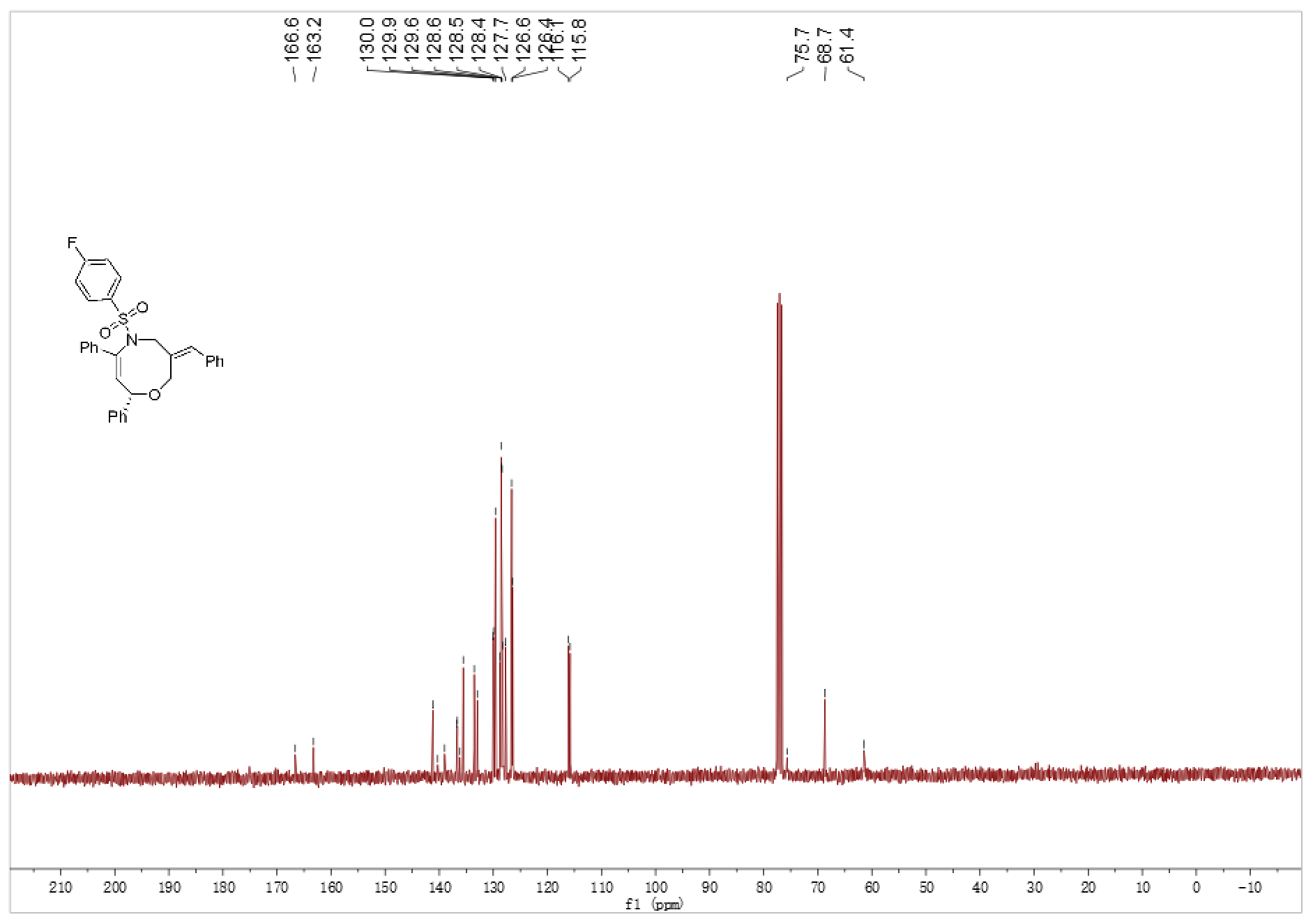
**

^13^C (CDCl_3_, 75 MHz) NMR of compound **19**

**
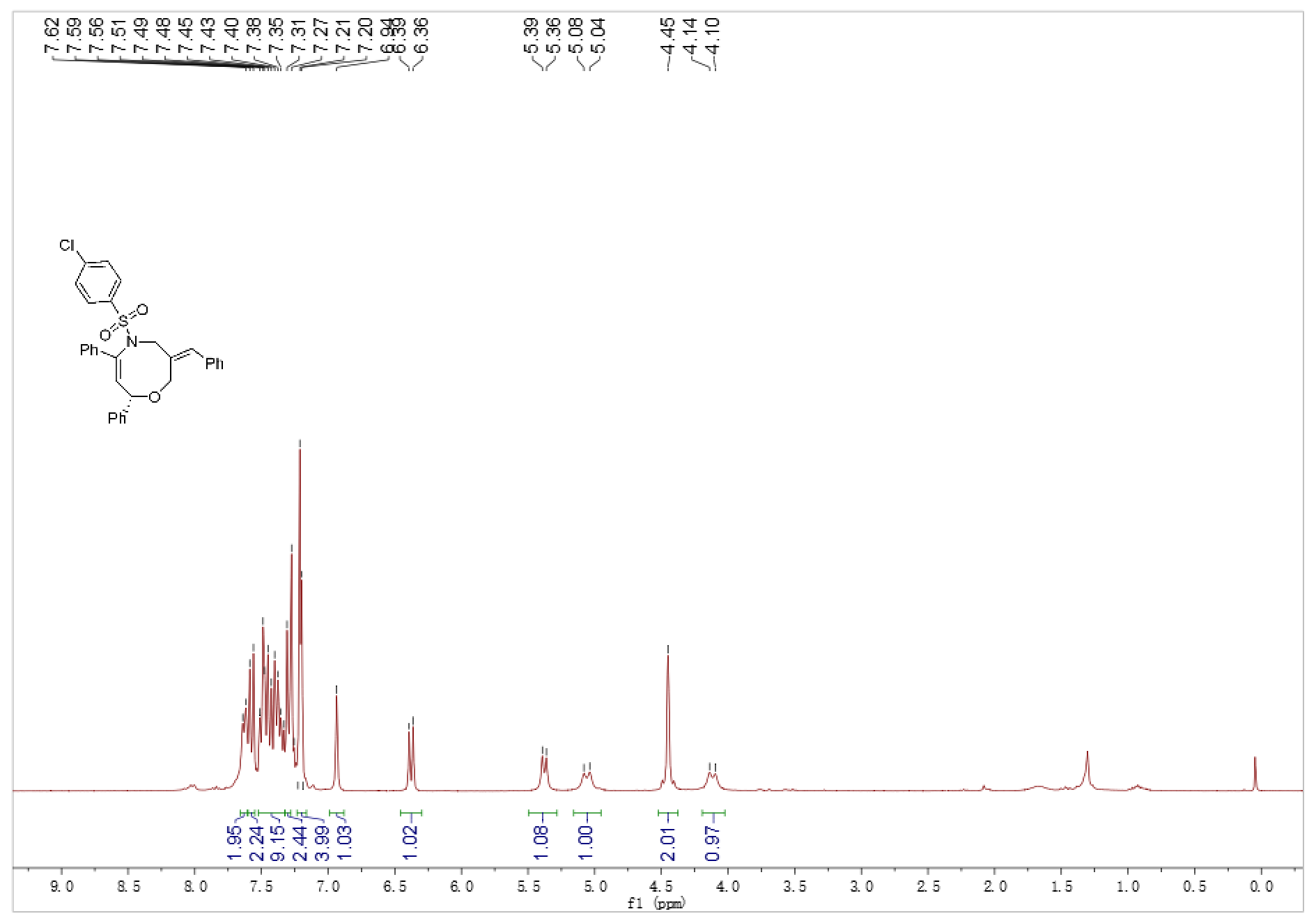
**

^1^H (CDCl_3_, 300 MHz) NMR of compound **20**

**
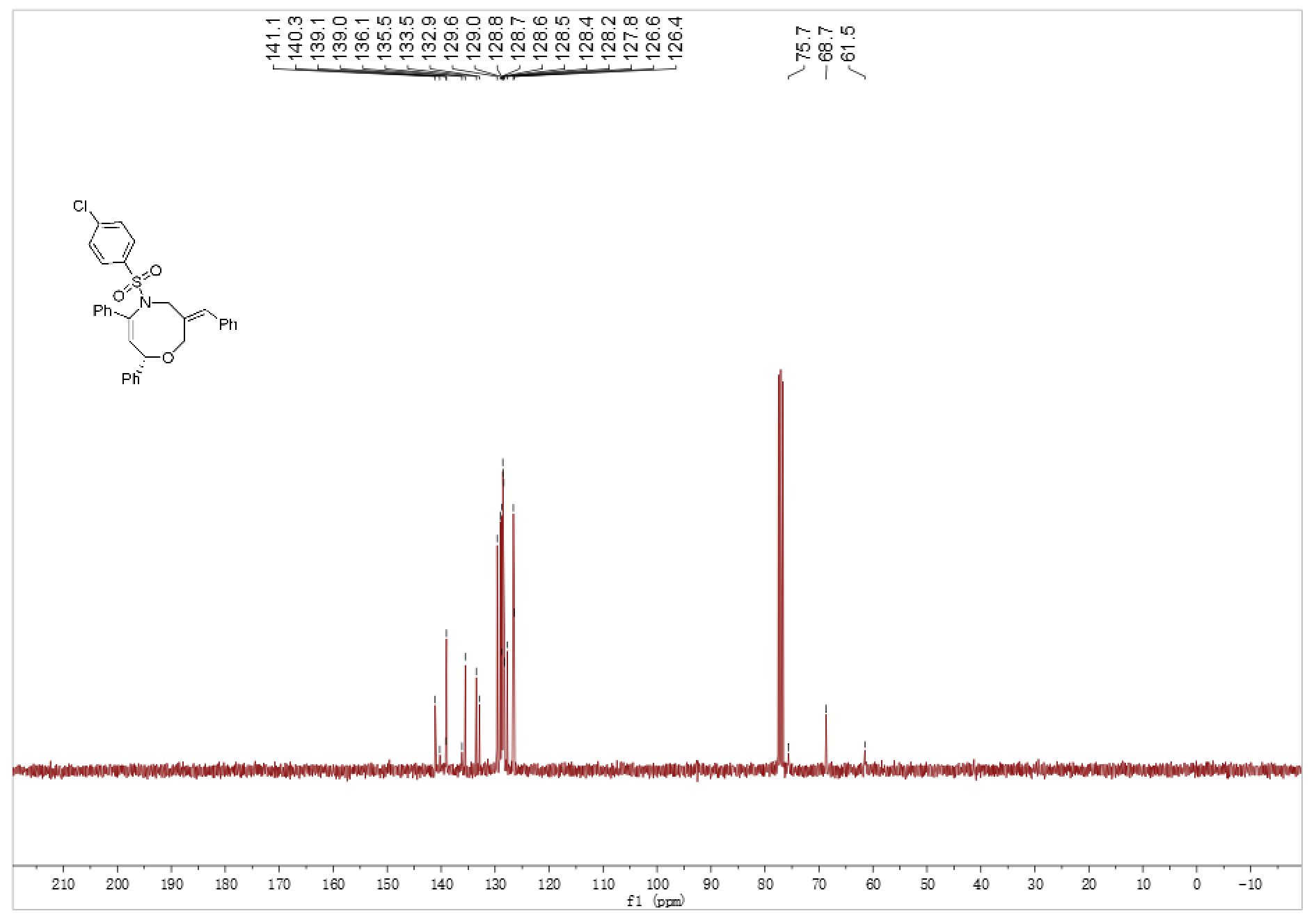
**

^13^C (CDCl_3_, 75 MHz) NMR of compound **20**

**
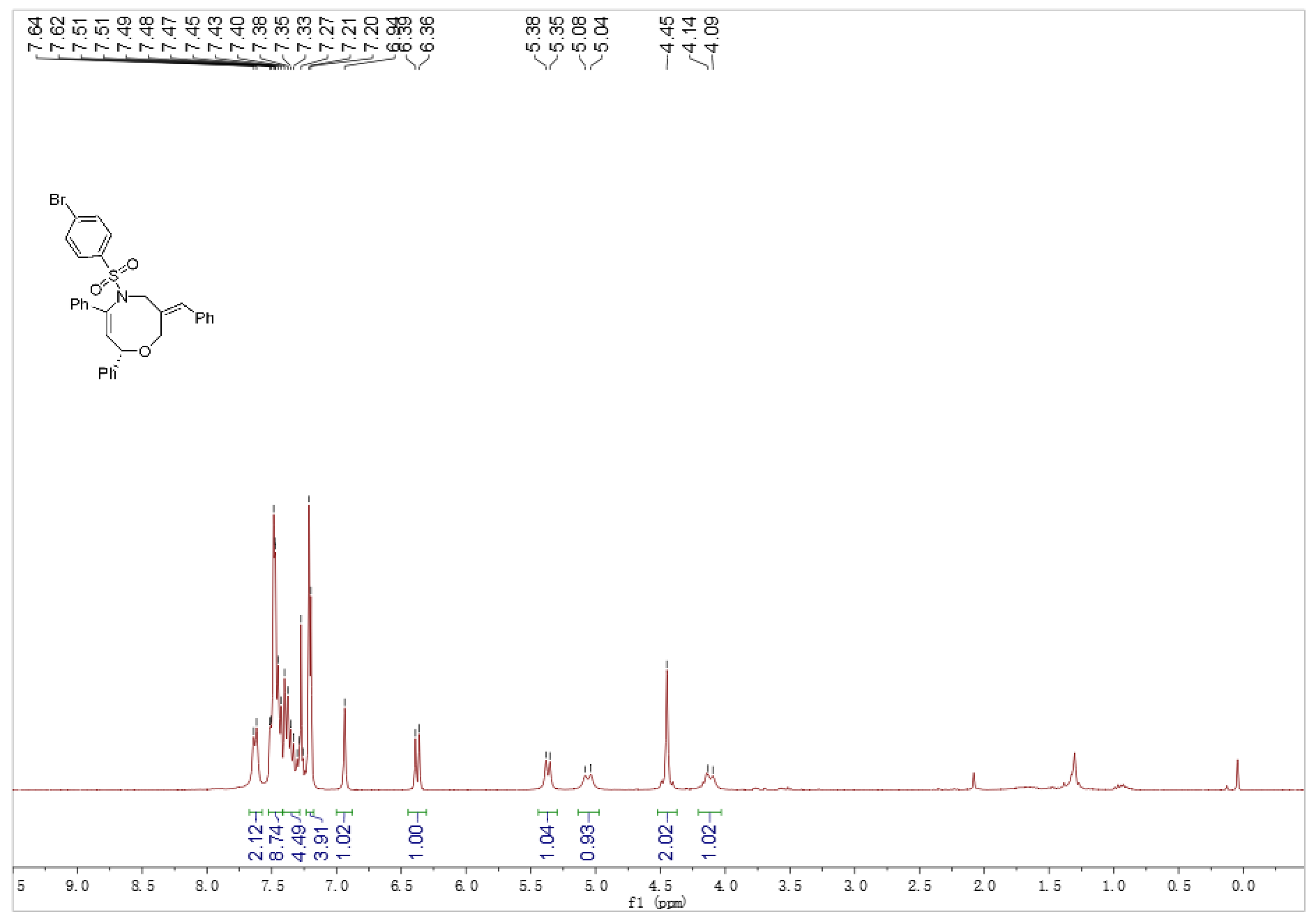
**

^1^H (CDCl_3_, 300 MHz) NMR of compound **21**

**
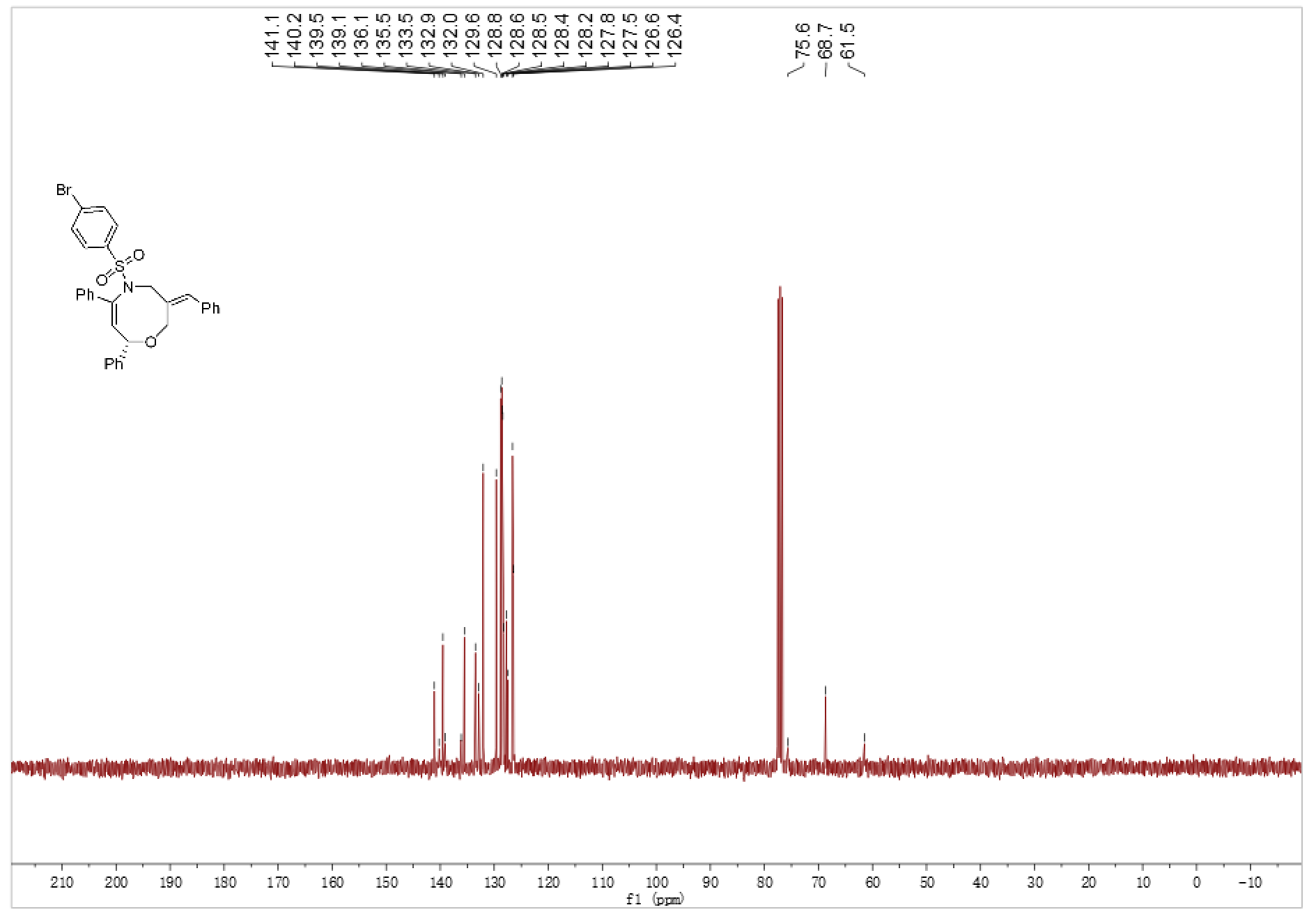
**

^13^C (CDCl_3_, 75 MHz) NMR of compound **21**

**
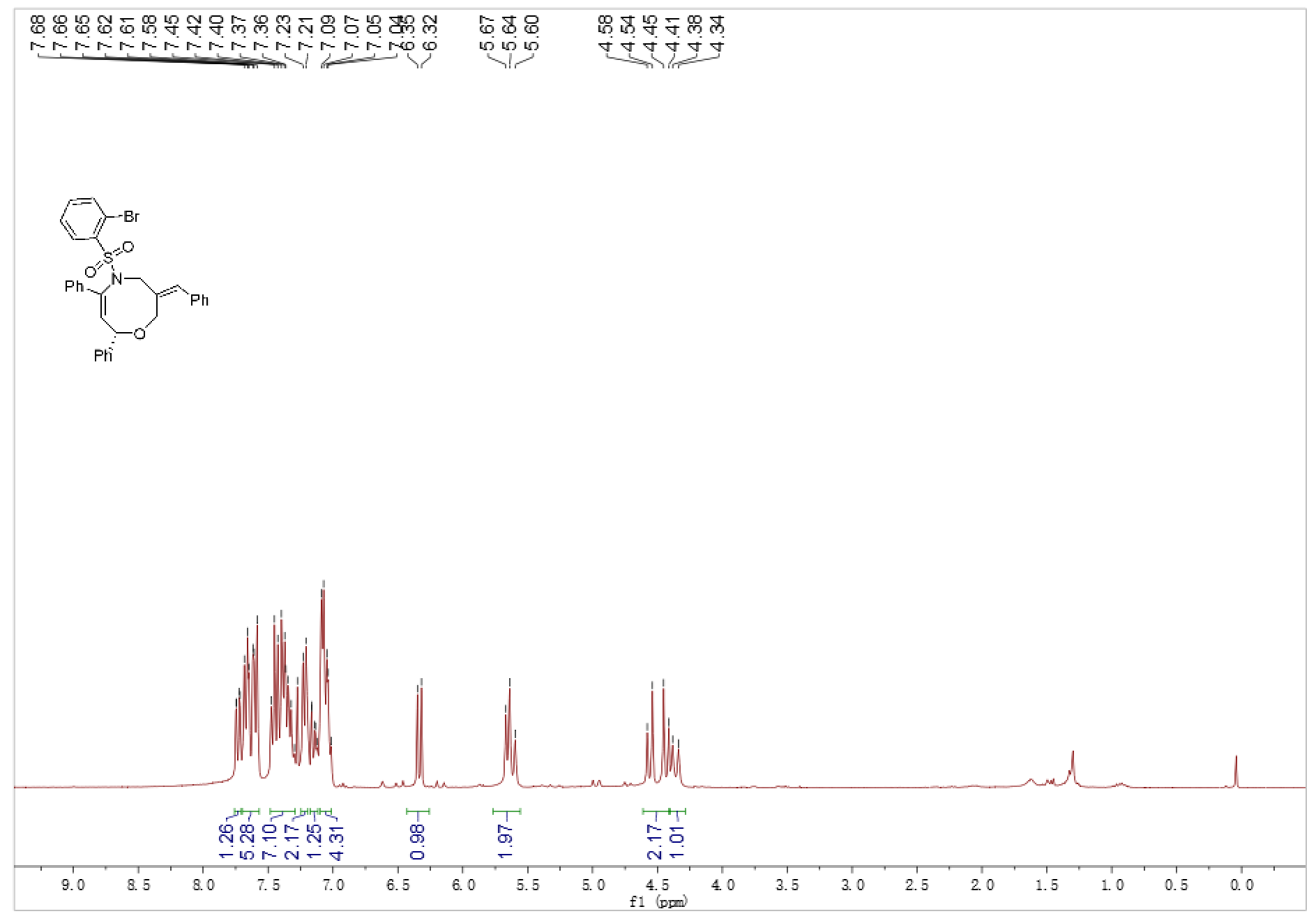
**

^1^H (CDCl_3_, 300 MHz) NMR of compound **22**

**
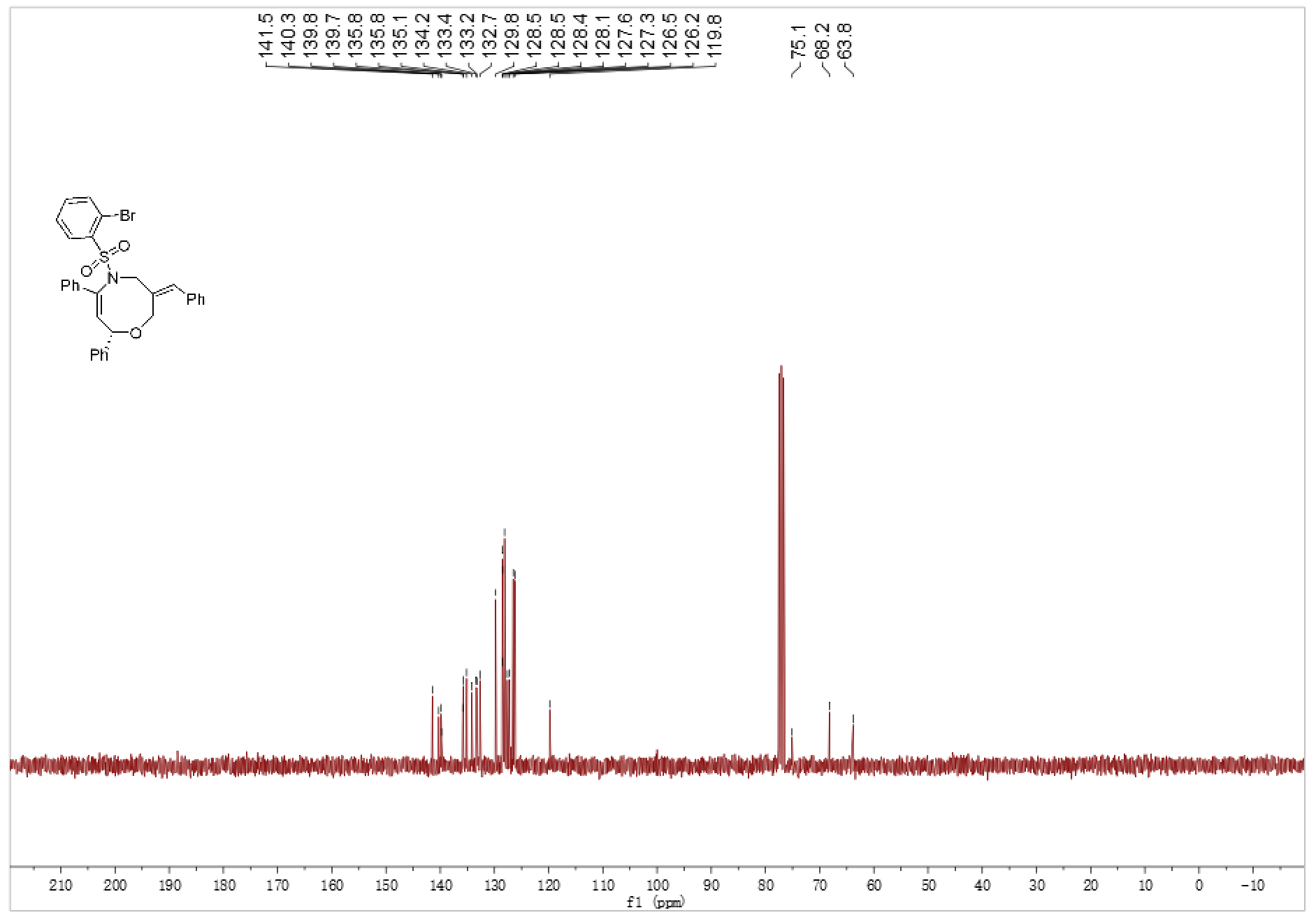
**

^13^C (CDCl_3_, 75 MHz) NMR of compound **22**

**
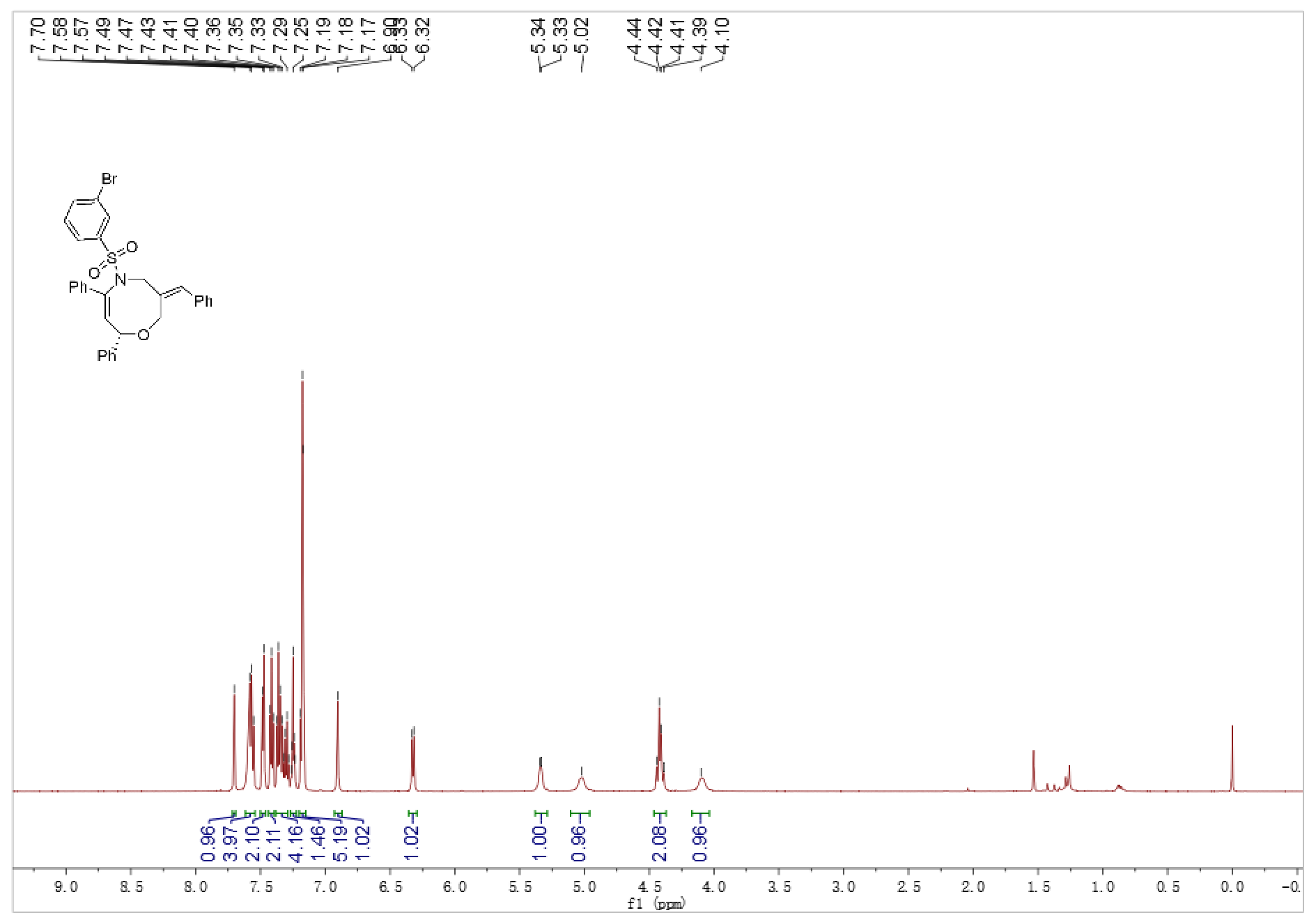
**

^1^H (CDCl_3_, 600 MHz) NMR of compound **23**

**
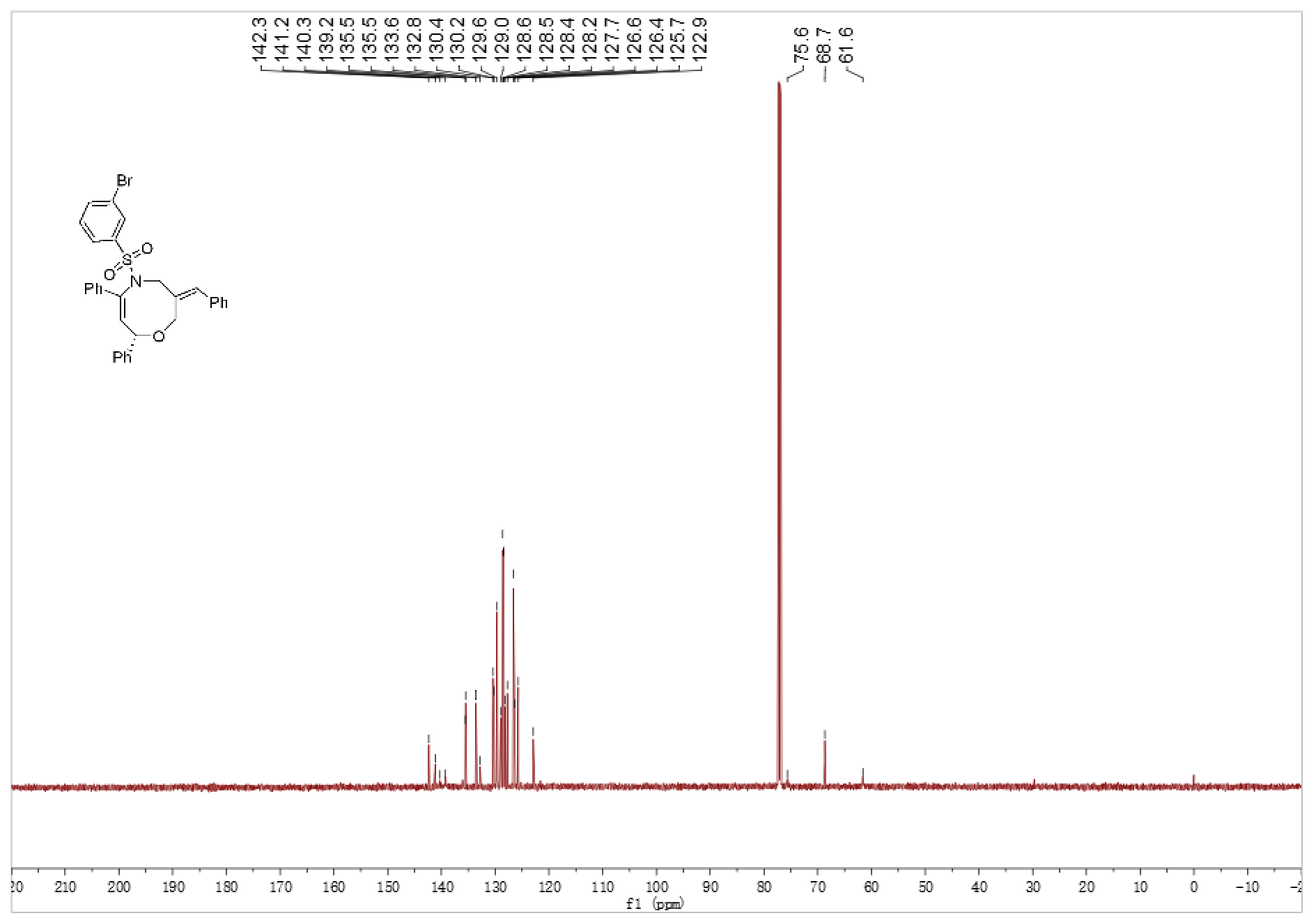
**

^13^C (CDCl_3_, 151 MHz) NMR of compound **23**

**
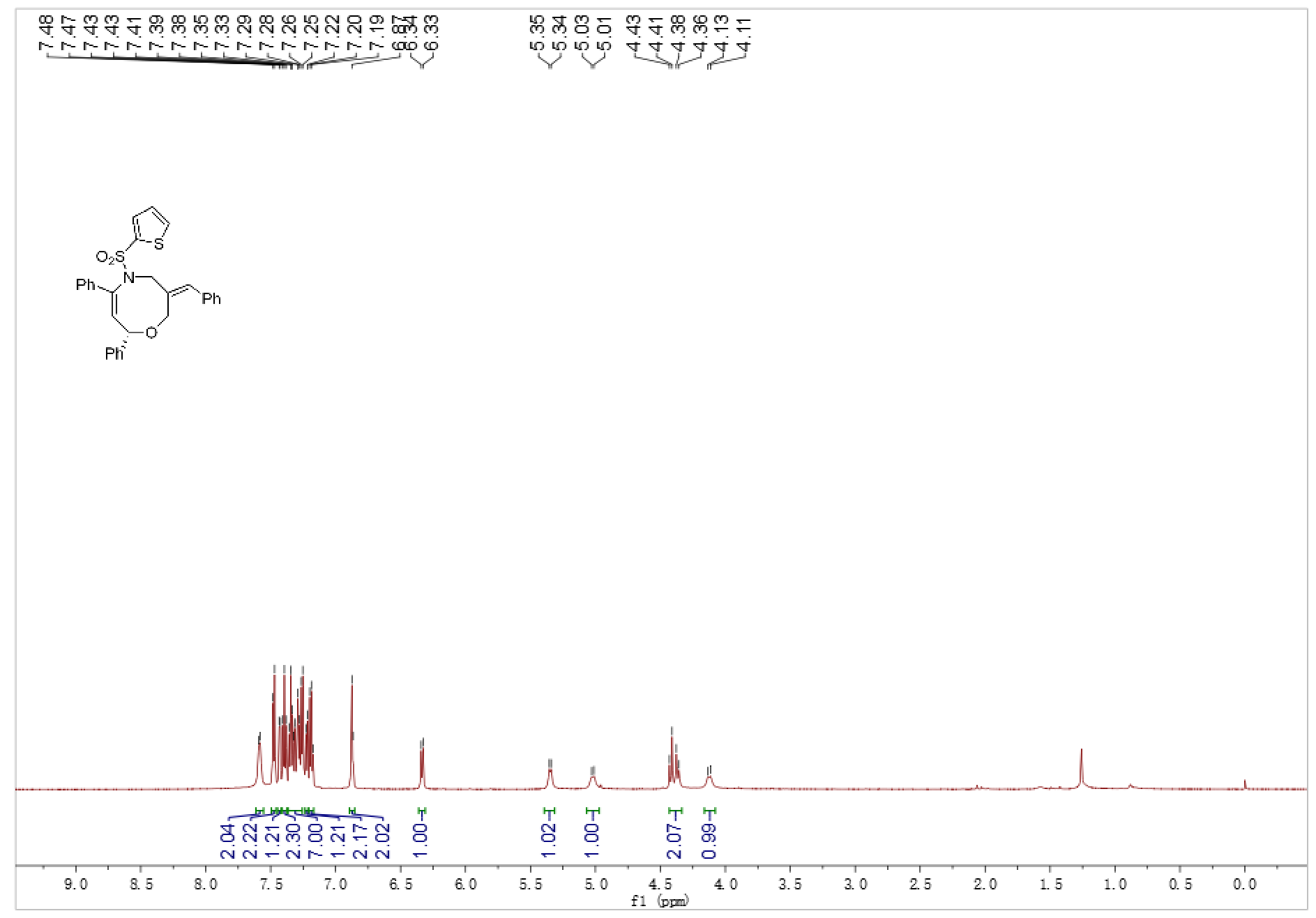
**

^1^H (CDCl_3_, 600 MHz) NMR of compound **24**

**
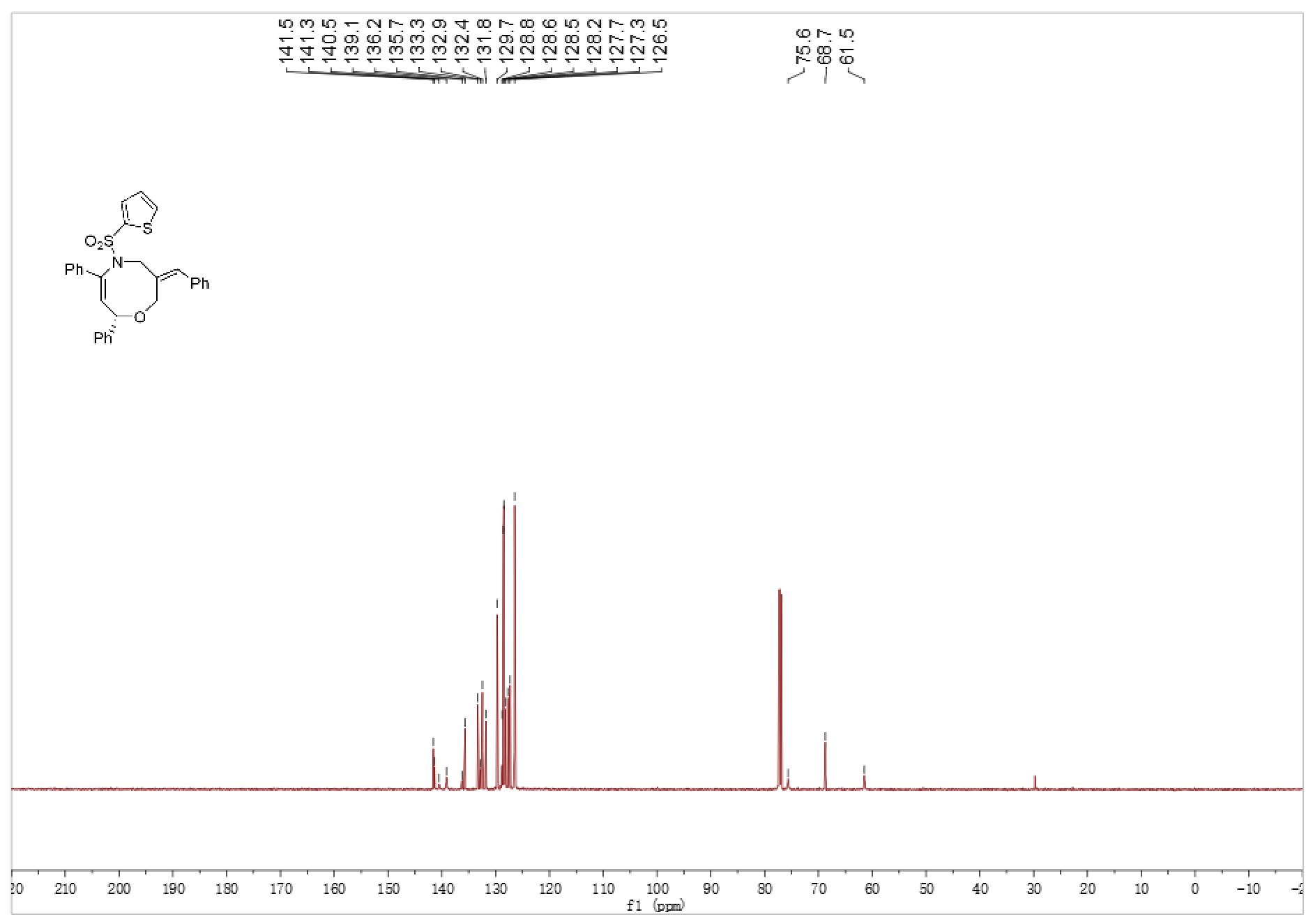
**

^13^C (CDCl_3_, 151 MHz) NMR of compound **24**

**
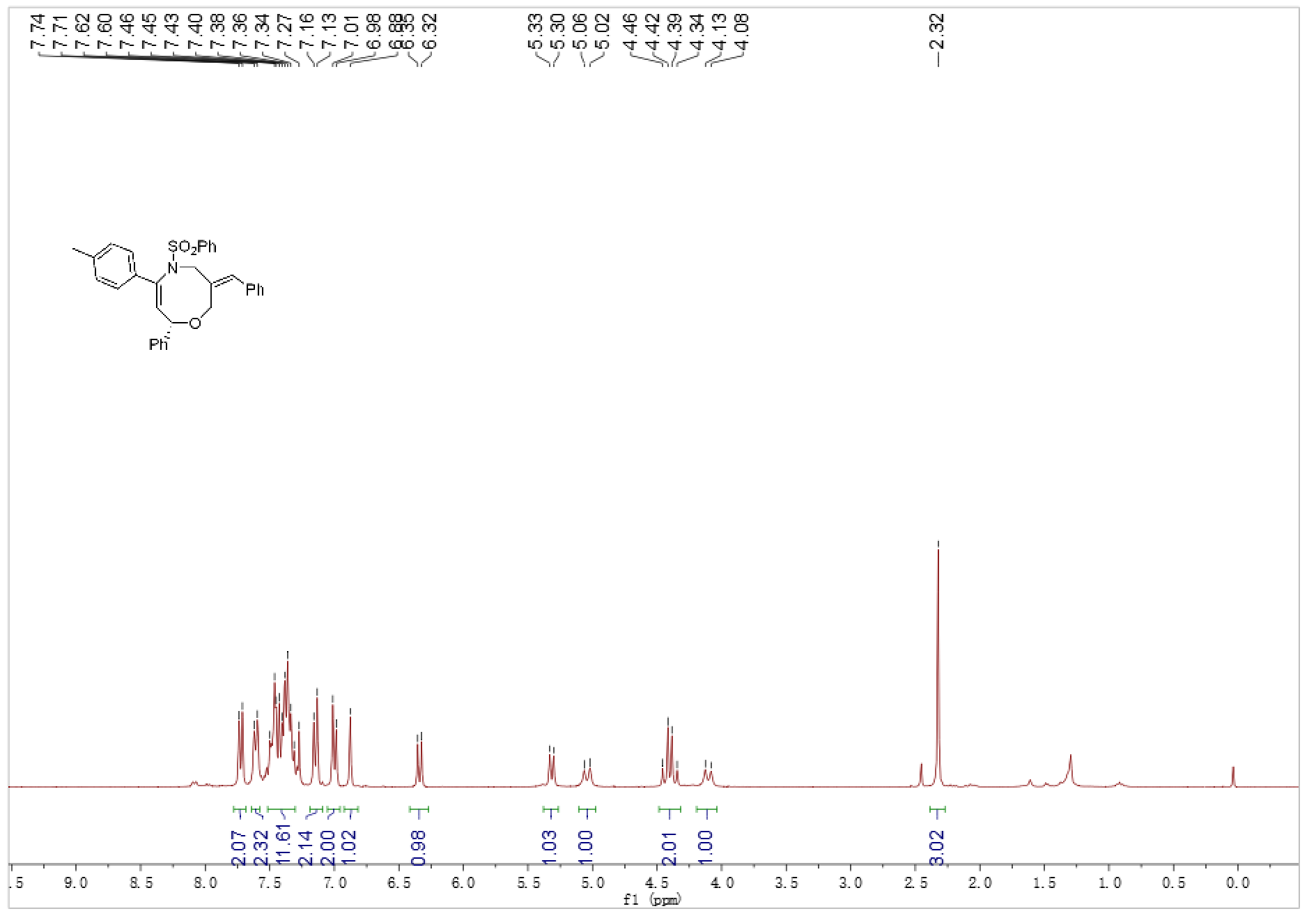
**

^1^H (CDCl_3_, 300 MHz) NMR of compound **25**

**
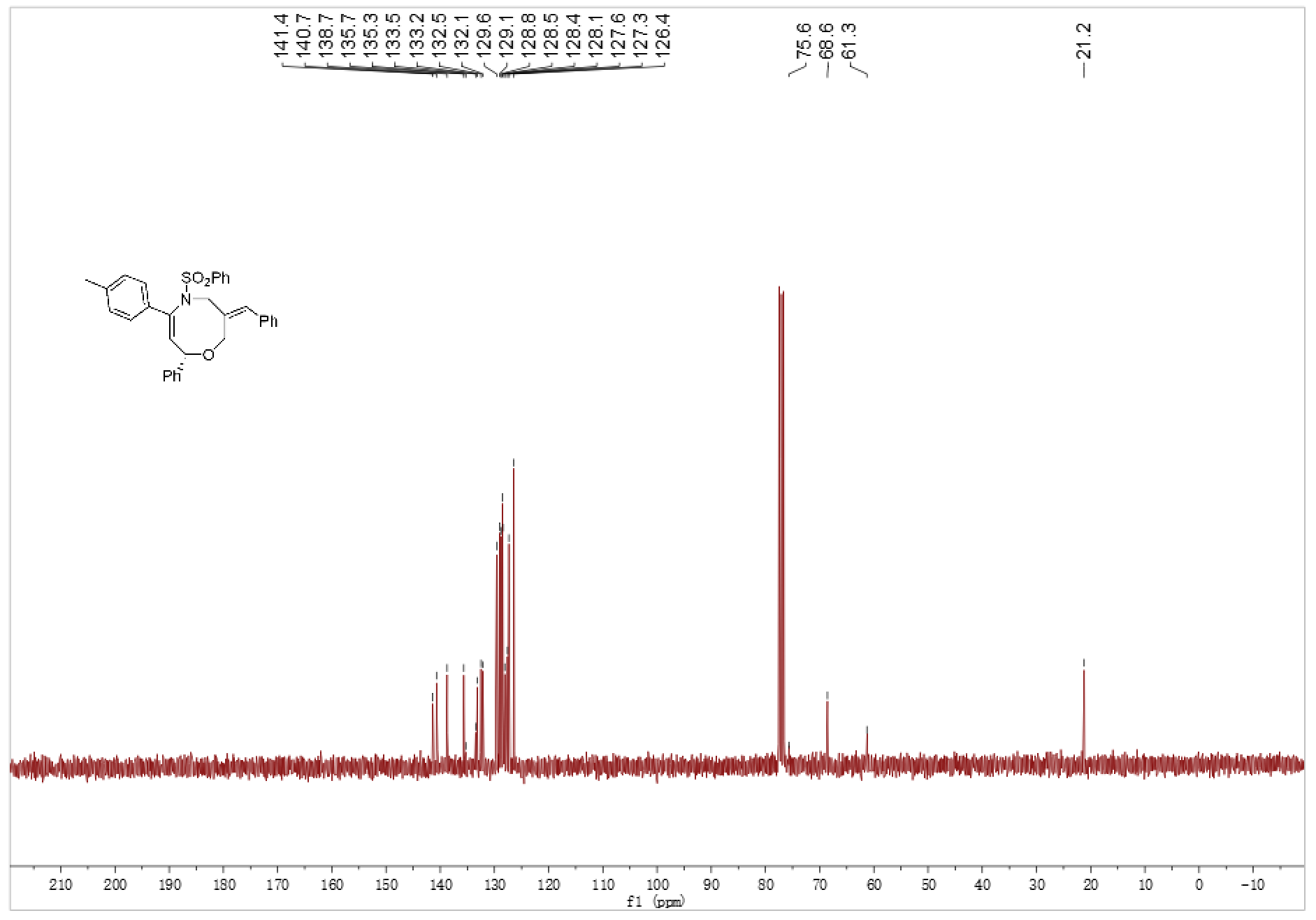
**

^13^C (CDCl_3_, 75 MHz) NMR of compound **25**

**
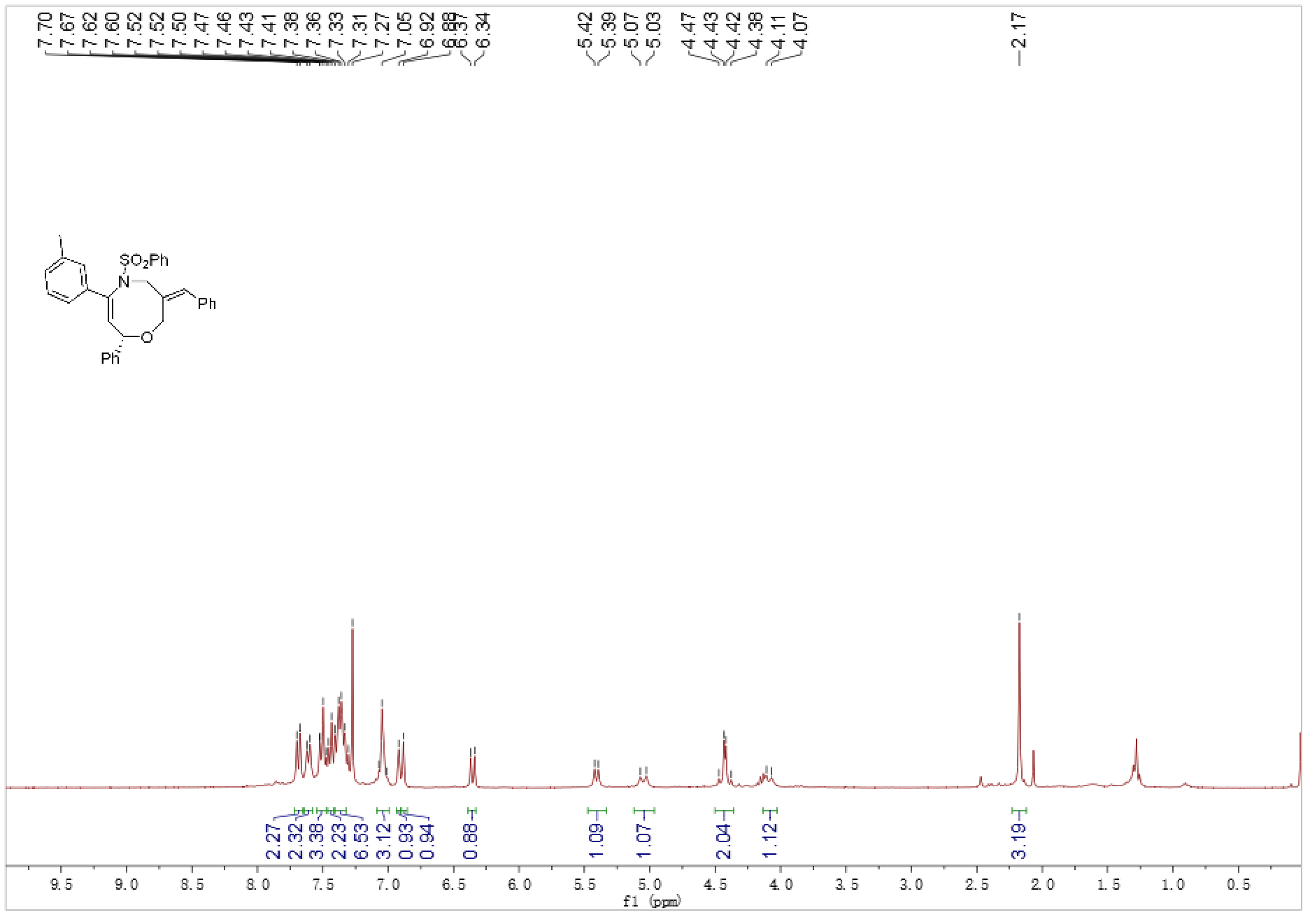
**

^1^H (CDCl_3_, 300 MHz) NMR of compound **26**

**
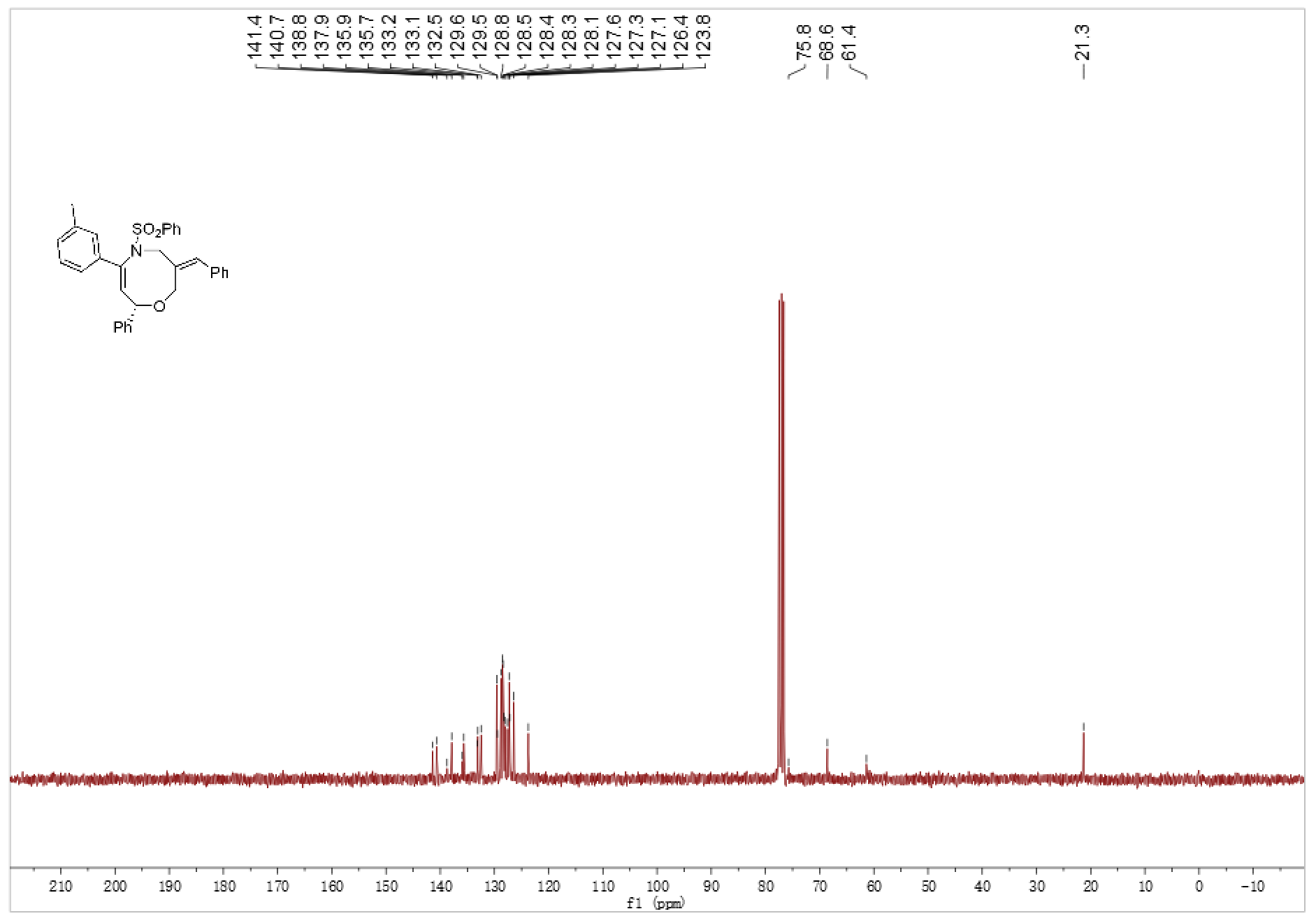
**

^13^C (CDCl_3_, 75 MHz) NMR of compound **26**

**
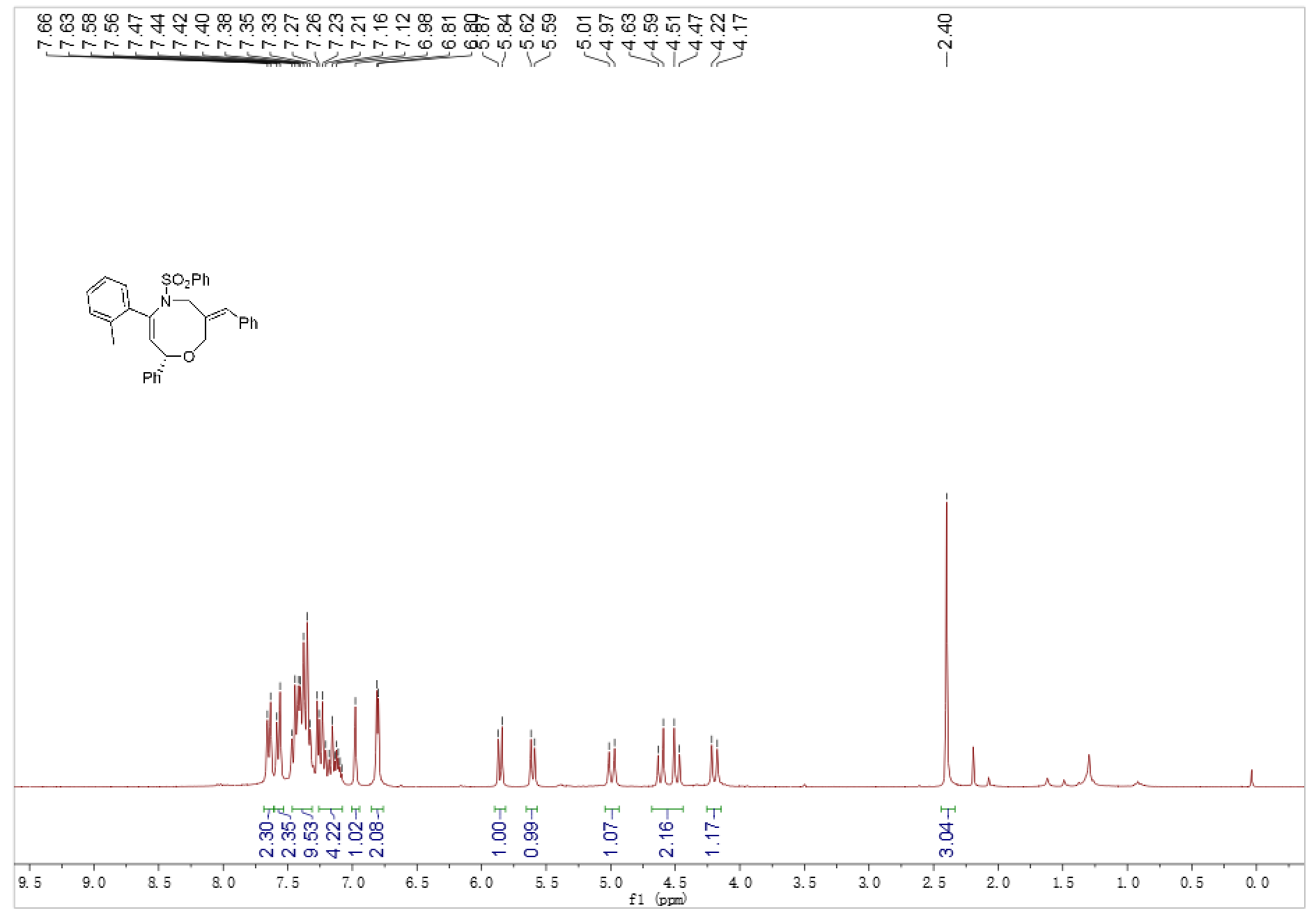
**

^1^H (CDCl_3_, 300 MHz) NMR of compound **27**

**
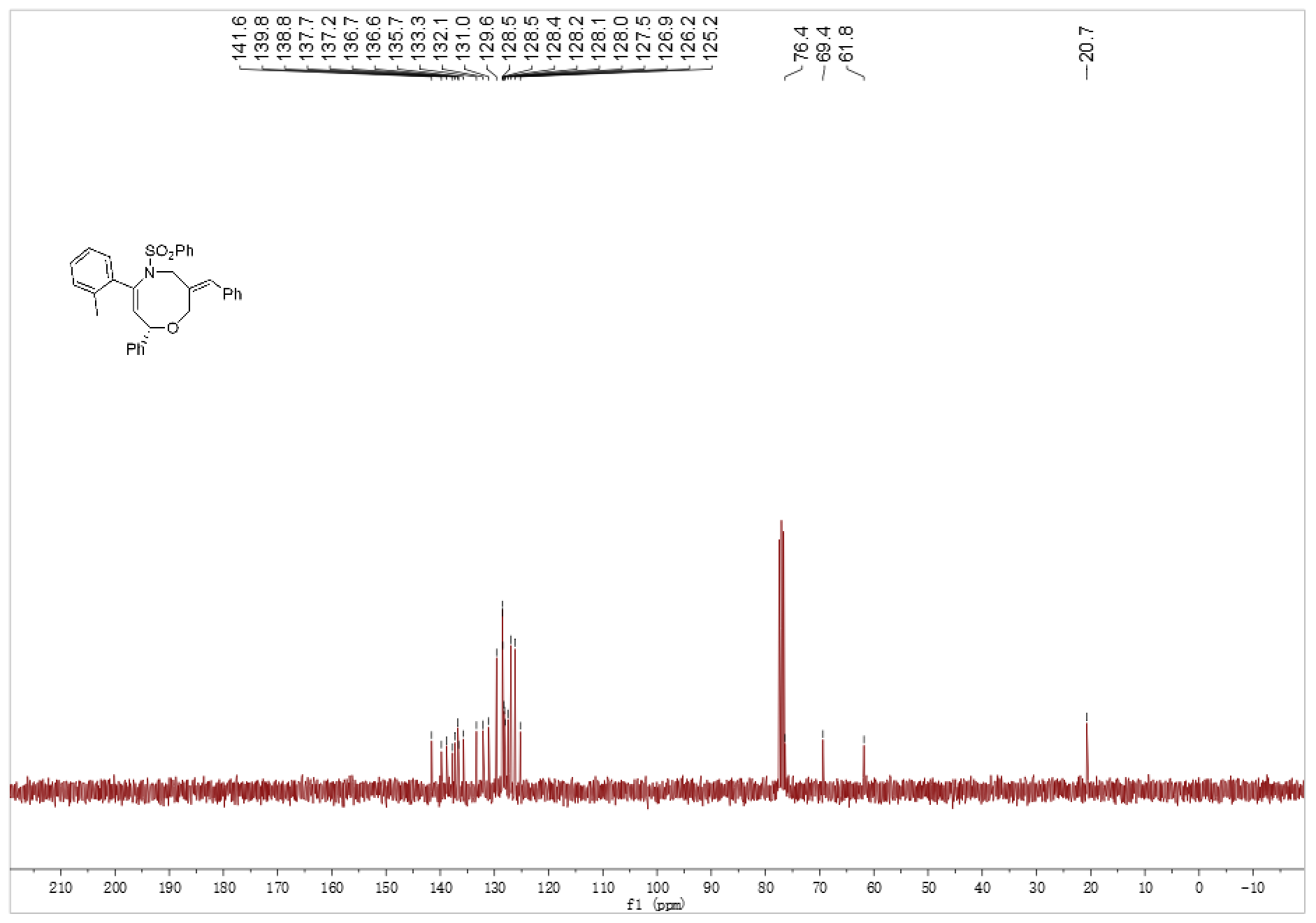
**

^13^C (CDCl_3_, 75 MHz) NMR of compound **27**

**
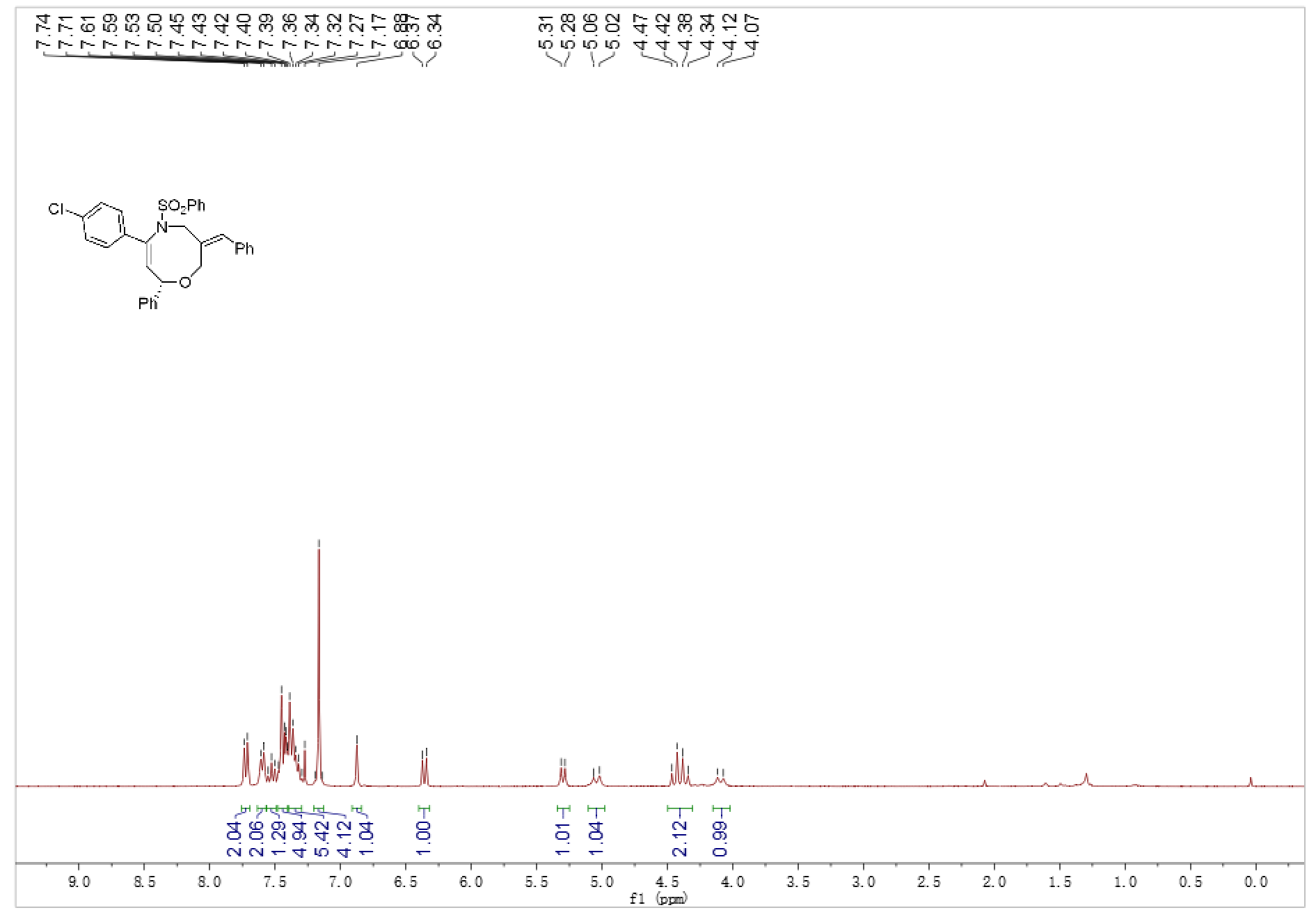
**

^1^H (CDCl_3_, 300 MHz) NMR of compound **28**

**
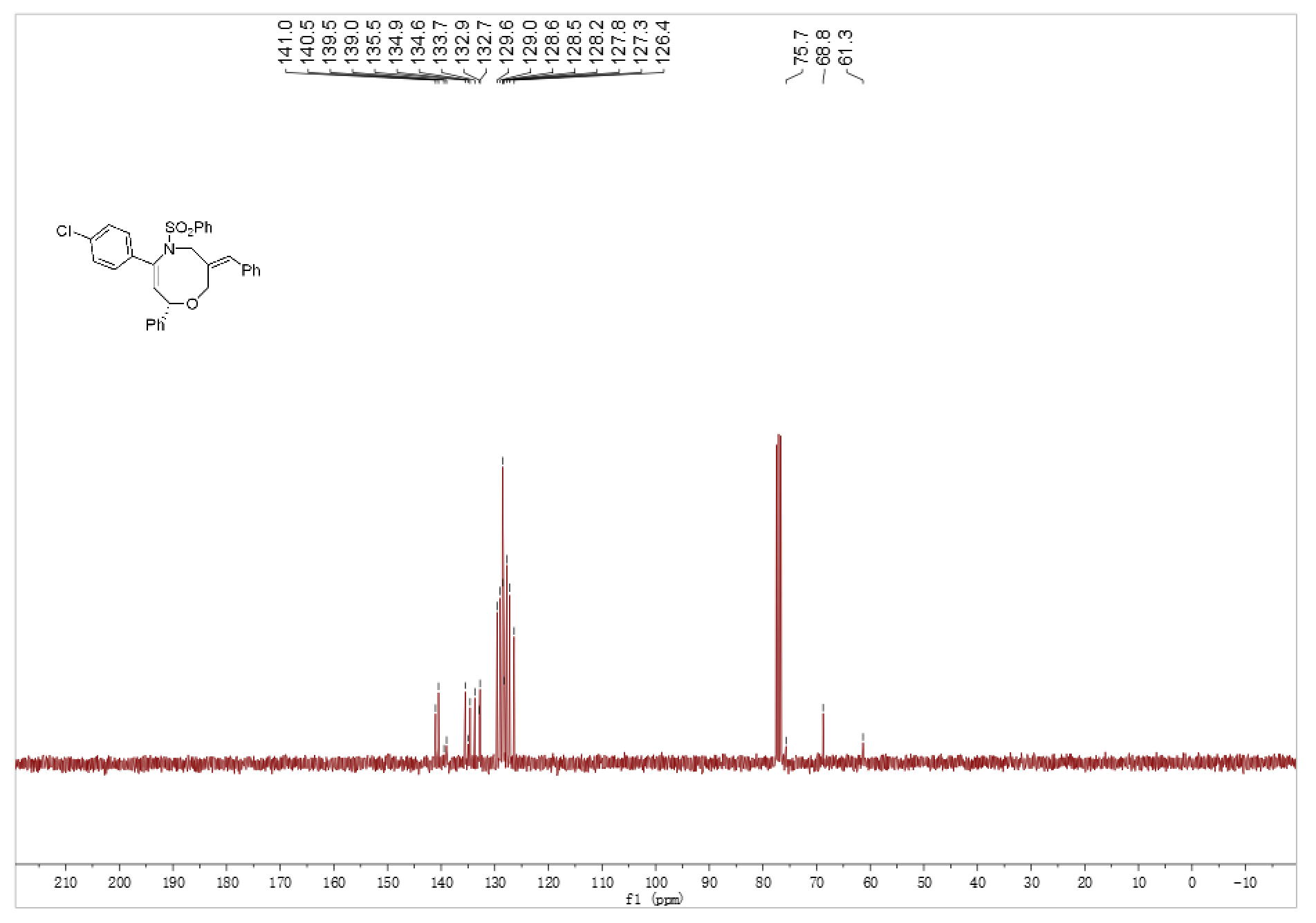
**

^13^C (CDCl_3_, 75 MHz) NMR of compound **28**

**
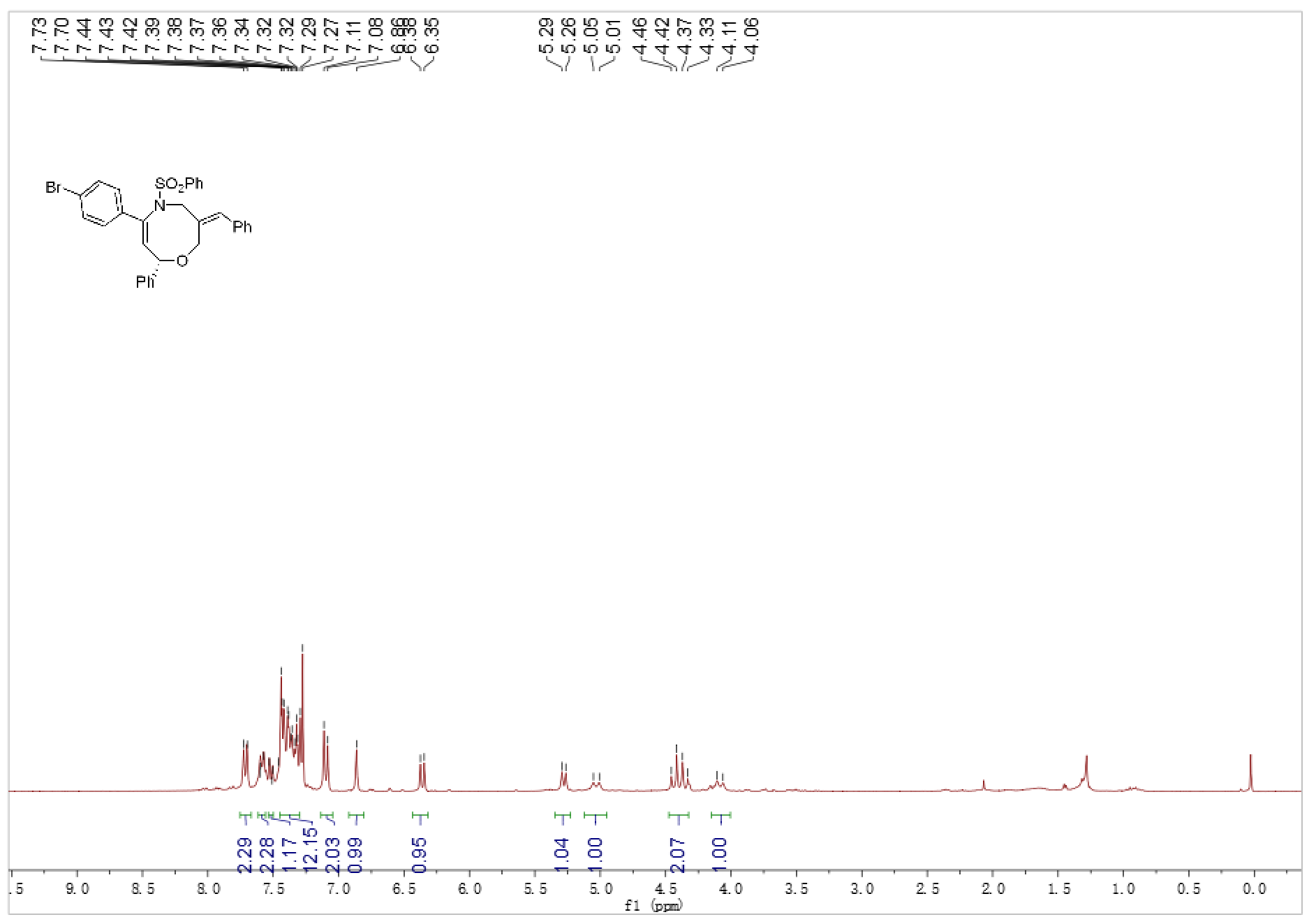
**

^1^H (CDCl_3_, 300 MHz) NMR of compound **29**

**
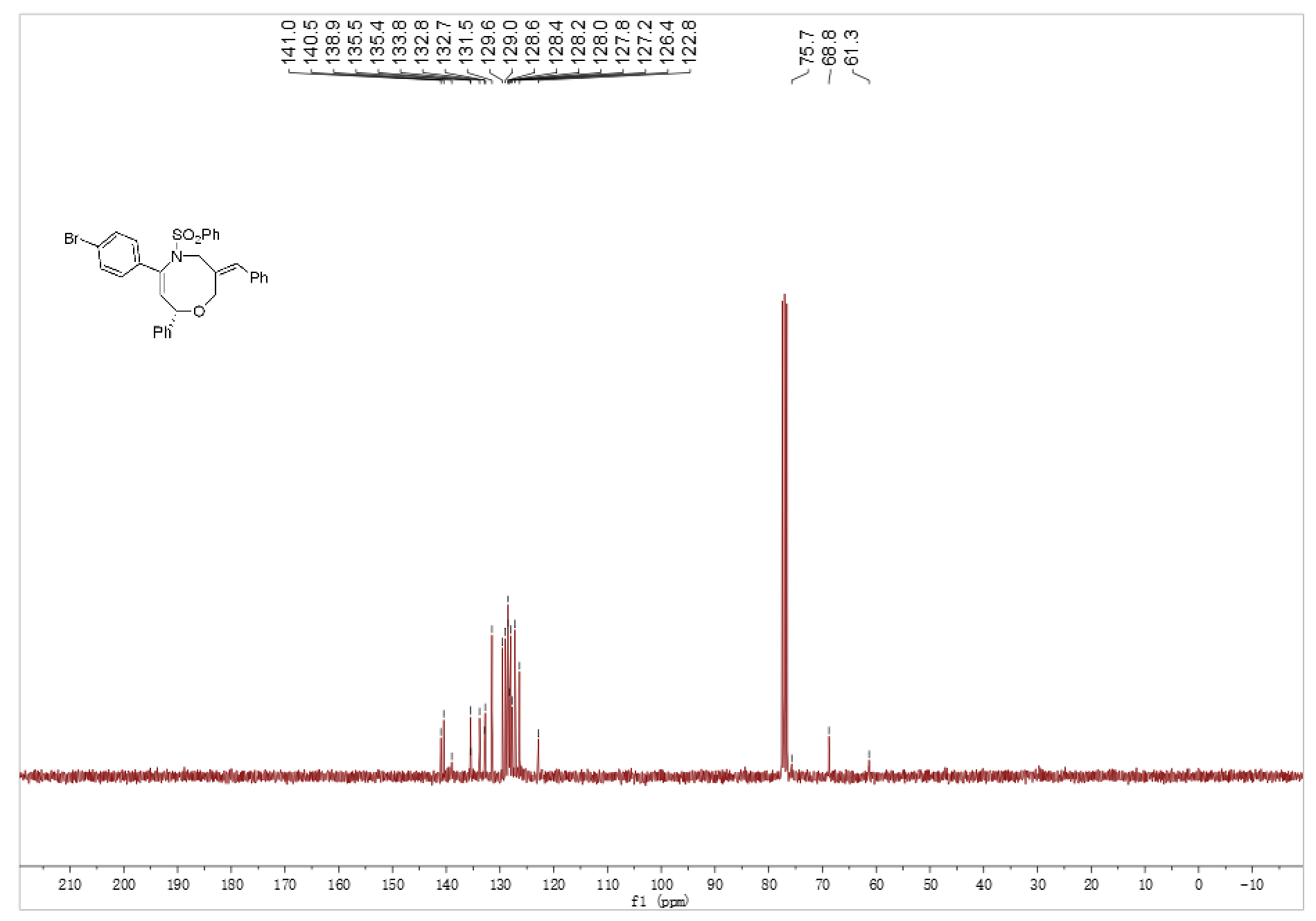
**

^13^C (CDCl_3_, 75 MHz) NMR of compound **29**

**
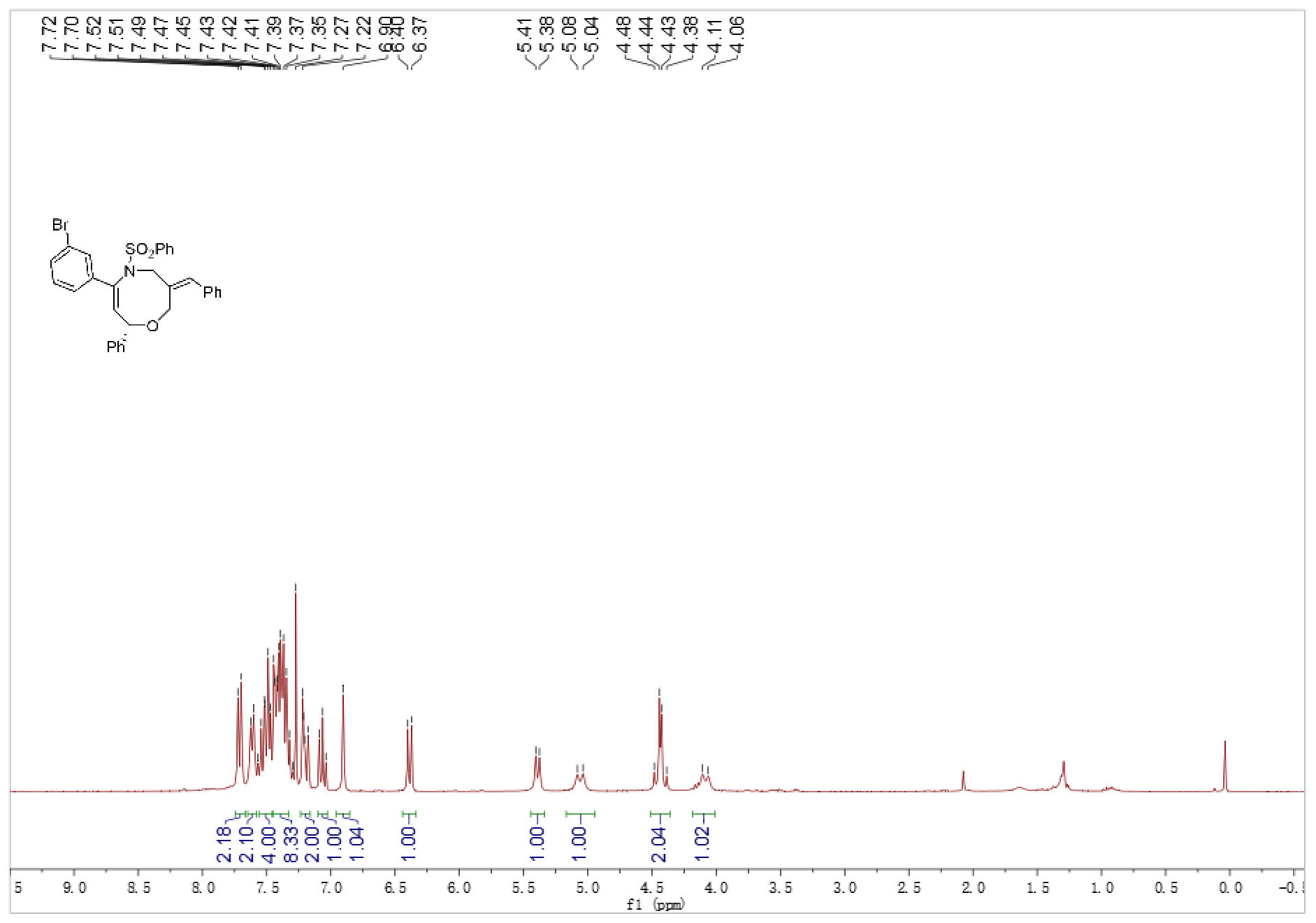
**

^1^H (CDCl_3_, 300 MHz) NMR of compound **30**

**
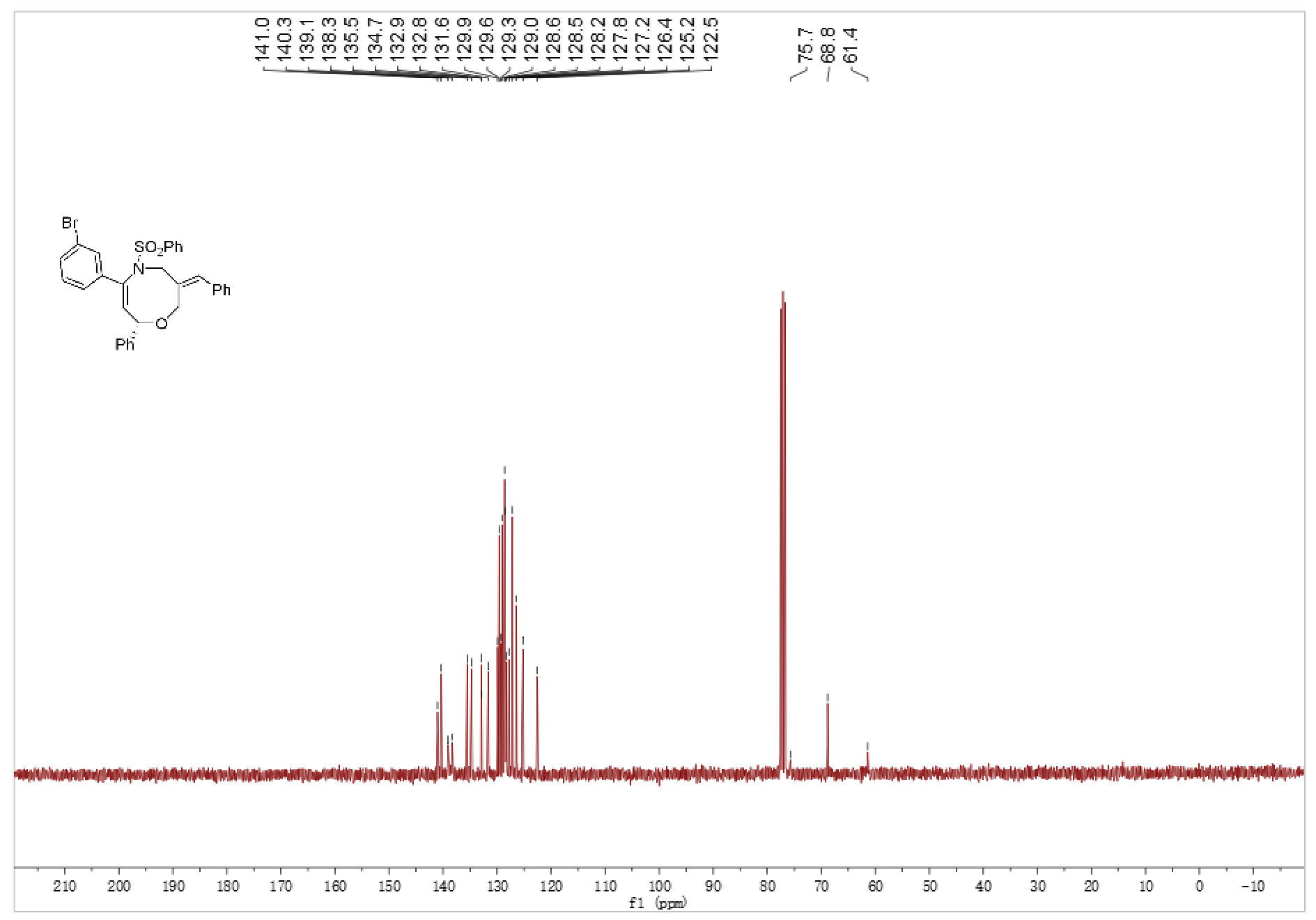
**

^13^C (CDCl_3_, 75 MHz) NMR of compound **30**

**
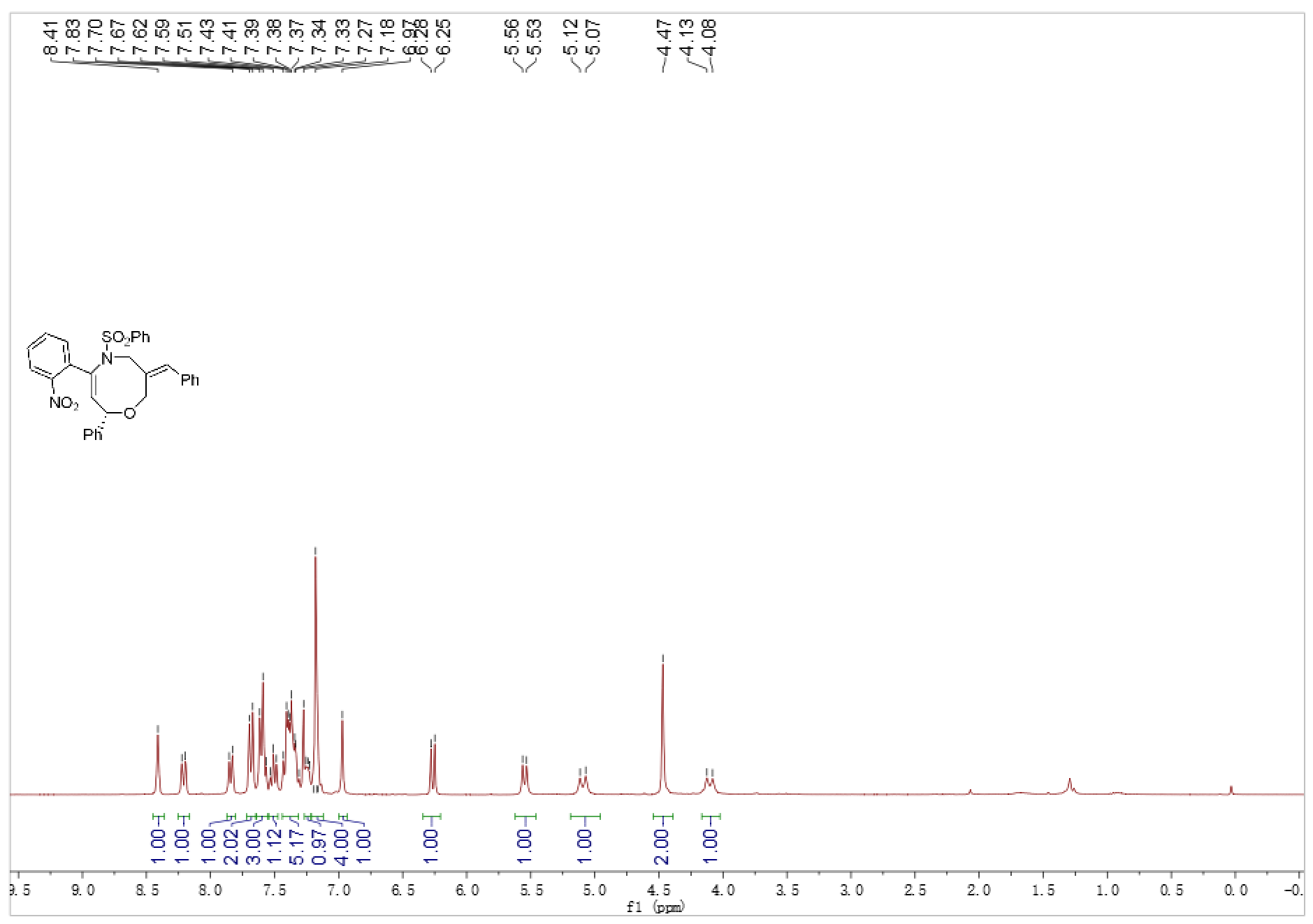
**

^1^H (CDCl_3_, 300 MHz) NMR of compound **31**

**
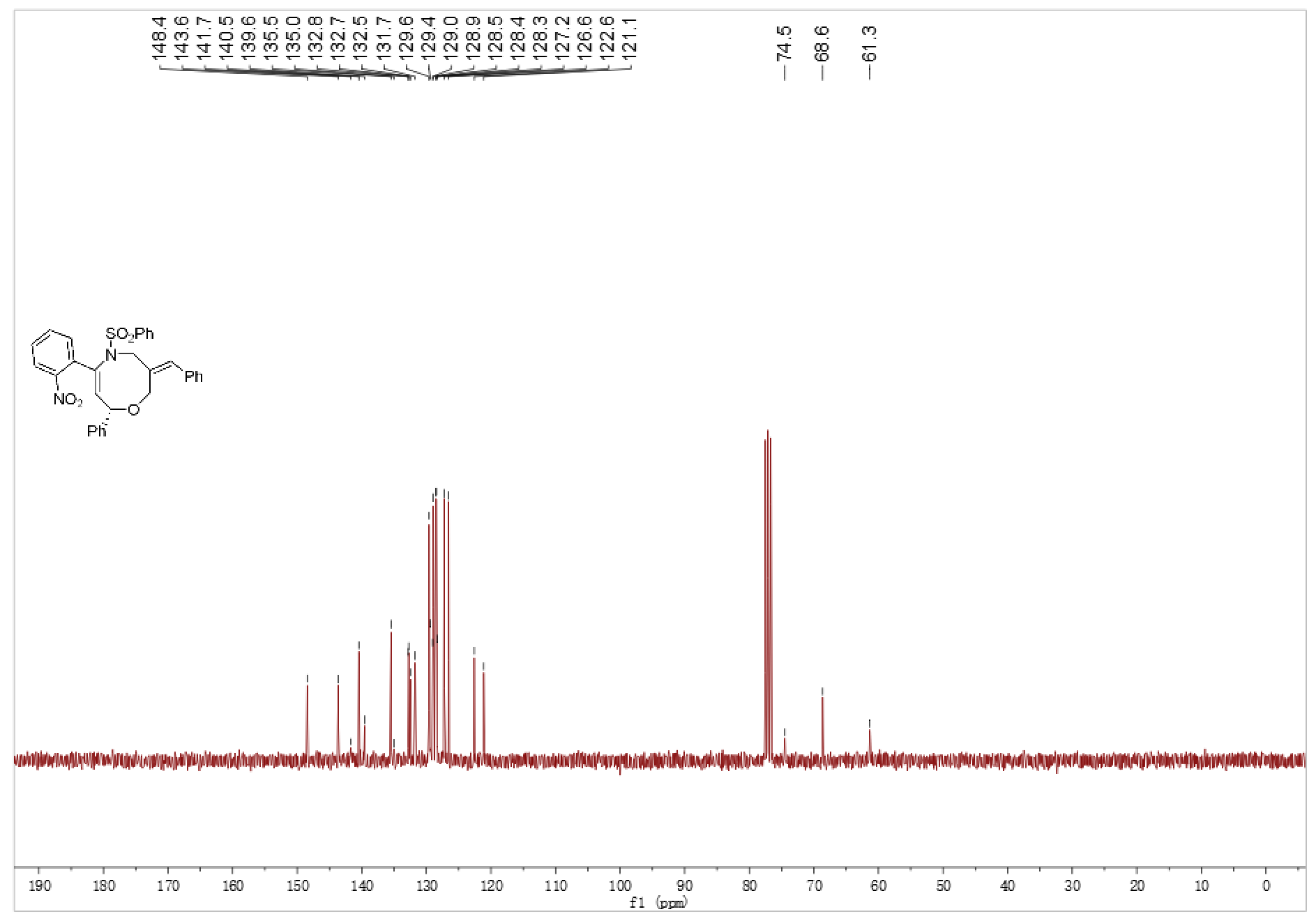
**

^13^C (CDCl_3_, 75 MHz) NMR of compound **31**

**
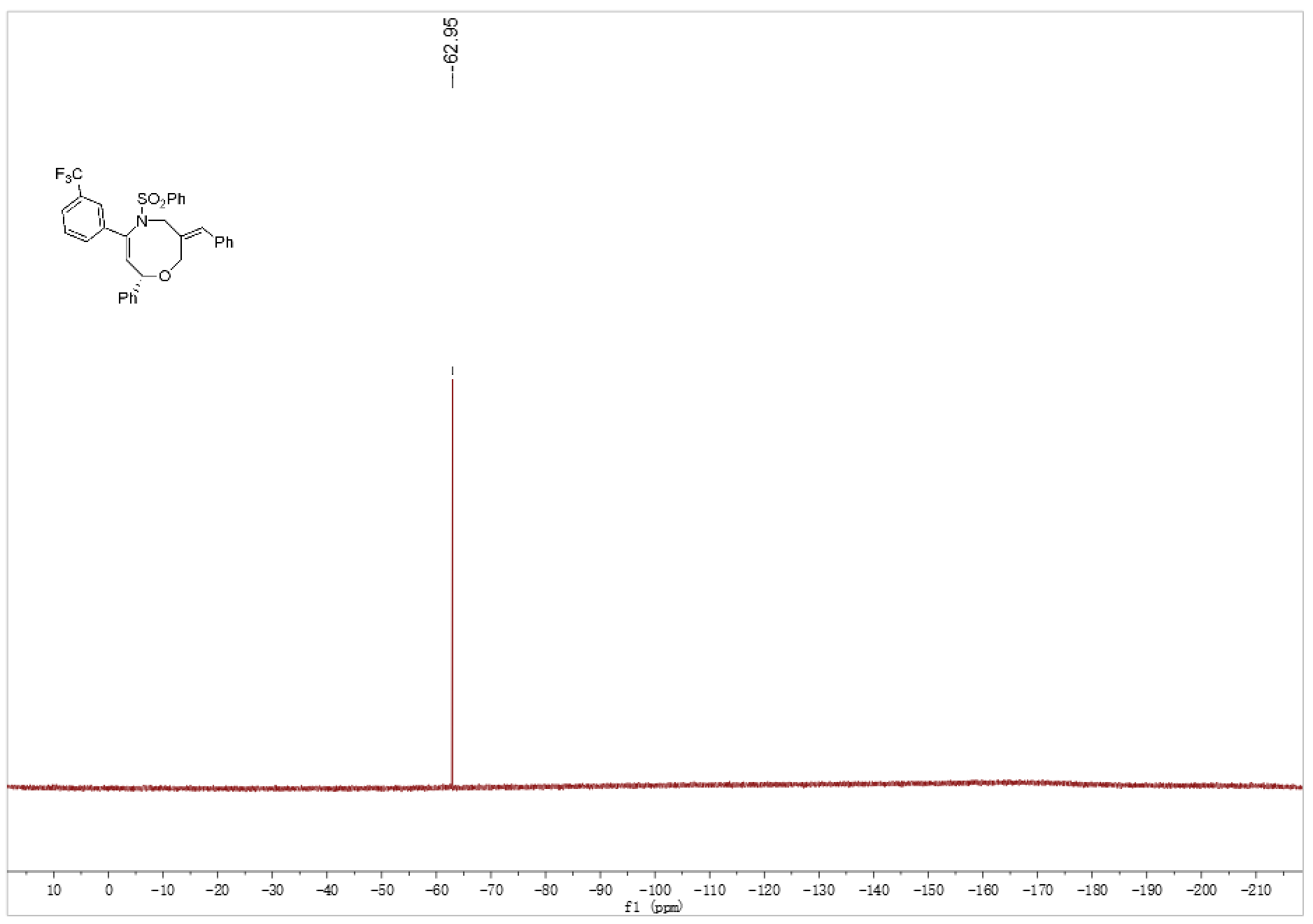
**

^19^F (CDCl_3_, 282 MHz) NMR of compound **32**

**
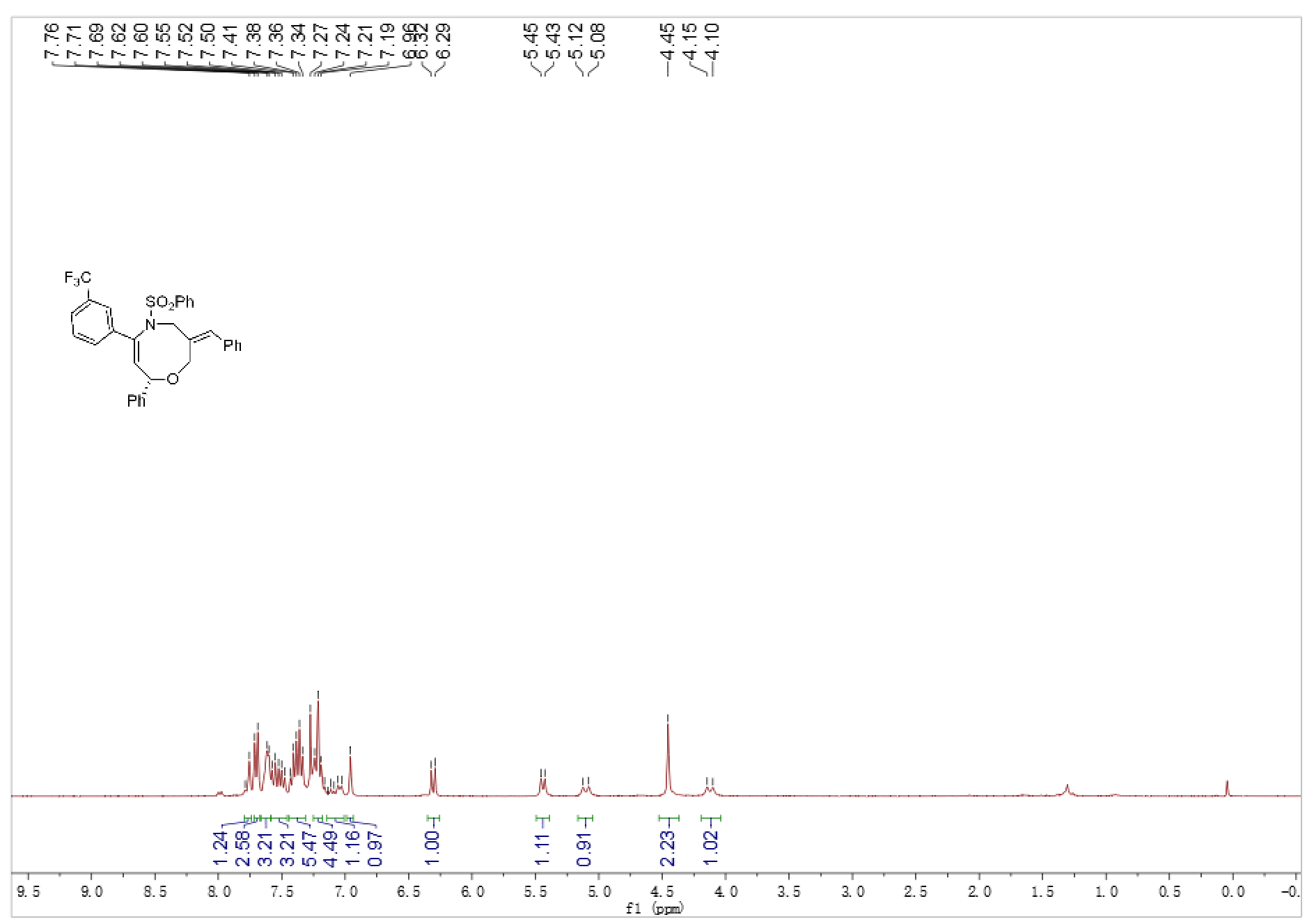
**

^1^H (CDCl_3_, 300 MHz) NMR of compound **32**

**
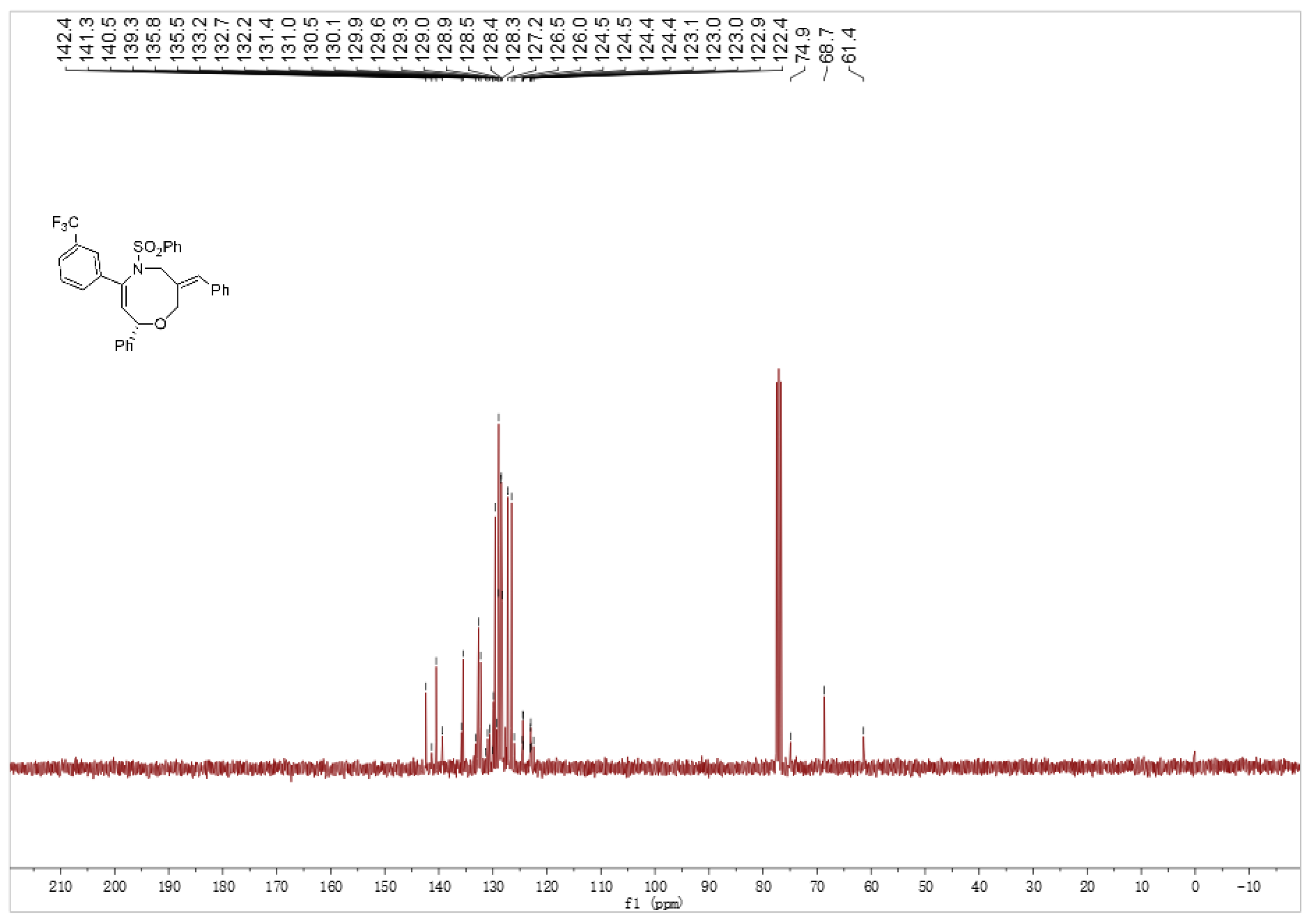
**

^13^C (CDCl_3_, 75 MHz) NMR of compound **32**

**
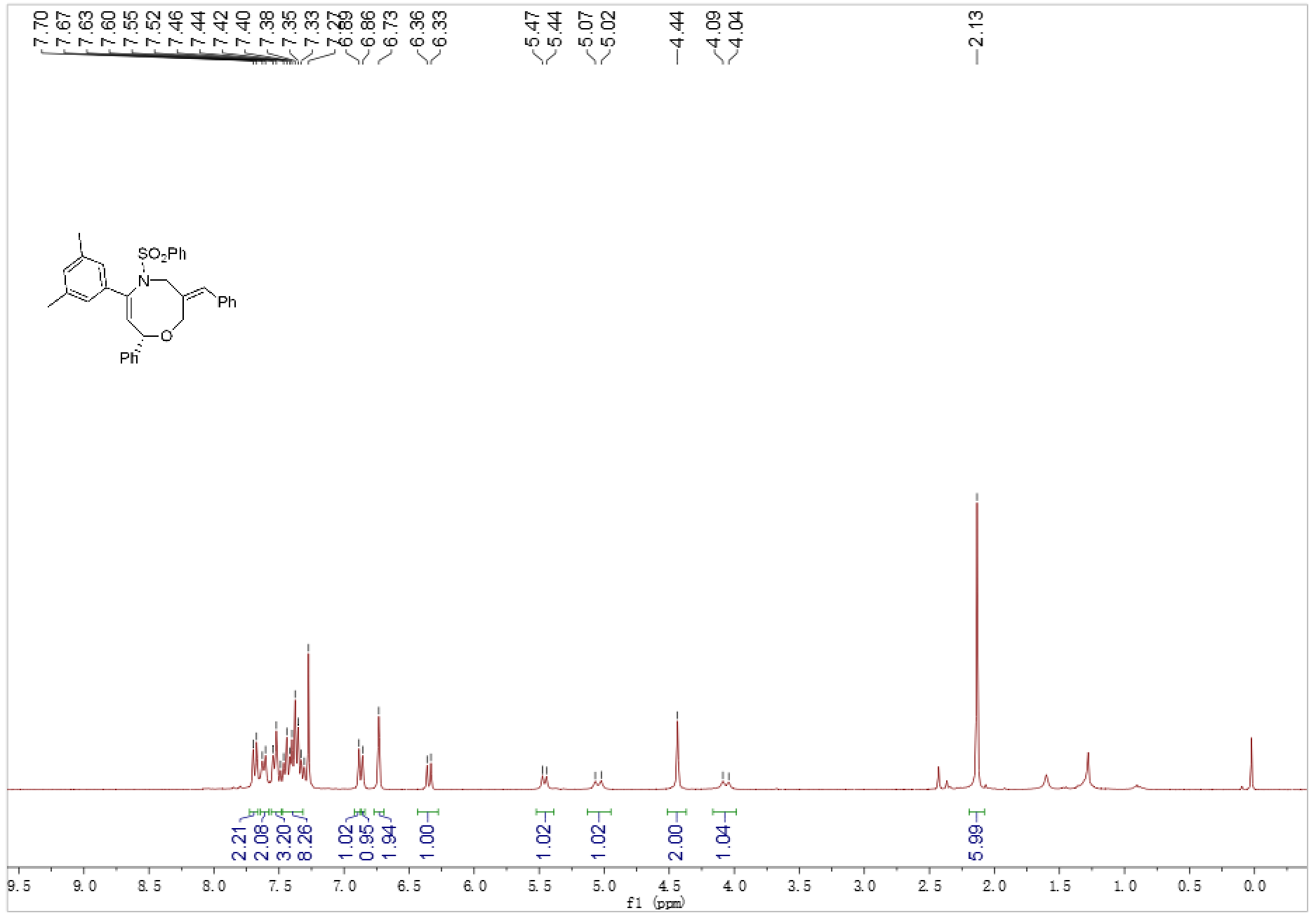
**

^1^H (CDCl_3_, 300 MHz) NMR of compound **33**

**
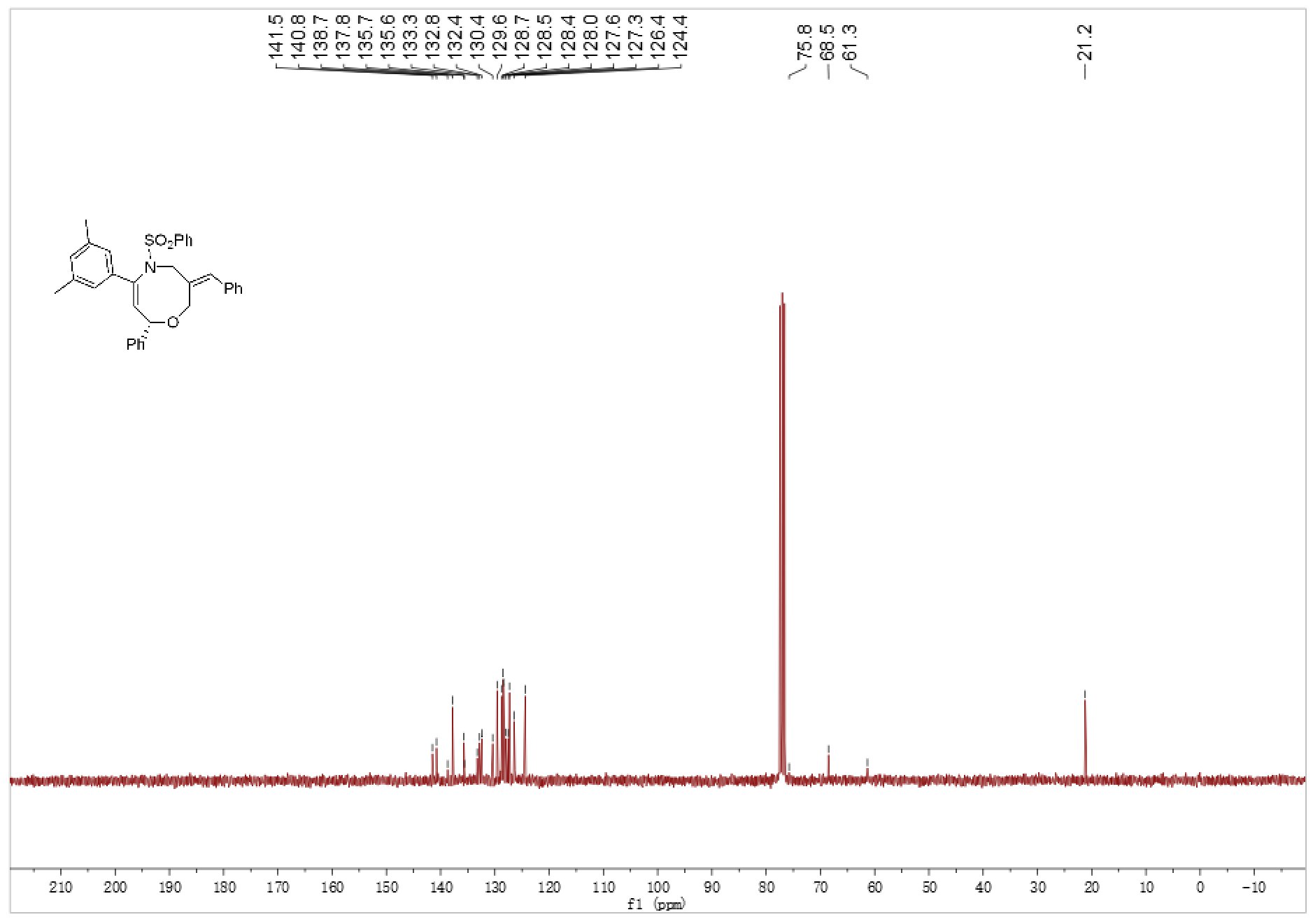
**

^13^C (CDCl_3_, 75 MHz) NMR of compound **33**

**
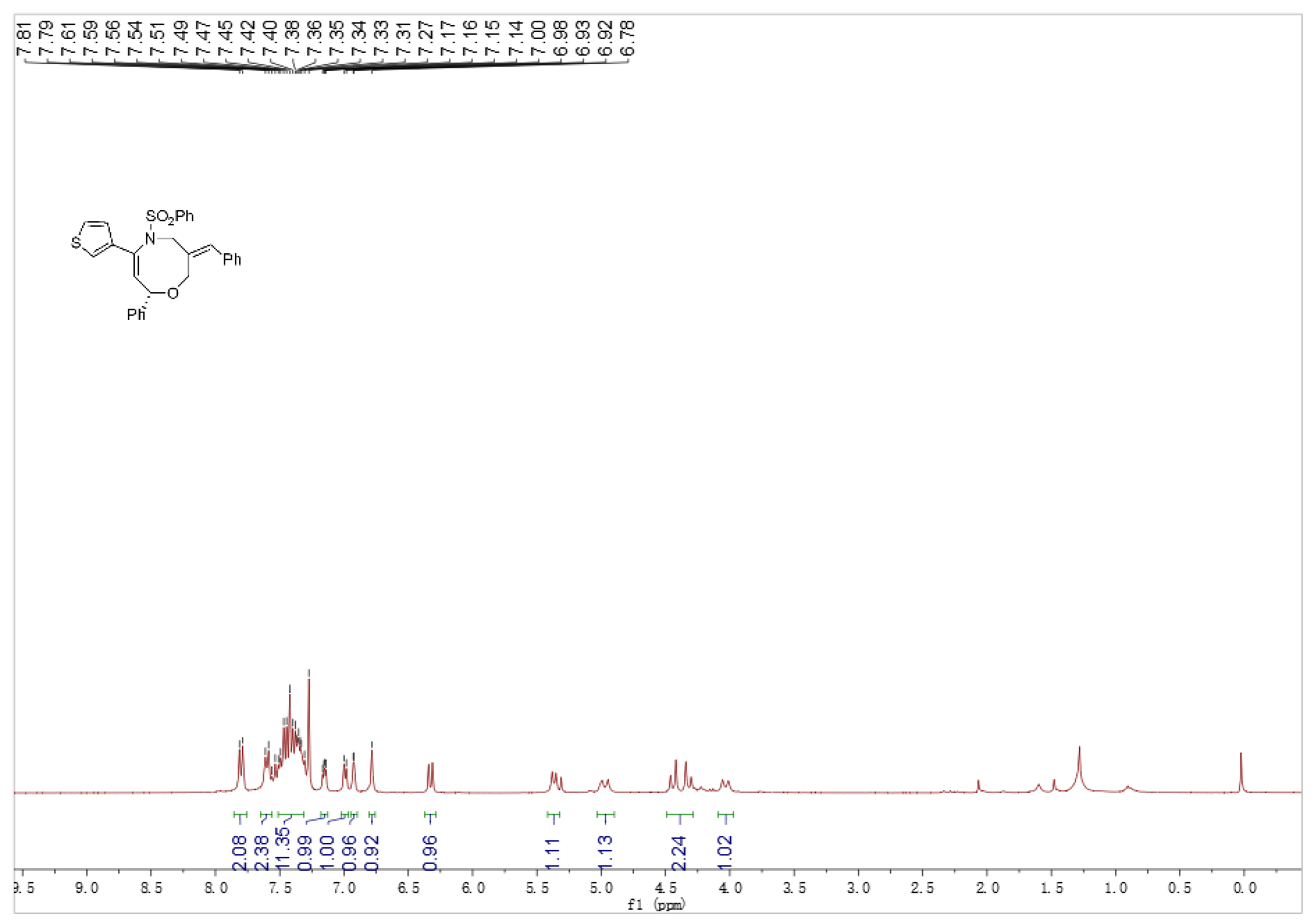
**

^1^H (CDCl_3_, 300 MHz) NMR of compound **34**

**
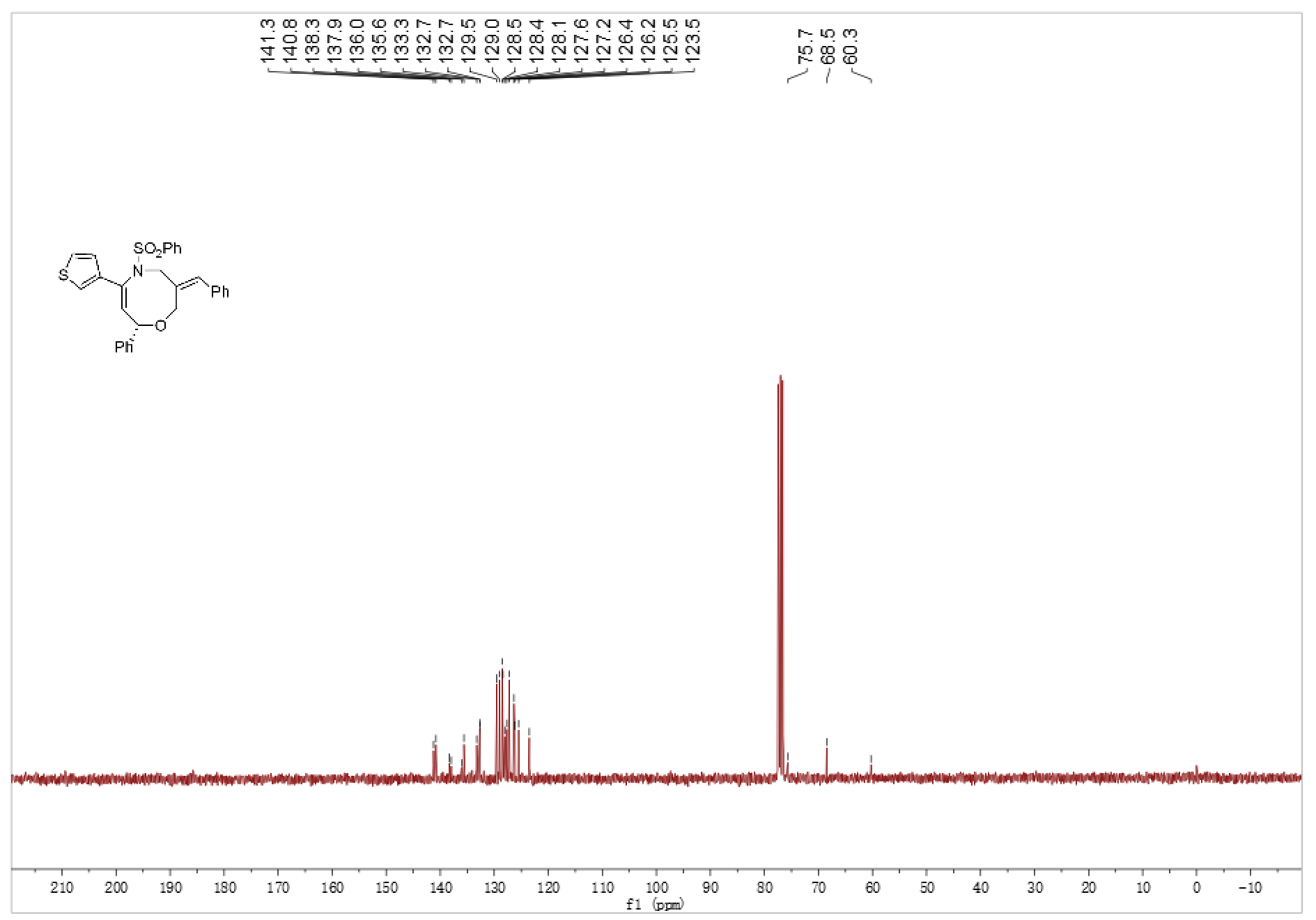
**

^13^C (CDCl_3_, 75 MHz) NMR of compound **34**

**
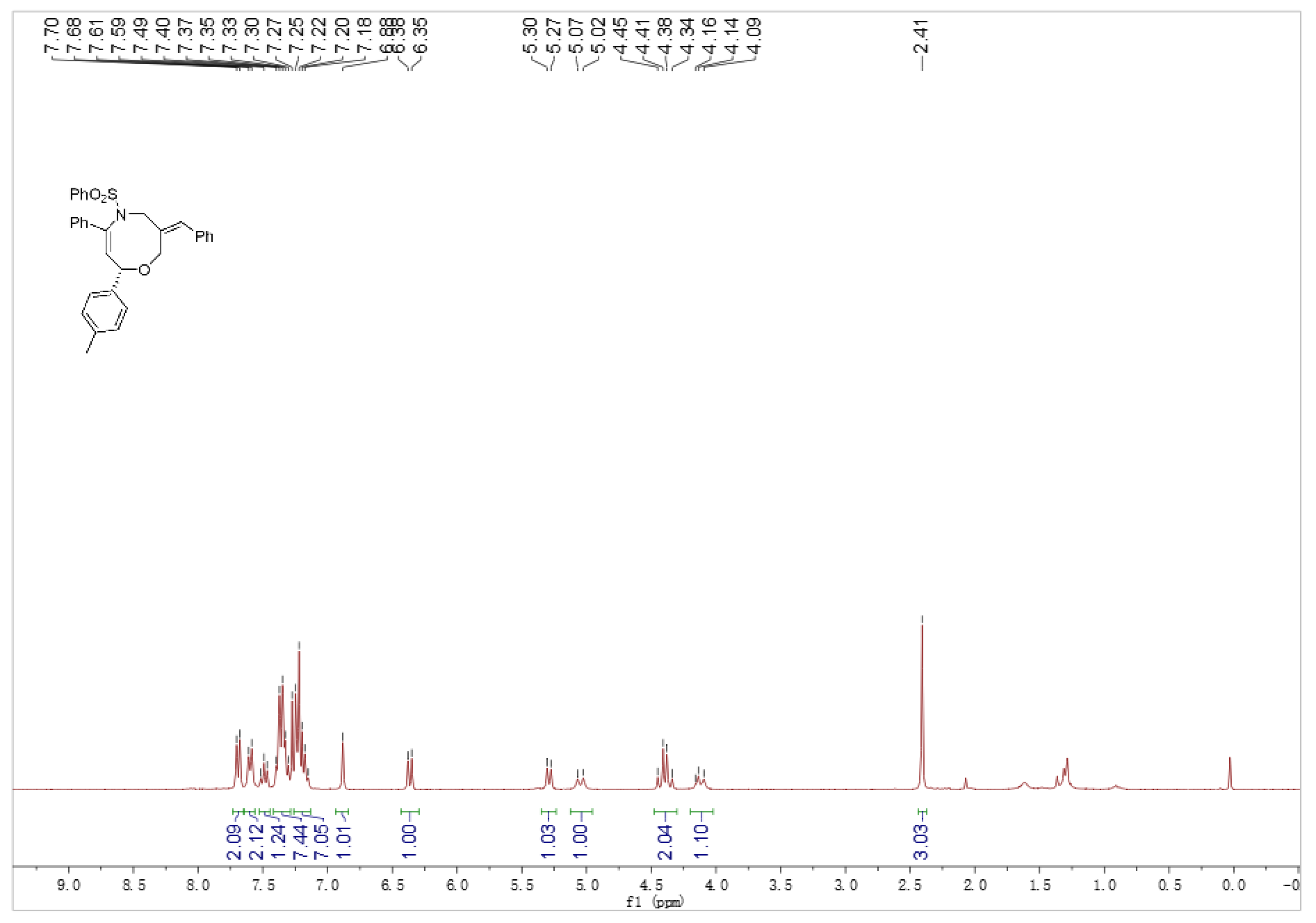
**

^1^H (CDCl_3_, 300 MHz) NMR of compound **35**

**
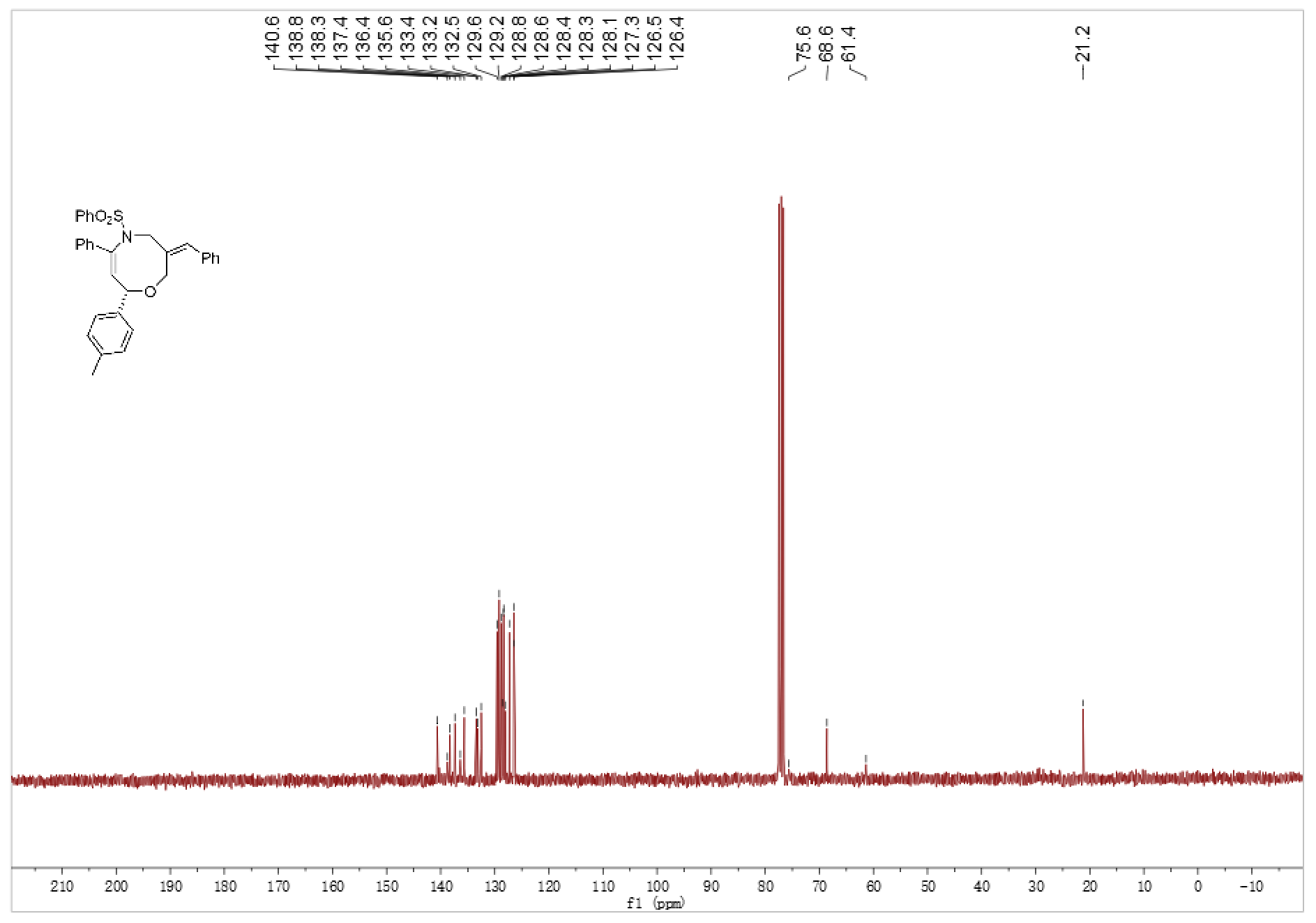
**

^13^C (CDCl_3_, 75 MHz) NMR of compound **35**

**
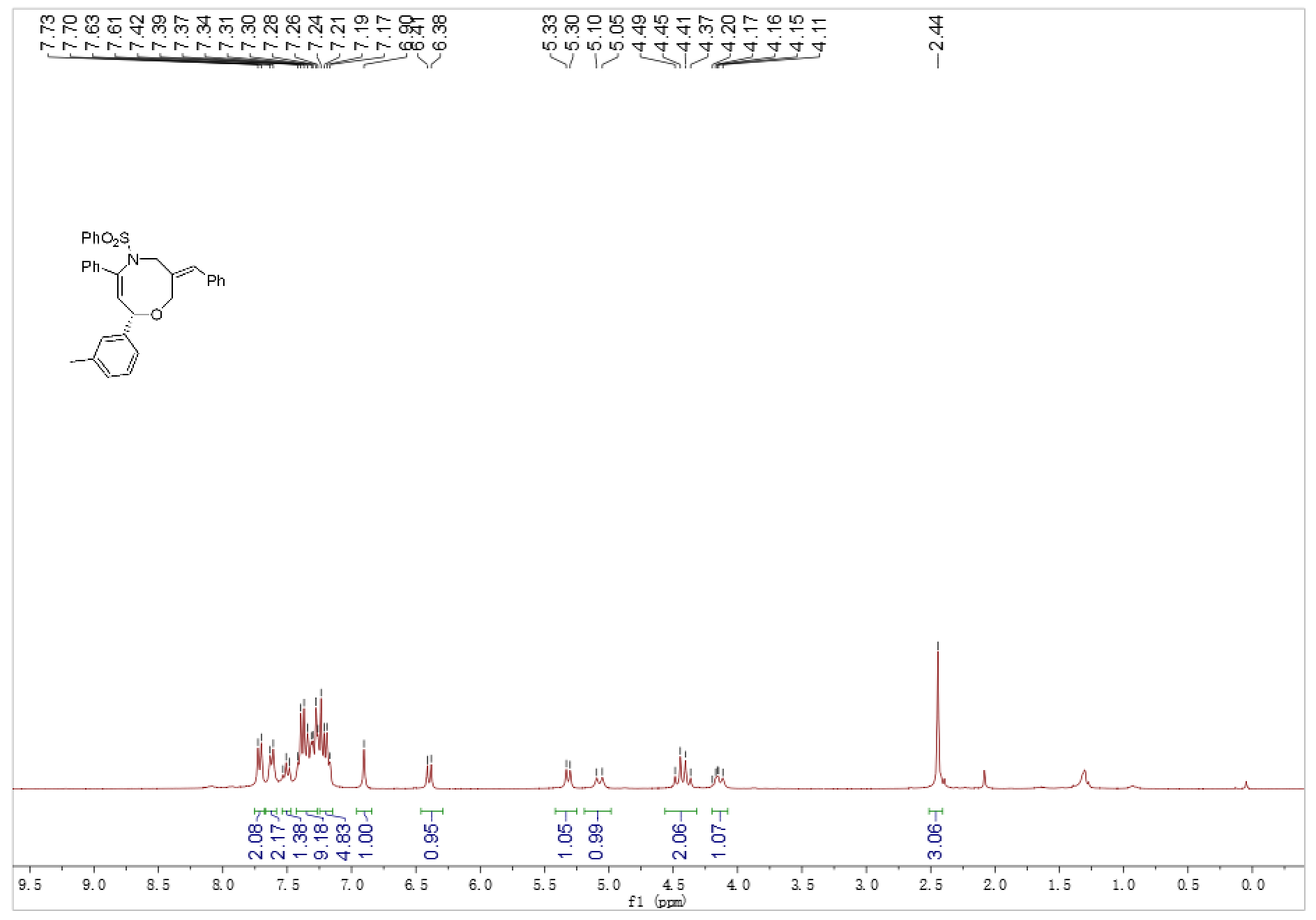
**

^1^H (CDCl_3_, 300 MHz) NMR of compound **36**

**
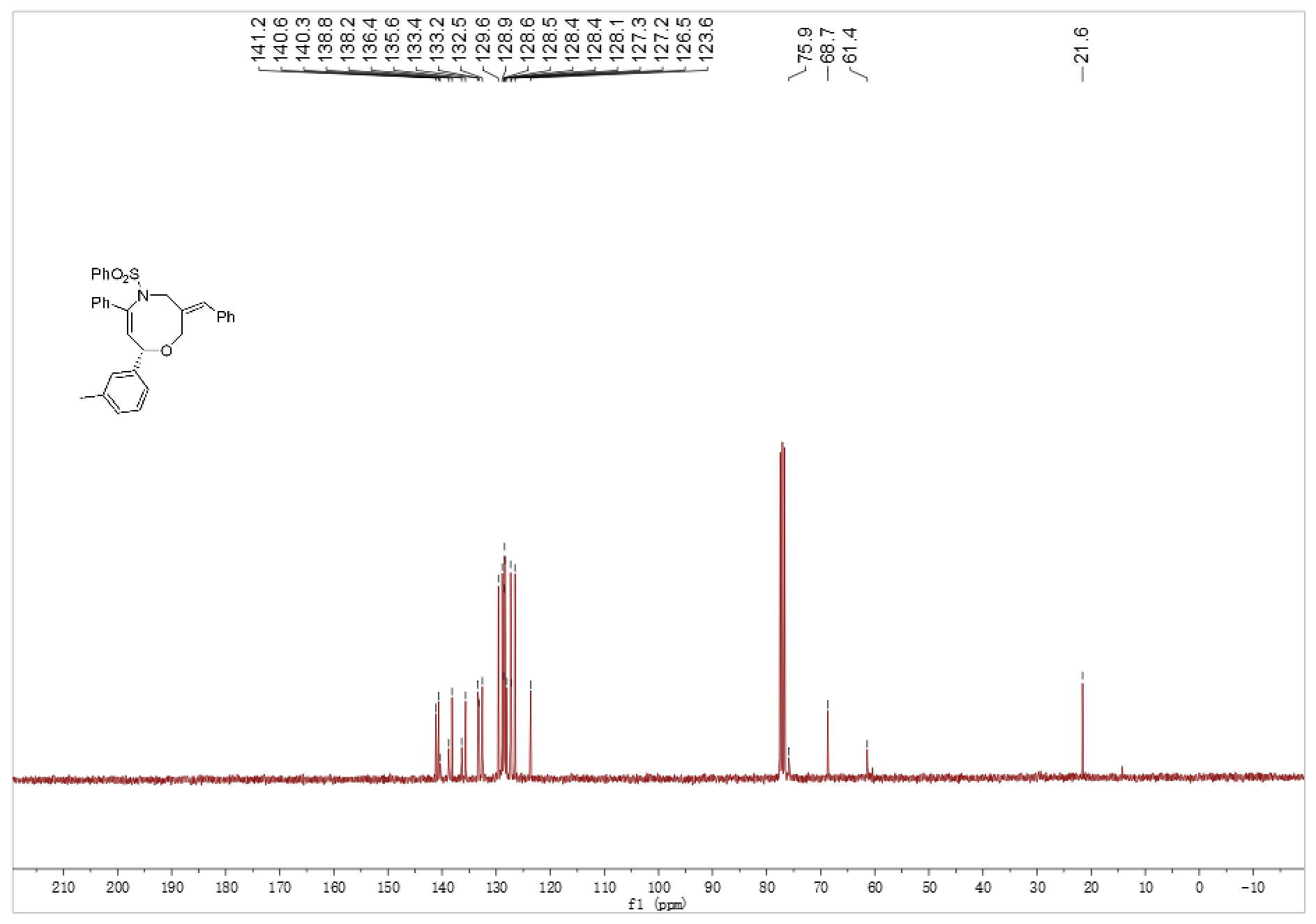
**

^13^C (CDCl_3_, 75 MHz) NMR of compound **36**

**
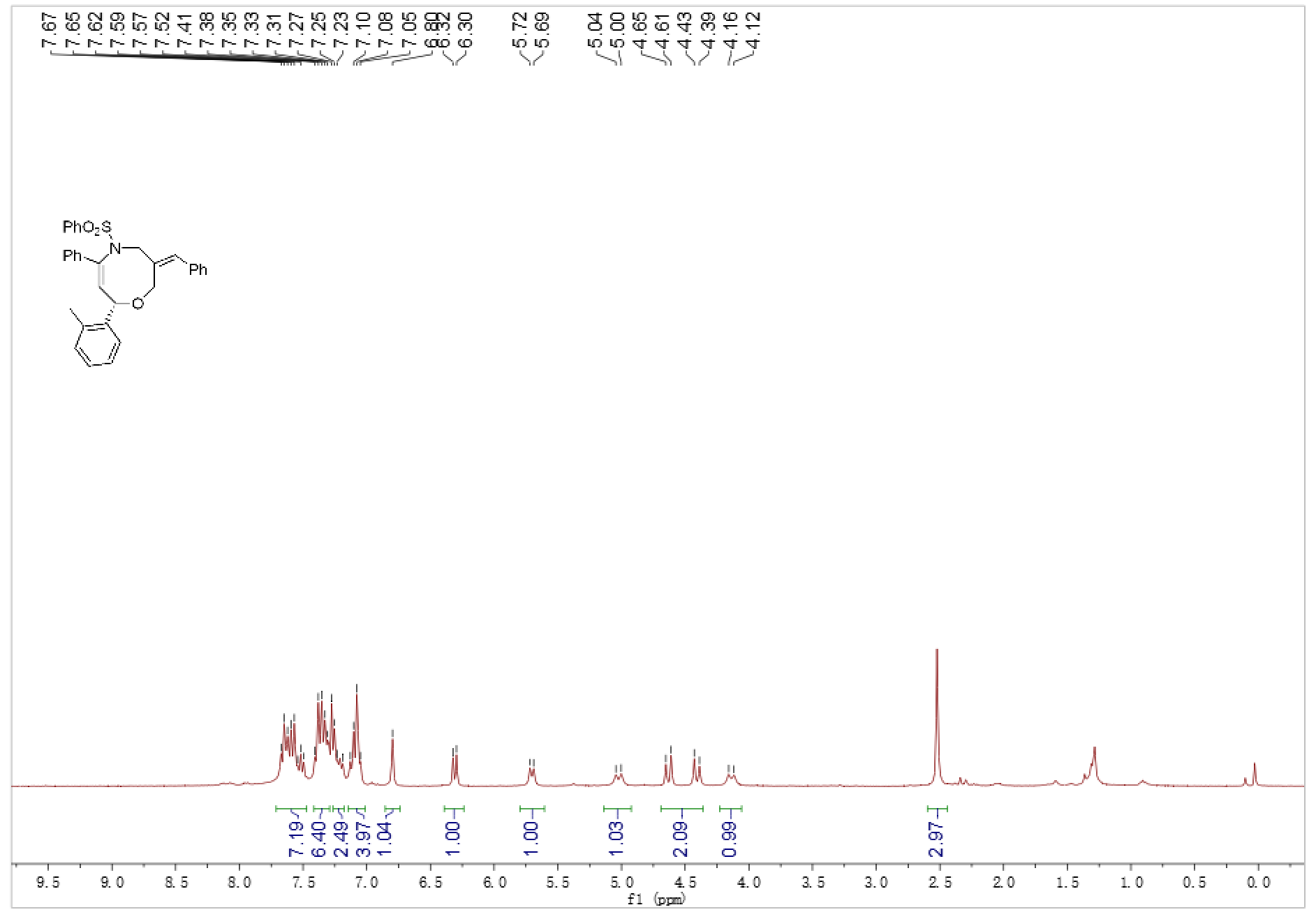
**

^1^H (CDCl_3_, 300 MHz) NMR of compound **37**


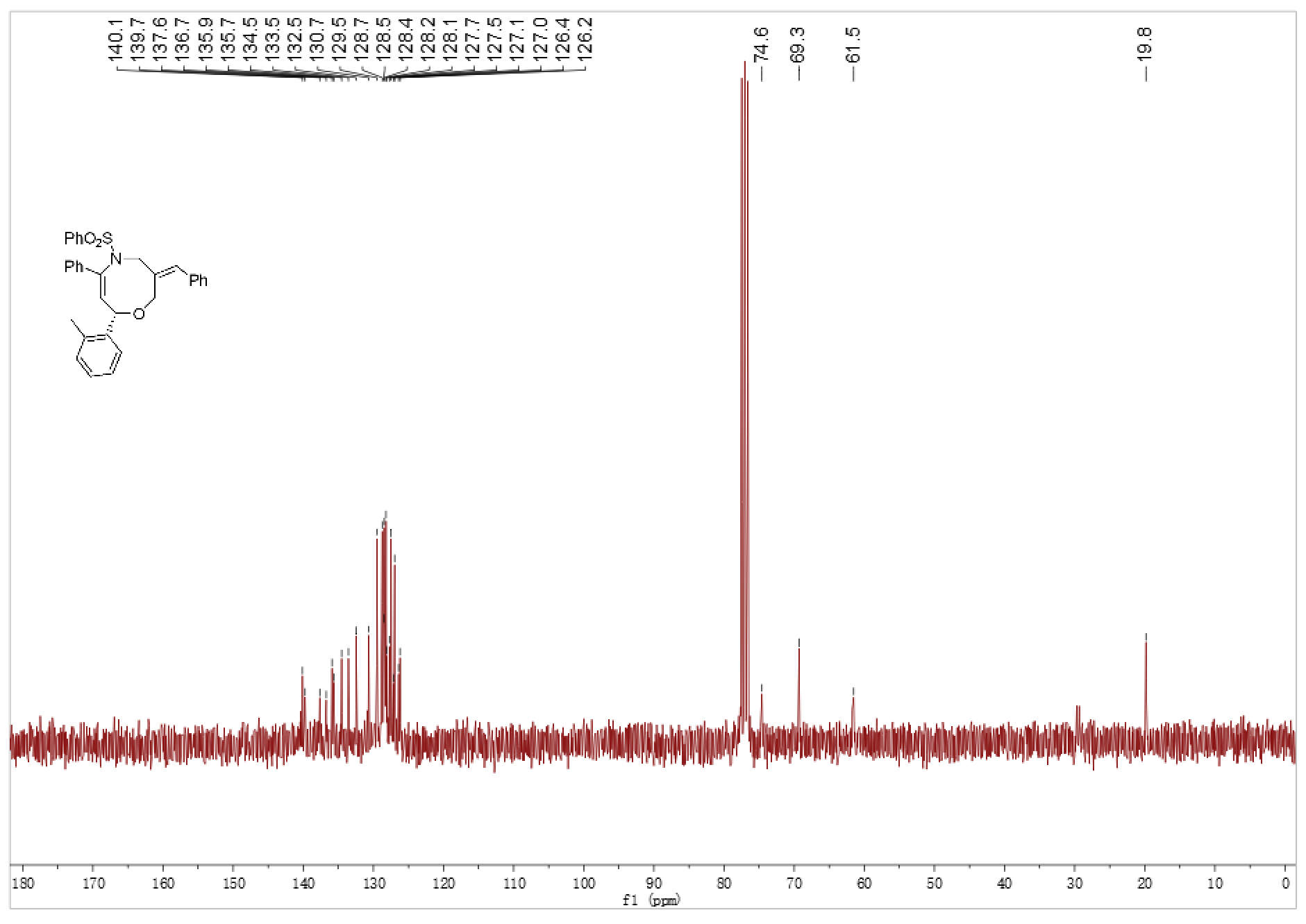


^13^C (CDCl_3_, 75 MHz) NMR of compound **37**

**
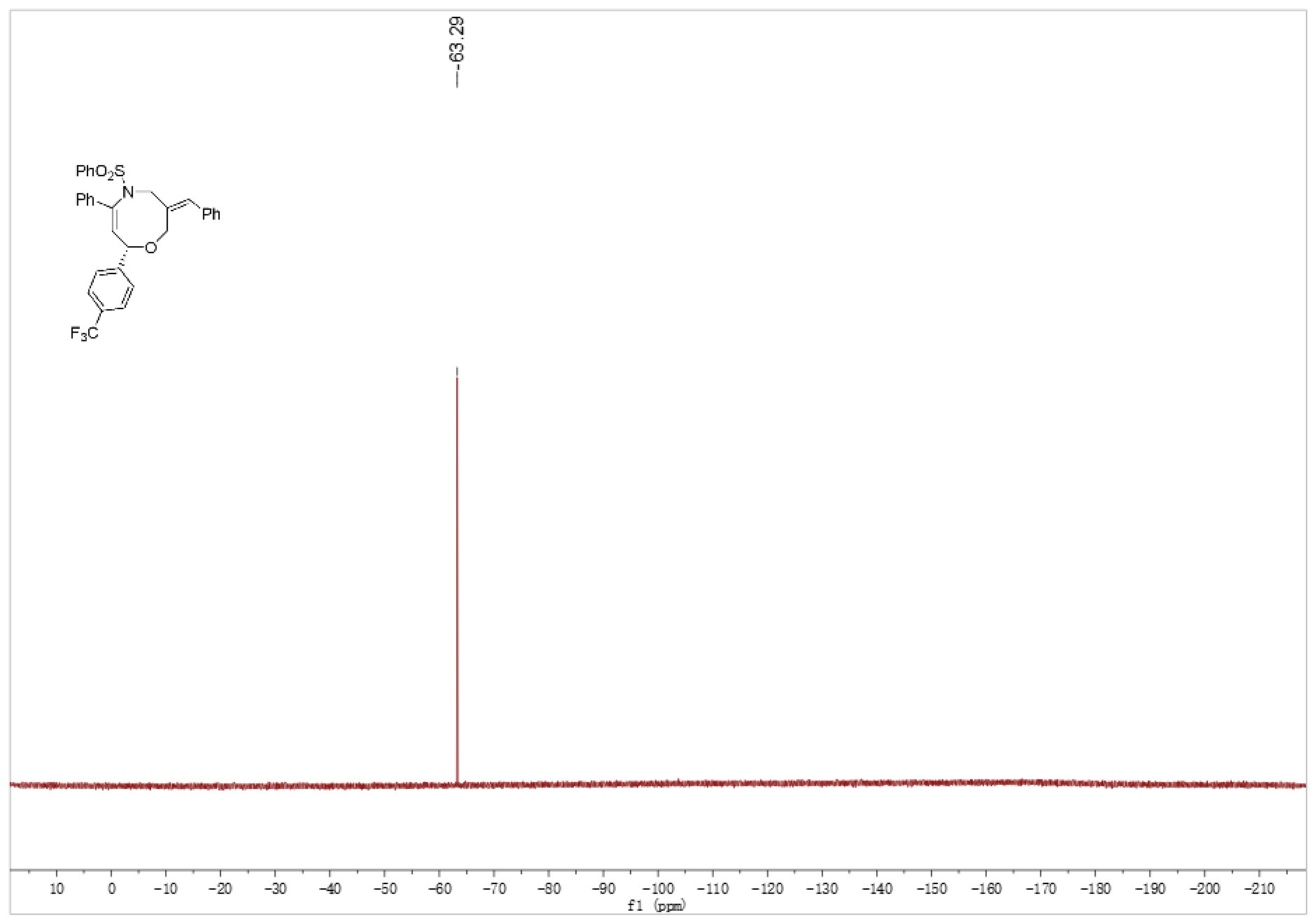
**

^19^F (CDCl_3_, 282 MHz) NMR of compound **38**

^1^H (CDCl_3_, 300 MHz) NMR of compound **38**

^13^C (CDCl_3_, 75 MHz) NMR of compound **38**

^1^H (CDCl_3_, 300 MHz) NMR of compound **39**

^13^C (CDCl_3_, 75 MHz) NMR of compound **39**

^19^F (CDCl_3_, 282 MHz) NMR of compound **40**

^1^H (CDCl_3_, 300 MHz) NMR of compound **40**

^13^C (CDCl_3_, 75 MHz) NMR of compound **40**

^19^F (CDCl_3_, 282 MHz) NMR of compound **41**

^1^H (CDCl_3_, 300 MHz) NMR of compound **41**

^13^C (CDCl_3_, 75 MHz) NMR of compound **41**

^19^F (CDCl_3_, 282 MHz) NMR of compound **42**

^1^H (CDCl_3_, 300 MHz) NMR of compound **42**

^13^C (CDCl_3_, 75 MHz) NMR of compound **42**

^1^H (CDCl_3_, 300 MHz) NMR of compound **43**

^13^C (CDCl_3_, 75 MHz) NMR of compound **43**

^1^H (CDCl_3_, 300 MHz) NMR of compound **44**

^13^C (CDCl_3_, 75 MHz) NMR of compound **44**

^1^H (CDCl_3_, 300 MHz) NMR of compound **45**

^13^C (CDCl_3_, 75 MHz) NMR of compound **45**

^1^H (CDCl_3_, 300 MHz) NMR of compound **46**

^13^C (CDCl_3_, 75 MHz) NMR of compound **46**

^1^H (CDCl_3_, 300 MHz) NMR of compound **47**

^13^C (CDCl_3_, 75 MHz) NMR of compound **47**

^19^F (CDCl_3_, 282 MHz) NMR of compound **48**

^1^H (CDCl_3_, 300 MHz) NMR of compound **48**

^13^C (CDCl_3_, 75 MHz) NMR of compound **48**

^1^H (CDCl_3_, 300 MHz) NMR of compound **49**

^13^C (CDCl_3_, 75 MHz) NMR of compound **49**

^1^H (CDCl_3_, 300 MHz) NMR of compound **50**

^13^C (CDCl_3_, 75 MHz) NMR of compound **50**

^1^H (CDCl_3_, 300 MHz) NMR of compound **51**

^13^C (CDCl_3_, 75 MHz) NMR of compound **51**

^19^F (CDCl_3_, 282 MHz) NMR of compound **52**

^1^H (CDCl_3_, 300 MHz) NMR of compound **52**

^13^C (CDCl_3_, 75 MHz) NMR of compound **52**

^1^H (CDCl_3_, 300 MHz) NMR of compound **53**

^13^C (CDCl_3_, 75 MHz) NMR of compound **53**

^1^H (CDCl_3_, 300 MHz) NMR of compound **54**

^13^C (CDCl_3_, 75 MHz) NMR of compound **54**

^19^F (CDCl_3_, 282 MHz) NMR of compound **55**

^1^H (CDCl_3_, 300 MHz) NMR of compound **55**

^13^C (CDCl_3_, 75 MHz) NMR of compound **55**

^1^H (CDCl_3_, 300 MHz) NMR of compound **56**

^13^C (CDCl_3_, 75 MHz) NMR of compound **56**

^1^H (CDCl_3_, 600 MHz) NMR of compound **59**

^13^C (CDCl_3_, 151 MHz) NMR of compound **59**

^1^H (CDCl_3_, 600 MHz) NMR of compound **60**

^13^C (CDCl_3_, 151 MHz) NMR of compound **60**

# **6. Copies of HPLC Chromatograms**

Discloser A 254 nm

| Peak | Retention time (min) | Area (%) |
| --- | --- | --- |
| 1 | 17.578 | 49.108 |
| 2 | 19.171 | 50.892 |
| total |  | 100 |

Discloser A 254 nm

| Peak | Retention time (min) | Area (%) |
| --- | --- | --- |
| 1 | 16.694 | 97.120 |
| 2 | 19.330 | 2.880 |
| total |  | 100 |

Discloser A 254 nm

| Peak | Retention time (min) | Area (%) |
| --- | --- | --- |
| 1 | 13.519 | 49.883 |
| 2 | 15.264 | 50.117 |
| total |  | 100 |

Discloser A 254 nm

| Peak | Retention time (min) | Area (%) |
| --- | --- | --- |
| 1 | 11.891 | 86.471 |
| 2 | 13.801 | 13.529 |
| total |  | 100 |

Discloser A 254 nm

| Peak | Retention time (min) | Area (%) |
| --- | --- | --- |
| 1 | 13.784 | 49.084 |
| 2 | 18.901 | 50.916 |
| total |  | 100 |

Discloser A 254 nm

| Peak | Retention time (min) | Area (%) |
| --- | --- | --- |
| 1 | 13.849 | 96.856 |
| 2 | 19.473 | 3.144 |
| total |  | 100 |

Discloser A 254 nm

| Peak | Retention time (min) | Area (%) |
| --- | --- | --- |
| 1 | 15.836 | 49.713 |
| 2 | 17.602 | 50.287 |
| total |  | 100 |

Discloser A 254 nm

| Peak | Retention time (min) | Area (%) |
| --- | --- | --- |
| 1 | 15.875 | 93.568 |
| 2 | 17.795 | 6.432 |
| total |  | 100 |

Discloser A 254 nm

| Peak | Retention time (min) | Area (%) |
| --- | --- | --- |
| 1 | 18.237 | 50.300 |
| 2 | 24.374 | 49.700 |
| total |  | 100 |

Discloser A 254 nm

| Peak | Retention time (min) | Area (%) |
| --- | --- | --- |
| 1 | 18.145 | 95.413 |
| 2 | 24.580 | 4.587 |
| total |  | 100 |

Discloser A 254 nm

| Peak | Retention time (min) | Area (%) |
| --- | --- | --- |
| 1 | 19.399 | 50.107 |
| 2 | 28.722 | 49.893 |
| total |  | 100 |

Discloser A 254 nm

| Peak | Retention time (min) | Area (%) |
| --- | --- | --- |
| 1 | 19.657 | 95.080 |
| 2 | 29.480 | 4.920 |
| total |  | 100 |

Discloser A 254 nm

| Peak | Retention time (min) | Area (%) |
| --- | --- | --- |
| 1 | 17.419 | 50.715 |
| 2 | 22.448 | 49.285 |
| total |  | 100 |

Discloser A 254 nm

| Peak | Retention time (min) | Area (%) |
| --- | --- | --- |
| 1 | 17.621 | 95.873 |
| 2 | 22.744 | 4.127 |
| total |  | 100 |

Discloser A 254 nm

| Peak | Retention time (min) | Area (%) |
| --- | --- | --- |
| 1 | 17.300 | 49.733 |
| 2 | 20.146 | 50.267 |
| total |  | 100 |

Discloser A 254 nm

| Peak | Retention time (min) | Area (%) |
| --- | --- | --- |
| 1 | 17.294 | 95.383 |
| 2 | 20.455 | 4.617 |
| total |  | 100 |

Discloser A 254 nm

| Peak | Retention time (min) | Area (%) |
| --- | --- | --- |
| 1 | 19.699 | 49.750 |
| 2 | 29.492 | 50.250 |
| total |  | 100 |

Discloser A 254 nm

| Peak | Retention time (min) | Area (%) |
| --- | --- | --- |
| 1 | 19.761 | 96.994 |
| 2 | 20.527 | 3.006 |
| total |  | 100 |

Discloser A 254 nm

| Peak | Retention time (min) | Area (%) |
| --- | --- | --- |
| 1 | 11.827 | 49.293 |
| 2 | 15.427 | 50.707 |
| total |  | 100 |

Discloser A 254 nm

| Peak | Retention time (min) | Area (%) |
| --- | --- | --- |
| 1 | 11.865 | 94.950 |
| 2 | 15.599 | 5.050 |
| total |  | 100 |

Discloser A 254 nm

| Peak | Retention time (min) | Area (%) |
| --- | --- | --- |
| 1 | 16.991 | 49.879 |
| 2 | 19.567 | 50.121 |
| total |  | 100 |

Discloser A 254 nm

| Peak | Retention time (min) | Area (%) |
| --- | --- | --- |
| 1 | 17.350 | 95.738 |
| 2 | 20.451 | 4.262 |
| total |  | 100 |

Discloser A 254 nm

| Peak | Retention time (min) | Area (%) |
| --- | --- | --- |
| 1 | 21.692 | 49.679 |
| 2 | 29.462 | 50.321 |
| total |  | 100 |

Discloser A 254 nm

| Peak | Retention time (min) | Area (%) |
| --- | --- | --- |
| 1 | 22.137 | 96.364 |
| 2 | 30.650 | 3.636 |
| total |  | 100 |

Discloser A 254 nm

| Peak | Retention time (min) | Area (%) |
| --- | --- | --- |
| 1 | 21.340 | 49.937 |
| 2 | 27.368 | 50.063 |
| total |  | 100 |

Discloser A 254 nm

| Peak | Retention time (min) | Area (%) |
| --- | --- | --- |
| 1 | 18.922 | 96.430 |
| 2 | 25.505 | 3.570 |
| total |  | 100 |

Discloser A 254 nm

| Peak | Retention time (min) | Area (%) |
| --- | --- | --- |
| 1 | 12.877 | 51.146 |
| 2 | 16.401 | 48.854 |
| total |  | 100 |

Discloser A 254 nm

| Peak | Retention time (min) | Area (%) |
| --- | --- | --- |
| 1 | 13.025 | 0.875 |
| 2 | 16.928 | 99.125 |
| total |  | 100 |

Discloser A 254 nm

| Peak | Retention time (min) | Area (%) |
| --- | --- | --- |
| 1 | 53.937 | 50.899 |
| 2 | 62.283 | 49.101 |
| total |  | 100 |

Discloser A 254 nm

| Peak | Retention time (min) | Area (%) |
| --- | --- | --- |
| 1 | 53.425 | 6.537 |
| 2 | 59.764 | 93.463 |
| total |  | 100 |

Discloser A 254 nm

| Peak | Retention time (min) | Area (%) |
| --- | --- | --- |
| 1 | 30.914 | 49.559 |
| 2 | 38.329 | 50.441 |
| total |  | 100 |

Discloser A 254 nm

| Peak | Retention time (min) | Area (%) |
| --- | --- | --- |
| 1 | 30.077 | 97.110 |
| 2 | 37.870 | 2.890 |
| total |  | 100 |

Discloser A 254 nm

| Peak | Retention time (min) | Area (%) |
| --- | --- | --- |
| 1 | 29.666 | 50.054 |
| 2 | 34.357 | 49.946 |
| total |  | 100 |

Discloser A 254 nm

| Peak | Retention time (min) | Area (%) |
| --- | --- | --- |
| 1 | 30.921 | 96.482 |
| 2 | 36.721 | 3.518 |
| total |  | 100 |

Discloser A 254 nm

| Peak | Retention time (min) | Area (%) |
| --- | --- | --- |
| 1 | 32.537 | 49.585 |
| 2 | 37.198 | 50.415 |
| total |  | 100 |

Discloser A 254 nm

| Peak | Retention time (min) | Area (%) |
| --- | --- | --- |
| 1 | 30.578 | 96.464 |
| 2 | 36.608 | 3.536 |
| total |  | 100 |

Discloser A 254 nm

| Peak | Retention time (min) | Area (%) |
| --- | --- | --- |
| 1 | 31.263 | 50.192 |
| 2 | 43.619 | 49.808 |
| total |  | 100 |

Discloser A 254 nm

| Peak | Retention time (min) | Area (%) |
| --- | --- | --- |
| 1 | 29.889 | 95.515 |
| 2 | 42.928 | 4.485 |
| total |  | 100 |

Discloser A 254 nm

| Peak | Retention time (min) | Area (%) |
| --- | --- | --- |
| 1 | 24.384 | 50.105 |
| 2 | 30.744 | 49.895 |
| total |  | 100 |

Discloser A 254 nm

| Peak | Retention time (min) | Area (%) |
| --- | --- | --- |
| 1 | 23.787 | 94.772 |
| 2 | 30.737 | 5.228 |
| total |  | 100 |

Discloser A 254 nm

| Peak | Retention time (min) | Area (%) |
| --- | --- | --- |
| 1 | 24.297 | 50.117 |
| 2 | 30.248 | 49.883 |
| total |  | 100 |

Discloser A 254 nm

| Peak | Retention time (min) | Area (%) |
| --- | --- | --- |
| 1 | 24.405 | 97.003 |
| 2 | 31.001 | 2.997 |
| total |  | 100 |

Discloser A 254 nm

| Peak | Retention time (min) | Area (%) |
| --- | --- | --- |
| 1 | 19.353 | 49.960 |
| 2 | 24.521 | 50.040 |
| total |  | 100 |

Discloser A 254 nm

| Peak | Retention time (min) | Area (%) |
| --- | --- | --- |
| 1 | 19.466 | 94.829 |
| 2 | 25.384 | 5.171 |
| total |  | 100 |

Discloser A 254 nm

| Peak | Retention time (min) | Area (%) |
| --- | --- | --- |
| 1 | 17.767 | 52.552 |
| 2 | 23.860 | 47.448 |
| total |  | 100 |

Discloser A 254 nm

| Peak | Retention time (min) | Area (%) |
| --- | --- | --- |
| 1 | 17.574 | 95.469 |
| 2 | 24.120 | 4.531 |
| total |  | 100 |

Discloser A 254 nm

| Peak | Retention time (min) | Area (%) |
| --- | --- | --- |
| 1 | 29.889 | 48.872 |
| 2 | 36.900 | 51.128 |
| total |  | 100 |

Discloser A 254 nm

| Peak | Retention time (min) | Area (%) |
| --- | --- | --- |
| 1 | 29.217 | 95.435 |
| 2 | 36.565 | 4.565 |
| total |  | 100 |

Discloser A 254 nm

| Peak | Retention time (min) | Area (%) |
| --- | --- | --- |
| 1 | 12.127 | 49.944 |
| 2 | 17.020 | 50.056 |
| total |  | 100 |

Discloser A 254 nm

| Peak | Retention time (min) | Area (%) |
| --- | --- | --- |
| 1 | 12.008 | 10.814 |
| 2 | 17.684 | 89.186 |
| total |  | 100 |

Discloser A 254 nm

| Peak | Retention time (min) | Area (%) |
| --- | --- | --- |
| 1 | 36.788 | 48.826 |
| 2 | 41.978 | 51.174 |
| total |  | 100 |

Discloser A 254 nm

| Peak | Retention time (min) | Area (%) |
| --- | --- | --- |
| 1 | 35.952 | 95.336 |
| 2 | 42.456 | 4.664 |
| total |  | 100 |

Discloser A 254 nm

| Peak | Retention time (min) | Area (%) |
| --- | --- | --- |
| 1 | 38.997 | 48.061 |
| 2 | 45.154 | 51.939 |
| total |  | 100 |

Discloser A 254 nm

| Peak | Retention time (min) | Area (%) |
| --- | --- | --- |
| 1 | 38.971 | 96.862 |
| 2 | 46.646 | 3.138 |
| total |  | 100 |

Discloser A 254 nm

| Peak | Retention time (min) | Area (%) |
| --- | --- | --- |
| 1 | 14.428 | 49.950 |
| 2 | 17.229 | 50.050 |
| total |  | 100 |

Discloser A 254 nm

| Peak | Retention time (min) | Area (%) |
| --- | --- | --- |
| 1 | 14.367 | 4.388 |
| 2 | 16.621 | 95.612 |
| total |  | 100 |

Discloser A 254 nm

| Peak | Retention time (min) | Area (%) |
| --- | --- | --- |
| 1 | 13.845 | 49.709 |
| 2 | 17.571 | 50.291 |
| total |  | 100 |

Discloser A 254 nm

| Peak | Retention time (min) | Area (%) |
| --- | --- | --- |
| 1 | 13.727 | 93.973 |
| 2 | 17.716 | 6.027 |
| total |  | 100 |

Discloser A 254 nm

| Peak | Retention time (min) | Area (%) |
| --- | --- | --- |
| 1 | 25.240 | 49.184 |
| 2 | 28.641 | 50.816 |
| total |  | 100 |

Discloser A 254 nm

| Peak | Retention time (min) | Area (%) |
| --- | --- | --- |
| 1 | 25.014 | 94.567 |
| 2 | 28.612 | 5.433 |
| total |  | 100 |

Discloser A 254 nm

| Peak | Retention time (min) | Area (%) |
| --- | --- | --- |
| 1 | 23.166 | 50.018 |
| 2 | 28.991 | 49.982 |
| total |  | 100 |

Discloser A 254 nm

| Peak | Retention time (min) | Area (%) |
| --- | --- | --- |
| 1 | 23.135 | 98.048 |
| 2 | 29.306 | 1.952 |
| total |  | 100 |

Discloser A 254 nm

| Peak | Retention time (min) | Area (%) |
| --- | --- | --- |
| 1 | 21.663 | 48.862 |
| 2 | 23.149 | 51.138 |
| total |  | 100 |

Discloser A 254 nm

| Peak | Retention time (min) | Area (%) |
| --- | --- | --- |
| 1 | 21.892 | 3.053 |
| 2 | 22.942 | 96.947 |
| total |  | 100 |

Discloser A 254 nm

| Peak | Retention time (min) | Area (%) |
| --- | --- | --- |
| 1 | 26.379 | 51.904 |
| 2 | 33.942 | 48.096 |
| total |  | 100 |

Discloser A 254 nm

| Peak | Retention time (min) | Area (%) |
| --- | --- | --- |
| 1 | 26.669 | 92.736 |
| 2 | 34.777 | 7.264 |
| total |  | 100 |

Discloser A 254 nm

| Peak | Retention time (min) | Area (%) |
| --- | --- | --- |
| 1 | 12.796 | 49.787 |
| 2 | 13.981 | 50.213 |
| total |  | 100 |

Discloser A 254 nm

| Peak | Retention time (min) | Area (%) |
| --- | --- | --- |
| 1 | 12.788 | 98.826 |
| 2 | 13.914 | 1.174 |
| total |  | 100 |

Discloser A 254 nm

| Peak | Retention time (min) | Area (%) |
| --- | --- | --- |
| 1 | 29.481 | 49.790 |
| 2 | 33.802 | 50.210 |
| total |  | 100 |

Discloser A 254 nm

| Peak | Retention time (min) | Area (%) |
| --- | --- | --- |
| 1 | 29.493 | 96.294 |
| 2 | 34.206 | 3.706 |
| total |  | 100 |

Discloser A 254 nm

| Peak | Retention time (min) | Area (%) |
| --- | --- | --- |
| 1 | 59.289 | 50.959 |
| 2 | 69.000 | 49.041 |
| total |  | 100 |

Discloser A 254 nm

| Peak | Retention time (min) | Area (%) |
| --- | --- | --- |
| 1 | 58.433 | 95.943 |
| 2 | 69.910 | 4.057 |
| total |  | 100 |

Discloser A 254 nm

| Peak | Retention time (min) | Area (%) |
| --- | --- | --- |
| 1 | 19.536 | 49.895 |
| 2 | 21.453 | 50.105 |
| total |  | 100 |

Discloser A 254 nm

| Peak | Retention time (min) | Area (%) |
| --- | --- | --- |
| 1 | 19.077 | 96.221 |
| 2 | 21.479 | 3.779 |
| total |  | 100 |

Discloser A 254 nm

| Peak | Retention time (min) | Area (%) |
| --- | --- | --- |
| 1 | 12.979 | 47.535 |
| 2 | 15.341 | 52.465 |
| total |  | 100 |

Discloser A 254 nm

| Peak | Retention time (min) | Area (%) |
| --- | --- | --- |
| 1 | 12.842 | 96.809 |
| 2 | 15.423 | 3.191 |
| total |  | 100 |

Discloser A 254 nm

| Peak | Retention time (min) | Area (%) |
| --- | --- | --- |
| 1 | 34.051 | 50.273 |
| 2 | 58.971 | 49.727 |
| total |  | 100 |

Discloser A 254 nm

| Peak | Retention time (min) | Area (%) |
| --- | --- | --- |
| 1 | 34.172 | 99.998 |
| 2 | 58.913 | 0.002 |
| total |  | 100 |

Discloser A 254 nm

| Peak | Retention time (min) | Area (%) |
| --- | --- | --- |
| 1 | 33.541 | 50.424 |
| 2 | 51.748 | 49.576 |
| total |  | 100 |

Discloser A 254 nm

| Peak | Retention time (min) | Area (%) |
| --- | --- | --- |
| 1 | 32.726 | 97.030 |
| 2 | 52.394 | 2.970 |
| total |  | 100 |

Discloser A 254 nm

| Peak | Retention time (min) | Area (%) |
| --- | --- | --- |
| 1 | 35.308 | 49.866 |
| 2 | 55.374 | 50.134 |
| total |  | 100 |

Discloser A 254 nm

| Peak | Retention time (min) | Area (%) |
| --- | --- | --- |
| 1 | 34.267 | 97.000 |
| 2 | 56.116 | 3.000 |
| total |  | 100 |

Discloser A 254 nm

| Peak | Retention time (min) | Area (%) |
| --- | --- | --- |
| 1 | 13.451 | 50.332 |
| 2 | 16.207 | 49.668 |
| total |  | 100 |

Discloser A 254 nm

| Peak | Retention time (min) | Area (%) |
| --- | --- | --- |
| 1 | 13.376 | 93.241 |
| 2 | 16.238 | 6.759 |
| total |  | 100 |

Discloser A 254 nm

| Peak | Retention time (min) | Area (%) |
| --- | --- | --- |
| 1 | 22.960 | 49.712 |
| 2 | 31.611 | 50.288 |
| total |  | 100 |

Discloser A 254 nm

| Peak | Retention time (min) | Area (%) |
| --- | --- | --- |
| 1 | 22.710 | 96.066 |
| 2 | 31.540 | 3.934 |
| total |  | 100 |

Discloser A 254 nm

| Peak | Retention time (min) | Area (%) |
| --- | --- | --- |
| 1 | 33.780 | 47.596 |
| 2 | 53.258 | 52.404 |
| total |  | 100 |

Discloser A 254 nm

| Peak | Retention time (min) | Area (%) |
| --- | --- | --- |
| 1 | 34.248 | 1.686 |
| 2 | 53.847 | 98.314 |
| total |  | 100 |

Discloser A 254 nm

| Peak | Retention time (min) | Area (%) |
| --- | --- | --- |
| 1 | 14.721 | 51.397 |
| 2 | 16.853 | 48.603 |
| total |  | 100 |

Discloser A 254 nm

| Peak | Retention time (min) | Area (%) |
| --- | --- | --- |
| 1 | 14.843 | 5.709 |
| 2 | 16.650 | 94.291 |
| total |  | 100 |

Discloser A 254 nm

| Peak | Retention time (min) | Area (%) |
| --- | --- | --- |
| 1 | 15.167 | 50.263 |
| 2 | 18.745 | 49.737 |
| total |  | 100 |

Discloser A 254 nm

| Peak | Retention time (min) | Area (%) |
| --- | --- | --- |
| 1 | 15.150 | 4.677 |
| 2 | 18.326 | 95.323 |
| total |  | 100 |

Discloser A 254 nm

| Peak | Retention time (min) | Area (%) |
| --- | --- | --- |
| 1 | 22.905 | 49.913 |
| 2 | 40.216 | 50.087 |
| total |  | 100 |

Discloser A 254 nm

| Peak | Retention time (min) | Area (%) |
| --- | --- | --- |
| 1 | 22.387 | 97.446 |
| 2 | 40.280 | 2.554 |
| total |  | 100 |

Discloser A 254 nm

| Peak | Retention time (min) | Area (%) |
| --- | --- | --- |
| 1 | 14.886 | 51.271 |
| 2 | 18.197 | 48.729 |
| total |  | 100 |

Discloser A 254 nm

| Peak | Retention time (min) | Area (%) |
| --- | --- | --- |
| 1 | 15.001 | 5.316 |
| 2 | 18.143 | 94.684 |
| total |  | 100 |

Discloser A 254 nm

| Peak | Retention time (min) | Area (%) |
| --- | --- | --- |
| 1 | 33.617 | 49.487 |
| 2 | 48.304 | 50.513 |
| total |  | 100 |

Discloser A 254 nm

| Peak | Retention time (min) | Area (%) |
| --- | --- | --- |
| 1 | 31.635 | 95.500 |
| 2 | 47.033 | 4.500 |
| total |  | 100 |

Discloser A 254 nm

| Peak | Retention time (min) | Area (%) |
| --- | --- | --- |
| 1 | 23.343 | 49.168 |
| 2 | 29.026 | 50.832 |
| total |  | 100 |

Discloser A 254 nm

| Peak | Retention time (min) | Area (%) |
| --- | --- | --- |
| 1 | 22.589 | 94.915 |
| 2 | 28.587 | 5.085 |
| total |  | 100 |

Discloser A 254 nm

| Peak | Retention time (min) | Area (%) |
| --- | --- | --- |
| 1 | 35.008 | 48.426 |
| 2 | 60.601 | 51.574 |
| total |  | 100 |

Discloser A 254 nm

| Peak | Retention time (min) | Area (%) |
| --- | --- | --- |
| 1 | 34.355 | 87.739 |
| 2 | 60.999 | 12.261 |
| total |  | 100 |

Discloser A 254 nm

| Peak | Retention time (min) | Area (%) |
| --- | --- | --- |
| 1 | 10.486 | 50.527 |
| 2 | 13.581 | 49.473 |
| total |  | 100 |

Discloser A 254 nm

| Peak | Retention time (min) | Area (%) |
| --- | --- | --- |
| 1 | 10.526 | 5.280 |
| 2 | 13.476 | 94.720 |
| total |  | 100 |

Discloser A 254 nm

| Peak | Retention time (min) | Area (%) |
| --- | --- | --- |
| 1 | 21.891 | 49.662 |
| 2 | 26.530 | 50.338 |
| total |  | 100 |

Discloser A 254 nm

| Peak | Retention time (min) | Area (%) |
| --- | --- | --- |
| 1 | 21.489 | 95.496 |
| 2 | 26.833 | 4.504 |
| total |  | 100 |

Discloser A 254 nm

| Peak | Retention time (min) | Area (%) |
| --- | --- | --- |
| 1 | 25.533 | 50.044 |
| 2 | 28.332 | 49.956 |
| total |  | 100 |

Discloser A 254 nm

| Peak | Retention time (min) | Area (%) |
| --- | --- | --- |
| 1 | 27.825 | 95.001 |
| 2 | 30.408 | 4.999 |
| total |  | 100 |

Discloser A 254 nm

| Peak | Retention time (min) | Area (%) |
| --- | --- | --- |
| 1 | 37.496 | 48.255 |
| 2 | 46.281 | 51.745 |
| total |  | 100 |

Discloser A 254 nm

| Peak | Retention time (min) | Area (%) |
| --- | --- | --- |
| 1 | 39.038 | 94.749 |
| 2 | 48.954 | 5.251 |
| total |  | 100 |

Discloser A 254 nm

| Peak | Retention time (min) | Area (%) |
| --- | --- | --- |
| 1 | 12.783 | 49.656 |
| 2 | 19.208 | 50.344 |
| total |  | 100 |

Discloser A 254 nm

| Peak | Retention time (min) | Area (%) |
| --- | --- | --- |
| 1 | 12.828 | 6.672 |
| 2 | 18.832 | 93.328 |
| total |  | 100 |

**7. X-ray crystal structures**

The X-ray crystallographic structures for **MQ Phos-1**, **3**, **42**. ORTEP view of the molecules of complex **MQ Phos-1**, **3**, **42**, showing ellipsoids at 30% probability level. Crystal data have been deposited to CCDC, number **MQ Phos-1** (2284070), **3** (2158695), **42** (2284069). A summary of the fundamental crystal and refinement data are given in the Table S1 of the Supporting Information. Atomic coordinates, anisotropic displacement parameters and bond lengths and angles can be found in the cif files.

Crystals suitable for X-ray diffraction (Gemini E) were grown by n-hexane/ethyl acetate solution of **MQ Phos-1**, **3**, **42** inside a penicillin bottle.

Crystal structure of **MQ Phos-1** **(2284070)**

**Table S5 Crystal data and structure refinement for** **MQ Phos-1.**

| Identification code | MQ Phos-1 |
| --- | --- |
| Empirical formula | C_59_H_57_NOP_2_ |
| Formula weight | 857.99 |
| Temperature/K | 293(2) |
| Crystal system | triclinic |
| Space group | P-1 |
| a/Å | 12.13260(19) |
| b/Å | 13.0770(2) |
| c/Å | 19.4768(3) |
| α/° | 89.1457(13) |
| β/° | 72.5669(14) |
| γ/° | 70.9255(14) |
| Volume/Å^3^ | 2774.51(8) |
| Z | 2 |
| ρ_calc_g/cm^3^ | 1.027 |
| μ/mm^‑1^ | 0.979 |
| F(000) | 912.0 |
| Crystal size/mm^3^ | 0.21 × 0.1 × 0.07 |
| Radiation | CuKα (λ = 1.54184) |
| 2Θ range for data collection/° | 7.184 to 134.144 |
| Index ranges | -12 ≤ h ≤ 14, -15 ≤ k ≤ 15, -23 ≤ l ≤ 23 |
| Reflections collected | 40776 |
| Independent reflections | 9919 [R_int_ = 0.0334, R_sigma_ = 0.0251] |
| Data/restraints/parameters | 9919/0/610 |
| Goodness-of-fit on F^2^ | 1.037 |
| Final R indexes [I>=2σ (I)] | R_1_ = 0.0438, wR_2_ = 0.1233 |
| Final R indexes [all data] | R_1_ = 0.0529, wR_2_ = 0.1308 |
| Largest diff. peak/hole / e Å^-3^ | 0.24/-0.21 |

Crystal structure of **3** **(2158695)**

**Table S6 Crystal data and structure refinement for 3.**

| Identification code | 3 |
| --- | --- |
| Empirical formula | C_31_H_27_NO_3_S |
| Formula weight | 493.59 |
| Temperature/K | 293(2) |
| Crystal system | orthorhombic |
| Space group | P2_1_2_1_2_1_ |
| a/Å | 9.4879(4) |
| b/Å | 10.7822(4) |
| c/Å | 24.8589(9) |
| α/° | 90 |
| β/° | 90 |
| γ/° | 90 |
| Volume/Å^3^ | 2543.10(17) |
| Z | 4 |
| ρ_calc_g/cm^3^ | 1.289 |
| μ/mm^‑1^ | 1.394 |
| F(000) | 1040.0 |
| Crystal size/mm^3^ | 0.17 × 0.13 × 0.1 |
| Radiation | CuKα (λ = 1.54184) |
| 2Θ range for data collection/° | 7.112 to 142.092 |
| Index ranges | -11 ≤ h ≤ 11, -12 ≤ k ≤ 12, -26 ≤ l ≤ 30 |
| Reflections collected | 10681 |
| Independent reflections | 4790 [R_int_ = 0.0343, R_sigma_ = 0.0451] |
| Data/restraints/parameters | 4790/0/325 |
| Goodness-of-fit on F^2^ | 1.065 |
| Final R indexes [I>=2σ (I)] | R_1_ = 0.0481, wR_2_ = 0.1233 |
| Final R indexes [all data] | R_1_ = 0.0571, wR_2_ = 0.1338 |
| Largest diff. peak/hole / e Å^-3^ | 0.16/-0.23 |
| Flack parameter | 0.005(19) |

Crystal structure of **42** **(2284069)**

**Table S7 Crystal data and structure refinement for 42.**

| Identification code | 42 |
| --- | --- |
| Empirical formula | C_31_H_26_FNO_3_S |
| Formula weight | 511.59 |
| Temperature/K | 293(2) |
| Crystal system | orthorhombic |
| Space group | P2_1_2_1_2_1_ |
| a/Å | 9.84863(18) |
| b/Å | 10.26608(18) |
| c/Å | 25.8675(5) |
| α/° | 90 |
| β/° | 90 |
| γ/° | 90 |
| Volume/Å^3^ | 2615.38(9) |
| Z | 4 |
| ρ_calc_g/cm^3^ | 1.299 |
| μ/mm^‑1^ | 1.431 |
| F(000) | 1072.0 |
| Crystal size/mm^3^ | 0.16 × 0.12 × 0.1 |
| Radiation | CuKα (λ = 1.54184) |
| 2Θ range for data collection/° | 6.834 to 140.89 |
| Index ranges | -12 ≤ h ≤ 11, -7 ≤ k ≤ 12, -31 ≤ l ≤ 30 |
| Reflections collected | 9530 |
| Independent reflections | 4897 [R_int_ = 0.0270, R_sigma_ = 0.0447] |
| Data/restraints/parameters | 4897/0/334 |
| Goodness-of-fit on F^2^ | 1.045 |
| Final R indexes [I>=2σ (I)] | R_1_ = 0.0455, wR_2_ = 0.1115 |
| Final R indexes [all data] | R_1_ = 0.0532, wR_2_ = 0.1188 |
| Largest diff. peak/hole / e Å^-3^ | 0.16/-0.25 |
| Flack parameter | 0.002(16) |
